# Three-Component 1,2-Dioxygenation of 1,3-Dienes Using Carboxylic Acids and TEMPO

**DOI:** 10.1021/acs.joc.4c02244

**Published:** 2024-11-04

**Authors:** Sophia M. Baldassarre, Heidi S. Sato, Adam P. Louise, Layna L. Summer, Benjamin P. Wilson, Brett N. Hemric

**Affiliations:** Department of Chemistry and Biochemistry, University of Tampa, Tampa, Florida 33606, United States

## Abstract

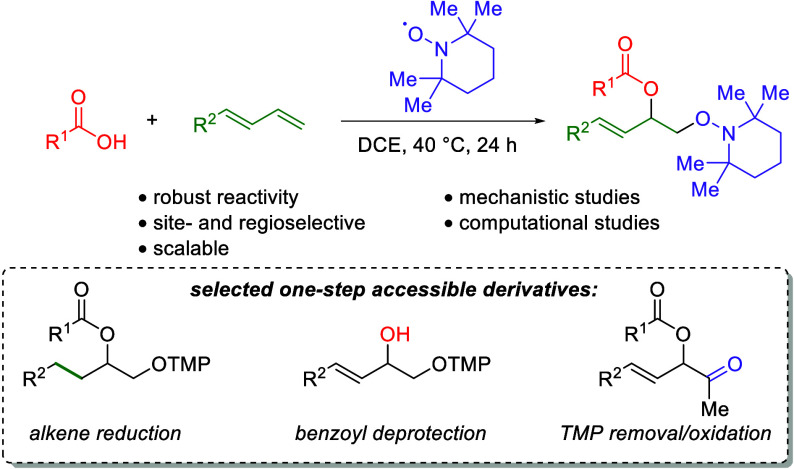

A mild, metal-free 1,2-dioxygenation of 1,3-dienes using TEMPO and carboxylic acids is reported. This method includes examples for a variety of 1,3-dienes as well as aliphatic and aromatic carboxylic acids. This reaction also demonstrates complete site- and regioselectivity in the oxygen addition. Furthermore, extensive derivatization of the differential oxygen groups in the product has been demonstrated, including reduction of the remaining alkene to access alkyl, vicinally dioxygenated scaffolds. Finally, this reaction is shown both experimentally and computationally to occur through carboxylic acid-driven disproportionation of TEMPO.

## Introduction

The installation of oxygen into carbon scaffolds remains at the forefront of synthetic efforts, as 96% of the top 200 small molecule drugs contain an oxygen atom.^1^ Furthermore, 23% of the aforementioned compounds contain a vicinal 1,2-dioxygen skeleton. Accordingly, synthetic methods to access these scaffolds are highly desirable.

**Scheme 1:**
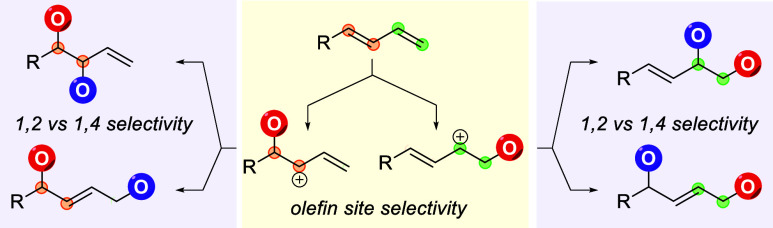
Challenges in Selectivity with the 1,2-Difunctionalization of 1,3-Dienes

The vicinal difunctionalization of olefins provides efficient access to 1,2-dioxygen skeletons and is one of the most foundational reactions in organic chemistry. It offers attractive features such as rapid, single-step assembly of molecular complexity from readily available feedstock chemicals. Accordingly, the 1,2-dioxygenation of alkenes has seen an abundance of method development to enact this transformation through a number of elegant processes.^2^ Despite the high number of methods for the 1,2-dioxygenation of alkenes, the dioxygenation of 1,3-dienes has been significantly less studied. Much of this lack of investigation can be attributed to the inherent two-fold difficulty in the selectivity of unsymmetrical 1,3-dienes (Scheme 1). First, there are two possible olefin addition sites that could react with the initial oxygen source to create issues with olefin site selectivity. Second, there are both 1,2- and 1,4-adducts that can be formed from either olefin site addition through a cationic or radical reaction intermediate. This creates the possibility of four products within a single reaction. Notably, there have been a select number of descriptions of 1,4-dioxygenation of 1,3-dienes, mainly driven by photo-oxidation with singlet oxygen, often through pericyclic reactivity.^3^ Despite these advances in 1,4-dioxygenation, 1,2-dioxygenation remains elusive.

**Scheme 2:**
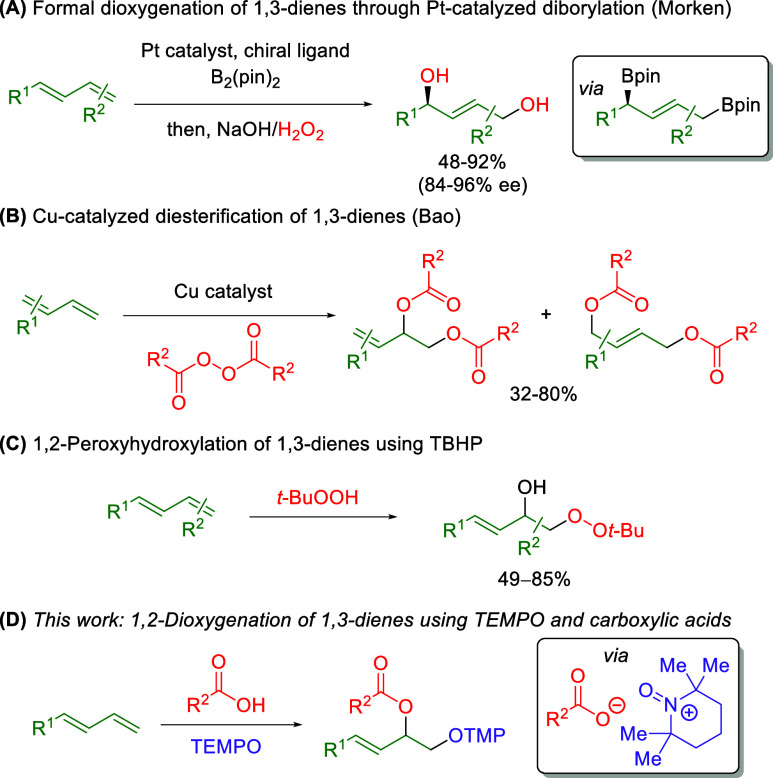
Methods for the 1,2-Dioxygenation of 1,3-Dienes

Morken and co-workers examined the enantioselective 1,4-diborylation of 1,3-dienes with subsequent oxidation to accomplish a formal 1,4-dioxygenation (Scheme 2A).^4^ Although this method allows for selective formation of the 1,4-dioxygenated products, additional work revealed that the 1,2-dioxygenated product can be selectively produced using 1,3-dienes with *Z* geometry or 1,1-disubstitution.^5^ Additionally, Bao and co-workers achieved a diesterification of 1,3-dienes using symmetric benzoyl peroxides in concert with copper catalysts (Scheme 2B).^6^ Although this strategy demonstrated good selectivity on some substrates, others often provided a mixture of 1,2 or 1,4 products or a mixture of olefin site isomers. Recently, two independent reports of 1,2-peroxyhydroxylation of 1,3-dienes using TBHP have been disclosed by Fernandes^7^ and Liu,^8^ providing the first example of differential oxygen sources in the 1,3-diene dioxygenation (Scheme 2C). Finally, there have also been a number of cases in which reports of alkene 1,2-dioxygenation have included a small number of 1,3-diene substrates.^2g,9^ This work entails the use of free radical TEMPO (2,2,6,6-tetramethylpiperidine 1-oxyl) with carboxylic acids for the 1,2-dioxygenation of 1,3-dienes driven by TEMPO disproportionation (Scheme 2D).

## Results and Discussion

Following the serendipitous discovery of this reaction,^10^ optimization was undertaken using pentafluorobenzoic acid (**1a**), (*E*)-1-phenyl-1,3-butadiene (**2a**), and TEMPO (**3a**) as standard substrates (Table 1). Using the same ratio of starting materials and catalyst loading present in the initial discovery, a 27% yield was observed (entry 1). Ultimately, the copper catalyst proved deleterious, as removal of the copper salt provided a similar yield to the 1 mol % case and a significantly higher yield than 50 and 100 mol % loadings (entries 2–4). When the equivalents of the acid, 1,3-diene, and TEMPO were tested, it was clear that excess carboxylic acid was necessary for elevated yields (entries 5–8). Further component screening revealed a slight preference for excess TEMPO over excess diene (entries 9–10). Finally, the temperature and time were examined concurrently, with a final condition of 40 °C for 24 h (entries 11–13), which was adopted as the standard conditions.

**Table 1:**
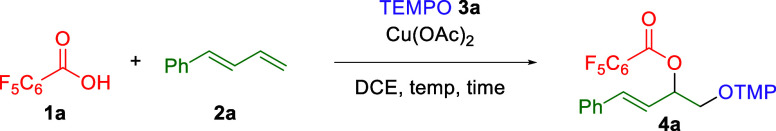
Optimization of Reaction Conditions for the 1,2-Dioxygenation of (*E*)-1-Phenyl-1,3-butadiene (**2a**)^a^

	**1a**	**2a**	**3a**				
entry	(equiv)			Cu(OAc)_2_ (mol %)	temp (°C)	time (h)	**4a** (%)^b^
1	2	3	1	1	60	2	27
2	2	3	1	–	60	2	25
3	2	3	1	50	60	2	21
4	2	3	1	100	60	2	14
5	1	1	1	–	60	2.5	18
6	1	1	3	–	60	2.5	1
7	1	3	1	–	60	2.5	19
8	3	1	1	–	60	2.5	43
9	3	2	1	–	60	2.5	45
10	3	1	2	–	60	2.5	49
11	3	1	2	–	60	24	47
12	3	1	2	–	80	24	34
13	3	1	2	–	40	24	59 (54)^c^

a Reactions on 0.1 mmol scale.

b Reaction yields determined by ^1^H NMR of the crude reaction with dibromomethane as a quantitative internal standard.

c Isolated yield.

Upon determining the optimal conditions for the 1,2-dioxygenation of 1,3-dienes, a survey of TEMPO derivatives was undertaken (Table 2). Moving from the unsubstituted TEMPO scaffold (**4a**), 4-hydroxy and 4-acetamide derivatives exhibit poor yields and complicated reactions (**4b**–**c**), likely due to poor solubility in DCE and the corresponding heterogeneity observed in the reaction. However, the use of a 4-methoxy derivative results in notably higher yield and conversion with scalability (**4d**). The 4-benzoyl derivative provides comparable yield to the standard TEMPO radical (**4e**), while the piperidone derivative produces no detectable product (**4f**).

**Table 2:**
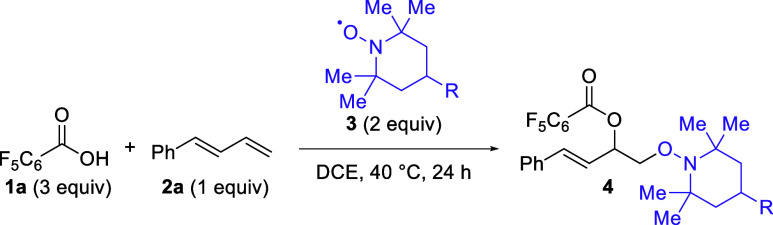

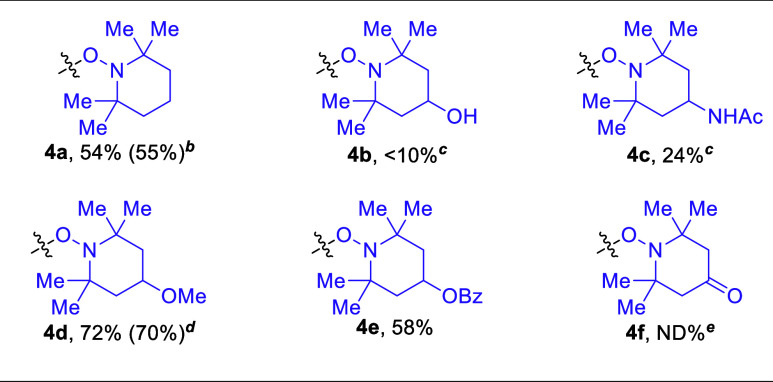
Scope of 2,2,6,6-Tetramethylpiperidine 1-Oxyl Derivatives in the 1,2-Dioxygenation of 1,3-Dienes^a^

a Isolated yields on a 0.1 mmol scale.

b Reaction run on 0.5 mmol scale.

c Reaction yields determined by ^1^H NMR of the crude reaction with dibromomethane as a quantitative internal standard.

d Reaction run on 1.0 mmol scale.

e ND = not detected by ^1^H NMR.

Using the 4-methoxy TEMPO derivative in future reactions, the scope of carboxylic acids was investigated (Table 3). It was found that highly acidic benzoic acid derivatives (p*K*_a_ < 2) exhibit good results (**5a**–**b**), comparable to the initial pentafluoro substrate (**4d**). However, use of less acidic derivatives results in no detectable product (**5c**–**d**). Similarly, use of dichloroacetic acid (1.2 p*K*_a_) produces a high yield (**5e**), while acetic acid (4.7 p*K*_a_) results in no product (**5f**). From these results, it is clear that the reaction yield and efficiency are correlated with the acidity of the corresponding carboxylic acid.

**Table 3:**
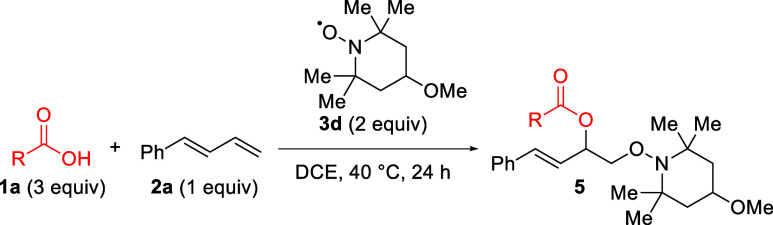

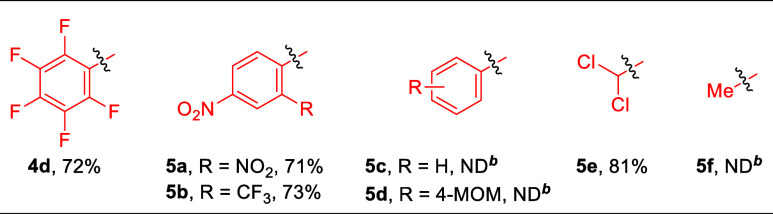
Scope of Carboxylic Acids in the 1,2-Dioxygenation of 1,3-Dienes^a^

a Isolated yields on a 0.1 mmol scale.

b ND = not detected by ^1^H NMR.

The scope of compatible 1,3-dienes was thoroughly examined, beginning with substitutions on the aryl ring of the initial 1-phenyl-1,3-butadiene substrate (Table 4). Although *para*-substitution of electronically neutral groups provides comparable yield (**6a**–**b**), an electron-donating *para*-methoxy group results in notably diminished yields (**6c**). However, a closer inspection determined that this substrate exhibits low stability to purification, as evidenced by a crude quantitative ^1^H NMR yield approaching that seen in other *para*-substituted scaffolds. Furthermore, appending *para*-halogens to the substrate results in comparable yield to unsubstituted cases (**6d**–**e**). Notably, when a significantly electron-withdrawing trifluoromethyl group is added, the yield is reduced (**6f**); furthermore, 1,2:1,4-addition products are observed in a 1.3:1 ratio, marking the only case of a 1,4-product observed within this study. The addition of a *meta*- or *ortho*-methoxy group results in high yields (**6g**–**h**) with minimal observation of product decomposition in the *ortho* case. When a 2-naphthyl system was tested (**6i**), the reaction proceeded smoothly. Attempts to utilize unactivated, alkyl 1,3-dienes, such as (*E*)-1-nonyl-1,3-butadiene resulted in no detectable product (**6j**). Following this exploration of monosubstituted systems, multisubstituted 1,3-dienes were examined. The addition of a methyl group to the terminal end (4-position) of the model scaffold results in a reduced yield (**6k**), while addition of two methyl groups fully obstructs the reactivity (**6l**). This result suggests a predictable susceptibility of this reaction to steric hindrance at the site of the TEMPO addition. Addition of methyl groups to the 3- or 2-position (**6m**, **6n**) results in minimal product formation for reasons that are currently not well understood. 1,1-Diaryl-substituted systems, as well as a fluorene scaffold, display high yields (**6o**–**q**), suggesting that steric hindrance is not a considerable factor in the reaction when isolated to one end of the 1,3-diene system. Additionally, simple alkyl dienes, such as isoprene and 2,3-dimethyl-1,3-butadiene, do not produce the desired product (**6r**–**s**). However, use of an electron-rich alkene (*trans*-anethole) results in fair yield (**6t**), providing analogous reactivity to previous efforts in this field.^2g,11^ Use of *para* and *ortho* methoxystyrene (**2u**–**v**) also results in yields that are comparable to *trans*-anethole, but electronically neutral styrene failed to produce any product (**6w**).

**Table 4:**
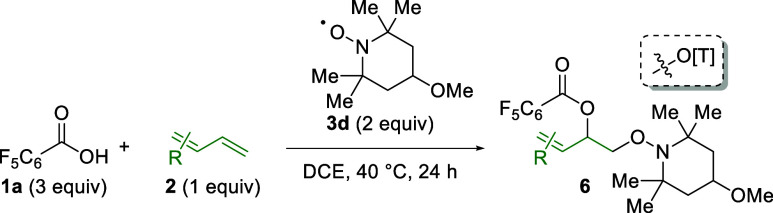

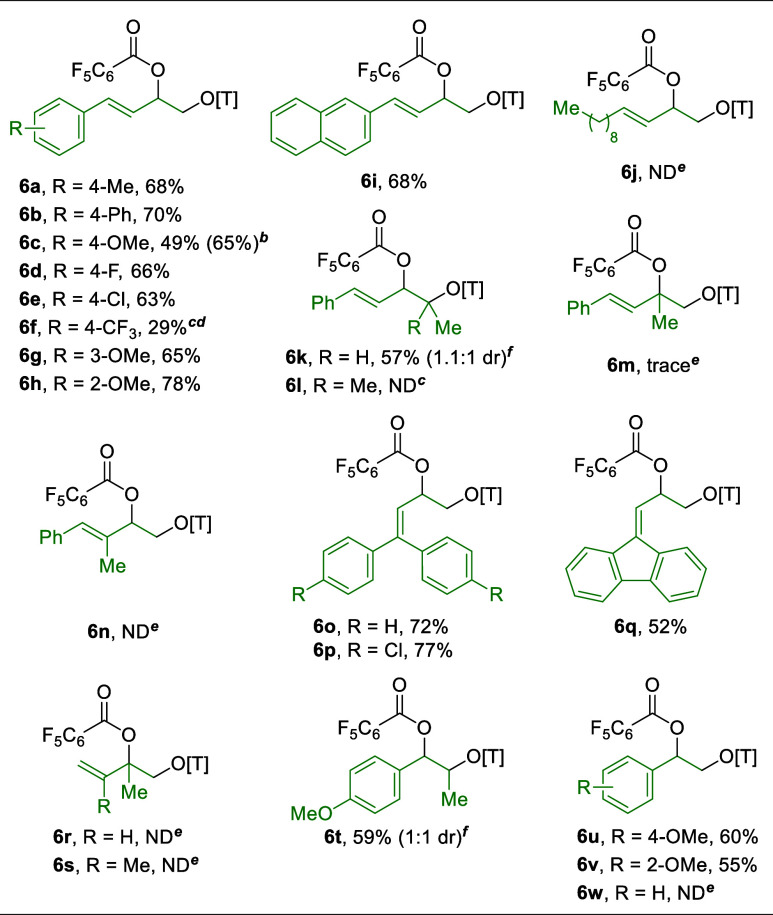
Scope of Dienes in the 1,2-Dioxygenation of 1,3-Dienes^a^

a Isolated yields on a 0.1 mmol scale.

b Reaction yield in parentheses determined by ^1^H NMR of the crude reaction with dibromomethane as a quantitative internal standard.

c Ratio of 1.3:1 for the 1,2:1,4-product by crude ^19^F NMR.

d Ratio of 2.2:1 for the 1,2:1,4-product by ^19^F NMR after chromatographic purification.

e ND = not detected by ^1^H NMR.

f dr = diastereomeric ratio, determined by ^1^H NMR of the crude reaction.

**Scheme 3:**
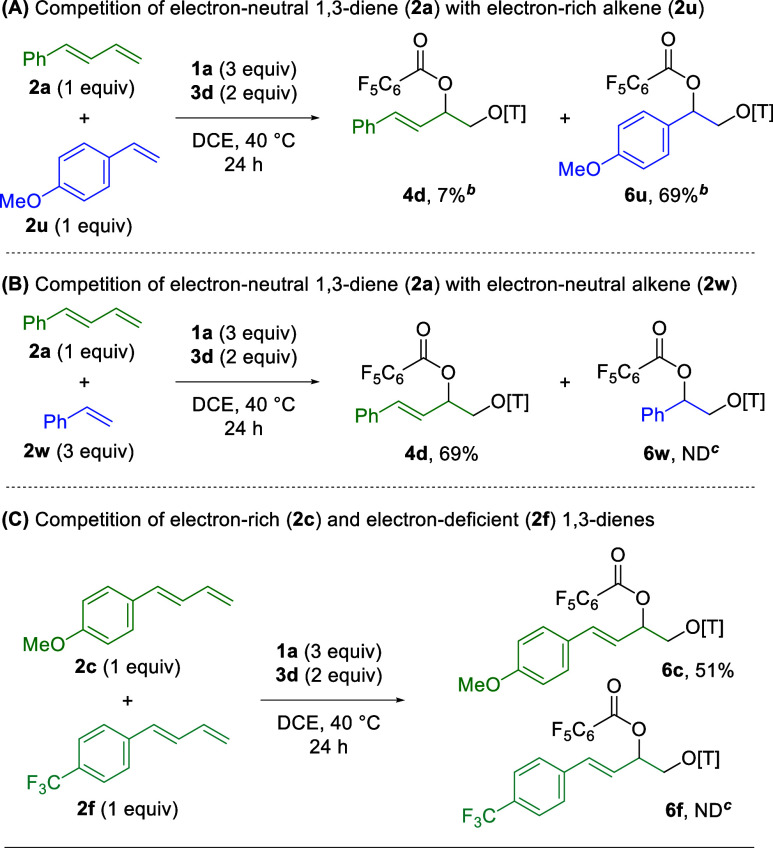
Competition of 1,3-Dienes and Alkenes^a^ Isolated yields on a 0.1 mmol scale. Isolated as a 1:9.7 mixture. ND = not detected by ^1^H NMR. [T] = 2,2,6,6-tetramethylpiperidine.

To further understand the reactivity of 1,3-dienes vs alkenes, competition experiments were conducted. When electron-neutral 1,3-diene **2a** is combined with electron-rich alkene **2u**, 1,2-dioxygenation of the alkene (**6u**) is observed preferentially over 1,2-dioxygenation of the 1,3-diene (**4d**) in a 9.7:1 ratio (76% combined yield, Scheme 3A). However, when electron-neutral 1,3-diene **2a** is combined with an excess of electron-neutral alkene **2w**, only the 1,3-diene adduct (**4d**) is observed in 69% yield (Scheme 3B). From this, it is clear that electron-rich alkenes provide superior reactivity in this system compared to electron-neutral 1,3-dienes, but electron-neutral 1,3-dienes are more effective than electron-neutral alkenes. Additionally, it is clear that the presence of the unreactive alkene (**2w**) is not deleterious in the reaction with the 1,3-diene (**2a**). Finally, when electron-rich 1,3-diene **2c** is combined with electron-deficient 1,3-diene **2f**, only the product of the electron-rich 1,3-diene is observed (**6c**) in comparable yield to the original system without the electron-deficient competitor (Table 4). This further demonstrates that electron-rich π-systems are superior to their electron-deficient counterparts in this reaction platform.

**Scheme 4:**
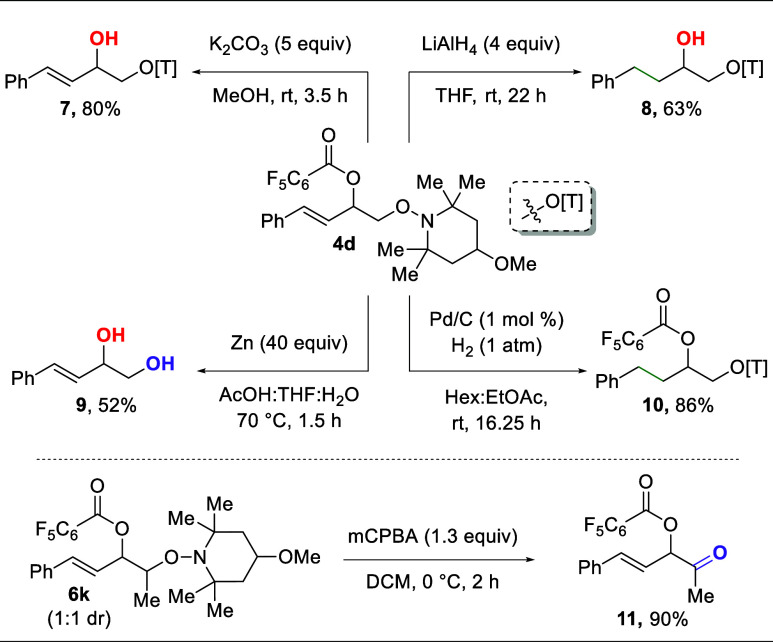
Derivatization of the Product Scaffold Isolated yields on a 0.05 mmol scale.

This vicinally diooxygenated scaffold (**4d**) can be further manipulated to afford differential oxygen sources (Scheme 4). First, the benzoyl group is easily cleaved through mild hydrolysis conditions to yield the allylic alcohol (**7**) in good yield, leaving the TMP group intact. Furthermore, the benzoyl group can be cleaved with simultaneous reduction of the alkene using LiAlH_4_ to afford alkyl scaffold **8**. Both the benzoyl group and the TMP group can be cleaved simultaneously through an aqueous zinc reduction at elevated temperatures to afford 1,2-diol **9**. Notably, attempts to remove the TMP group while preserving the benzoyl group were unsuccessful (see Supporting Information, Section 6). Additionally, the alkene of **4d** can be reduced with Pd/C to afford orthogonally protected **10**. The flexibility of these derivatizations to provide access to alkyl scaffolds through alkene reduction is quite important, as many alkene 1,2-dioxygenation methods struggle to provide good reactivity for unactivated (alkyl), terminal alkenes. This two-step sequence provides a rapid entry to these scaffolds. Finally, the TMP group on dioxygenation product **6j** can be easily oxidized to the corresponding ketone (**11**) without undesired reactivity on the alkene or ester functionality.

## Mechanistic Investigation

**Scheme 5:**
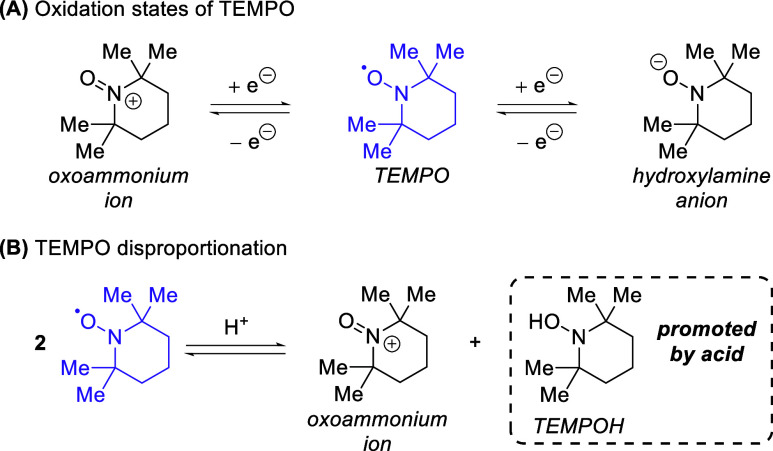
TEMPO Oxidation

TEMPO has long been studied for its oxidation properties due to its ability to move between three oxidation states as its oxidized form (oxoammonium ion) and reduced form (hydroxylamine anion) (Scheme 5a). This ability to move readily between oxidation states has been leveraged in a plethora of applications using both stoichiometric and catalytic TEMPO forms.^12^ Furthermore, TEMPO disproportionation involves the simultaneous oxidation and reduction of two TEMPO species to provide both an oxoammonium ion and a hydroxylamine anion (Scheme 5b). It has been noted that this equilibrium can be favored in acidic environments and the equilibrium fully shifted to the disproportionated form using strong acids (pH below 2).^13^

**Scheme 6:**
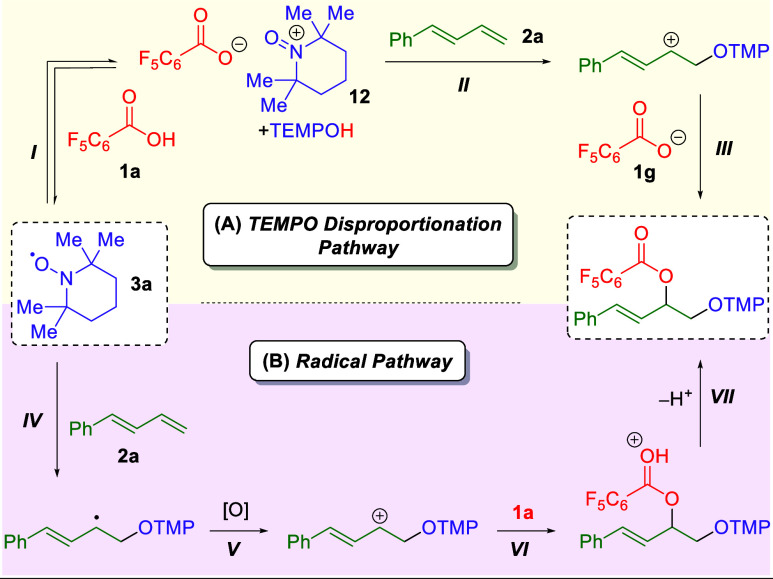
Mechanism for the 1,2-Dioxygenation of 1,3-Dienes through TEMPO Disproportionation (A) or Radical (B) Pathways

For the 1,2-dioxygenation of 1,3-dienes, two mechanistic possibilities were considered (Scheme 6), one relying on TEMPO disproportionation and the second relying on TEMPO radical addition. First, it was proposed that the carboxylic acid (**1a**) could promote TEMPO disproportionation (**I**) to produce the carboxylate, the TEMPO oxoammonium salt (**12**), and TEMPOH. This oxoammonium salt would then be attacked by the 1,3-diene (**2a**) to generate a carbocation intermediate (**II**), which could be attacked by the carboxylate to yield the final product (**III**). Alternatively, it was considered that TEMPO could add directly to the 1,3-diene (**2a**) as a radical (**IV**). This carbon radical could then be oxidized by exogenous oxygen (**V**) to afford a carbocation that could be attacked by the carboxylic acid (**1a**) to yield an oxonium ion (**VI**). Deprotonation of this intermediate would afford the final product (**VII**).

Notably, the oxoammonium salt of TEMPO has been previously demonstrated as an electrophilic oxygen source for use in alkene functionalization, either through direct use of the oxoammonium salt^11^ or proposed through disproportionation from the oxyl radical.^2g^ However, these methods all require electron-rich vinyl anisole derivatives to promote this addition. Reports of Ene-like reactions with the TEMPO oxoammonium ion have also been reported.^14^

**Scheme 7:**
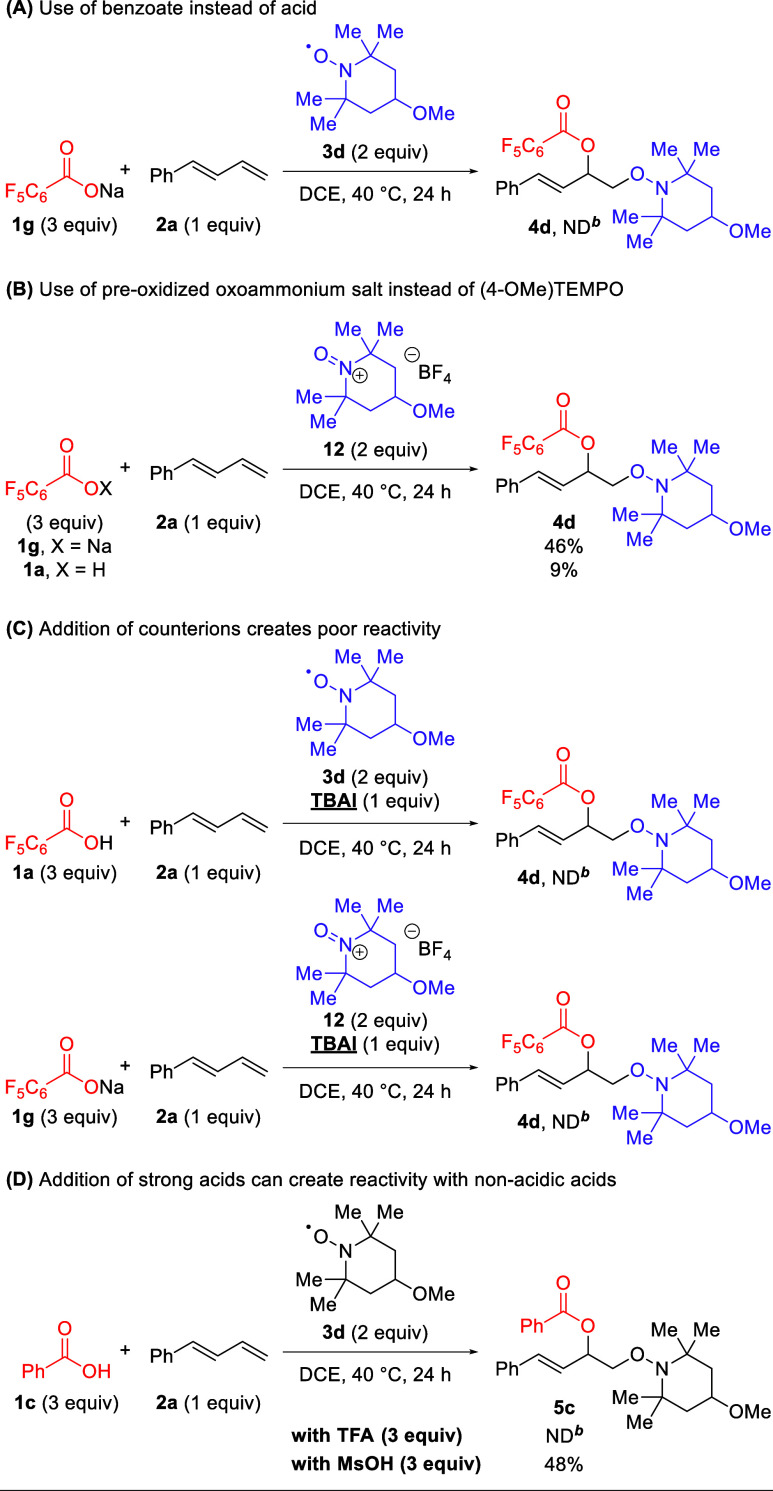
Insights into the Mechanism of the 1,2-Dioxygenation of 1,3-Dienes ND = not detected by ^1^H NMR. Isolated yields on a 0.1 mmol scale.

Because the exact mechanism of TEMPO addition to olefins had not been directly investigated, a number of mechanistic experiments were conducted (Scheme 7). Radical scavengers such as BHT and DPE were added to the reaction but failed to produce insightful adducts or the 1,2-dioxygenation product (see Supporting Information, Section 7). There are two conceivable means of TEMPO addition to 1,3-dienes: either through polar means using TEMPO disproportionation to create an oxoammonium cation or through direct addition of the TEMPO oxygen radical. To probe for the possible radical addition, sodium pentafluorobenzoate (**1g**) was added to the standard reaction conditions (A) in place of pentafluorobenzoic acid. No product was produced, demonstrating that the addition of the TEMPO radical to the 1,3-diene is unlikely to be the operative pathway. Furthermore, when the oxoammonium salt of TEMPO (**12**) was used as the TEMPO source with the benzoate salt (**1g**), the reaction occurred in 46% yield (B). This demonstrated the operative nature of a cationic addition to the 1,3-diene with nucleophilic trapping from the benzoate. Notably, when pentafluorobenzoic acid (**1a**) is tested with the oxoammonium salt (**12**), minimal product is created. This prompted the testing of exogenous counterion additives in solution for potential interference with the reaction (C). When TBAI is added to either of the aforementioned successful reactions (A/B), no product is recovered, leading to a hypothesis that the ion pairing of the benzoate and oxoammonium salts is likely important to the viability of this reaction. Finally, it can be hypothesized that the poor reactivity of higher p*K*_a_ carboxylic acids (Table 3), such as benzoic acid, is due to an inability to promote TEMPO disproportionation. This is further supported by ultraviolet–visible (UV–vis) studies that suggest pentafluorobenzoic acid (**1a**) as being deprotonated in the presence of TEMPO, while benzoic acid (**1c**) remains protonated in the presence of TEMPO (see Supporting Information, Section 8). Accordingly, it was hypothesized that the use of exogenous acids might provide the necessary acidity to promote TEMPO disproportionation and reactivity with benzoic acid. To test this, additives of trifluoroacetic acid (TFA) or methanesulfonic acid (MsOH) were added to the standard reaction with benzoic acid (**1c**). In the case of TFA, no reaction was observed, but the presence of MsOH provides a moderate yield of the desired 1,2-dioxygenation adduct, offering promise for the use of this system with a wider range of nucleophiles.

## DFT Calculations

Two primary mechanisms for the 1,2-dioxygenation were considered, and corresponding DFT calculations were used to support experimental findings. Geometry optimizations and single point energy calculations were conducted with Gaussian 16^15^ and prepared with GaussView 6^16^ for relevant reactants and intermediates in a series of reactions. The DFT calculations were carried out at the wB97xD^17^/Def2-TZVP^18^ level of theory, with solvent effects considered with the SMD^19^ model with 1,2-dichloroethane as the solvent to match experimental conditions. Coordinates, structures, and thermodynamic properties for each compound are provided in the Supporting Information. Two mechanisms were considered in this study: a mechanism initialized by the disproportionation of TEMPO and a mechanism that begins with the radical addition of TEMPO to the diene. The first mechanism considered here is the disproportionation due to the support of this mechanism in the experimental results.

**Scheme 8:**
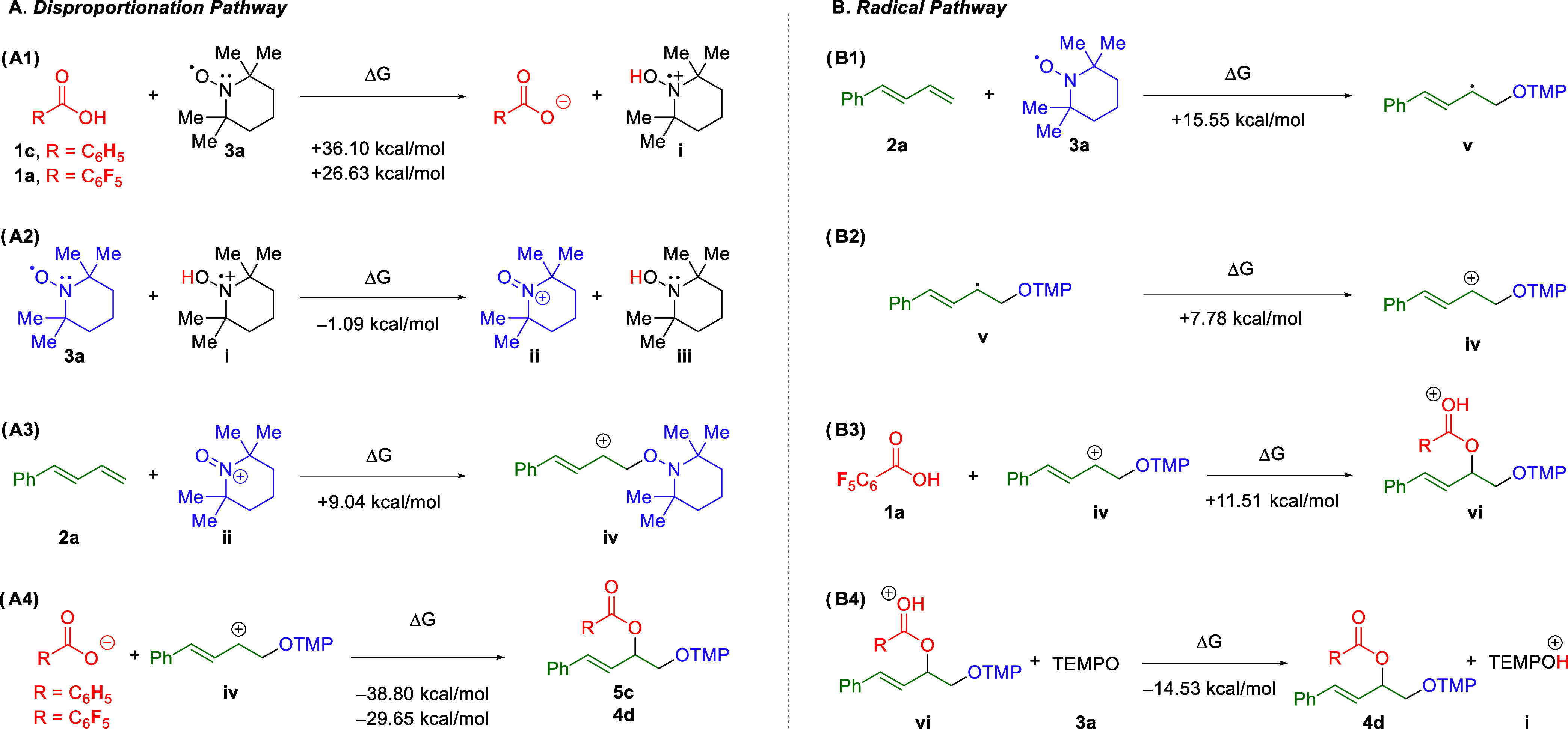
Reactions and Calculated Δ*G* Values in DCE for the Disproportionation (A) and Radical (B) Mechanisms

Mechanisms for the disproportionation of TEMPO have been studied extensively in aqueous solution. Specifically, strong acids^13b,13c,20^ and Lewis acids^21^ have been shown to be effective catalysts for disproportionation. This work extended the theory of this reaction to carboxylic acids acting in organic solvents. Scheme 8A shows several reactions of interest, along with the calculated changes in Gibbs energy for each reaction. The initiating step for the mechanism involves the transfer of a proton from the acid to TEMPO (**3a**) to form the radical cation TEMPOH^+^ (i). The energies for this step are high, but this is again likely due to the low rate of deprotonation observed in 1,2-dichloroethane.^22^ The reaction energy for pentafluorobenzoic acid is notably smaller (∼10 kcal/mol) than that of benzoic acid, owing to its lower p*K*_*a*_. This result matches the experimental favorability of the reaction with pentafluorobenzoic acid over unreactive benzoic acid. Following proton transfer, disproportionation of TEMPOH^+^ (i) is favored, forming the oxoammonium cation (TEMPO^+^) (ii) and the hydroxylamine (TEMPOH) (iii). The cation is then attacked by the diene to create the carbocation intermediate (iv). The final step in this scheme is the reaction between the deprotonated acid and the carbocation intermediate (iv). For each acid examined, this final step is exergonic, though the calculated Δ*G* for the reaction involving benzoic acid is ∼9 kcal/mol lower than pentafluorobenzoic acid. This disparity was examined by considering the calculated Hirschfeld charges^23^ for each anion species. The oxygen atoms in the deprotonated carboxyl group for benzoic acid have a calculated Hirschfeld charge of −0.46, while the presence of the electron-withdrawing fluorines in pentafluorobenzoic acid resulted in a calculated Hirschfeld charge of −0.44.

A second mechanism was examined due to the relatively high calculated energies in the disproportionation mechanism. The relevant reactions and calculated Δ*G* values are shown in Scheme 8B. This reaction was studied computationally only with pentafluorobenzoic acid due to its demonstrated experimental efficacy. The mechanism begins with the straightforward addition of the radical TEMPO (**3a**) to the diene, forming a radical intermediate (v). The radical intermediate is then oxidized by solvated oxygen to form a carbocation intermediate (iv). The neutral carboxylic acid is then added nucleophilically, followed by the transfer of a proton to an additional TEMPO to form the product (**4d**). The first three steps of this mechanism are endergonic; nevertheless, the relative energies for each step are within reasonable limits for a slow reaction. However, the final step, while exergonic, shows a release of energy that is insufficient for this radical path to be energetically favorable in comparison to the disproportionation mechanism proposed (Scheme 8A). With the values presented here, the disproportionation path is likely the mechanism for the reaction.

**Figure 1:**
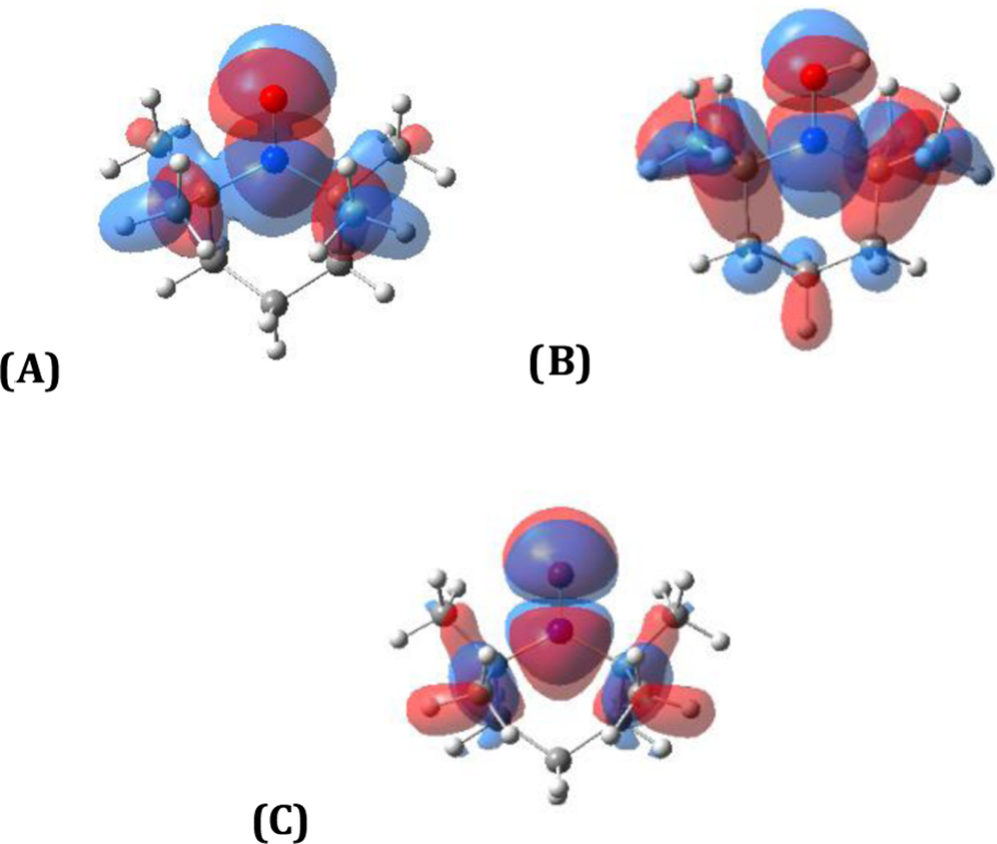
Molecular orbitals relevant to the proposed mechanism. (A) SOMO for TEMPO (**3a**), (B) SOMO for TEMPOH^+^ radical (i), and (C) LUMO for TEMPO^+^ (ii).

Molecular orbitals relevant to the disproportionation mechanism are shown in Figure 1 as a partial explanation for the disproportionation of TEMPO by carboxylic acids in 1,2-dichloroethane. The neutral radical has a singly occupied molecular orbital (SOMO) centered on the aminoxyl functional group (Figure 1a), allowing for the transfer of electrons through disproportionation. However, this process is pH-dependent and unlikely to occur without a catalyst in organic solvents. The mechanism proposed first depends on the transfer of a proton from the carboxylic acid, a transfer that is likely due to the location of the SOMO on the radical. The location of the SOMO does not significantly change after proton transfer, as shown in Figure 1b, indicating that the first step in the mechanism does not prevent disproportionation with a second TEMPO molecule. Following the disproportionation, we consider the LUMO of the oxoammonium cation (ii) in Figure 1c and conclude that the location of the molecular orbital is well positioned for the cation to act as an electrophile in the reaction with the diene.

In conclusion, a 1,2-dioxygenation reaction has been established for 1,3-dienes using TEMPO and carboxylic acids. This reaction exhibits good yields with a range of substrates and provides site- and regioselective reactivity for 1,3-dienes. Computational studies provide backing for experimental results that the reaction proceeds through acid-promoted TEMPO disproportionation, with the resulting TEMPO oxoammonium ion adding to the 1,3-diene (Scheme 6A). Future studies will seek to expand the range of accessible nucleophiles using acid additives and apply this TEMPO disproportionation platform to other reaction systems.

## Data Availability

The data underlying this study are available in the published article and its Supporting Information.

## Supporting Information Available

Experimental procedures and full characterization data for all new compounds with copies of ^1^H, ^13^C, and ^19^F spectra, UV–vis studies, and computational details (PDF)
