# Supplementary material for: Three-Component 1,2-Dioxygenation of 1,3-Dienes Using Carboxylic Acids and TEMPO
Source: J Org Chem. 2024 Nov 4;89(22):16865–72. doi: 10.1021/acs.joc.4c02244 (PMC11574855; doi:10.1021/acs.joc.4c02244)
Supplement: Supplementary file 1 — jo4c02244_si_001.pdf [file jo4c02244_si_001.pdf]

Three-Component 1,2-Dioxygenation of 1,3-Dienes Using Carboxylic Acids and TEMPO

Sophia M. Baldassarre, Heidi S. Sato, Adam P. Louise, Layna Summer, Benjamin P. Wilson,* and Brett N. Hemric*

Department of Chemistry and Biochemistry, University of Tampa, Tampa, FL 33606

Email: bhemric@ut.edu

SUPPORTING INFORMATION

Table of Contents **Page**

1. General methods S1
2. Condition Optimization for the 1,3-Diene 1,2-Dioxygenation Reaction S2
3. Synthesis of Starting Materials S5
4. Diene 1,2-Dioxygenation Protocols and Characterization of Novel Compounds S10
5. Competition Experiments S21
6. Derivatization Reactions S22
7. Controls and Mechanistic Experiments S25
8. UV-Vis Studies S27
9. Computational (DFT) Details S29
10. References S38
11. ^{1}H, ^{13}C, and ^{19}F NMR Spectra S39

Experimental Section

1. General Methods

General Procedures

Stir bars were cleaned stirring in concentrated nitric acid overnight, rinsed with water and acetone, dried in an oven at 120 °C overnight and cooled/stored in a desiccator filled with Drierite. Optimization and substrate screens were performed in 1 Dram glass vials with Teflon-coated micro stir bar. All other reactions were performed in round-bottom flasks with rubber septa and Teflon-coated stir bars, unless otherwise noted. Plastic syringes were used for the transfer of pure solvents, while glass pipets were used for transfer of crude reaction solutions. Analytical thin-layer chromatography (TLC) was performed using aluminum plates coated with a 0.25 mm layer of 230–400 mesh silica gel with fluorescent indicator (254 nm). TLC plates were visualized by exposure to ultraviolet light and treatment with vanillin stain. Organic solutions were concentrated under reduced pressure using a rotary evaporator and flash chromatography performed using 60 Å silica gel.

Materials

Commercial reagents and anhydrous solvents were used as received.

Instrumentation

Nuclear magnetic resonance spectra were recorded on a JEOL 400 MHz cryoprobe at room temperature unless otherwise noted. Chemical shifts for ^{1}H NMR are reported in parts per million (ppm, δ) and

referenced to residual protium in $CDCl_3$ (δ 7.26). Chemical shifts for ^{13}C NMR are reported as total carbons in parts per million (ppm, δ) and referenced to the carbon resonances of $CDCl_3$ (δ 77.0) unless otherwise noted. Chemical shifts for ^{19}F NMR are reported in parts per million (ppm, δ) and referenced to the fluorine resonance of $PhCF_3$ (δ –63.3) unless otherwise noted. NMR values are reported as follows: chemical shift, multiplicity (s = singlet, d = doublet, t = triplet, q = quartet, p = pentet, m = multiplet, br = broad), coupling constant (Hz), and integration. Infrared spectroscopic data was obtained on a Thermo Nicolet iS10 FTIR and is reported in wavenumbers (cm–1). High-resolution mass spectra of compounds were obtained using a Thermo Scientific Orbitrap Exploris 120 electrospray ionization mass spectrometer, introduced through direct injection (5 µL/min flow rate, 80 psi nitrogen gas curtain). UV-Vis spectra were obtained on a Agilent Cary 300 double-beam UV-Vis spectrophotometer using semi-microscale quartz cuvettes.

2. Condition Optimization for the 1,2-Dioxygenation of 1,3-Dienes.

General Example of Optimization Screening Conditions

To a 1-dram vial equipped with a Teflon-coated stir bar was added the 1,3-diene **2** (0.1 mmol, 1.0 equiv). 1,2-Dichloroethane (0.33 mL) was added, followed by sequential addition of the acid **1** (0.3 mmol, 3.0 equiv) and oxyl radical **3** (0.2 mmol, 2.0 equiv). The vial was capped and stirred at 40 °C in an aluminum heating block. for 24 h The resulting crude mixture was filtered through activated, neutral Al_2O_3 (Brockman Grade I, 58–60 Å mesh powder) and concentrated *in vacuo* to yield the crude product. Yields were determined by quantitative ^1H NMR spectroscopy through addition of $CDCl_3$ (0.5 mL) and dibromomethane (7.0 µL, 0.1 mmol via a 10 µL microsyringe) to the crude reaction. The resulting solution was analyzed by ^1H NMR with a 45° pulse angle and 4 second relaxation delay with 16 scans. The resulting spectra were analyzed in MestReNova, with the dibromomethane singlet peak (found at 4.905 ppm) set to a relative integration of 2.0.

Table S1. *O*-Benzoylhydroxylamine Survey[a]

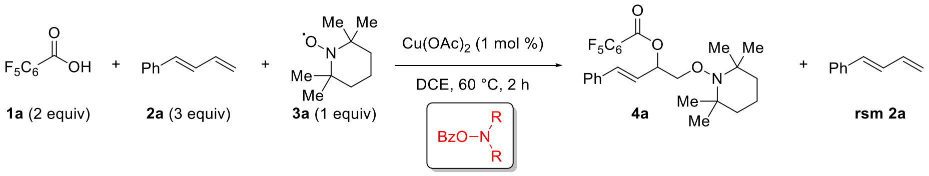

entry	*O*-benzoylhydroxylamine	(equiv)	**4a** (%)[b]	rsm **2a** (equiv)[b]
1	Morpholine	1	16	1.5
2	Morpholine	1	10	1.4
3	NBn_2	1	1	1.3
4	NBn_2	3	0	2.2
5	–	–	27	1.9

[a]Run on 0.1 mmol scale. [b]Determined with quantitative ^1H NMR with dibromomethane (7.0 µL, 0.1 mmol).

Table S2. Copper Catalyst Survey[a]

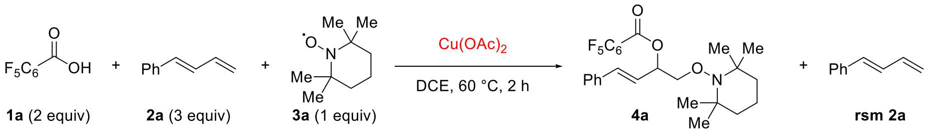

entry	Cu(OAc)2 (mol %)	**4a** (%)[b]	rsm **2a** (equiv)[b]
1	0	25	2.0
2	1	27	2.2
3	20	28	2.2
4	50	21	2.2
5	100	14	2.2

[a]Run on 0.1 mmol scale. [b]Determined with quantitative ^{1}H NMR with dibromomethane (7.0 µL, 0.1 mmol).

Table S3. Component Equivalents Survey[a]

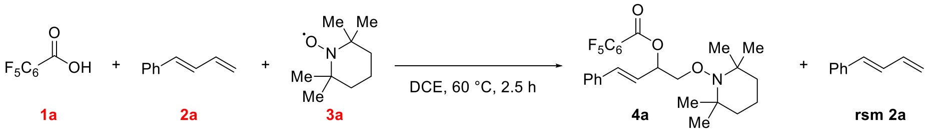

entry	**1a** (equiv)	**2a** (equiv)	**3a** (equiv)	**4a** (%)[b]	rsm **2a** (equiv)[b]
1	1	1	1	18	0.6
2	1	3	1	19	2.5
3	1	1	3	1	0.7
4	2	1	1	33	0.6
5	3	1	1	43	0.5
6	2	2	1	38	1.5
7	2	1	2	33	0.5
8	3	2	1	35	1.0
9	3	1	1.25	38	0.4
10	3	1	1.5	47	0.4
11	3	1	1.75	48	0.4
12	3	1	2	50	0.4
13	3	1	2.5	50	0.4
14	3	1	3	46	0.3

[a]Run on 0.1 mmol scale. [b]Determined with quantitative ^{1}H NMR with dibromomethane (7.0 µL, 0.1 mmol).

Table S4. Temperature and Time Survey[a]

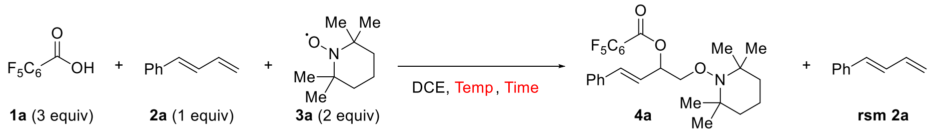

1a (3 equiv) **2a** (1 equiv) **3a** (2 equiv) **4a** **rsm 2a**

entry	temp (°C)	time (h)	**4a** (%)[b]	rsm **2a** (equiv)[b]
1	0	2.5	7	0.5
2	20	2.5	18	0.4
3	40	2.5	29	0.3
4	80	2.5	36	0.4
5	100	2.5	36	0.4
6	20	24	35	0.3
7	40	24	59	0.2
8	60	24	47	0.2
9	80	24	34	0.3
10	40	24	54	0.2
11	40	48	60	0.2
12	40	72	60	0.2

[a]Run on 0.1 mmol scale. [b]Determined with quantitative ^{1}H NMR with dibromomethane (7.0 μL, 0.1 mmol).

Table S5. Solvent Survey[a]

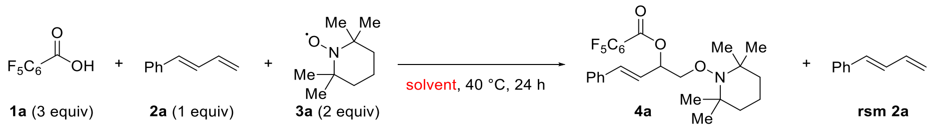

1a (3 equiv) **2a** (1 equiv) **3a** (2 equiv) **4a** **rsm 2a**

entry	solvent	**4a** (%)[b]	rsm **2a** (equiv)[b]
1	DCE	55	0.3
2	DCM	61	0.1
3	$PhCF_3$	46	0.3
4	PhMe	45	0.3
5	THF	3	0.7
6	MeCN	42	0.4
7	acetone	0	0.7

[a]Run on 0.1 mmol scale. [b]Determined with quantitative ^{1}H NMR with dibromomethane (7.0 μL, 0.1 mmol).

Table S6. Concentration Survey[a]

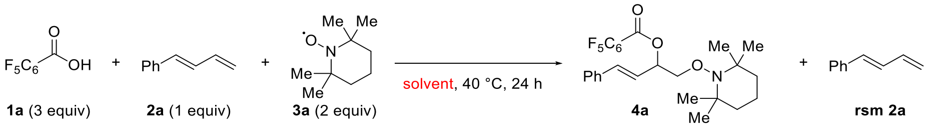

entry	solvent	conc (M)	**4a** (%)[b]	rsm **2a** (equiv)[b]
1	DCE	0.05	43	0.3
2	DCE	0.1	43	0.2
3	DCE	0.2	48	0.2
4	DCE	0.3	51	0.2
5	DCE	0.5	52	0.1
6	DCM	0.05	42	0.3
7	DCM	0.1	44	0.1
8	DCM	0.2	52	0.1
9	DCM	0.3	41	0.1
10	DCM	0.5	51	0.1

[a]Run on 0.1 mmol scale. [b]Determined with quantitative ^{1}H NMR with dibromomethane (7.0 µL, 0.1 mmol). Note that 0.5 M proved impractical on a 0.1 mmol scale, particularly with 24 h heating.

3. Synthesis of Starting Materials

3.1 TEMPO Derivatives

All TEMPO derivatives (**3a–f**) were purchased from commercial suppliers and used as received.

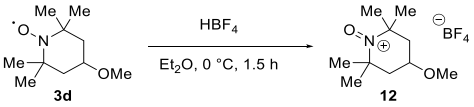

4-Methoxy-2,2,6,6-tetramethyl-1-oxopiperidin-1-ium tetrafluoroborate (12). To a stirring solution of 4-methoxyTEMPO **3d** (931.4 mg, 5.0 mmol, 1.0 equiv) in Et_2O (15 mL) at 0 °C was added tetrafluoroboric acid (48 wt%, 1.4 mL, 11.0 mmol, 2.2 equiv) dropwise over 10 min. The reaction was stirred at 0 °C for 10 min, then allowed to warm to room temperature. After 2.75 h, the reaction was filtered through a Buchner funnel, rinsing with Et_2O (10 mL). The precipitate was dried under vacuum to afford **12** as a yellow solid (550.6 mg, 40%). This compound was consistent with previous report.[1]

3.2 Carboxylic acids

Acids **1a, 1A, 1b, 1c, 1e, 1f** were purchased from commercial suppliers and used as received.

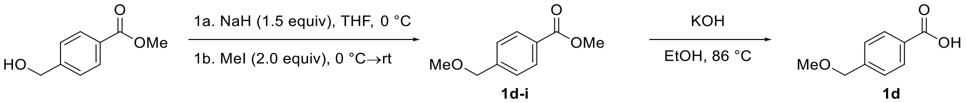

4-(Methoxymethyl)benzoic acid (1d). To a stirred solution of methyl 4-(hydroxymethyl)benzoate (3.32 g, 20.0 mmol, 1.0 equiv) in THF (60 mL) at 0 °C was added NaH (0.72 g, 30.0 mmol, 1.5 equiv). The solution was stirred at 0 °C for 30 min. Methyl iodide (2.50 mL, 40.0 mmol, 2.0 equiv) was added. The reaction was stirred at 0 °C for 10 min, then allowed to warm to room temperature. After 2 h, the reaction was quenched with a saturated aqueous solution of NH_4Cl (20 mL) and DI H_2O (20 mL). The layers were separated and the aqueous solution was extracted with EtOAc (30 mL x 2). The combined organic layers were washed with brine (50 mL), dried with Na_2SO_4, and concentrated *in vacuo* to afford **1d-i** as a clear oil (2.90 g) that was used without further purification.

To a stirred solution of **1d-i** (2.90 g, 16.1 mmol, 1.0 equiv) in EtOH (95% aq, 37 mL) was added a solution of KOH (1.3 M in 95% aq EtOH, 44 mL). The reaction was heated at 86 °C in an aluminum heating block for 1 h, upon which TLC indicated full consumption of **1d-i**. The crude reaction was evaporated *in vacuo* to remove 75% of the EtOH. The resulting solution was quenched with a saturated aqueous solution of NH_4Cl (50 mL) an HCl (3 M, 20 mL). The layers were separated and the aqueous layer was extracted with EtOAc (70 mL x 2). The combined organic layers were washed with brine (50 mL), dried with Na_2SO_4, and concentrated *in vacuo* to afford the **1d** as a white solid that did not necessitate further purification (2.00 g, 60% over 2 steps). Spectra matched previous reports.[2]

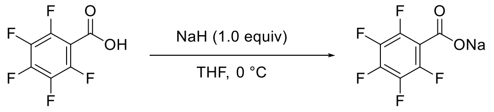

1a (1.0 equiv) **1g**, quantitative

Sodium pentafluorobenzoate (1g). To a stirring solution of anhydrous sodium hydride (120.0 mg, 5.0 mmol, 1.0 equiv) in THF (20 mL) at 0 °C slowly was added pentafluorobenzoic acid (1.060 g, 5.0 mmol, 1.0 equiv). Rapid gas evolution was observed and a white precipitate was formed. After 5 min, the reaction was allowed to stir at room temperature for 2 h. The suspension was concentrated *in vacuo* to afford **1g** as a white solid (1.171 g, 100%). **$^{13}C\{^1H\}$ NMR** (D_2O, 100 MHz, MeOH as internal standard: 49.5 ppm): δ 166.1, 143.5 (d, $J_{C–F}$ = 245.6 Hz, 2C), 141.6 (d, $J_{C–F}$ = 250.9 Hz, 1C), 138.0 (d, $J_{C–F}$ = 251.3 Hz, 2C), 115.0 (t, $J_{C–F}$ = 21.2 Hz, 1C); **^{19}F NMR** (D_2O, 376.5 MHz, $HOCH_2CF_3$ as internal standard: –76.5 ppm): δ –143.8 (dd, *J* = 23.7, 8.4 Hz, 2F), –155.3 (t, *J* = 20.6 Hz, 1F), –161.6 (m, 2F); **FTIR** (solid): cm^{-1} 1651, 1603, 1529, 1475, 1381, 1300, 1116, 985, 826, 759; **HRMS** (ESI^-, m/z): Calcd for $C_6F_5^-$ ([M–$NaCO_2$]): 166.9926; found: 166.9924.

3.3 1,3-Dienes

Olefins (**2r–v**) were purchased from commercial suppliers and used as received.

Standard Conditions for the Conversion of Aldehydes or Cinnamaldehydes to 1,3-Dienes

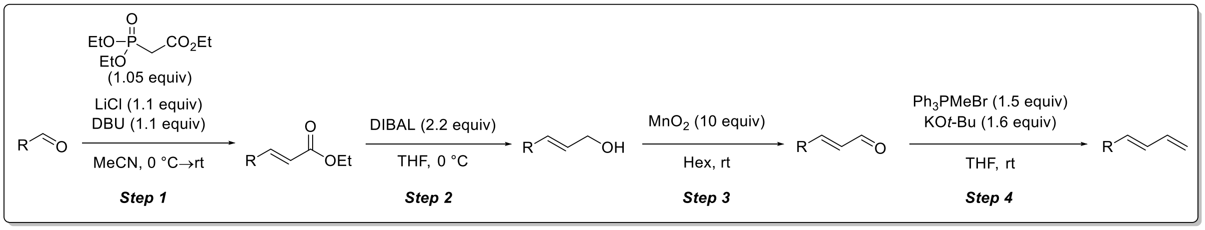

<u>**Step 1 (aldehyde to cinnamyl ester)**</u>: Modified from previous report.[3] To a stirring solution of lithium chloride (1.1 equiv) in MeCN (0.33 M) at 0 °C (ice bath) was added triethyl phosphonoacetate (1.05 equiv). After 15 min, the aldehyde (1.0 equiv) and 1,8-Diazabicyclo[5.4.0]undec-7-ene (DBU, 1.1 equiv) was added.

The reaction was removed from the ice bath and stirred at room temperature overnight. The volatiles were removed *in vacuo*. The crude reaction was diluted with Et_2O (2 mL/mmol) and DI H_2O (2 mL/mmol). The layers were separated and the aqueous layer extracted with Et_2O (2mL/mmol x 3). The combined organic layers were washed with brine (2 mL/mmol x 2), dried with Na_2SO_4, and concentrated *in vacuo* to yield the crude cinnamyl ester, which was used without further purification.

<u>Step 2 (cinnamyl ester to cinnamyl alcohol)</u>: Modified from previous report.[2] To a stirring solution of the cinnamyl ester (1.0 equiv) in THF (0.73 mL/mmol) at 0 °C (ice bath) under nitrogen was added DIBAL (2.2 equiv, 1.2M in THF) slowly over 20 min. The reaction was stirred at 0 °C for 2 h. The reaction was diluted with Et_2O (2mL/mmol). DI H_2O (0.1 mL/mmol), NaOH (2M, 0.2 mL/mmol), and DI H_2O (0.2 mL/mmol) were added and the solution was stirred at 0 °C for 30 min. The reaction was removed from the ice bath and Mg_2SO_4 was added forming a gel-like solid. The suspension was filtered through a silica pad with EtOAc (ca. 17 mL/mmol) and the solution concentrated *in vacuo* to yield the crude cinnamyl alcohol, which was used without further purification.

<u>Step 3 (cinnamyl alcohol to cinnamaldehyde)</u>: Modified from previous report.[2] Note that MnO_2 was synthesized as described previously.[2] To a stirring solution of the cinnamyl alcohol (1.0 equiv) in hexanes (0.1 M) was added the prepared MnO_2 (10.0 equiv). In cases of poor solubility, up to 2 mL/mmol of THF was added. The reaction was stirred at room temperature until consumption of the alcohol was observed (ca. 2 h). The solution was filtered through silica pad with EtOAc (ca. 12.5 mL/mmol). The filtrate was concentrated *in vacuo* to yield the crude cinnamaldehyde, which was used without further purification.

<u>Step 4 (cinnamaldehyde to 1,3-diene)</u>: Modified from previous report.[2] To a stirring solution of KO*t*-Bu (1.6 equiv) in THF (0.33 M relative to aldehyde) was added methyltriphenylphosphonium bromide (1.5 equiv). The bright yellow solution was stirred at room temperature for 0.5 h. The aldehyde (1.0 equiv) was added and the reaction stirred at room temperature until consumption of the aldehyde (ca. 1.5 h). The reaction was quenched with a saturated solution of NH_4Cl (2 mL/mmol) and ca. 90% of THF removed *in vacuo*. The resulting oil was extracted with ethyl acetate (6mL/mmol x 3). The combined organic layers were dried with Na_2SO_4 and concentrated *in vacuo* to yield the crude 1,3-diene. Purification by column chromatography (silica gel, hexanes/ethyl acetate) afforded the pure 1,3-diene. (See below for specific details on scale and purification.)

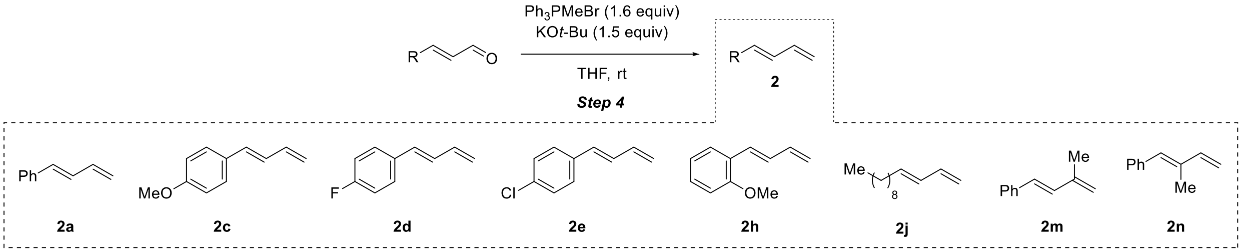

(*E*)-Buta-1,3-dien-1-ylbenzene (2a) was synthesized from cinnamaldehyde by Step 4 as previously reported[2] and matched the characterization in previous reports.[4]

(*E*)-1-(Buta-1,3-dien-1-yl)-4-methoxybenzene (2c) was synthesized from (*E*)-3-(4-methoxyphenyl) acrylaldehyde by Step 4 as previously reported[2] and matched the characterization in previous reports.[4]

(*E*)-1-(Buta-1,3-dien-1-yl)-4-fluorobenzene (2d) was synthesized from (*E*)-3-(4-fluorophenyl) acrylaldehyde by Step 4 and matched the characterization in previous reports.[5]

(*E*)-1-(Buta-1,3-dien-1-yl)-4-chlorobenzene (2e) was synthesized from (*E*)-3-(4-chlorophenyl) acrylaldehyde by Step 4 as previously reported[2] and matched the characterization in previous reports.[4]

(*E*)-1-(Buta-1,3-dien-1-yl)-2-methoxybenzene (2h) was synthesized from (*E*)-3-(2-methoxyphenyl) acrylaldehyde by Step 4 as previously reported[2] and matched the characterization in previous reports.[4]

(*E*)-trideca-1,3-diene (2j) was synthesized from (*E*)-dodec-2-enal by Step 4 on a 10.0 mmol scale. Purification by flash column chromatography (100% hexanes) afforded **2j** as a colorless oil (1.48 g, 82%). Spectra matched previous reports.[6]

(*E*)-(3-Methylbuta-1,3-dien-1-yl)benzene (2m) was synthesized from (*E*)-4-phenylbut-3-en-2-one by Step 4 as previously reported[2] and matched the characterization in previous reports.[7]

(*E*)-(2-Methylbuta-1,3-dien-1-yl)benzene (2n) was synthesized from (*E*)-2-methyl-3-phenylacrylaldehyde by Step 4 as previously reported[2] and matched the characterization in previous reports.[8]

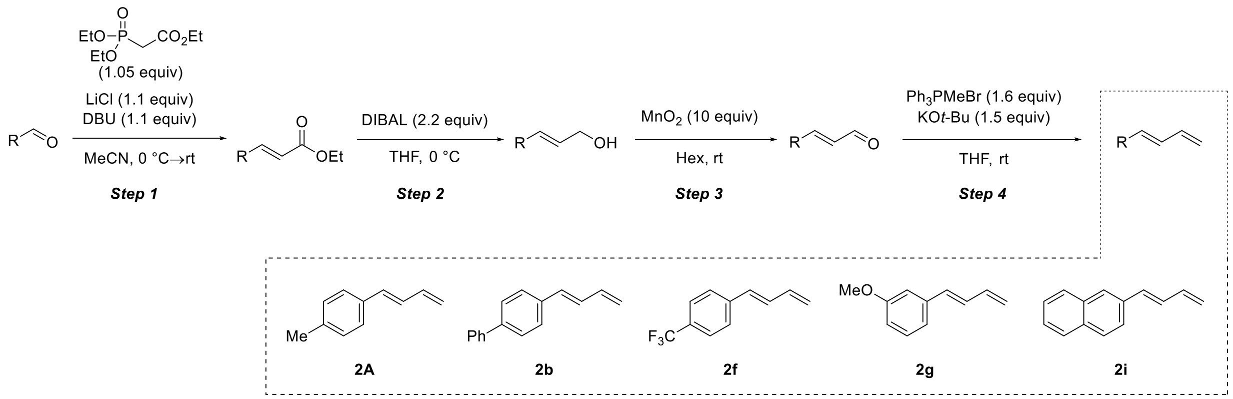

(*E*)-1-(Buta-1,3-dien-1-yl)-4-methylbenzene (2A). Synthesized from 4-methylbenzaldehyde using Standard Conditions Steps 1–4 starting on a 20.0 mmol scale. Purification conducted after Step 4 by flash column chromatography (silica gel, 100% hexanes) afforded **2A** as a colorless oil (43% over 4 steps). Spectra matched previous report.[4]

(*E*)-4-(Buta-1,3-dien-1-yl)-1,1'-biphenyl (2b). Synthesized from 4-phenylbenzaldehyde using Standard Conditions Steps 1–4 starting on a 20.0 mmol scale. Purification conducted after Step 4 by flash column chromatography (silica gel, 100% hexanes to 1% ethyl acetate–hexanes) afforded **2b** as a white solid (32% over 4 steps). Spectra matched previous report.[4]

(*E*)-1-(Buta-1,3-dien-1-yl)-4-(trifluoromethyl)benzene (2f). Synthesized from 4-(trifluoromethyl) benzaldehyde using Standard Conditions Steps 1–4 starting on a 10.0 mmol scale. Purification conducted after Step 4 by flash column chromatography (silica gel, 100% hexanes) afforded **2f** as a white solid (29% over 4 steps). Spectra matched previous report.[9]

(*E*)-1-(Buta-1,3-dien-1-yl)-3-methoxybenzene (2g). Synthesized from 3-methoxybenzaldehyde using Standard Conditions Steps 1–4 starting on a 20.0 mmol scale. Purification conducted after Step 4 by flash column chromatography (silica gel, 100% hexanes to 5% ethyl acetate–hexanes) afforded **2g** as a colorless oil (56% over 4 steps). Spectra matched previous report.[4]

(*E*)-2-(Buta-1,3-dien-1-yl)naphthalene (2i). Synthesized from 2-naphthaldehyde using Standard Conditions Steps 1–4 starting on a 20.0 mmol scale. Purification conducted after Step 4 by flash column chromatography (silica gel, 100% hexanes to 5% ethyl acetate–hexanes) afforded **2i** as a white solid (22% over 4 steps). Spectra matched previous report.[10]

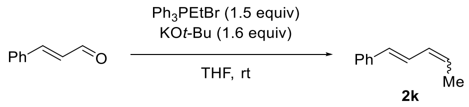

((1*E*)-penta-1,3-dien-1-yl)benzene (2k). Ethyltriphenylphosphonium bromide (5.69 g, 15.0 mmol, 1.5 equiv) and potassium *tert*-butoxide (1.80 g, 16.0 mmol, 1.6 equiv) were combined in THF (30.0 mL). The bright red-orange solution was stirred at room temperature for 0.5 h, upon which cinnamaldehyde (1.25 mL, 10.0 mmol, 1.0 equiv) was added. The reaction was stirred at room temperature for 1.75 h, upon which the aldehyde appeared consumed. The reaction was quenched with a saturated solution of NH_4Cl (20 mL) and 90% of THF was removed *in vacuo*. To the concentrated solution was added hexanes (20 mL) and the solution was stirred for 10 min (Ph_3PO crashed out slowly as a thick, goopy solid). The solution was filtered through celite, rinsing with hexanes to remove the Ph_3PO. The resulting layers were separated. The aqueous layer was extracted once with hexanes (20 mL). The combined organic layers were washed with brine (25 mL), dried (Na_2SO_4), and concentrated *in vacuo* to yield the crude diene. Purification by column chromatography (100% hexanes) afforded **2k** as a lightly yellow oil (758.7 mg, 5.3 mmol, 53%) with a 4.1:1 *Z*:*E* ratio, based on previously reported spectra,[11] which were consistent with the synthesized material.

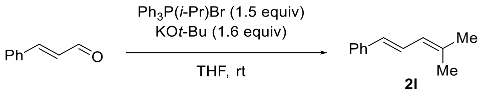

(*E,E*)-(4-Methylpenta-1,3-dien-1-yl)benzene (2l). Isopropyltriphenylphosphonium iodide (6.48 g, 15.0 mmol, 1.5 equiv) and potassium *tert*-butoxide (1.80 g, 16.0 mmol, 1.6 equiv) were combined in THF (30.0 mL). The bright red solution was stirred at room temperature for 0.5 h, upon which cinnamaldehyde (1.26 mL, 10.0 mmol, 1.0 equiv) was added. The reaction became muddy brown. The reaction was stirred at room temperature for 27.75 h, upon which the aldehyde appeared consumed. The reaction was quenched with a saturated solution of NH_4Cl (10 mL). The aqueous layer was extracted with EtOAc (30 mL x 3). The combined organic layers were washed with brine (50 mL), dried (Na_2SO_4), and concentrated *in vacuo* to yield the crude diene. Purification by column chromatography (100% hexanes to 5% ethyl acetate–hexanes) afforded **2l** as a colorless oil (1.08 g, 6.8 mmol, 68%). ***R_f*** = 0.63 (100% hexanes); **^{1}H NMR** ($CDCl_3$, 400 MHz): δ 7.40 (d, *J* = 7.1 Hz, 2H), 7.30 (t, *J* = 7.7 Hz, 2H), 7.19 (t, *J* = 7.3 Hz, 1H), 7.00 (dd, *J* = 15.5, 11.0 Hz, 1H), 6.43 (d, *J* = 15.5 Hz, 1H), 6.01 (d, *J* = 11.0 Hz, 1H), 1.87 (s, 3H), 1.85 (s, 3H); **^{13}C{^{1}H} NMR** ($CDCl_3$, 100 MHz): δ 138.1, 136.6, 129.5, 128.5 (2C), 126.9, 126.0 (2C), 125.7, 125.4, 26.2, 18.6; **FTIR** (thin film): cm^{-1} 3031, 2966, 2909, 1644, 1595, 1497, 1449, 1377, 1354, 984, 953, 746, 691; **HRMS** (ESI$^+$, m/z): Calcd for $C_{12}H_{15}^+$ ([M+H]$^+$): 159.1168; found: 159.1168.

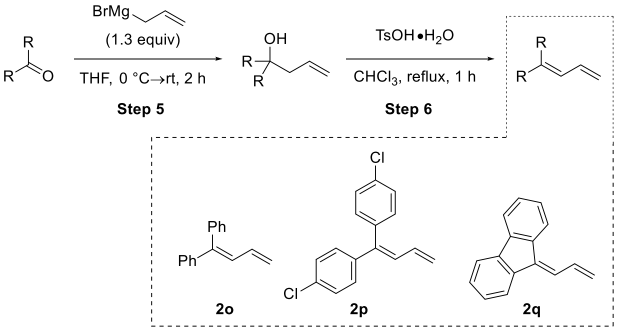

<u>Step 5 (ketone to homoallylic alcohol)</u>: Modified from previous report.[2] To a stirring anhydrous solution of benzophenone derivative (1.0 equiv) in THF (0.33 M) at 0 °C was added allylmagnesium bromide (1 M in Et_2O, 1.3 equiv) slowly over 5 min. The reaction was warmed to room temperature. After 2 h, the reaction was quenched with a saturated aqueous solution of NH_4Cl (1 mL/mmol) and acidified with HCl (2 M, 1

mL/mmol). The aqueous layer was extracted with Et_2O (2 mL/mmol x 4). The combined organic layers were washed with brine (3 mL/mmol), dried with Na_2SO_4, and concentrated in vacuo to yield the crude alcohol, which was used without further purification.

Step 6 (homoallylic alcohol to 1,3-diene): Modified from previous report.[2] To a solution of the homoallylic alcohol (1.0 equiv) in $CHCl_3$ (0.4 M) was added $TsOH{\cdot}H_2O$ (0.1 equiv). The reaction was set to reflux at 64 °C in an aluminum heating block. After 1 h, the reaction was cooled to room temperature and quenched with a saturated aqueous solution of $NaHCO_3$ (2.5 mL/mmol). The aqueous layer was extracted with DCM (2.5 mL/mmol x 3). The combined organic layers were washed with brine (6 mL/mmol), dried with Na_2SO_4, and concentrated *in vacuo* to yield the crude diene. Purification by column chromatography (silica gel, hexanes/ethyl acetate) afforded the pure 1,3-diene. (See below for specific details on scale and purification.)

Buta-1,3-diene-1,1-diyldibenzene (2o) was synthesized from benzophenone by Steps 5–6 as previously reported[2] and matched the characterization previously reported.[12]

4,4'-(Buta-1,3-diene-1,1-diyl)bis(chlorobenzene) (2p) was synthesized from 4,4'-dichlorobenzophenone by Steps 5–6 starting on a 10.0 mmol scale. Purification conducted after Step 6 by flash column chromatography (silica gel, 100% hexanes to 2% ethyl acetate–hexanes) afforded **2p** as a white solid (76% over 2 steps). Spectra matched previous report.[12]

9-Allylidene-9*H*-fluorene (2q) was synthesized from 9-fluorenone by Steps 5–6 starting on a 10.0 mmol scale. Purification conducted after Step 6 by flash column chromatography (silica gel, 100% hexanes to 2% ethyl acetate–hexanes) afforded **2q** as a white solid (11% over 2 steps). Spectra matched previous report.

4. Diene 1,2-Dioxygenation Protocols and Characterization of Novel Compounds

Standard Conditions for the 1,2-dioxygenation of 1,3-dienes

To a 1-dram vial equipped with a Teflon-coated stir bar was added the 1,3-diene **2** (0.1 mmol, 1.0 equiv). 1,2-Dichloroethane (0.33 mL) was added, followed by sequential addition of the acid **1** (0.3 mmol, 3.0 equiv) and oxyl radical **3** (0.2 mmol, 2.0 equiv). The vial was capped and stirred at 40 °C in an aluminum heating block for 24 h. The resulting crude mixture was filtered through activated, neutral Al_2O_3 (Brockman Grade I, 58–60 Å mesh powder) and concentrated *in vacuo* to yield the crude product.

4.1 TEMPO Scope

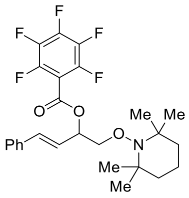

4a

(*E*)-4-Phenyl-1-((2,2,6,6-tetramethylpiperidin-1-yl)oxy)but-3-en-2-yl 2,3,4,5,6-pentafluorobenzoate (4a). Synthesized using standard conditions. Isolated by flash column chromatography (100% hexanes to 2% ethyl acetate–hexanes) as a clear oil (26.7 mg, 54%).

0.5 mmol scale-up: To a 2-dram vial equipped with a teflon-coated stir bar was added 1-phenyl-1,3-butadiene **2a** (65.1 mg, 0.5 mmol, 1.0 equiv). 1,2-Dichloroethane (1.67 mL) was added, followed by sequential addition of pentafluorobenzoic acid **1a** (318.1 mg, 1.5 mmol, 3.0 equiv) and TEMPO **3a** (156.3 mg, 1.0 mmol, 2.0 equiv). The vial was capped and stirred at 40 °C for 24 h. The resulting crude mixture was filtered through activated, neutral Al_2O_3 (Brockman Grade I, 58–60 Å mesh powder) and concentrated

in vacuo to yield the crude product. Isolation by flash column chromatography (100% hexanes to 10% ethyl acetate-hexanes) afforded the product as a colorless oil (136.4 mg, 55%).

Spectra matched previous report.[2] (^{1}H and ^{13}C NMRs characterization and spectra provided below as C–F couplings in the ^{13}C NMR were not determined in the previous report.) **^{1}H NMR** ($CDCl_3$, 400 MHz): δ 7.40 (d, *J* = 7.3 Hz, 2H), 7.34 (t, *J* = 7.3 Hz, 2H), 7.28 (t, *J* = 7.2 Hz, 1H), 6.79 (d, *J* = 16.0 Hz, 1H), 6.25 (dd, *J* = 16.0, 7.4 Hz, 1H), 5.90 (td, *J* = 7.3, 3.5 Hz, 1H), 4.10 (dd, *J* = 10.3, 7.2 Hz, 1H), 4.03 (dd, *J* = 10.3, 3.6 Hz, 1H), 1.58–1.41 (m, 5H), 1.36–1.27 (m, 1H), 1.19 (s, 3H), 1.16 (s, 3H), 1.12 (s, 3H), 1.09 (s, 3H); **^{13}C{^{1}H} NMR** ($CDCl_3$, 100 MHz): δ 158.3, 145.4 (d, $J_{C\text{–}F}$ = 258.6 Hz, 2C), 143.1 (d, $J_{C\text{–}F}$ = 259.1 Hz, 1C), 137.6 (d, $J_{C\text{–}F}$ = 255.3 Hz, 2C), 135.9, 134.5, 128.6 (2C), 128.3, 126.7 (2C), 123.0, 108.4 (t, $J_{C\text{–}F}$ = 15.4 Hz, 1C), 77.4, 75.9, 60.1, 60.1, 39.6, 39.6, 32.9, 32.8, 19.9 (2C), 17.0.

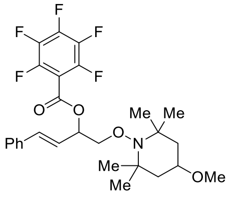

4d

(*E*)-1-((4-Methoxy-2,2,6,6-tetramethylpiperidin-1-yl)oxy)-4-phenylbut-3-en-2-yl 2,3,4,5,6-pentafluorobenzoate (4d). Synthesized using standard conditions. Isolated by flash column chromatography (100% hexanes to 5% ethyl acetate–hexanes) as a white solid (38.1 mg, 72%).

1.0 mmol scale-up: To a 2-dram vial equipped with a teflon-coated stir bar was added 1-phenyl-1,3-butadiene **2a** (130.2 mg, 1.0 mmol, 1.0 equiv). 1,2-Dichloroethane (3.33 mL) was added, followed by sequential addition of pentafluorobenzoic acid **1a** (636.2 mg, 3.0 mmol, 3.0 equiv) and 4-methoxyTEMPO **3d** (372.6 mg, 2.0 mmol, 2.0 equiv). The vial was capped and stirred at 40 °C for 24 h. The resulting crude mixture was filtered through activated, neutral Al_2O_3 (Brockman Grade I, 58–60 Å mesh powder) and concentrated *in vacuo* to yield the crude product. Isolation by flash column chromatography (silica gel, 100% hexanes to 15% ethyl acetate-hexanes) afforded the product as a white solid (367.4 mg, 70%).

R_f = 0.76 (25% EtOAc–hexanes); **^{1}H NMR** ($CDCl_3$, 400 MHz): δ 7.40 (d, *J* = 7.5 Hz, 2H), 7.33 (t, *J* = 7.4 Hz, 2H), 7.28 (t, *J* = 7.1 Hz, 1H), 6.79 (d, *J* = 15.9 Hz, 1H), 6.24 (dd, *J* = 16.0, 7.3 Hz, 1H), 5.91 (td, *J* = 7.3, 3.3 Hz, 1H), 4.11 (dd, *J* = 10.3, 7.3 Hz, 1H), 4.04 (dd, *J* = 10.4, 3.4 Hz, 1H), 3.44 (tt, *J* = 11.5, 4.2 Hz, 1H), 3.32 (s, 3H), 1.88–1.83 (m, 2H), 1.38 (t, *J* = 12.1 Hz, 2H), 1.23 (s, 3H), 1.21 (s, 3H), 1.17 (s, 3H), 1.14 (s, 3H); **^{13}C{^{1}H} NMR** ($CDCl_3$, 100 MHz): δ 158.1, 145.1 (d, $J_{C\text{–}F}$ = 257.4 Hz, 2C), 142.9 (d, $J_{C\text{–}F}$ = 259.3 Hz, 1C), 138.7 (d, $J_{C\text{–}F}$ = 255.9 Hz, 2C), 134.5, 128.4, 128.1, 126.5, 122.6, 108.2 (t, $J_{C\text{–}F}$ = 15.6 Hz, 1C), 75.6, 71.4, 60.0 (2C), 55.5, 44.3, 44.2, 32.9, 32.8, 20.7 (2C); **^{19}F NMR** ($CDCl_3$, 376.5 MHz): δ –138.4 (ddt, *J* = 25.2, 6.2, 5.7 Hz, 2F), –149.1 (tt, *J* = 20.9, 4.6 Hz, 1F), –160.8 (ddt, *J* = 26.1, 20.1, 5.8 Hz, 2F); **FTIR** (thin film): cm^{-1} 2975, 2935, 1738, 1652, 1523, 1496, 1326, 1222, 1097, 997; **HRMS** (ESI^+, m/z): Calcd for $C_{27}H_{31}F_5NO_4^+$ ($[M+H]^+$): 528.2168; found: 528.2151.

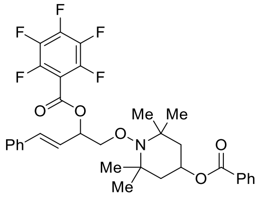

4e

(*E*)-1-((4-(Benzoyloxy)-2,2,6,6-tetramethylpiperidin-1-yl)oxy)-4-phenylbut-3-en-2-yl 2,3,4,5,6-pentafluorobenzoate (4e). Synthesized using standard conditions. Isolated by flash column chromatography (silica gel, 100% hexanes to 5% ethyl acetate–hexanes) as a white solid (36.0 mg, 58%). ***R_f*** = 0.82 (25% EtOAc–hexanes); **^{1}H NMR** ($CDCl_3$, 400 MHz): δ 7.93 (d, *J* = 8.4 Hz, 1H), 7.47 (t, *J* = 6.9 Hz, 1H), 7.39–7.30 (m, 4H), 7.26 (t, *J* = 7.2 Hz, 2H), 7.23–7.16 (m, 1H), 6.72 (d, *J* = 16.0 Hz, 1H), 6.18 (dd,

J = 16.0, 7.4 Hz, 1H), 5.88–5.80 (m, 1H), 5.25–5.13 (m, 1H), 4.10–4.02 (m, 1H), 4.02–3.96 (m, 1H), 1.94–1.86 (m, 2H), 1.63 (t, *J* = 1.63 Hz, 2H), 1.20 (s, 6H), 1.17 (s, 6H); ^{13}**C{^{1}H} NMR** ($CDCl_3$, 100 MHz): δ 166.1, 158.3, 145.4 (d, $J_{C–F}$ = 265.4 Hz, 2C), 143.1 (d, $J_{C–F}$ = 259.6 Hz, 1C), 137.7 (d, $J_{C–F}$ = 260.1 Hz, 2C), 135.7, 134.7, 132.9, 130.4, 129.4 (2C), 128.6 (2C), 128.4, 128.3 (2C), 126.7 (2C), 122.7, 108.2 (t, $J_{C–F}$ = 15.4 Hz, 1C), 77.6, 75.8, 67.1, 60.4, 60.4, 44.1, 44.0, 32.9, 32.9, 20.8 (2C); ^{19}**F NMR** ($CDCl_3$, 376.5 MHz): δ –138.4 (ddt, *J* = 25.2, 5.9, 5.5 Hz, 2F), –148.9 (tt, *J* = 20.8, 4.4 Hz, 1F), –160.7 (ddt, *J* = 26.2, 20.1, 5.9 Hz, 2F); **FTIR** (thin film): cm^{-1} 2976, 2941, 1737, 1716, 1652, 1523, 1495, 1314, 1275, 1223, 1113, 998, 712; **HRMS** (ESI^+, m/z): Calcd for $C_{33}H_{33}F_5NO_5^+$ ($[M+H]^+$): 618.2273; found: 618.2274.

4.2 Carboxylic Acid Scope

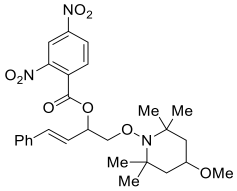

5a

(*E*)-1-((4-Methoxy-2,2,6,6-tetramethylpiperidin-1-yl)oxy)-4-phenylbut-3-en-2-yl 2,4-dinitrobenzoate (5a). Synthesized using standard conditions. Isolated by flash column chromatography (silica gel, 100% hexanes to 15% ethyl acetate–hexanes) as a white solid (37.2 mg, 71%). R_f = 0.54 (25% EtOAc–hexanes); ^{1}**H NMR** ($CDCl_3$, 400 MHz): δ 8.74 (d, *J* = 2.3 Hz, 1H), 8.47 (dd, *J* = 8.4, 2.1 Hz, 1H), 7.91 (d, *J* = 8.4 Hz, 1H), 7.37 (d, *J* = 7.5 Hz, 2H), 7.29 (t, *J* = 7.4 Hz, 2H), 7.23 (t, *J* = 6.9 Hz, 1H), 6.76 (d, *J* = 16.0 Hz, 1H), 6.19 (dd, *J* = 16.0, 7.7 Hz, 1H), 5.83 (td, *J* = 7.3, 3.5 Hz, 1H), 4.06 (dd, *J* = 10.4, 7.1 Hz, 1H), 4.00 (dd, *J* = 10.4, 3.6 Hz, 1H), 3.38 (tt, *J* =11.4, 4.2 Hz, 1H), 3.27 (s, 3H), 1.85–1.76 (m, 2H), 1.32 (t, *J* = 12.1 Hz, 2H), 1.17 (s, 3H), 1.14 (s, 3H), 1.12 (s, 3H), 1.10 (s, 3H); ^{13}**C{^{1}H} NMR** ($CDCl_3$, 100 MHz): δ 162.8, 148.7, 147.9, 135.6, 135.2, 132.9, 131.0, 128.5 (2C), 128.3, 127.3, 126.6 (2C), 122.2, 119.5, 77.2, 76.4, 71.4, 60.1 (2C), 55.6, 44.4, 44.3, 33.0, 32.9, 20.9, 20.8; **FTIR** (thin film): cm^{-1} 2975, 2936, 1736, 1604, 1537, 1346, 1277, 1243, 1093, 730, 693; **HRMS** (ESI^+, m/z): Calcd for $C_{27}H_{34}N_3O_8^+$ ($[M+H]^+$): 528.2340; found: 528.2340.

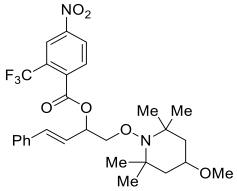

5b

(*E*)-1-((4-Methoxy-2,2,6,6-tetramethylpiperidin-1-yl)oxy)-4-phenylbut-3-en-2-yl 4-nitro-2-(trifluoromethyl)benzoate (5b). Synthesized using standard conditions. Isolated by flash column chromatography (silica gel, 100% hexanes to 5% ethyl acetate–hexanes) as a colorless oil (40.1 mg, 73%). R_f = 0.62 (25% EtOAc–hexanes); ^{1}**H NMR** ($CDCl_3$, 400 MHz): δ 8.67 (d, *J* = 1.7 Hz, 1H), 8.52 (dd, *J* = 8.0, 1.8 Hz, 1H), 8.05 (d, *J* = 8.1 Hz, 1H), 7.45 (d, *J* = 7.4 Hz, 2H), 7.39 (t, *J* = 7.3 Hz, 2H), 7.33 (t, *J* = 7.2 Hz, 1H), 6.86 (d, *J* = 15.9 Hz, 1H), 6.30 (dd, *J* = 15.9, 7.7 Hz, 1H), 5.94 (td, *J* = 7.4, 3.3 Hz, 1H), 4.19 (dd, *J* = 10.3, 7.2 Hz, 1H), 4.10 (dd, *J* = 10.3, 3.5 Hz, 1H), 3.46 (tt, *J* = 11.3, 4.0 Hz, 1H), 3.37 (s, 3H), 1.94–1.86 (m, 2H), 1.43 (t, *J* = 11.9 Hz, 2H), 1.28 (s, 3H), 1.25 (s, 3H), 1.21 (s, 3H), 1.20 (s, 3H); ^{13}**C{^{1}H} NMR** ($CDCl_3$, 100 MHz): δ 163.2, 147.8, 134.9, 134.3, 130.7, 129.5 (q, $^{2}J_{C–F}$ = 34.2 Hz, 1C), 127.8 (2C), 127.7 (q, $^{3}J_{C–F}$ = 11.7 Hz, 1C), 127.5, 125.8 (2C), 125.7, 121.8, 121.4 (q, $^{3}J_{C–F}$ = 5.3 Hz, 1C), 121.2 (q, $^{1}J_{C–F}$ = 274.5 Hz ,1C), 75.2, 70.7, 59.3 (2C), 54.9, 43.6, 43.6, 32.2, 32.2, 20.1, 20.0; ^{19}**F NMR** ($CDCl_3$, 376.5 MHz): δ –60.1 (s, 3F); **FTIR** (thin film): cm^{-1} 2933, 1740, 1617, 1537, 1355, 1290, 1254, 1150, 1096, 1046; **HRMS** (ESI^+, m/z): Calcd for $C_{28}H_{34}F_3N_2O_6^+$ ($[M+H]^+$): 551.2363; found: 551.2364.

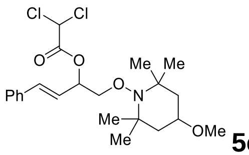

5e

(*E*)-1-((4-Methoxy-2,2,6,6-tetramethylpiperidin-1-yl)oxy)-4-phenylbut-3-en-2-yl 2,2-dichloroacetate (5e). Synthesized using standard conditions. Isolated by flash column chromatography (silica gel, 100% hexanes to 15% ethyl acetate–hexanes) as a colorless oil (35.9 mg, 81%). R_f = 0.71 (25% EtOAc–hexanes); **^{1}H NMR** ($CDCl_3$, 400 MHz): δ 7.43 (d, *J* = 7.5 Hz, 2H), 7.38 (t, *J* = 7.3 Hz, 2H), 7.33 (t, *J* = 7.0 Hz, 1H), 6.81 (d, *J* = 15.9 Hz, 1H), 6.22 (dd, *J* = 15.9, 7.3 Hz, 1H), 6.02 (s, 1H), 5.75 (td, *J* = 7.5, 3.0 Hz, 1H), 4.12 (dd, *J* = 10.3, 7.6 Hz, 1H), 4.04 (dd, *J* = 10.5, 3.0 Hz ,1H), 3.48 (tt, *J* = 11.3, 3.9 Hz, 1H), 3.37 (s, 3H), 1.91 (dd, *J* = 12.7, 3.9 Hz, 2H), 1.42 (t, *J* = 11.8 Hz, 2H), 1.29 (s, 3H), 1.27 (s, 3H), 1.22 (s, 3H), 1.20 (s, 3H); **^{13}C{^{1}H} NMR** ($CDCl_3$, 100 MHz): δ 162.8, 134.8, 134.1, 127.8 (2C), 127.6, 125.9 (2C), 121.3, 76.4, 75.7, 70.7, 63.8, 59.5, 59.4, 54.9, 43.7, 43.6, 32.3, 32.2, 20.1, 20.1; **FTIR** (thin film): cm^{-1} 2973, 2934, 1763, 1695, 1626, 1597, 1515, 1465, 1255, 1168, 1096, 741; **HRMS** (ESI$^+$, m/z): Calcd for $C_{22}H_{32}Cl_2NO_4^+$ ([M+H]$^+$): 444.1703; found: 444.1705.

4.3 Diene Scope

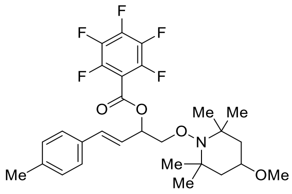

6a

(*E*)-1-((4-Methoxy-2,2,6,6-tetramethylpiperidin-1-yl)oxy)-4-(p-tolyl)but-3-en-2-yl 2,3,4,5,6-pentafluorobenzoate (6a). Synthesized using standard conditions. Isolated by flash column chromatography (silica gel, 100% hexanes to 5% ethyl acetate–hexanes) as a white solid (37.0 mg, 68%). R_f = 0.77 (25% EtOAc–hexanes); **^{1}H NMR** ($CDCl_3$, 400 MHz): δ 7.31 (d, *J* = 7.6 Hz, 2H), 7.16 (d, *J* = 7.6 Hz, 2H), 6.77 (d, *J* = 16.0 Hz, 1H), 6.20 (dd, *J* = 16.0, 7.4 Hz, 1H), 5.91 (td, *J* = 7.3, 2.8 Hz, 1H), 4.12 (dd, *J* = 9.9, 7.5 Hz, 1H), 4.05 (dd, *J* = 10.1, 2.5 Hz, 1H), 3.49–3.40 (m, 1H), 3.34 (s, 3H), 2.36 (s, 3H), 1.92–1.83 (m, 2H), 1.39 (t, *J* = 12.1 Hz, 2H), 1.25 (s, 3H), 1.22 (s, 3H), 1.19 (s, 3H), 1.16 (s, 3H); **^{13}C{^{1}H} NMR** ($CDCl_3$, 100 MHz): δ 158.0, 145.1 (d, $J_{C–F}$ = 258.2 Hz, 2C), 143.5 (d, $J_{C–F}$ = 259.6 Hz, 1C), 138.0, 137.2 (d, $J_{C–F}$ = 254.9 Hz, 2C), 134.4, 132.7, 129.0 (2C), 126.3 (2C), 121.3, 108.1 (t, $J_{C–F}$ = 16.3 Hz, 1C), 77.3, 75.7, 71.3, 60.0, 59.9, 55.5, 44.2, 44.1, 32.8, 32.7, 20.9, 20.6 (2C); **^{19}F NMR** ($CDCl_3$, 376.5 MHz): δ –138.4 (ddt, *J* = 24.8, 5.8, 5.3 Hz, 2F), –149.2 (tt, *J* = 20.8, 4.6 Hz, 1F), –160.8 (ddt, *J* = 26.2, 20.0, 5.8 Hz, 2F); **FTIR** (thin film): cm^{-1} 2975, 2934, 1737, 1652, 1523, 1496, 1330, 1222, 1097, 996; **HRMS** (ESI$^+$, m/z): Calcd for $C_{28}H_{33}F_5NO_4^+$ ([M+H]$^+$): 542.2324; found: 542.2326.

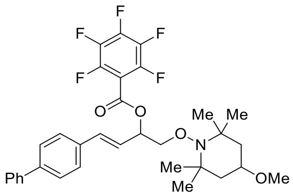

6b

(*E*)-4-([1,1'-Biphenyl]-4-yl)-1-((4-methoxy-2,2,6,6-tetramethylpiperidin-1-yl)oxy)but-3-en-2-yl 2,3,4,5,6-pentafluorobenzoate (6b). Synthesized using standard conditions. Isolated by flash column chromatography (silica gel, 100% hexanes to 5% ethyl acetate–hexanes) as a white solid (42.3 mg, 70%). R_f = 0.70 (25% EtOAc–hexanes); **^{1}H NMR** ($CDCl_3$, 400 MHz): δ 7.69–7.61 (m, 4H), 7.54 (d, *J* = 8.5 Hz,

2H), 7.50 (t, *J* = 7.8 Hz, 2H), 7.41 (t, *J* = 7.2 Hz, 1H), 6.89 (d, *J* = 15.9 Hz, 1H), 6.35 (dd, *J* = 15.9, 7.2 Hz, 1H), 5.99 (td, *J* = 7.2, 3.4 Hz, 1H), 4.19 (dd, *J* = 10.3, 7.2 Hz, 1H), 4.12 (dd, *J* = 10.7, 3.2 Hz, 1H), 3.51 (tt, *J* = 10.8, 3.9 Hz, 1H), 3.39 (s, 3H), 1.97–1.90 (m, 2H), 1.45 (t, *J* = 12.0 Hz, 2H), 1.31 (s, 3H), 1.28 (s, 3H), 1.25 (s, 3H), 1.22 (s, 3H); **$^{13}C\{^1H\}$ NMR** ($CDCl_3$, 100 MHz): δ 157.2, 144.3 (d, $J_{C–F}$ = 268.3 Hz, 2C), 142.1 (d, $J_{C–F}$ = 258.2 Hz, 1C), 140.1, 139.4, 136.6 (d, $J_{C–F}$ = 250.9 Hz, 2C), 133.7, 133.2, 127.8 (2C), 126.4, 126.3 (2C), 126.1 (2C), 125.9 (2C), 121.8, 107.3 (t, $J_{C–F}$ = 15.4 Hz, 1C), 74.8, 70.6, 59.2 (2C), 54.8, 43.5, 43.4, 32.1, 32.0, 19.9 (2C); **^{19}F NMR** ($CDCl_3$, 376.5 MHz): δ –138.5 (d, *J* = 18.6 Hz, 2F), –149.1 (t, *J* = 21.1 Hz, 1F), –160.9 (t, *J* = 18.6 Hz, 2F); **FTIR** (thin film): cm^{-1} 2974, 2935, 1738, 1652, 1523, 1496, 1323, 1222, 1095, 995, 762, 732, 697; **HRMS** (ESI^+, m/z): Calcd for $C_{33}H_{35}F_5NO_4^+$ ($[M+H]^+$): 604.2481; found: 604.2485.

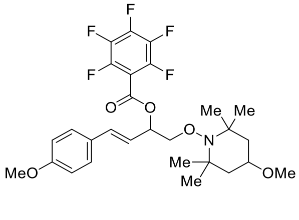

6c

(*E*)-1-((4-Methoxy-2,2,6,6-tetramethylpiperidin-1-yl)oxy)-4-(4-methoxyphenyl)but-3-en-2-yl 2,3,4,5,6-pentafluorobenzoate (6c). Synthesized using standard conditions. Isolated by flash column chromatography (silica gel, 100% hexanes to 5% ethyl acetate–hexanes) as a white solid (27.4 mg, 49%). [Note that this compound is highly susceptible to decomposition, particularly when exposed to silica gel. ***R_f*** = 0.62 (25% EtOAc–hexanes); **^1H NMR** ($CDCl_3$, 400 MHz): δ 7.35 (d, *J* = 8.3 Hz, 2H), 6.88 (d, *J* = 8.3 Hz, 2H), 6.75 (d, *J* = 15.8 Hz, 1H), 6.11 (dd, *J* = 15.9, 7.5 Hz, 1H), 5.90 (td, *J* = 7.4, 3.2 Hz, 1H), 4.12 (dd, *J* = 10.3, 7.3 Hz, 1H), 4.04 (dd, *J* = 10.4, 3.3 Hz, 1H), 3.83 (s, 3H), 3.45 (tt, *J* = 11.3, 4.0 Hz, 1H), 3.34 (s, 3H), 1.94–1.83 (m, 2H), 1.40 (t, *J* = 12.0 Hz, 2H), 1.25 (s, 3H), 1.22 (s, 3H), 1.19 (s, 3H), 1.16 (s, 3H); **$^{13}C\{^1H\}$ NMR** ($CDCl_3$, 100 MHz): δ 159.4, 157.9, 144.9 (d, $J_{C–F}$ = 242.8 Hz, 2C), 142.7 (d, $J_{C–F}$ = 259.7 Hz, 1C), 137.2 (d, $J_{C–F}$ = 250.9 Hz, 2C), 134.0, 128.1, 127.6 (2C), 120.0, 113.6 (2C), 108.1 (t, $J_{C–F}$ = 15.8 Hz, 1C), 77.2, 75.7, 71.2, 59.8, 59.8, 55.4, 54.9, 44.2, 44.1, 32.7, 32.6, 20.5 (2C); **^{19}F NMR** ($CDCl_3$, 376.5 MHz): δ –138.4 (ddt, *J* = 25.0, 6.2, 5.4 Hz, 2F), –149.4 (tt, *J* = 20.8, 4.5 Hz, 1F), –160.9 (ddt, *J* = 26.8, 20.2, 5.8 Hz, 2F); **FTIR** (thin film): cm^{-1} 2975, 2936, 1737, 1652, 1607, 1496, 1222, 1174, 1096, 996; **HRMS** (ESI^+, m/z): Calcd for $C_{28}H_{33}F_5NO_5^+$ ($[M+H]^+$): 558.2273; found: 558.2274.

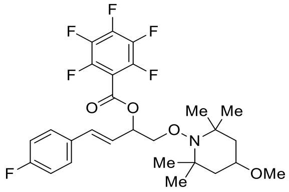

6d

(*E*)-4-(4-Fluorophenyl)-1-((4-methoxy-2,2,6,6-tetramethylpiperidin-1-yl)oxy)but-3-en-2-yl 2,3,4,5,6-pentafluorobenzoate (6d). Synthesized using standard conditions. Isolated by flash column chromatography (silica gel, 100% hexanes to 10% ethyl acetate–hexanes) as a colorless oil (36.2 mg, 66%). ***R_f*** = 0.67 (25% EtOAc–hexanes); **^1H NMR** ($CDCl_3$, 400 MHz): δ 7.37 (dd, *J* = 8.6, 5.5 Hz, 2H), 7.02 (t, *J* = 8.6 Hz, 2H), 6.75 (d, *J* = 15.9 Hz, 1H), 6.16 (dd, *J* = 16.0, 7.3 Hz, 1H), 5.88 (td, *J* = 7.4, 3.3 Hz, 1H), 4.10 (dd, *J* = 10.3, 7.2 Hz, 1H), 4.03 (dd, *J* = 10.4, 3.5 Hz, 1H), 3.43 (tt, *J* = 11.5, 4.1 Hz, 1H), 3.32 (s, 3H), 1.89–1.81 (m, 2H), 1.37 (t, *J* = 12.1 Hz, 2H), 1.23 (s, 3H), 1.20 (s, 3H), 1.17 (s, 3H), 1.14 (s, 3H); **$^{13}C\{^1H\}$ NMR** ($CDCl_3$, 100 MHz): δ 162.6 (d, $J_{C–F}$ = 247.96 Hz, 1C), 158.2, 145.3 (d, $J_{C–F}$ = 258.5 Hz, 2C), 143.1 (d, $J_{C–F}$ = 259.4 Hz, 1C), 137.6 (d, $J_{C–F}$ = 137.5 Hz, 2C), 133.4, 131.9 (d, *J* = 3.4 Hz, 1C), 128.2 (d, $J_{C–F}$ = 8.1 Hz, 2C), 122.5 (d, $J_{C–F}$ = 2.3 Hz, 1C), 115.6 (d, $J_{C–F}$ = 21.8 Hz, 2C), 108.2 (t, $J_{C–F}$ = 14.1 Hz, 1C), 77.4, 75.7, 71.5, 60.2, 60.2, 55.7, 44.5, 44.4, 33.0, 32.9, 20.8 (2C); **^{19}F NMR** ($CDCl_3$, 376.5 MHz): δ –113.7 (s, 1F), –138.5 (m, 2F), –149.1 (t, *J* = 20.6 Hz, 1F), –160.9 (m, 2F); **FTIR** (thin film): cm^{-1} 2975, 2937, 1738, 1652, 1602, 1496, 1329, 1222, 1096, 996, 968, 937; **HRMS** (ESI, m/z): Calcd for $C_{27}H_{30}F_6NO_4^+$ ($[M+H]^+$): 546.2074; found: 546.2071.

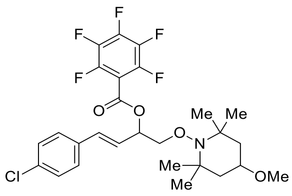

6e

(*E*)-4-(4-Chlorophenyl)-1-((4-methoxy-2,2,6,6-tetramethylpiperidin-1-yl)oxy)but-3-en-2-yl 2,3,4,5,6-pentafluorobenzoate (6e). Synthesized using standard conditions. Isolated by flash column chromatography (silica gel, 100% hexanes to 5% ethyl acetate–hexanes) as a white solid (35.2 mg, 63%). ***R_f*** = 0.72 (25% EtOAc–hexanes); **^{1}H NMR** ($CDCl_3$, 400 MHz): δ 7.37–7.30 (m, 4H), 6.76 (d, *J* = 16.0 Hz, 1H), 6.25 (dd, *J* = 15.9, 7.2 Hz, 1H), 5.91 (td, *J* = 7.2, 3.2 Hz, 1H), 4.12 (dd, *J* = 10.3, 7.2 Hz, 1H), 4.06 (dd, *J* = 10.5, 3.6 Hz, 1H), 3.46 (tt, *J* = 11.6, 4.0 Hz, 1H), 3.35 (s, 3H), 1.92–1.85 (m, 2H), 1.40 (t, *J* = 12.0 Hz, 2H), 1.25 (s, 3H), 1.22 (s, 3H), 1.20 (s, 3H), 1.16 (s, 3H); **^{13}C{^{1}H} NMR** ($CDCl_3$, 100 MHz): δ 157.7, 144.8 (d, $J_{C–F}$ = 263.5 Hz, 2C), 142.6 (d, $J_{C–F}$ = 259.6 Hz, 1C), 137.1 (d, $J_{C–F}$ = 255.4 Hz, 2C), 133.7, 133.4, 132.7, 128.3 (2C), 127.3 (2C), 122.9, 107.6 (t, $J_{C–F}$ = 17.3 Hz, 1C), 76.8, 71.0, 59.7 (2C), 55.2, 43.9, 43.8, 32.5, 32.4, 20.3 (2C); **^{19}F NMR** ($CDCl_3$, 376.5 MHz): δ –138.3 (ddt, *J* = 24.8, 5.8, 5.4 Hz, 2F), –149.0 (tt, *J* = 21.1, 4.4 Hz, 1F), –160.7 (ddt, *J* = 26.0, 20.0, 5.7 Hz, 2F); **FTIR** (thin film): cm^{-1} 2975, 2934, 1738, 1652, 1523, 1493, 1328, 1221, 1093, 996, 968, 938; **HRMS** (ESI^+, m/z): Calcd for $C_{27}H_{30}ClF_5NO_4^+$ ($[M+H]^+$): 562.1778; found: 562.1783.

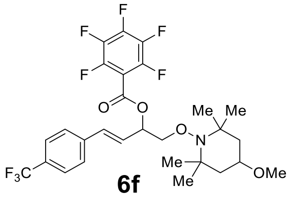

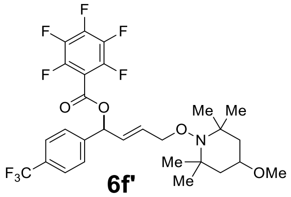

(*E*)-1-((4-Methoxy-2,2,6,6-tetramethylpiperidin-1-yl)oxy)-4-(4-(trifluoromethyl)phenyl)but-3-en-2-yl 2,3,4,5,6-pentafluorobenzoate (6f) and (*E*)-4-((4-Methoxy-2,2,6,6-tetramethylpiperidin-1-yl)oxy)-1-(4-(trifluoromethyl)phenyl)but-2-en-1-yl 2,3,4,5,6-pentafluorobenzoate (6f'). Synthesized using standard conditions. A ratio of 1.3:1 for **6f**:**6f'** was determined by ^{19}F NMR of the crude reaction. Isolated by flash column chromatography (silica gel, 100% hexanes to 10% ethyl acetate–hexanes) as a clear oil and an inseparable 2.2:1 mixture of **6f** and **6f'** (17.0 mg, 29%). ***R_f*** = 0.86 (25% EtOAc–hexanes); **^{1}H NMR** ($CDCl_3$, 400 MHz): δ 7.61 (d, *J* = 7.7 Hz, 2H, *6f'*) 7.54 (d, *J* = 7.9 Hz, 2H, *6f*), 7.50 (d, *J* = 7.9 Hz, 2H, *6f'*), 7.44 (d, *J* = 7.9 Hz, 2H, *6f*), 6.76 (d, *J* = 16.0 Hz, 1H, *6f*), 6.51 (d, *J* = 5.1 Hz, 1H, *6f'*), 6.29 (ddd, *J* = 15.9, 7.0, 1.8 Hz, 1H, *6f*), 5.91–5.82 (m, 1H each *6f*+*6f'*), 4.29 (s, 2H, *6f'*), 4.05 (dd, *J* = 10.3, 6.8 Hz, 1H, *6f*), 4.03–3.97 (m, 1H, *6f*), 3.38 (t, *J* = 11.4 Hz, 1H each, *6f*+ *6f'*), 3.27 (s, 3H each, *6f*+*6f'*), 1.84–1.77 (m, 2H each, *6f*+*6f'*), 1.37–1.27 (m, 2H each, *6f*+*6f'*), 1.18 (s, 3H, *6f*), 1.15 (s, 3H, *6f*), 1.12 (s, 3H, *6f*; 6H, *6f'*), 1.09 (s, 3H, *6f*; 6H, *6f'*); **^{13}C{^{1}H} NMR** ($CDCl_3$, 100 MHz): δ 158.2 (*6f*), 157.9 (*6f'*), 145.4 (d, $J_{C–F}$ = 256.6 Hz, 2C each, *6f*+*6f'*), 143.2 (d, $J_{C–F}$ = 250.5 Hz, 1C each, *6f*+*6f'*), 141.8 (*6f'*), 139.2 (*6f*), 137.6 (d, $J_{C–F}$ = 254.2 Hz, 2C each, *6f*+*6f'*), 132.8 (*6f*), 131.5 (*6f'*), 127.4 (2C, *6f'*), 127.1 (*6f'*), 126.8 (2C, *6f*), 125.6 (3C, *6f*; 2C, *6f'*), 108.3–107.6 (m, 1C each, *6f*+*6f'*), 77.7 (*6f*), 76.4 (*6f'*), 75.2 (*6f*), 71.7 (*6f'*), 71.5 (*6f*), 60.2 (2C, *6f*), 59.9 (2C each, *6f'*), 55.7 (*6f*+*6f'*), 44.4 (2C each, *6f*+*6f'*), 33.0 (2C each, *6f*+*6f'*), 21.1 (2C, *6f'*), 20.8 (2C, *6f*); **^{19}F NMR** ($CDCl_3$, 376.5 MHz): δ –63.2 (s, 3F, *6f*), –63.4 (s, 3F, *6f'*), –138.4 (2F each, *6f*+*6f'*), –148.4 (t, *J* = 20.3 Hz, 1F, *6f'*), –148.8 (t, *J* = 20.8 Hz, 1F, *6f*), –160.7 (m, 2F each, *6f*+*6f'*); **FTIR** (thin film): cm^{-1} 2977, 2937, 1741, 1652, 1617, 1524, 1498, 1324, 1224, 1168, 1128, 1098, 1067, 998; **HRMS** (ESI, m/z): Calcd for $C_{28}H_{30}F_8NO_4^+$ ($[M+H]^+$): 596.2042; found: 596.2043.

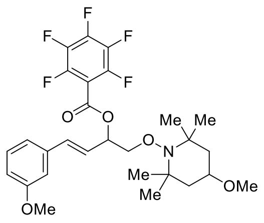

6g

(*E*)-1-((4-Methoxy-2,2,6,6-tetramethylpiperidin-1-yl)oxy)-4-(3-methoxyphenyl)but-3-en-2-yl 2,3,4,5,6-pentafluorobenzoate (6g). Synthesized using standard conditions. Isolated by flash column chromatography (silica gel, 100% hexanes to 10% ethyl acetate–hexanes) as a colorless oil (36.2 mg, 65%). R_f = 0.69 (25% EtOAc–hexanes); **^{1}H NMR** ($CDCl_3$, 400 MHz): δ 7.30 (t, *J* = 7.9 Hz, 1H), 7.04 (d, *J* = 7.5 Hz, 1H), 6.97 (s, 1H), 6.89 (d, *J* = 8.1 Hz, 1H), 6.81 (d, *J* = 15.9 Hz, 1H), 6.28 (dd, *J* = 15.9, 7.1 Hz, 1H), 5.98–5.92 (m, 1H), 4.15 (dd, *J* = 10.2, 7.2 Hz, 1H), 4.09 (dd, *J* = 10.4, 3.2 Hz, 1H), 3.87 (s, 3H), 3.48 (tt, *J* = 10.5, 3.9 Hz, 1H), 3.37 (s, 3H), 1.95–1.86 (m, 2H), 1.42 (t, *J* = 11.9 Hz, 2H), 1.28 (s, 3H), 1.25 (s, 3H), 1.22 (s, 3H), 1.19 (s, 3H); **^{13}C{^{1}H} NMR** ($CDCl_3$, 100 MHz): δ 158.8, 157.3, 144.4 (d, $J_{C–F}$ = 263.0 Hz, 2C), 142.2 (d, $J_{C–F}$ = 264.7 Hz, 1C), 136.8 (d, $J_{C–F}$ = 256.9 Hz, 2C), 136.3, 133.6, 128.7, 122.2, 118.4, 113.1, 111.0, 107.4, (t, $J_{C–F}$ = 16.0 Hz, 1C), 76.6, 74.8, 70.7, 59.3 (2C), 54.9, 54.3, 43.6, 43.5, 32.2, 32.1, 20.0 (2C); **^{19}F NMR** ($CDCl_3$, 376.5 MHz): δ –138.4 (ddt, *J* = 24.7, 6.0, 5.4 Hz, 2F), –149.2 (t, *J* = 20.9 Hz, 1F), –160.9 (ddt, *J* = 25.8, 20.5, 5.7 Hz, 2F); **FTIR** (thin film): cm^{-1} 2974, 2937, 1740, 1652, 1599, 1581, 1523, 1496, 1329, 1224, 1097, 997; **HRMS** (ESI$^+$, m/z): Calcd for $C_{28}H_{33}F_5NO_5^+$ ([M+H]$^+$): 558.2273; found: 558.2272.

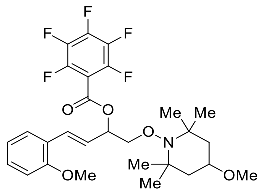

6h

(*E*)-1-((4-Methoxy-2,2,6,6-tetramethylpiperidin-1-yl)oxy)-4-(2-methoxyphenyl)but-3-en-2-yl 2,3,4,5,6-pentafluorobenzoate (6h). Synthesized using standard conditions. Isolated by flash column chromatography (silica gel, 100% hexanes to 5% ethyl acetate–hexanes) as a colorless oil (43.5 mg, 78%). R_f = 0.69 (25% EtOAc–hexanes); **^{1}H NMR** ($CDCl_3$, 400 MHz): δ 7.49 (d, *J* = 7.4 Hz, 1H), 7.68 (t, *J* = 7.7 Hz, 1H), 7.17 (d, *J* = 16.0 Hz, 1H), 7.00 (t, *J* =7.3 Hz, 1H), 6.95 (d, *J* = 8.1 Hz, 1H), 6.37 (dd, *J* = 16.1, 7.2 Hz, 1H), 6.03–5.97 (m, 1H), 4.19 (dd, *J* = 9.7, 7.4 Hz, 1H), 4.12 (dd, *J* = 10.4, 3.2 Hz, 1H), 3.93 (s, 3H), 3.56–3.46 (m, 1H), 3.40 (s, 3H), 1.98–1.89 (m, 2H), 1.45 (t, *J* = 11.9 Hz, 2H), 1.31 (s, 3H), 1.29 (s, 3H), 1.25 (s, 3H), 1.22 (s, 3H); **^{13}C{^{1}H} NMR** ($CDCl_3$, 100 MHz): δ 156.9, 155.7, 143.9 (d, $J_{C–F}$ = 254.3 Hz, 2C), 141.6 (d, $J_{C–F}$ = 259.6 Hz, 1C), 136.2 (d, $J_{C–F}$ = 253.6 Hz, 2C), 128.4, 128.1, 125.9, 123.3, 121.9, 119.2, 109.5, 107.2 (t, $J_{C–F}$ = 16.1 Hz, 1C), 76.2, 75.0, 70.2, 58.9, 58.8, 54.4, 54.1, 43.2, 43.1, 31.7, 31.6, 19.5 (2C); **^{19}F NMR** ($CDCl_3$, 376.5 MHz): δ –138.4 (ddt, *J* = 24.2, 5.6, 5.2 Hz, 2F), –149.5 (t, *J* = 20.7 Hz, 1F), –160.9 (ddt, *J* = 26.4, 20.5, 6.0 Hz, 2F); **FTIR** (thin film): cm^{-1} 2974, 2937, 1737, 1652, 1599, 1523, 1493, 1465, 1329, 1222, 1096, 996, 751; **HRMS** (ESI$^+$, m/z): Calcd for $C_{28}H_{33}F_5NO_5^+$ ([M+H]$^+$): 558.2273; found: 558.2277.

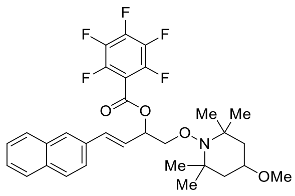

6i

(*E*)-1-((4-Methoxy-2,2,6,6-tetramethylpiperidin-1-yl)oxy)-4-(naphthalen-2-yl)but-3-en-2-yl 2,3,4,5,6-pentafluorobenzoate (6i). Synthesized using standard conditions. Isolated by flash column chromatography (silica gel, 100% hexanes to 20% ethyl acetate–hexanes) as a colorless oil (39.4 mg, 68%). ***R_f*** = 0.69 (25% EtOAc–hexanes); **^{1}H NMR** ($CDCl_3$, 400 MHz): δ 7.85–7.76 (m, 4H), 7.60 (d, *J* = 8.6 Hz, 1H), 7.51–7.44 (m, 2H), 6.96 (d, *J* = 15.9 Hz, 1H), 6.37 (dd, *J* = 16.0, 7.3 Hz, 1H), 5.97 (td, *J* = 7.3, 3.3 Hz, 1H), 4.15 (dd, *J* = 10.2, 7.4 Hz, 1H), 4.09 (dd, *J* = 10.5, 3.3 Hz, 1H), 3.46 (tt, *J* = 11.4, 4.0 Hz, 1H), 3.33 (s, 3H), 1.92–1.83 (m, 2H), 1.39 (t, *J* = 12.1 Hz, 2H), 1.26 (s, 3H), 1.23 (s, 3H), 1.20 (s, 3H), 1.16 (s, 3H); **^{13}C{^{1}H} NMR** ($CDCl_3$, 100 MHz): δ 158.2, 145.3 (d, $J_{C–F}$ = 258.2 Hz, 2C), 143.0 (d, $J_{C–F}$ = 258.6 Hz, 1C), 137.5 (d, $J_{C–F}$ = 255.9 Hz, 2C), 134.6, 133.3, 133.1, 133.1, 128.3, 128.0, 127.6, 127.2, 126.3, 126.2, 123.1, 122.9, 108.2 (t, $J_{C–F}$ = 16.0 Hz, 1C), 75.8, 71.5, 60.2 (2C), 55.7, 44.4, 44.3, 33.0, 32.9, 20.8 (2C); **^{19}F NMR** ($CDCl_3$, 376.5 MHz): δ –138.4 (d, *J* = 20.4 Hz, 2F), –149.1 (t, *J* = 20.9 Hz, 1F), –160.9 (t, *J* = 18.2 Hz, 2F); **FTIR** (thin film): cm^{-1} 2975, 2937, 1737, 1652, 1523, 1496, 1324.51, 1221, 1096, 996, 734; **HRMS** (ESI$^+$, m/z): Calcd for $C_{31}H_{33}F_5NO_4^+$ ([M+H]$^+$): 578.2324; found: 578.2326.

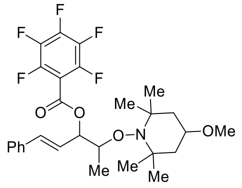

6k

(E)-4-((4-methoxy-2,2,6,6-tetramethylpiperidin-1-yl)oxy)-1-phenylpent-1-en-3-yl 2,3,4,5,6-pentafluorobenzoate (6k). Synthesized using standard conditions. A diastereomeric ratio of 1.1:1 was determined by ^{1}H NMR of the crude reaction. Isolated by flash column chromatography (silica gel, 100% hexanes to 5% ethyl acetate–hexanes) as a colorless oil (31.0 mg, 57%) as a 1:1.2 mixture of diastereomers. Careful purification attempts allowed for the isolation of small amounts of each diastereomer from the whole sample. The ^{1}H NMR spectra of the full sample is provided with an ^{1}H NMR of each diastereomer in section 8.

Diasteromer 1 – minor *R_f* = 0.77 (25% EtOAc–hexanes); **^{1}H NMR** ($CDCl_3$, 400 MHz): δ 7.41 (d, *J* = 7.6 Hz, 2H), 7.33 (t, *J* = 7.5 Hz, 2H), 7.30–7.26 (m, 1H), 6.76 (d, *J* = 16.0 Hz, 1H), 6.30 (dd, *J* = 16.0, 7.2 Hz, 1H), 5.82 (t, *J* = 6.2 Hz, 1H), 4.29 (p, *J* = 6.5 Hz, 1H), 3.44 (tt, *J* = 10.9, 4.1 Hz, 1H), 3.32 (s, 3H), 1.86 (t, *J* = 1.7 Hz, 2H), 1.46–1.35 (m, 2H), 1.30 (d, *J* = 6.5 Hz, 3H), 1.25 (s, 3H), 1.18 (s, 6H), 1.13 (s, 3H); **^{13}C{^{1}H} NMR** ($CDCl_3$, 100 MHz): δ 158.1, 145.5 (d, $J_{C–F}$ = 257.5 Hz, 2C), 142.9 (d, $J_{C–F}$ = 247.9 Hz, 1C), 137.7 (d, $J_{C–F}$ = 255.1 Hz, 2C), 136.1, 134.4, 128.6 (2C), 128.2, 126.6 (2C), 123.1, 108.3 (t, $J_{C–F}$ = 15.4 Hz, 1C), 78.9, 78.8, 71.6, 60.9, 59.6, 55.7, 45.2, 45.0, 34.3, 34.3, 21.3 (2C), 15.8; **^{19}F NMR** ($CDCl_3$, 376.5 MHz): δ –138.3 (m, 2F), –149.1 (t, *J* = 20.0 Hz, 1F), –160.9 (m, 2F); **FTIR** (thin film): cm^{-1} 2975, 2928, 1739, 1652, 1524, 1497, 1328, 1226, 1099, 1005; **HRMS** (ESI$^+$, m/z): Calcd for $C_{28}H_{33}F_5NO_4^+$ ([M+H]$^+$): 542.2324; found: 542.2322.

Diasteromer 2 – major *R_f* = 0.71 (25% EtOAc–hexanes); **^{1}H NMR** ($CDCl_3$, 400 MHz): δ 7.40 (d, *J* = 7.8 Hz, 2H), 7.32 (t, *J* = 7.5 Hz, 2H), 7.26 (t, *J* =7.1 Hz, 1H), 6.73 (d, *J* = 16.0 Hz, 1H), 6.20 (d, *J* = 16.0, 7.3 Hz, 1H), 5.93 (dd, *J* = 7.6, 2.7 Hz, 1H), 4.23 (qd, *J* = 6.6, 2.4 Hz, 1H), 3.44 (tt, *J* = 11.2, 4.1 Hz, 1H), 3.32 (s, 3H), 1.90–1.82 (m, 2H), 1.41 (t, *J* = 12.2 Hz, 2H), 1.32 (d, *J* = 6.6 Hz, 3H), 1.23 (s, 3H), 1.20 (s, 3H), 1.16 (s, 3H), 1.14 (s, 3H); **^{13}C{^{1}H} NMR** ($CDCl_3$, 100 MHz): δ 158.2, 145.3 (d, $J_{C–F}$ = 258.0 Hz, 2C), 143.1, (d, $J_{C–F}$ = 259.4 Hz, 1C), 137.6 (d, $J_{C–F}$ = 256.1 Hz, 2C), 136.3, 135.9, 128.6 (2C), 128.2, 126.6 (2C), 123.3, 108.4 (t, $J_{C–F}$ = 15.9 Hz, 1C), 79.6, 79.5, 71.6, 60.7, 60.0, 55.7, 45.1, 45.0, 34.5, 34.2, 21.3, 21.2, 14.5; **^{19}F NMR** ($CDCl_3$, 376.5 MHz): δ –138.6 (m, 2F), –149.3 (t, *J* = 21.0 Hz, 1F), –160.9 (m, 2F); **FTIR** (thin film): cm^{-1} 2976, 2931, 1740, 1652, 1524, 1497, 1328, 1226, 1098, 1004; **HRMS** (ESI$^+$, m/z): Calcd for $C_{28}H_{33}F_5NO_4^+$ ([M+H]$^+$): 542.2324; found: 542.2322.

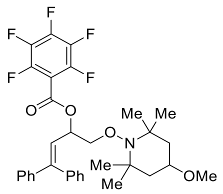

6o

1-((4-Methoxy-2,2,6,6-tetramethylpiperidin-1-yl)oxy)-4,4-diphenylbut-3-en-2-yl 2,3,4,5,6-pentafluorobenzoate (6o). Synthesized using standard conditions. Isolated by flash column chromatography (silica gel, 100% hexanes to 5% ethyl acetate–hexanes) as a colorless oil (43.7 mg, 72%). ***R_f*** = 0.76 (25% EtOAc–hexanes); **^{1}H NMR** ($CDCl_3$, 400 MHz): δ 7.41–7.31 (m, 3H), 7.28–7.19 (m, 7H), 6.09 (d, *J* = 9.0 Hz, 1H), 5.78–5.72 (m, 1H), 4.03 (dd, *J* = 10.3, 7.2 Hz, 1H), 3.92 (dd, *J* = 10.4, 3.4 Hz, 1H), 3.38 (tt, *J* = 11.6, 4.0 Hz, 1H), 3.28 (s, 3H), 1.84–1.75 (m, 2H), 1.32 (td, *J* = 12.2, 6.7 Hz, 2H), 1.17 (s, 3H), 1.09 (s, 3H), 1.07 (s, 3H), 1.03 (s, 3H); **^{13}C{^{1}H} NMR** ($CDCl_3$, 100 MHz): δ 157.6, 146.6, 144.9 (d, $J_{C–F}$ = 256.7 Hz, 2C), 142.7 (d, $J_{C–F}$ = 259.6 Hz, 1C), 140.5, 138.1, 137.3 (d, $J_{C–F}$ = 250.5 Hz, 2C), 129.0 (2C), 128.2 (2C), 127.9 (2C), 127.8, 127.6, 127.1 (2C), 121.8, 108.2 (t, $J_{C–F}$ = 15.8 Hz, 1C), 74.1, 71.3, 59.9, 59.8, 55.4, 44.2, 44.1, 32.7, 32.5, 20.6, 20.6; **^{19}F NMR** ($CDCl_3$, 376.5 MHz): δ –138.6 (ddt, *J* = 24.9, 6.4, 5.5 Hz, 2F), –149.5 (tt, *J* = 20.9, 4.4 Hz, 1F), –160.9 (t, *J* = 26.6, 20.2, 5.7 Hz, 2F); **FTIR** (thin film): cm^{-1} 2975, 2936, 1740, 1652, 1523, 1496, 1223, 1097, 996, 941, 764, 701; **HRMS** (ESI^+, m/z): Calcd for $C_{33}H_{35}F_5NO_4^+$ ($[M+H]^+$): 604.2481; found: 604.2483.

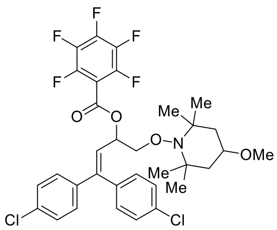

6p

4,4-Bis(4-chlorophenyl)-1-((4-methoxy-2,2,6,6-tetramethylpiperidin-1-yl)oxy)but-3-en-2-yl 2,3,4,5,6-pentafluorobenzoate (6p). Synthesized using standard conditions. Isolated by flash column chromatography (silica gel, 100% hexanes to 10% ethyl acetate–hexanes) as a colorless oil (51.7 mg, 77%). ***R_f*** = 0.74 (25% EtOAc–hexanes); **^{1}H NMR** ($CDCl_3$, 400 MHz): δ 7.39 (d, *J* = 8.2 Hz, 2H), 7.25 (d, *J* = 8.2 Hz, 2H), 7.20 (d, *J* = 8.3 Hz, 2H), 7.14 (d, *J* = 8.3 Hz, 2H), 6.08 (d, *J* = 9.1 Hz, 1H), 5.73–5.66 (m, 1H), 4.03 (dd, *J* = 10.1, 7.4 Hz, 1H), 3.92 (dd, *J* = 10.0, 3.0 Hz, 1H), 3.40 (tt, *J* = 11.4, 4.2 Hz, 1H), 3.30 (s, 3H), 1.86–1.79 (m, 2H), 1.34 (td, *J* = 11.9, 5.6 Hz, 2H), 1.17 (s, 3H), 1.11 (s, 3H), 1.10 (s, 3H), 1.06 (s, 3H); **^{13}C{^{1}H} NMR** ($CDCl_3$, 100 MHz): δ 158.0, 145.3 (d, $J_{C–F}$ = 258.0 Hz, 2C), 144.6, 143.1 (d, $J_{C–F}$ = 259.4 Hz, 1C), 138.9, 137.5 (d, $J_{C–F}$ = 251.8 Hz, 2C), 136.4, 134.3, 134.2, 130.7 (2C), 128.9 (2c), 128.6 (2C), 128.5 (2C), 123.2, 108.1 (t, $J_{C–F}$ = 15.8 Hz, 1C), 77.1, 73.9, 71.5, 60.2, 60.1, 55.8, 44.5, 44.4, 33.0, 32.8, 20.9, 20.9; **^{19}F NMR** ($CDCl_3$, 376.5 MHz): δ –138.5 (ddt, *J* = 25.3, 5.9, 5.5 Hz, 2F), –149.0 (tt, *J* = 21.0, 4.6 Hz, 1F), –160.7 (t, *J* = 26.4, 20.2, 6.0 Hz, 2F); **FTIR** (thin film): cm^{-1} 2976, 2939, 1739, 1652, 1523, 1494, 1338, 1322, 1222, 1091, 996, 827, 730; **HRMS** (ESI^+, m/z): Calcd for $C_{33}H_{33}Cl_2F_5NO_4^+$ ($[M+H]^+$): 672.1701; found: 672.1710.

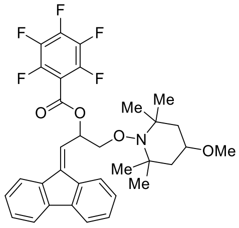

6q

1-(9H-Fluoren-9-ylidene)-3-((4-methoxy-2,2,6,6-tetramethylpiperidin-1-yl)oxy)propan-2-yl 2,3,4,5,6-pentafluorobenzoate (6q). Synthesized using standard conditions. Isolated by flash column chromatography (silica gel, 100% hexanes to 15% ethyl acetate–hexanes) as a white solid (31.4 mg, 52%). ***R**$_f$* = 0.66 (25% EtOAc–hexanes); **^{1}H NMR** ($CDCl_3$, 400 MHz): δ 7.90 (d, *J* = 7.9 Hz, 1H), 7.73 (d, *J* = 7.3 Hz, 1H), 7.67 (t, *J* = 8.1 Hz, 2H), 7.42 (t, *J* = 7.5 Hz, 1H), 7.39–7.33 (m, 2H), 7.29 (t, *J* = 7.5 Hz, 1H), 6.89 (td, *J* = 8.0, 3.3 Hz, 1H), 6.60 (d, *J* = 8.5 Hz, 1H), 4.27 (dd, *J* = 10.4, 7.5 Hz, 1H), 4.19 (dd, *J* = 10.5, 3.4 Hz, 1H), 3.44 (tt, *J* = 11.3, 4.1 Hz, 1H), 3.32 (s, 3H), 1.89–1.80 (m, 2H), 1.37 (t, *J* = 12.1 Hz, 2H), 1.25 (s, 3H), 1.19 (s, 6H), 1.15 (s, 3H); **^{13}C{^{1}H} NMR** ($CDCl_3$, 100 MHz): δ 158.4, 145.4 (d, $J_{C–F}$ = 258.5 Hz, 2C), 143.2 (d, $J_{C–F}$ = 259.9 Hz, 1C), 141.5, 139.5, 138.6, 137.7 (d, $J_{C–F}$ = 256.1 Hz, 2C), 135.7, 129.1, 128.9, 127.4, 127.2, 125.5, 121.1, 120.4, 120.1, 119.6, 108.1 (t, $J_{C–F}$ = 15.4 Hz, 1C), 76.9, 72.5, 71.6, 60.4, 60.1, 55.8, 44.5, 44.4, 33.2, 32.9, 20.9, 20.9; **^{19}F NMR** ($CDCl_3$, 376.5 MHz): δ –138.0 (ddt, *J* = 25.1, 6.1, 5.7 Hz, 2F), –148.8 (tt, *J* = 21.0, 4.7 Hz, 1F), –160.6 (ddt, *J* = 26.6, 20.2, 5.8 Hz, 2F); **FTIR** (thin film): cm^{-1} 2975, 2931, 1740, 1652, 1523, 1497, 1450, 1325, 1220, 1096, 997, 728; **HRMS** (ESI^+, m/z): Calcd for $C_{33}H_{33}F_5NO_4^+$ ($[M+H]^+$): 602.2324; found: 602.2328.

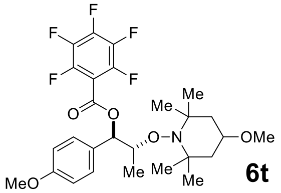
6t

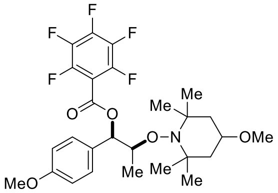
6t'

2-((4-Methoxy-2,2,6,6-tetramethylpiperidin-1-yl)oxy)-1-(4-methoxyphenyl)propyl 2,3,4,5,6-pentafluorobenzoate (6t). Synthesized using standard conditions. A 1:1 mixture of diastereomers by crude ^{1}H NMR. Isolated by flash column chromatography (silica gel,100% hexanes to 10% ethyl acetates–hexanes) (32.2 mg, 59%).

Diasteromer 1 (6t). Isolated as a white solid (15.4 mg). ***R**$_f$* = 0.64 (25% EtOAc–hexanes); **^{1}H NMR** ($CDCl_3$, 400 MHz): δ 7.32 (d, *J* = 8.7 Hz, 2H), 6.89 (d, *J* = 8.7 Hz, 2H), 5.98 (d, *J* = 6.9 Hz, 1H), 4.40 (p, *J* = 6.6 Hz, 1H), 3.81 (s, 3H), 3.42 (tt, *J* = 11.2, 4.1 Hz, 1H), 3.31 (s, 3H), 1.86 (dt, *J* = 12.6, 3.8 Hz, 1H), 1.79 (dt, *J* = 12.6, 3.7 Hz, 1H), 1.39 (t, *J* = 11.9 Hz, 1H), 1.33 (t, *J* = 11.9 Hz, 1H), 1.21 (s, 3H), 1.13–1.06 (m, 12H); **^{13}C{^{1}H} NMR** ($CDCl_3$, 100 MHz): δ 159.6, 158.0, 145.6 (d, $J_{C–F}$ = 253.8 Hz, 2C), 143.1 (d, *J* = 246.1 Hz, 1C), 137.7 (d, *J* = 249.5 Hz, 2C), 128.9 (2C), 128.7, 108.3 (t, $J_{C–F}$ = 15.6 Hz, 1C), 113.7 (2C), 80.6, 79.3, 71.7, 61.1, 59.4, 55.7, 55.2, 45.3, 45.0, 34.4, 34.2, 21.4, 21.3, 16.1; **^{19}F NMR** ($CDCl_3$, 376.5 MHz): δ –138.1 (ddt, *J* = 25.8, 6.4, 5.8 Hz, 2F), –149.1 (t, *J* = 21.0 Hz, 1F), –160.9 (ddt, *J* = 25.8, 20.3, 5.7 Hz, 2F); **FTIR** (thin film): cm^{-1} 2937, 1736, 1652, 1613, 1496, 1326, 1228, 1177, 1097, 1005, 732; **HRMS** (ESI^+, m/z): Calcd for $C_{27}H_{33}F_5NO_5^+$ ($[M+H]^+$): 546.2273; found: 546.2272.

Diasteromer 2 (6t'). Isolated as a colorless oil (16.8 mg). ***R**$_f$* = 0.65 (25% EtOAc–hexanes); **^{1}H NMR** ($CDCl_3$, 400 MHz): δ 7.30 (d, *J* = 8.7 Hz, 2H), 6.89 (d, *J* = 8.7 Hz, 2H), 6.20 (d, *J* = 3.4 Hz, 1H), 4.26 (qd, *J* = 6.7, 3.2 Hz, 1H), 3.80 (s, 3H), 3.43 (tt, *J* = 11.3, 4.1 Hz, 1H), 3.32 (s, 3H), 1.88–1.79 (m, 2H), 1.39 (q, *J* = 13.1 Hz, 2H), 1.23 (d, *J* = 6.6 Hz, 3H), 1.20 (s, 3H), 1.12 (s, 3H), 1.10 (s, 3H), 1.08 (s, 3H); **^{13}C{^{1}H} NMR** ($CDCl_3$, 100 MHz): δ 159.3, 158.1, 145.5 (d, $J_{C–F}$ = 258.6 Hz, 2C), 143.1 (d, $J_{C–F}$ = 233.6 Hz, 1C), 137.7 (d, $J_{C–F}$ = 255.8 Hz, 2C), 129.1, 128.0 (2C), 113.7 (2C), 108.3 (t, $J_{C–F}$ = 15.0 Hz, 1C), 80.6, 79.9, 71.7, 60.7, 60.0, 55.7, 55.2, 45.1 (2C), 34.5, 34.3, 21.4, 21.3, 14.0; **^{19}F NMR** ($CDCl_3$, 376.5 MHz): δ –138.3 (ddt, *J* = 24.8, 5.7, 5.4 Hz, 2F), –149.1 (tt, *J* = 21.1, 4.4 Hz, 1F), –160.7 (ddt, *J* = 26.1, 20.1, 5.8 Hz, 2F); **FTIR** (thin film): cm^{-1} 2978, 2938, 1741, 1652, 1613, 1497, 1327, 1226, 1177, 1096, 1004, 731; **HRMS** (ESI^+, m/z): Calcd for $C_{27}H_{33}F_5NO_5^+$ ($[M+H]^+$): 546.2273; found: 546.2275.

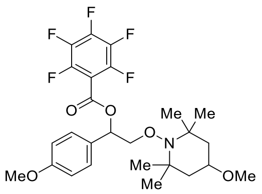

6u

2-((4-Methoxy-2,2,6,6-tetramethylpiperidin-1-yl)oxy)-1-(4-methoxyphenyl)ethyl 2,3,4,5,6-pentafluorobenzoate (6u). Synthesized using standard conditions. Isolated by flash column chromatography (silica gel, 100% hexanes to 20% ethyl acetate–hexanes) as a white solid (32.0 mg, 60%). ***R**f* = 0.68 (25% EtOAc–hexanes); **^{1}H NMR** ($CDCl_3$, 400 MHz): δ 7.34 (d, *J* = 8.7 Hz, 2H), 6.89 (d, *J* = 8.7 Hz, 2H), 6.18 (dd, *J* = 8.3, 3.4 Hz, 1H), 4.20 (dd, *J* = 10.4, 8.2 Hz, 1H), 4.00 (dd, *J* = 10.4, 3.5 Hz, 1H), 3.80 (s, 3H), 3.41 (tt, *J* = 11.3, 4.1 Hz, 1H), 3.31 (s, 3H), 1.88–1.79 (m, 2H), 1.34 (t, *J* = 12.1 Hz, 2H), 1.16 (s, 3H), 1.15 (s, 3H), 1.14 (s, 3H), 1.09 (s, 3H); **^{13}C{^{1}H} NMR** ($CDCl_3$, 100 MHz): δ 159.7, 158.2, 145.4 (d, $J_{C–F}$ = 258.0 Hz, 2C), 143.1 (d, $J_{C–F}$ = 259.9 Hz, 1C), 137.6 (d, $J_{C–F}$ = 255.1 Hz, 2C), 128.4, 128.3 (2C), 113.9 (2C), 108.2 (t, $J_{C–F}$ = 15.4 Hz, 1C), 78.6, 76.6, 71.6, 60.2, 60.2, 55.7, 55.2, 44.5, 44.4, 33.1, 32.9, 20.9 (2C); **^{19}F NMR** ($CDCl_3$, 376.5 MHz): δ –138.3 (ddt, *J* = 25.0, 6.2, 5.6 Hz, 2F), –149.2 (tt, *J* = 21.0, 4.7 Hz, 1F), –160.9 (ddt, *J* = 26.8, 20.2, 5.7 Hz, 2F); **FTIR** (thin film): cm^{-1} 2964, 2937, 1739, 1652, 1614, 1496, 1328, 1223, 1176, 1096, 1032, 996, 944, 831; **HRMS** (ESI^+, m/z): Calcd for $C_{26}H_{31}F_5NO_5^+$ ($[M+H]^+$): 532.2117; found: 532.2117.

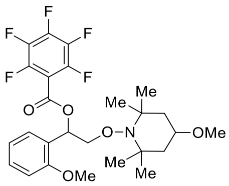

6v

2-((4-Methoxy-2,2,6,6-tetramethylpiperidin-1-yl)oxy)-1-(2-methoxyphenyl)ethyl 2,3,4,5,6-pentafluorobenzoate (6v). Synthesized using standard conditions. Isolated by flash column chromatography (silica gel, 100% hexanes to 20% ethyl acetate–hexanes) as a colorless oil (29.3 mg, 55%). ***R**f* = 0.62 (25% EtOAc–hexanes); **^{1}H NMR** ($CDCl_3$, 400 MHz): δ 7.37 (dd, *J* = 7.6, 1.7 Hz, 1H), 7.29 (td, *J* = 8.0, 1.7 Hz, 1H), 6.96 (t, *J* = 7.5 Hz, 1H), 6.89 (d, *J* = 8.2 Hz, 1H), 6.69 (dd, *J* = 8.0, 2.8 Hz, 1H), 4.08 (dd, *J* = 10.2, 8.0 Hz, 1H), 4.05 (dd, *J* = 10.5, 2.8 Hz, 1H), 3.86 (s, 3H), 3.42 (tt, *J* = 11.3, 3.7 Hz, 1H), 3.31 (s, 3H), 1.89–1.78 (m, 2H), 1.34 (td, *J* = 11.9, 7.7 Hz, 2H), 1.25 (s, 3H), 1.18 (s, 3H), 1.11 (s, 3H), 1.07 (s, 3H); **^{13}C{^{1}H} NMR** ($CDCl_3$, 100 MHz): δ 158.0, 155.9, 145.4 (d, $J_{C–F}$ = 254.2 Hz, 2C), 143.0, (d, $J_{C–F}$ = 259.2 Hz, 1C), 137.6 (d, $J_{C–F}$ = 255.6 Hz, 2C), 129.3, 126.6, 124.5, 120.4, 110.3, 108.4 (t, $J_{C–F}$ = 15.8 Hz, 1C), 77.7, 72.3, 71.6, 60.0, 60.0, 55.7, 55.3, 44.5, 44.4, 32.9, 32.8, 20.8, 20.8; **^{19}F NMR** ($CDCl_3$, 376.5 MHz): δ –138.3 (m, 2F), –149.5 (t, *J* = 21.0 Hz, 1F), –160.9 (m, 2F); **FTIR** (thin film): cm^{-1} 2975, 2937, 1742, 1652, 1523, 1495, 1223, 1097, 1033, 996, 945, 754; **HRMS** (ESI^+, m/z): Calcd for $C_{26}H_{31}F_5NO_5^+$ ($[M+H]^+$): 532.2117; found: 532.2114.

5. Competition Experiments

1-Phenyl-1,3-butadiene -vs- Styrene

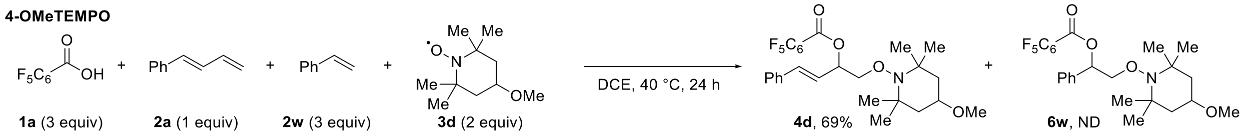

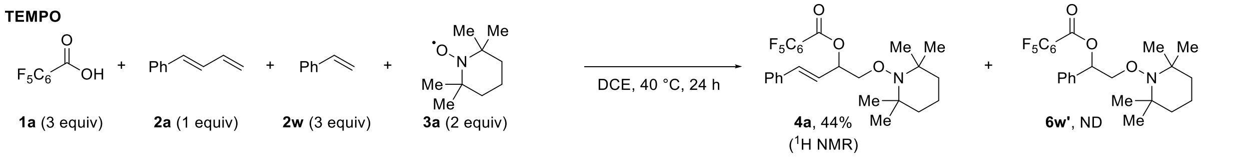

Using 4-OMeTEMPO. To a 1-dram vial equipped with a Teflon-coated stir bar was added the 1-phenyl-1,3-butadiene **2a** (13.0 mg, 0.1 mmol, 1.0 equiv). 1,2-Dichloroethane (0.33 mL) was added, followed by sequential addition of pentafluorobenzoic acid **1a** (63.6 mg, 0.3 mmol, 3.0 equiv), 4-OMeTEMPO **3d** (37.3 mg, 0.2 mmol, 2.0 equiv), and styrene **2w** (34.5 μL, 0.3 mmol, 3.0 equiv). The vial was capped and stirred at 40 °C in an aluminum heating block for 24 h. The resulting crude mixture was filtered through activated, neutral Al_2O_3 (Brockman Grade I, 58–60 Å mesh powder) and concentrated *in vacuo*. Purification by column chromatography (silica gel, 100% hexanes to 15% ethyl acetate–hexanes) afforded **4d** as a colorless oil (36.2 mg, 69%). Product **4w** was not detected from the crude ^{1}H NMR.

Using TEMPO. To a 1-dram vial equipped with a Teflon-coated stir bar was added the 1-phenyl-1,3-butadiene **2a** (13.0 mg, 0.1 mmol, 1.0 equiv). 1,2-Dichloroethane (0.33 mL) was added, followed by sequential addition of pentafluorobenzoic acid **1a** (63.6 mg, 0.3 mmol, 3.0 equiv), TEMPO **3a** (31.3 mg, 0.2 mmol, 2.0 equiv), and styrene **2w** (34.5 μL, 0.3 mmol, 3.0 equiv). The vial was capped and stirred at 40 °C in an aluminum heating block for 23.5 h. The resulting crude mixture was filtered through activated, neutral Al_2O_3 (Brockman Grade I, 58–60 Å mesh powder) and concentrated *in vacuo*. The yield of **4a** was determined by ^{1}H NMR of the crude reaction with dibromomethane (7.0 μL, 0.1 mmol) as an internal standard. Product **4w'** was not detected from the crude ^{1}H NMR.

1-Phenyl-1,3-butadiene -vs- 4-Methoxystyrene

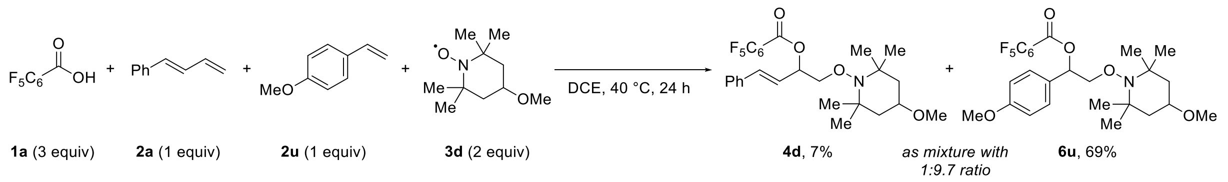

To a 1-dram vial equipped with a Teflon-coated stir bar was added the 1-phenyl-1,3-butadiene **2a** (13.0 mg, 0.1 mmol, 1.0 equiv). 1,2-Dichloroethane (0.33 mL) was added, followed by sequential addition of pentafluorobenzoic acid **1a** (63.6 mg, 0.3 mmol, 3.0 equiv), 4-OMeTEMPO **3d** (37.3 mg, 0.2 mmol, 2.0 equiv), and 4-methoxystyrene **2w** (39.9 μL, 0.3 mmol, 3.0 equiv). The vial was capped and stirred at 40 °C in an aluminum heating block for 24 h. The resulting crude mixture was filtered through activated, neutral Al_2O_3 (Brockman Grade I, 58–60 Å mesh powder) and concentrated *in vacuo*. Purification by column chromatography (silica gel, 100% hexanes to 15% ethyl acetate–hexanes) afforded a 1:9.7 mixture of **4d**:**6u** as a colorless oil (40.2 mg). (Analysis results in 7% **4d** and 69% **6u**).

1-(4-Methoxyphenyl)-1,3-butadiene -vs- 1-(4-(Trifluoromethyl)phenyl)-1,3-butadiene

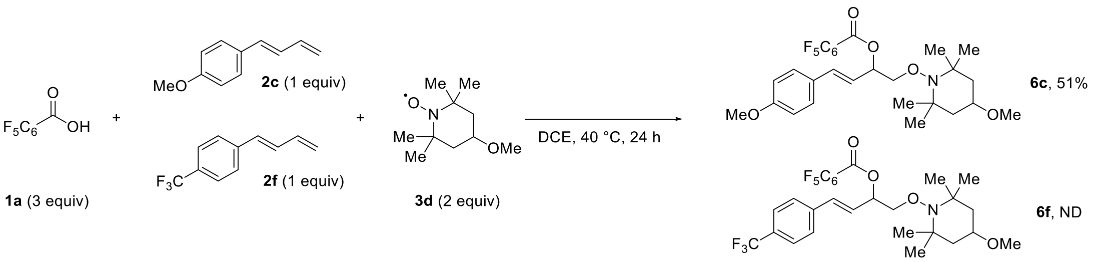

To a 1-dram vial equipped with a Teflon-coated stir bar was added the 1-(4-(trifluoromethyl)phenyl)-1,3-butadiene **2f** (19.8 mg, 0.1 mmol, 1.0 equiv). 1,2-Dichloroethane (0.33 mL) was added, followed by sequential addition of pentafluorobenzoic acid **1a** (63.6 mg, 0.3 mmol, 3.0 equiv), 4-OMeTEMPO **3d** (37.3 mg, 0.2 mmol, 2.0 equiv), and 1-(4-methoxyphenyl)-1,3-butadiene **2c** (16.0 mg, 0.1 mmol, 1.0 equiv). The vial was capped and stirred at 40 °C in an aluminum heating block for 24 h. The resulting crude mixture was filtered through activated, neutral Al_2O_3 (Brockman Grade I, 58–60 Å mesh powder) and concentrated *in vacuo*. Purification by column chromatography (silica gel, 100% hexanes to 5% ethyl acetate–hexanes) afforded **6c** as a colorless oil (28.6 mg, 51%).

6. Derivatization Experiments

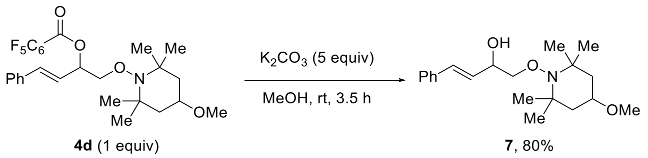

(*E*)-1-((4-Methoxy-2,2,6,6-tetramethylpiperidin-1-yl)oxy)-4-phenylbut-3-en-2-ol (7). To a stirring 1-dram vial containing a solution of **4d** (26.4 mg, 0.05 mmol, 1.0 equiv) in methanol (0.5 mL) was added potassium carbonate (34.6 mg, 0.25 mmol, 5.0 equiv). The solution was stirred at room temperature for 3.5 h. The crude reaction was filtered through a cotton plug and concentrated *in vacuo*. Purification by flash column chromatography (silica gel, 100% hexanes to 50% ethyl acetate–hexanes) afforded **7** as a white solid (13.3 mg, 80%). $\boldsymbol{R_f}$ = 0.40 (25% EtOAc–hexanes); **^{1}H NMR** ($CDCl_3$, 400 MHz): δ 7.35 (d, *J* = 7.6 Hz, 2H), 7.29 (t, *J* = 7.5 Hz, 2H), 7.22 (t, *J* = 7.5 Hz, 1H), 6.66 (d, *J* = 16.0 Hz, 1H), 6.17 (dd, *J* = 16.0, 6.1 Hz, 1H) 4.54–4.47 (m, 1H), 3.86 (dd, *J* = 9.2, 3.3 Hz, 1H), 3.84–3.78 (m, 1H), 3.41 (tt, *J* = 11.4, 4.3 Hz, 1H), 3.30 (s, 3H), 2.45 (s, 1H), 1.88–1.81 (m, 2H), 1.37 (t, *J* = 11.9 Hz, 2H), 1.23 (s, 3H), 1.21 (s, 3H), 1.16 (s, 6H); **^{13}C{^{1}H} NMR** ($CDCl_3$, 100 MHz): δ 136.6, 131.4, 128.5 (2C), 127.8, 127.7, 126.4 (2C), 80.2, 71.6, 71.3, 60.3, 60.2, 55.8, 44.6, 44.5, 33.3 (2C), 21.2, 21.1; **FTIR** (thin film): cm^{-1} 3426 (broad), 2974, 2936, 1494, 1451, 1375, 1361, 1096, 1046, 999, 996, 749, 693; **HRMS** (ESI$^+$, m/z): Calcd for $C_{20}H_{32}NO_3^+$ ([M+H]$^+$): 334.2377; found: 334.2375.

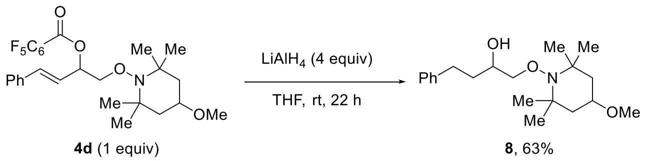

1-((4-Methoxy-2,2,6,6-tetramethylpiperidin-1-yl)oxy)-4-phenylbutan-2-ol (8). To a stirring 1-dram vial containing a suspension of $LiAlH_4$ (7.6 mg, 0.2 mmol, 4.0 equiv) in THF (0.33 mL) was added **4d** (26.4 mg, 0.05 mmol, 1.0 equiv). The solution was stirred at room temperature for 22 h. The reaction was quenched with addition of aqueous HCl (1.0 M, 1 mL). The crude reaction was extracted with EtOAc (3 mL x 3). The combined organic layers were washed with brine (10 mL), dried with Na_2SO_4, and concentrated *in vacuo*. Purification by flash column chromatography (silica gel, 100% hexanes to 50% ethyl acetate–hexanes)

afforded **10** as a clear oil (10.5 mg, 63%). ***R$_f$*** = 0.49 (25% EtOAc–hexanes); **^{1}H NMR** ($CDCl_3$, 400 MHz): δ 7.29 (t, *J* = 7.5 Hz, 2H), 7.22–7.16 (m, 3H), 3.86–3.71 (m, 3H), 3.43 (tt, *J* = 11.4, 4.2 Hz, 1H), 3.32 (s, 3H), 2.82 (ddd, *J* = 13.7, 9.7, 5.6 Hz, 1H), 2.69 (ddd, *J* = 13.7, 9.5, 6.9 Hz, 1H), 2.25 (br s, 1H), 1.89–1.82 (m, 2H), 1.38 (t, *J* = 12.0 Hz, 2H), 1.23 (s, 3H), 1.19 (s, 3H), 1.16 (s, 3H), 1.15 (s, 3H); **^{13}C{^{1}H} NMR** ($CDCl_3$, 100 MHz): δ 141.9, 128.4 (2C), 128.4 (2C), 125.8, 80.6, 71.6, 69.6, 60.2, 60.1, 55.8, 44.6, 44.5, 35.0, 33.3, 33.2, 31.8, 21.2, 21.0; **FTIR** (thin film): cm^{-1} 3433 (br), 2975, 2926, 1604, 1496, 1455, 1375, 1361, 1211, 1170, 1097, 1043, 699; **HRMS** (ESI$^+$, m/z): Calcd for $C_{20}H_{34}NO_3^+$ ([M+H]$^+$): 336.2533; found: 336.2531.

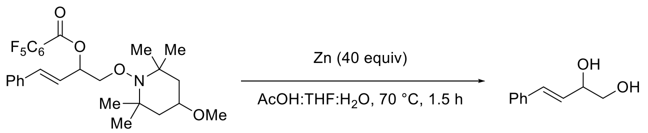

4d (1 equiv) **9**, 52%

(*E*)-4-Phenylbut-3-ene-1,2-diol (9). To a stirring solution of **4d** (26.4 mg, 0.05 mmol, 1.0 equiv) in THF (0.2 mL), DI H_2O (0.2 mL), and AcOH (0.6 mL) was added zinc dust (130.4 mg, 2.0 mmol, 40.0 equiv). The suspension was stirred at 70 °C in an aluminum heating block for 1.5 h. After removal from heat, the reaction was diluted with EtOAc (2 mL) and quenched with a saturated aqueous solution of $NaHCO_3$ (until pH ~7, ca. 10 mL). The layer were separated and the aqueous layer extracted with EtOAc (8 mL x 2). The combined organic layers were washed with a saturated aqueous solution of $NaHCO_3$ (10 mL) and brine (10 mL). The organic layer was dried with Na_2SO_4 and concentrated *in vacuo*. Purification by flash column chromatography (silica gel, 100% hexanes to 60% ethyl acetate–hexanes) afforded **9** as a white solid (4.3 mg, 52%). ***R$_f$*** = 0.08 (25% EtOAc–hexanes); **^{1}H NMR** ($CDCl_3$, 400 MHz): δ 7.39 (d, *J* = 6.8 Hz, 2H), 7.32 (t, *J* = 7.2 Hz, 2H), 7.26 (t, *J* = 7.2 Hz, 1H), 6.70 (dd, *J* = 16.1, 1.4 Hz, 1H), 6.21 (dd, *J* = 16.0, 6.3 Hz, 1H), 4.44 (dddd, *J* = 7.6, 6.3, 3.6, 1.3 Hz, 1H), 3.76 (dd, *J* = 11.1, 3.6 Hz, 1H), 3.61 (dd, *J* = 11.2, 7.4 Hz, 1H), 2.09 (br s, 2H); **^{13}C{^{1}H} NMR** ($CDCl_3$, 100 MHz): δ 136.2, 132.2, 128.6 (2C), 127.9, 127.6, 126.5 (2C), 73.2, 66.4; **FTIR** (thin film): cm^{-1} 3356 (br), 3027, 2926, 2872, 1494, 1449, 1325, 1071, 1030, 968, 748, 693; **HRMS** (ESI, m/z): Calcd for $C_{10}H_{12}O_2Na^+$ ([M+Na]$^+$): 187.0730; found: 187.0729.

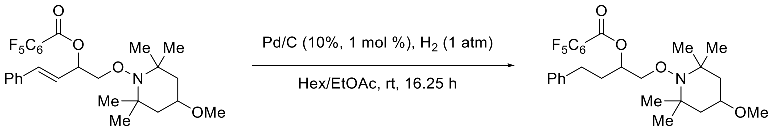

4d (1 equiv) **10**, 86%

1-((4-Methoxy-2,2,6,6-tetramethylpiperidin-1-yl)oxy)-4-phenylbutan-2-yl 2,3,4,5,6-pentafluoro benzoate (10). To a pear flask was added **4d** (26.4 mg, 0.05 mmol, 1.0 equiv). The flask was vacuum purged 3x, backfilling with nitrogen. Hexanes (0.3 mL), ethyl acetate (0.3 mL), and palladium on carbon (10 wt%, 0.6 mg, 0.0005 mmol, 0.01 equiv) were added. The flask was vacuum purged once more, backfilling with hydrogen gas. The reaction was stirred under an atmosphere of hydrogen for 16.25 h. The crude reaction was filtered through a plug of celite with EtOAc and concentrated *in vacuo*. Purification by flash column chromatography (silica gel, 100% hexanes to 20% ethyl acetate–hexanes) afforded **10** as a white solid (22.7 mg, 86%). ***R$_f$*** = 0.74 (25% EtOAc–hexanes); **^{1}H NMR** ($CDCl_3$, 400 MHz): δ 7.29 (t, *J* = 7.9 Hz, 2H), 7.22–7.17 (m, 3H), 5.40–5.33 (m, 1H), 4.00 (dd, *J* = 10.4, 6.3 Hz, 1H), 3.95 (dd, *J* = 10.3, 3.8 Hz, 1H), 3.42 (tt, *J* = 11.5, 4.1 Hz, 1H), 3.31 (s, 3H), 2.77 (ddd, *J* = 13.7, 9.9, 5.7 Hz, 1H), 2.70 (ddd, *J* = 13.7, 9.6, 6.5 Hz, 1H), 2.09 (dddd, *J* = 14.0, 9.5, 8.1, 5.8 Hz, 1H), 2.00 (dddd, *J* = 14.2, 9.8, 6.4, 4.5 Hz, 1H), 1.85 (dq, *J* = 12.6, 3.8 Hz, 2H), 1.36 (t, *J* = 12.0 Hz, 2H), 1.18 (s, 6H), 1.14 (s, 3H), 1.11 (s, 3H); **^{13}C{^{1}H} NMR** ($CDCl_3$, 100 MHz): δ 158.6, 145.2 (d, $J_{C\text{–}F}$ = 252.7 Hz, 2C), 143.0 (d, $J_{C\text{–}F}$ = 259.4 Hz, 1C), 140.8, 137.6 (d, $J_{C\text{–}F}$ = 255.6 Hz, 2C), 128.5, 128.3, 126.1, 108.5 (td, $J_{C\text{–}F}$ = 16.6, 4.0 Hz, 1C), 77.2, 75.1, 71.6, 60.2, 60.2, 55.8, 44.5, 44.4, 33.1, 33.0, 32.8, 31.5, 20.9, 20.9; **^{19}F NMR** ($CDCl_3$, 376.5 MHz): δ –138.7 (ddt, *J* = 24.9, 6.4, 5.1 Hz, 2F), –149.5 (tt, *J* = 20.8, 4.4 Hz, 1F), –160.8 (ddt, *J* = 26.7, 20.5, 5.8 Hz, 2F); **FTIR** (thin film): cm^{-1} 2975, 2937, 1740, 1653, 1523, 1497, 1324, 1228, 1097, 996; **HRMS** (ESI, m/z): Calcd for $C_{27}H_{32}F_5NO_4Na^+$ ([M+Na]$^+$): 552.2144; found: 552.2142.

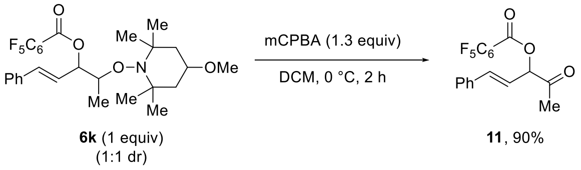

(*E*)-4-Oxo-1-phenylpent-1-en-3-yl 2,3,4,5,6-pentafluorobenzoate (11). To a stirring solution of **6k** (27.1 mg, 0.05 mmol, 1.0 equiv, 1:1 dr) in DCM at 0 °C was added mCPBA (70%, 16.0 mg, 0.065 mmol, 1.3 equiv). The solution was stirred at 0 °C for 2 h, upon which an aqueous saturated solution of sodium thiosulfate (1 mL) was added. The mixture was stirred at room temperature for 1 h. The layers were separated and the aqueous layer extracted with DCM (2 mL x 2). The combined organic layers were dried with Na_2SO_4 and concentrated *in vacuo*. Purification by flash column chromatography (silica gel, 100% hexanes to 10% ethyl acetate–hexanes) afforded **11** as a white solid (16.6 mg, 90%). R_f = 0.67 (25% EtOAc–hexanes); **^{1}H NMR** ($CDCl_3$, 400 MHz): δ 7.43 (d, *J* = 7.1 Hz, 2H), 7.39–7.30 (m, 3H), 6.92 (d, *J* = 15.9 Hz, 1H), 6.23 (dd, *J* = 15.9, 7.8 Hz, 1H), 5.89 (d, *J* = 7.8 Hz, 1H), 2.31 (s, 3H); **^{13}C{^{1}H} NMR** ($CDCl_3$, 100 MHz): δ 200.6, 158.2, 145.9 (d, $J_{C–F}$ = 265.2 Hz, 2C), 143.6 (d, $J_{C–F}$ = 260.5 Hz, 1C), 137.8 (d, $J_{C–F}$ = 251.3 Hz, 2C), 137.3, 135.1, 129.0, 128.8 (2C), 126.9 (2C), 119.1, 107.1 (t, $J_{C–F}$ = 14.6 Hz, 1C), 81.7, 26.1; **^{19}F NMR** ($CDCl_3$, 376.5 MHz): δ –137.3 (ddt, *J* = 25.2, 5.8, 5.7, 2F), –147.8 (tt, *J* = 20.7, 4.9 Hz, 1F), –160.4 (ddt, *J* = 26.1, 19.8, 6.0 Hz, 2F); **FTIR** (thin film): cm^{-1} 2925, 2850, 1731 (br), 1652, 1525, 1496, 1331, 1220, 1002, 968, 746, 693; **HRMS** (ESI$^+$, m/z): Calcd for $C_{18}H_{11}F_5O_3Na^+$ ([M+Na]$^+$): 393.0521; found: 393.0523.

Failed Conditions for Zn Reduction of OTMP.[a]

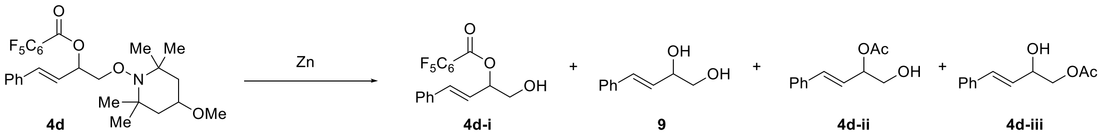

entry	Zn (equiv)	Solvent	ratio	[M]	Temp (°C)	Time (h)	Result
1	10	AcOH:THF:H_2O	1:100:100	0.001	rt	3.5	rsm (**4d**)
2	30	MeOH:aq (sat) NH_4Cl	1:1	0.05	60	19	rsm (**4d**)
3	10	AcOH:THF:H_2O	1:1:1	0.033	rt	22	rsm (**4d**)
4	80	AcOH:THF:H_2O	3:1:1	0.05	50	52	6.7 : 4.1 : 1 (**9** : **4d-iii** : **4d-ii**)
5	10	AcOH:THF:H_2O	1:1:1	0.067	rt	72	rsm (**4d**)
6	40	AcOH:THF:H_2O	3:1:1	0.05	70	2	65%[b] **9**, 16%[b] **4d-ii**, 8%[b] **4d-iii**
7	20	AcOH:THF:H_2O	2:1:1	0.05	40	24	rsm (**4d**)
8	40	AcOH:THF:H_2O	2:1:1	0.05	60	4.5	49%[b] **9**, 14%[b] **4d-ii**, 14%[b] **4d-iii**
9	40	AcOH:THF:H_2O	3:1:1	0.05	70	2	52%[b] **9**, 12%[b] **4d-ii**, 12%[b] **4d-iii**
10	40	AcOH:THF	1:1	0.05	70	72	rsm (**4d**)
11	40	AcOH:THF:*i*-PrOH	3:1:1	0.05	70	29	13%[c] **4d**, 3%[c] **4d-ii**, 13%[c] **4d-iii**
12	40[d]	AcOH:THF	3:1	0.063	70	72	rsm (**4d**)

[a]Reactions run on a 0.05 mmol scale. [b]Isolated yields. [c]Yields from quantitative ^{1}H NMR with dibromomethane as an internal standard. [d]Zn was pre-activated with TMSCl (3.2 equiv, 0.33 M in Et_2O) prior to introduction in the reaction.

7. Controls and Mechanistic Experiments

7.1 Radical trapping reactions without pentafluorobenzoic acid present

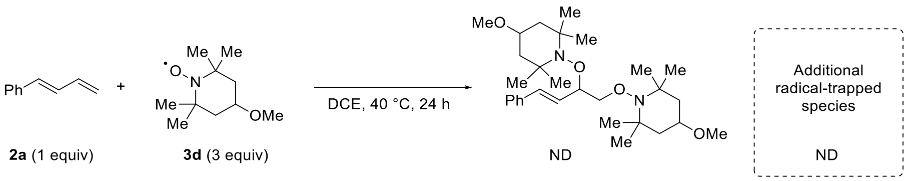

To a 1-dram vial equipped with a Teflon-coated stir bar was added the 1-phenyl-1,3-butadiene **2a** (13.0 mg, 0.1 mmol, 1.0 equiv). 1,2-Dichloroethane (0.33 mL) was added, followed by addition of 4-OMeTEMPO **3d** (55.9 mg, 0.3 mmol, 3.0 equiv). The vial was capped and stirred at 40 °C in an aluminum heating block for 24 h. The resulting crude mixture was filtered through activated, neutral Al_2O_3 (Brockman Grade I, 58–60 Å mesh powder) and concentrated *in vacuo*. Neither the di-TEMPO addition adduct or additional radical-trapped species were observed by either ^{1}H NMR.

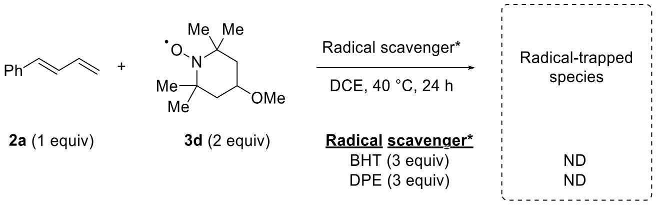

To a 1-dram vial equipped with a Teflon-coated stir bar was added the 1-phenyl-1,3-butadiene **2a** (13.0 mg, 0.1 mmol, 1.0 equiv). 1,2-Dichloroethane (0.33 mL) was added, followed by sequential addition of 4-OMeTEMPO **3d** (37.3 mg, 0.2 mmol, 2.0 equiv) and the radical scavenger (0.3 mmol, 3.0 equiv). The vial was capped and stirred at 40 °C in an aluminum heating block for 24 h. The resulting crude mixture was filtered through activated, neutral Al_2O_3 (Brockman Grade I, 58–60 Å mesh powder) and concentrated *in vacuo*. No radical-trapped species were observed by either ^{1}H NMR or GCMS.

7.2 Use of sodium pentafluorobenzoate instead of pentafluorobenzoic acid

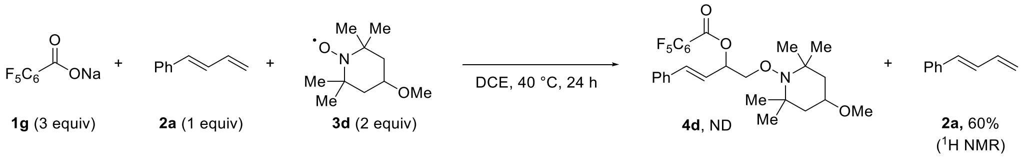

To a 1-dram vial equipped with a Teflon-coated stir bar was added the 1-phenyl-1,3-butadiene **2a** (13.0 mg, 0.1 mmol, 1.0 equiv). 1,2-Dichloroethane (0.33 mL) was added, followed by sequential addition of sodium pentafluorobenzoate **1g** (70.2 mg, 0.3 mmol, 3.0 equiv) and 4-OMeTEMPO **3d** (37.3 mg, 0.2 mmol, 2.0 equiv). The vial was capped and stirred at 40 °C in an aluminum heating block for 24 h. The resulting crude mixture was filtered through activated, neutral Al_2O_3 (Brockman Grade I, 58–60 Å mesh powder) and concentrated *in vacuo*. From the crude ^{1}H NMR with dibromomethane (7.0 µL, 0.1 mmol) as an internal standard, product **4d** was not detected and **2a** was recovered in 60% yield.

7.3 Use of oxoammonium salt instead of TEMPO radical

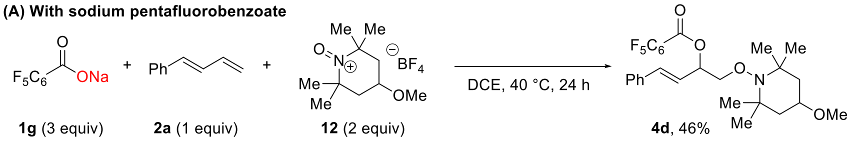

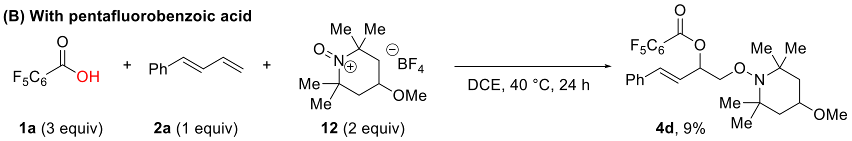

<u>Reaction A</u>. To a 1-dram vial equipped with a Teflon-coated stir bar was added the 1-phenyl-1,3-butadiene **2a** (13.0 mg, 0.1 mmol, 1.0 equiv). 1,2-Dichloroethane (0.33 mL) was added, followed by sequential addition of sodium pentafluorobenzoate **1g** (70.2 mg, 0.3 mmol, 3.0 equiv) and 4-OMeTEMPO oxoammonium salt **12** (54.6 mg, 0.2 mmol, 2.0 equiv). The vial was capped and stirred at 40 °C in an aluminum heating block for 24 h. The resulting crude mixture was filtered through activated, neutral Al_2O_3 (Brockman Grade I, 58–60 Å mesh powder) and concentrated *in vacuo*. Purification by column chromatography (silica gel, 100% hexanes to 20% ethyl acetate–hexanes) afforded **4d** as a colorless oil (24.6 mg, 46%).

<u>Reaction B</u>. Reaction was conducted as above, with pentafluorobenzoic acid (63.6 mg, 0.3 mmol, 3.0 equiv) instead of sodium pentafluorobenzoate. Purification by column chromatography (silica gel, 100% hexanes to 20% ethyl acetate–hexanes) afforded **4d** as a colorless oil (4.7 mg, 9%).

7.4 Addition of TBAI to reaction conditions.

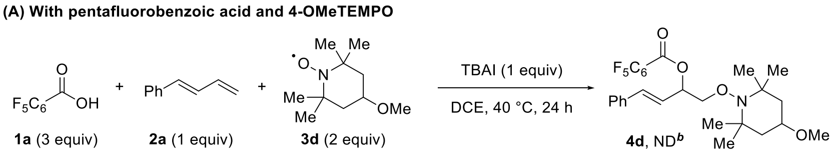

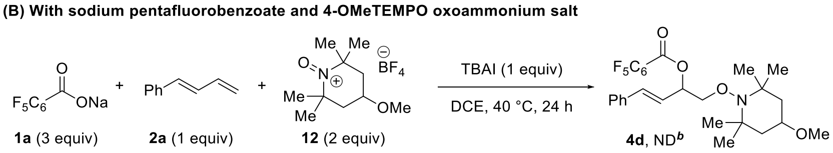

<u>Reaction A</u>. To a 1-dram vial equipped with a Teflon-coated stir bar was added the 1-phenyl-1,3-butadiene **2a** (13.0 mg, 0.1 mmol, 1.0 equiv). 1,2-Dichloroethane (0.33 mL) was added, followed by sequential addition of pentafluorobenzoic acid **1a** (63.6 mg, 0.3 mmol, 3.0 equiv), 4-OMeTEMPO **3d** (37.3 mg, 0.2 mmol, 2.0 equiv), and tetra-butylammonium iodide (36.9 mg, 0.1 mmol, 1.0 equiv). The vial was capped and stirred at 40 °C in an aluminum heating block for 24 h. The resulting crude mixture was filtered through activated, neutral Al_2O_3 (Brockman Grade I, 58–60 Å mesh powder) and concentrated *in vacuo*. Product **4d** was not detected in the crude ^{1}H NMR in using dibromomethane (7.0 µL, 0.1 mmol) as an internal standard. Column chromatography (silica gel, 100% hexanes to 20% ethyl acetate–hexanes) was performed for clarity and no product was found.

<u>Reaction B</u>. To a 1-dram vial equipped with a Teflon-coated stir bar was added the 1-phenyl-1,3-butadiene **2a** (13.0 mg, 0.1 mmol, 1.0 equiv). 1,2-Dichloroethane (0.33 mL) was added, followed by sequential addition of sodium pentafluorobenzoate **1g** (70.2 mg, 0.3 mmol, 3.0 equiv), 4-OMeTEMPO oxoammonium salt **12** (54.6 mg, 0.2 mmol, 2.0 equiv), and tetra-butylammonium iodide (36.9 mg, 0.1 mmol, 1.0 equiv). The vial was capped and stirred at 40 °C in an aluminum heating block for 24 h. The resulting crude mixture was

filtered through activated, neutral Al_2O_3 (Brockman Grade I, 58–60 Å mesh powder) and concentrated *in vacuo*. Product **4d** was not detected in the crude ^{1}H NMR in using dibromomethane (7.0 µL, 0.1 mmol) as an internal standard.

7.5 Addition of acids with previously unreactive benzoic acid to promote TEMPO disproportionation

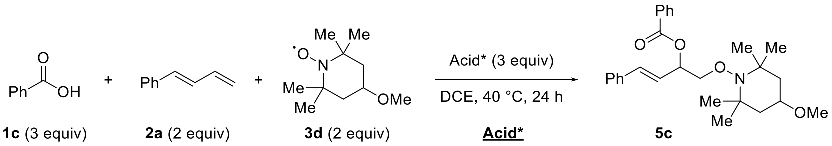

(*E*)-4-Phenylbut-3-ene-1,2-diol (5c). To a 1-dram vial equipped with a Teflon-coated stir bar was added the 1-phenyl-1,3-butadiene **2a** (13.0 mg, 0.1 mmol, 1.0 equiv). 1,2-Dichloroethane (0.33 mL) was added, followed by sequential addition of benzoic acid **1c** (36.6 mg, 0.3 mmol, 3.0 equiv), 4-OMeTEMPO **3d** (37.3 mg, 0.2 mmol, 2.0 equiv), and methanesulfonic acid (19.0 µL mg, 0.3 mmol, 3.0 equiv). The vial was capped and stirred at 40 °C in an aluminum heating block for 24 h. The resulting crude mixture was filtered through activated, neutral Al_2O_3 (Brockman Grade I, 58–60 Å mesh powder) and concentrated *in vacuo*. Purification by column chromatography (silica gel, 100% hexanes to 10% ethyl acetate–hexanes) as an off-white solid (21.0 mg, 48%). In the cases where TFA or TfOH were used instead of MsOH, product **5c** was not detected in the crude ^{1}H NMR in using dibromomethane (7.0 µL, 0.1 mmol) as an internal standard. R_f = 0.64 (25% EtOAc–hexanes); **^{1}H NMR** ($CDCl_3$, 400 MHz): δ 8.11 (d, *J* = 6.9 Hz, 2H), 7.57 (t, *J* = 7.5 Hz, 1H), 7.46 (t, *J* = 7.7 Hz, 2H), 7.39 (d, *J* = 7.0 Hz, 2H), 7.31 (t, *J* = 7.3 Hz, 2H), 7.25 (t, *J* = 7.2 Hz, 1H), 6.76 (d, *J* = 16.0 Hz, 1H), 6.31 (dd, *J* = 16.0, 7.1 Hz, 1H), 5.87 (td, *J* = 7.1, 3.7 Hz, 1H), 4.13 (dd, *J* = 9.9, 6.9 Hz, 1H), 4.07 (dd, *J* = 9.9, 3.8 Hz, 1H), 3.42 (tt, *J* = 11.4, 4.1 Hz, 1H), 3.31 (s, 3H), 1.88–1.81 (m, 2H), 1.38 (t, *J* = 12.1 Hz, 2H), 1.26 (s, 3H), 1.23 (s, 3H), 1.18 (s, 3H), 1.10 (s, 3H); **^{13}C{^{1}H} NMR** ($CDCl_3$, 100 MHz): δ 165.7, 136.2, 133.4, 132.9, 130.4, 129.6 (2C), 128.5 (2C), 128.3 (2C), 128.0, 126.6 (2C), 124.4, 77.9, 73.4, 71.6, 60.3, 60.2, 55.7, 44.5, 44.5, 33.2, 33.1, 21.0, 20.9; **FTIR** (thin film): cm^{-1} 2974, 2936, 1718, 1601, 1451, 1375, 1361, 1267, 1096, 711, 693; **HRMS** (ESI$^+$, m/z): Calcd for $C_{27}H_{36}NO_4^+$ ([M+H]$^+$): 438.2639; found: 438.2639.

8. UV-Vis Studies

First, the UV-Vis spectrum of TEMPO, TEMPO$^+$, pentafluorobenzoic acid (PFBA), and sodium pentafluorobenzoate were compared to the spectrum of TEMPO combined with PFBA (Figure S1). In this spectrum, the combined TEMPO & PFBA displayed a similar spectrum to the pentafluorobenzoate (PFBNa), possessing an elevated absorbance at the second λ_{max} of PFBA around 275 nm compared to pentafluorobenzoic acid alone. This result implies that PFBA is more deprotonated in the presence of TEMPO than without TEMPO, with disproportionation as a possible conclusion for this trend.

Figure S1. UV-Vis Spectroscopy of TEMPO, pentafluorobenzoic acid (PFBA), TEMPO & PFBA, and TEMPO$^+$. DCE (solvent) removed using double-beam UV-Vis instrument with DCE blank.

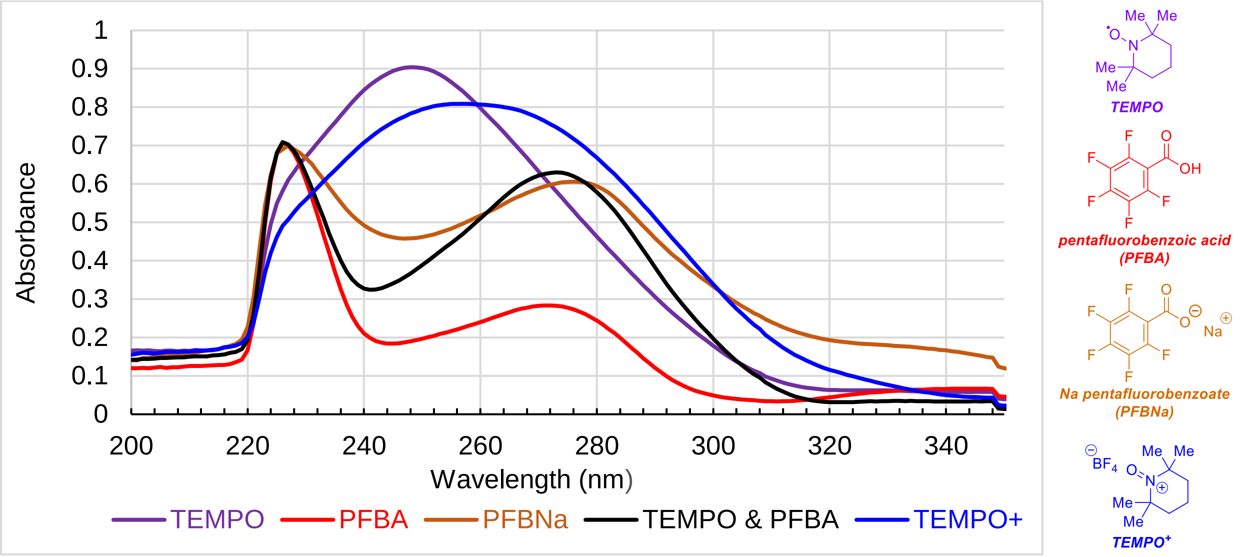

Secondly, benzoic acid was subjected to the same parameters as PFBA above (Figure S2). The combination of TEMPO and BzOH resulted in an identical spectrum to that of BzOH alone. This suggests that no effect is seen with BzOH creating any TEMPO disproportionation.

Figure S2. UV-Vis Spectroscopy of TEMPO, benzoic acid (BzOH), TEMPO & BzOH, and TEMPO$^+$ (BzOH and TEMPO & BzOH normalized). DCE (solvent) removed using double-beam UV-Vis instrument with DCE blank.

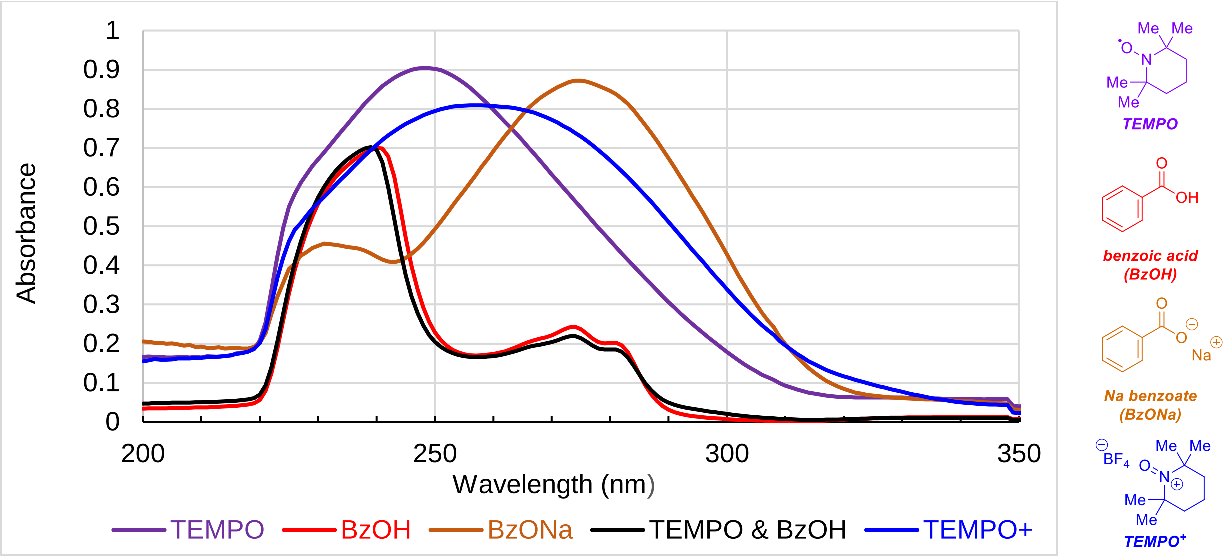

(To obtain UV-Vis spectra, solutions were made of each standard (TEMPO, acid, TEMPO$^+$), in DCE. The samples were diluted until the λ_{max} was under an absorbance of 1. For the combination spectra, the solutions were mixed, then diluted to until the λ_{max} was under an absorbance of 1. In the case of BzOH and TEMPO & BzOH, the absorbances exceeded 1 and were normalized concurrently.)

9. Computational Details

Density functions theory (DFT) calculations were all performed with Gaussian 16[13] and prepared using GaussView 6.[14] Structures were optimized in the gas phase with the wB97XD[15] functional and the def2-TZVP[16] basis set. Frequency calculations were performed at the same level of theory to confirm all structures were minima on the potential energy surface with zero imaginary frequencies. Single-point energy calculations were then performed at the same level of theory with SMD[17] solvation model in dichloroethane. The reported Gibbs energies were computed using the direct method, described previously,[18] and a correction for the change int state from 1 atm to 1 M was included.[19] The results presented here use the same methodology as Jiang et al,[20] and the gas phase thermodynamic results presented here are close to their results for TEMPO and its derivatives. The differences in the corrected Gibbs energies stem from using 1,2-dichloroethane as the solvent.

Table S1: Thermodynamic values for compounds reported in the manuscript. All units are in Hartrees unless otherwise noted. The species numbering in this table matches the numbering system in the manuscript.

Species	DFT energy	ZPVE	T.C.	S (J/mol K)	H	G_corrected
**BzOH 1c**	-420.8589	0.11680	0.00800	352.8660	-420.7341	-420.7711
**BzO (-)**	-420.3736	0.10264	0.00786	351.0334	-420.2631	-420.2999
**PFBA 1a**	-917.0853	0.07588	0.01248	447.5541	-916.9969	-917.0447
**PFBA(-)**	-916.6147	0.06181	0.01252	451.2026	-916.5404	-916.5886
**O_2**	-150.2796	0.00389	0.00331	195.6313	-150.2724	-150.2916
**O_2^-**	-150.4409	0.00289	0.00332	203.2796	-150.4347	-150.4547
**TEMPO 3a**	-483.7713	0.26405	0.01269	432.0398	-483.4946	-483.5406
**TEMPO+ ii**	-483.5874	0.26551	0.01270	428.3203	-483.3092	-483.3548
**TEMPOH+ i**	-484.1977	0.27688	0.01298	435.9393	-483.9078	-483.9543
**TEMPOH iii**	-484.3855	0.27611	0.01288	425.6174	-484.0965	-484.1418
**1-phenyl-1,3-butadiene 2a**	-387.0635	0.16800	0.00993	404.7476	-386.8856	-386.9285
**Cation Int. iv**	-870.6582	0.43705	0.02192	639.5746	-870.1991	-870.2688
**Radical Int. v**	-870.8307	0.43504	0.02197	648.4949	-870.3737	-870.4443
**BzOH product 5c**	-1291.1165	0.45896	0.11630	812.7462	-1290.5413	-1290.6305
**PFBA product 4d**	-1787.3442	0.50497	0.03391	901.1206	-1786.8054	-1786.9046
**PFBA Rad Prod int. vi**	-1787.7499	0.51787	0.03380	879.9454	-1787.1983	-1787.2951

Optimized solution phase structures and xyz coordinates in 1,2-dichloroethane

Benzoic Acid

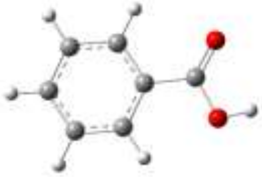

15
0 1
C -2.5553423 -0.0435320 0.0000054
C -1.8952114 1.17736720 0.0000023
C -0.5122504 1.21469650 -0.0000009
C 0.2157426 0.0292011 -0.0000012
C -0.4474056 -1.1940269 0.0000017
C -1.8319273 -1.2271138 0.0000051
H -3.6377811 -0.0725283 0.0000080
H -2.4603943 2.1003793 0.0000024
H 0.0225444 2.1551831 -0.0000033
H 0.1218202 -2.1130690 0.0000015
H -2.3479496 -2.1785272 0.0000076
C 1.6989258 0.1210271 -0.0000045
O 2.3227235 1.1489662 -0.0000021
O 2.3039739 -1.0800221 -0.0000054
H 3.2529934 -0.9087065 -0.0000045

Benzoic Acid (-)

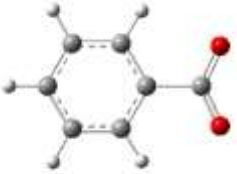

14
-1 1
C -2.5252638 -0.0000000 0.0000128
C -1.8243974 -1.1992475 0.0001066
C -0.4363871 -1.1945493 0.0001096
C 0.2754433 0.0000001 0.0000008
C -0.4363872 1.1945494 -0.0001054
C -1.8243975 1.1992474 -0.0000894
C 1.8271654 0.0000000 -0.0000135
O 2.3580774 -1.1259956 -0.0001101
O 2.3580777 1.1259955 0.0000906
H -2.3647323 -2.1404177 0.0001911
H 0.1378009 -2.1132330 0.0002004
H -3.6100314 -0.0000001 0.0000175
H -2.3647325 2.1404177 -0.0001724
H 0.1378007 2.1132332 -0.0002089

Pentafluorobenzoic acid

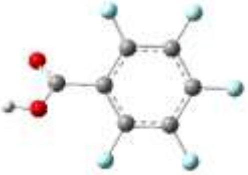

15
0 1
C 1.8914715 -0.0335302 0.0068357
C 1.2287566 1.1823580 0.0491726
C -0.1549498 1.2065546 0.0299012
C -0.8981089 0.0335853 -0.0159376
C -0.2112981 -1.1728651 -0.0522960
C 1.1720319 -1.2160421 -0.0474850
C -2.3926690 0.0982042 -0.0727006
O -3.0034869 0.7971031 -0.8263424
O -2.9610657 -0.7116452 0.8249541
H -3.9175769 -0.6403187 0.7113694
F -0.7581056 2.3812744 0.0916526
F 1.9202448 2.3085941 0.1131168
F 3.2105380 -0.0652714 0.0173125
F 1.8091931 -2.3747610 -0.1012542
F -0.8682490 -2.3201619 -0.1302948

Pentafluorobenzoic acid (-)

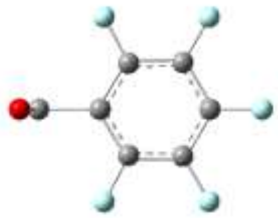

14
-1 1
C 1.8600097 -0.0000085 -0.0000104
C 1.1619835 1.1942152 -0.0000271
C -0.2231017 1.1756023 -0.0000205
C -0.9484398 0.0000091 0.0000049
C -0.2231164 -1.1755930 0.0000221
C 1.1619685 -1.1942234 0.0000138
C -2.5194490 0.0000189 0.0000146
O -3.0095428 -0.0000244 -1.1315828
O -3.0095276 0.0000273 1.1316188
F -0.8504240 2.3562579 -0.0000362
F 1.8367987 2.3496890 -0.0000506
F 3.1976913 -0.0000168 -0.0000177
F 1.8367692 -2.3497057 0.0000298
F -0.8504535 -2.3562408 0.0000444

Molecular oxygen

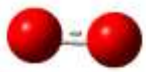

2
0 1
O 0.0000000 0.0000000 0.5969030
O O.0000000 0.0000000 -0.5969030

Reduced molecular oxygen

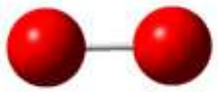

2
-1 2
O 0.0000000 0.0000000 0.6641320
O 0.0000000 0.0000000 -0.6641320

TEMPO

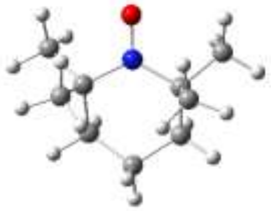

29
0 2
C 1.3800930 0.5003443 1.2363169
H 1.3901348 1.5948257 1.2361846
H 1.8872899 0.1801463 2.1491275
C 2.1130591 0.0091241 0.0000000
H 3.1369776 0.3874761 0.0000000
H 2.1877134 -1.0813373 0.0000000
C 1.3800930 0.5003443 -1.2363169
H 1.3901348 1.5948257 -1.2361846
H 1.8872899 0.1801463 -2.1491275
C -0.0739152 0.0246131 -1.3143947
C -0.0739152 0.0246131 1.3143947
N -0.7469656 0.2079970 -0.0000000
C -0.1564742 -1.4514847 -1.7234818
H -1.1870961 -1.7970711 -1.6448806
H 0.1705981 -1.5644108 -2.7585824
H 0.4709018 -2.0890833 -1.1013254
C -0.8266892 0.8722265 -2.3375921
H -0.8622482 1.9151707 -2.0197594
H -0.3096855 0.8186927 -3.2974622
H -1.8470255 0.5172800 -2.4642397

C	-0.1564742	-1.4514847	1.7234818
H	-1.1870961	-1.7970711	1.6448806
H	0.4709018	-2.0890833	1.1013254
H	0.1705981	-1.5644108	2.7585824
C	-0.8266892	0.8722265	2.3375921
H	-0.3096855	0.8186927	3.2974622
H	-0.8622482	1.9151707	2.0197594
H	-1.8470255	0.5172800	2.4642397
O	-2.0095248	0.0854557	-0.0000000

TEMPO+

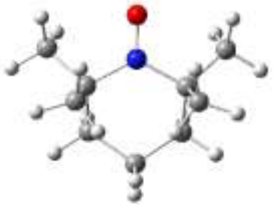

29

1 1

C	-1.3426292	-0.5680137	1.2428830
H	-1.2755884	-1.6588175	1.2723270
H	-1.8650770	-0.2624529	2.1502774
C	-2.1021389	-0.1397363	0.0000000
H	-3.0834594	-0.6135282	0.0000000
H	-2.2863488	0.9360355	0.0000000
C	-1.3426292	-0.5680137	-1.2428830
H	-1.2755884	-1.6588175	-1.2723270
H	-1.8650770	-0.2624529	-2.1502774
C	0.0751466	0.0106200	-1.3460083
C	0.0751466	0.0106200	1.3460083
N	0.7708955	-0.1562781	0.0000000
C	0.0751466	1.5204358	-1.656447
H	1.0710204	1.9482473	-1.5311756
H	-0.2189194	1.6280270	-2.6891790
H	-0.6312570	2.0779259	-1.0383545
C	0.9030155	-0.7267275	-2.3879499
H	1.0517140	-1.7724904	-2.1181933
H	0.3452992	-0.6956306	-3.3233689
H	1.8724007	-0.2615998	-2.5538990
C	0.0751466	1.5204358	1.6456447
H	1.0710204	1.9482473	1.5311756
H	-0.6312570	2.0779259	1.0383545
H	-0.2189194	1.6280270	2.6891790
C	0.9030155	-0.7267275	2.3879499
H	0.3452992	-0.6956306	3.3233689
H	1.0517140	-1.7724904	2.1181933
H	1.8724007	-0.2615998	2.5538990
O	1.9198793	-0.4040420	0.0000000

TEMPOH+

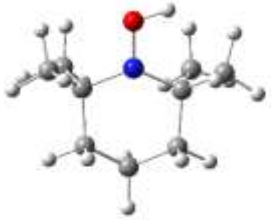

30

1 2

C	-1.2563166	1.3815792	-0.5333538
H	-1.2738018	1.3577780	-1.6257026
H	-2.1676562	1.8849517	-0.2101863
C	-0.0196993	2.1329350	-0.0726820
H	-0.0273910	3.1353811	-0.4996017
H	-0.0158469	2.2606048	1.0116963
C	1.2243522	1.3999061	-0.5430569
H	1.2315945	1.3676137	-1.6352787
H	2.1313995	1.9181093	-0.2312501
C	1.3307087	-0.0327286	-0.0098426
C	-1.3396510	-0.0581075	-0.0132024
N	-0.0007231	-0.6845891	-0.1601896
C	1.7079133	-0.0642182	1.4795000
H	1.6456717	-1.0737129	1.8850135
H	2.7392764	0.2754925	1.5660840
H	1.0803705	0.5930701	2.0756919
C	2.3572242	-0.8163945	-0.8289707
H	2.0261791	-0.9643188	-1.8564384
H	3.2732197	-0.2285637	-0.8497042
H	2.6302039	-1.7766434	-0.3826309
C	-1.7222871	-0.1127873	1.4743091
H	-1.6544049	-1.1309246	1.8554903
H	-1.1025394	0.5389579	2.0856080
H	-2.7559065	0.2198552	1.5611809
C	-2.3445281	-0.8564809	-0.8422331
H	-3.2817690	-0.3013333	-0.8405601
H	-2.0095779	-0.9599145	-1.8738959
H	-2.5296760	-1.8429102	-0.4230290
O	-0.0515770	-1.9970904	-0.1196935
H	0.8520346	-2.3468668	-0.1964172

TEMPOH

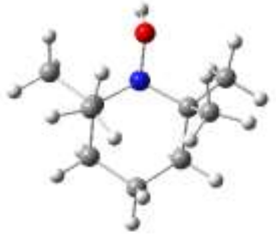

30

0 1

C	1.4171110	0.4734626	1.2416879
H	1.4482519	1.5668991	1.2719681
H	1.9062570	0.1129475	2.1497350
C	2.1529703	-0.0034022	-0.0000000
H	3.1737591	0.3844258	-0.0000000
H	2.2403899	-1.0927850	-0.0000000
C	1.4171110	0.4734626	-1.2416879
H	1.4482519	1.5668991	-1.2719681
H	1.9062570	0.1129475	-2.1497350
C	-0.0515464	0.0324887	-1.2802961
C	-0.0515464	0.0324887	1.2802961
N	-0.6662124	0.4399396	0.0000000
C	-0.1716073	-1.4645802	-1.6058603
H	-1.1872335	-1.8115841	-1.4188649
H	0.0535376	-1.6250877	-2.6618772
H	0.5143690	-2.0802248	-1.0279710
C	-0.7633152	0.8201779	-2.3830631
H	-0.7826899	1.8827212	-2.1348475
H	-0.2368817	0.6941898	-3.3310938
H	-1.7872453	0.4711200	-2.5168314
C	-0.1716072	-1.4645802	1.6058603
H	-1.1872335	-1.8115841	1.4188649
H	0.5143690	-2.0802248	1.0279710
H	0.0535376	-1.6250877	2.6618772
C	-0.7633152	0.8201779	2.3830631
H	-0.2368817	0.6941898	3.3310938
H	-0.7826899	1.8827212	2.1348475
H	-1.7872453	0.4711200	2.5168315
O	-2.0218328	0.0076517	0.0000000
H	-2.5182576	0.8274314	0.0000000

1-Phenyl-1,3-butadiene

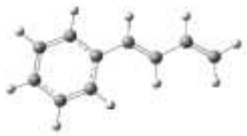

20

0 1

C	3.2412900	0.3819780	0.0000000
C	2.2808220	1.3851180	0.0000000
C	0.9355170	1.0664450	0.0000000
C	0.5144270	-0.2659910	0.0000000
C	1.4923900	-1.2614290	0.0000000
C	2.8409600	-0.9442330	0.0000000
H	4.2937560	0.6352110	0.0000000
H	2.5846370	2.4244760	0.0000000
H	0.2061830	1.8662740	0.0000010
H	1.1863440	-2.3013390	0.0000000
H	3.5798500	-1.7356740	-0.0000010
C	-0.8964110	-0.6624580	0.0000000
H	-1.0707230	-1.7357420	0.0000010
C	-1.9644680	0.1395410	0.0000000
H	-1.8433740	1.2188040	-0.0000010
C	-3.3306850	-0.3505930	0.0000000
H	-3.4572740	-1.4299370	0.0000010
C	-4.4063960	0.4324200	0.0000000
H	-5.4066050	0.0199960	0.0000000
H	-4.3174630	1.5131410	-0.0000010

Cation intermediate

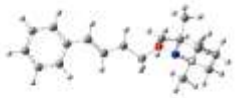

49

1 1

C	-6.9140054	-0.8505253	0.5379306

C	-7.3549009	0.1641533	-0.3044496
C	-6.4542842	1.0325524	-0.9219553
C	-5.1068227	0.8906845	-0.6997932
C	-4.6349979	-0.1328135	0.1525554
C	-5.5652729	-0.9999528	0.7661178
H	-7.6253992	-1.5149138	1.0084775
H	-8.4158439	0.2834720	-0.4851184
H	-6.8193979	1.8145527	-1.5733864
H	-4.4133633	1.5666036	-1.1811650
H	-5.2072044	-1.7857463	1.4201453
C	-3.2659931	-0.3320472	0.4231434
H	-3.0293843	-1.1547070	1.0938108
C	-2.1789505	0.3956364	-0.0598158
H	-2.3227851	1.2387010	-0.7242565
C	-0.9196758	0.0329574	0.3117675
H	-0.7832445	-0.8063674	0.9898861
C	0.3153510	0.6879970	-0.1471164
H	0.4624623	0.4513068	-1.2123864
H	0.2000942	1.7818513	-0.0992820
O	1.3958514	0.2434383	0.6081824
N	2.5676912	0.1480991	-0.2033357
C	3.5427767	1.1635140	0.2629518
C	4.7882325	1.0149964	-0.6211691
H	5.5482396	1.7057084	-0.2507772
H	4.5319186	1.3340210	-1.6357707
C	5.3224593	-0.4067747	-0.6704418
H	5.7022864	-0.7107490	0.3075545
H	6.1701774	-0.4599986	-1.3549590
C	4.2258116	-1.3493081	-1.1373274
H	4.5742933	-2.3840968	-1.1423756
H	3.9560403	-1.0963795	-2.1669218
C	2.9585472	-1.2802503	-0.2737932
C	3.9044526	1.0786260	1.7522973
H	4.3995070	2.0031904	2.0522818
H	3.0090219	0.9645870	2.3626341
H	4.5821469	0.2598948	1.9796931
C	2.9254726	2.5387878	-0.0010367
H	2.5448984	2.6014620	-1.0220119
H	2.1185940	2.7564536	0.7011854
H	3.6831734	3.3123316	0.1280737
C	3.1740959	-1.9607888	1.0853755
H	2.3636626	-1.7203686	1.7732121
H	3.1903281	-3.0427609	0.9468757
H	4.1130007	-1.6801182	1.5548300
C	1.8416599	-2.0253670	-1.0095553
H	0.9390410	-2.1036189	-0.3993345
H	1.5978716	-1.5294226	-1.9507275
H	2.1654776	-3.0415555	-1.2364102

Radical intermediate

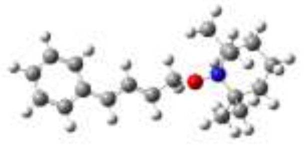

49

0 2

C	-6.9904300	-0.5995620	0.5875760
C	-7.2603940	0.6677830	0.0878060
C	-6.2203460	1.4349310	-0.4242370
C	-4.9287630	0.9477470	-0.4389930
C	-4.6320400	-0.3331320	0.0619290
C	-5.7000140	-1.0911180	0.5748850
H	-7.7924540	-1.2062200	0.9890960
H	-8.2709540	1.0552300	0.0967440
H	-6.4221950	2.4242140	-0.8157720
H	-4.1393050	1.5672680	-0.8435560
H	-5.4976530	-2.0810540	0.9670280
C	-3.3113670	-0.8925010	0.0729320
H	-3.2175530	-1.8920130	0.4867160
C	-2.1380020	-0.2837790	-0.3891060
H	-2.2005800	0.7162780	-0.8087260
C	-0.9099740	-0.8682820	-0.3500820
H	-0.8003860	-1.8597700	0.0797970
C	0.3273010	-0.2373590	-0.8792800
H	0.7279950	-0.8125490	-1.7200400
H	0.1246200	0.7764490	-1.2394340
O	1.2847230	-0.2183680	0.1699610
N	2.5888650	0.0415580	-0.3214560
C	3.0341570	1.3488110	0.2125840
C	4.4611780	1.5873450	-0.2971880
H	4.8284140	2.5182240	0.1415540
H	4.4175970	1.7369111	-1.3803870
C	5.4052340	0.4347220	0.0017890
H	5.5616870	0.3357640	1.0788290
H	6.3871260	0.6345310	-0.4322240
C	4.8348750	-0.8498320	-0.5754760
H	5.4745100	-1.7036740	-0.3393400
H	4.8033360	-0.7630150	-1.6658490

C	3.4177150	-1.1616310	-0.0776010
C	2.9635730	1.4821460	1.7425770
H	3.0277340	2.5369880	2.0160850
H	2.0154840	1.0927780	2.1115960
H	3.7719000	0.9639430	2.2533280
C	2.1268310	2.4232660	-0.3918240
H	2.0607780	2.3032660	-1.4740360
H	1.1236330	2.3766020	0.0329440
H	2.5345620	3.4126420	-0.1759300
C	3.4370040	-1.6465650	1.3811330
H	2.4280330	-1.6465540	1.7919040
H	3.8200570	-2.6680410	1.4180240
H	4.0699930	-1.0381880	2.0229330
C	2.8516380	-2.2930580	-0.9396520
H	1.8738060	-2.6145480	-0.5795980
H	2.7595290	-1.9748770	-1.9789460
H	3.5213790	-3.1541420	-0.9008690

BzOH product

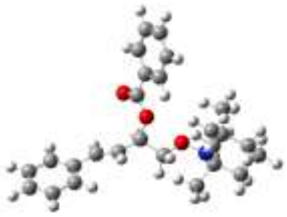

63

0 1

C	7.1850890	-1.9334830	-0.6909150
C	6.1463910	-2.0716480	-1.6017450
C	4.8470320	-1.7793880	-1.2283750
C	4.5580830	-1.3365140	0.0632520
C	5.6091720	-1.2154940	0.9709820
C	6.9115880	-1.5062880	0.5984570
H	8.2008770	-2.1653080	-0.9847590
H	6.3508860	-2.4167540	-2.6074890
H	4.0465020	-1.9120750	-1.9452290
H	5.3998780	-0.8798370	1.9800170
H	7.7136280	-1.4001480	1.3179110
C	3.1985130	-0.9938930	0.5017400
C	2.1497150	-0.7538550	-0.2769620
H	3.0641480	-0.9040520	1.5764290
C	0.8003860	-0.4089820	0.2659080
H	2.2367490	-0.7732990	-1.3595780
H	0.7974370	-0.4839300	1.3530330
C	-0.2904200	-1.2853650	-0.3104680
H	-0.0068140	-2.3347220	-0.1789100
H	-0.4084320	-1.0943740	-1.3799270
O	-1.4765470	-0.9841610	0.3868510
N	-2.6200470	-1.5141100	-0.2608840
C	-4.4853600	-3.0564510	-0.0608990
H	-4.1841410	-3.6160000	-0.9518680
H	-4.9733450	-3.7641540	0.6135100
C	-5.4411800	-1.9501570	-0.4752760
H	-6.2945660	-2.3747470	-1.0080510
H	-5.8497590	-1.4454720	0.4036990
C	-4.7117230	-0.9611650	-1.3686000
H	-5.3644590	-0.1316750	-1.6508200
H	-4.4205200	-1.4699060	-2.2927110
C	-3.4487000	-0.3755400	-0.7247550
C	-2.6565030	0.3552110	-1.8116340
C	-3.8016050	0.6486090	0.3652770
H	-2.3301220	-0.3436380	-2.5834720
H	-3.2878840	1.1141760	-2.2775460
H	-1.7842560	0.8537000	-1.3901640
H	-4.5753830	0.2980150	1.0443270
H	-2.9164210	0.8990330	0.9487200
H	-4.1669030	1.5637230	-0.1041170
C	-3.2197900	-2.5339980	0.6301630
C	-3.5301770	-2.0475040	2.0549600
C	-2.2216450	-3.6896320	0.7361250
H	-4.4235300	-1.4297020	2.1084810
H	-3.6903150	-2.9108450	2.7034500
H	-2.6919680	-1.4758440	2.4513160
H	-1.8878540	-3.9984810	-0.2554100
H	-1.3524970	-3.4082050	1.3314910
H	-2.6964410	-4.5426210	1.2245550
O	0.4706160	0.9467030	-0.0950650
C	0.7185650	1.9049830	0.8051160
C	0.3045880	3.2515990	0.3172530
C	-0.1857460	3.4566540	-0.9682250
C	0.4174580	4.3252470	1.1938670
C	-0.5626620	4.7272880	-1.3699840
C	0.0376830	5.5933600	0.7905560
C	-0.4532080	5.7952170	-0.4916260
O	1.2077660	1.7031740	1.8854860
H	-0.9430120	4.8844710	-2.3711600
H	-0.7499970	6.7876110	-0.8073520
H	0.1244080	6.4264060	1.4762420
H	0.8035510	4.1464490	2.1885520
H	-0.2670750	2.6223970	-1.6503180

PFBA Product

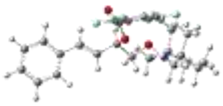

63
0 1

C	-5.8162430	-4.7684870	0.7306640
C	-4.7778990	-4.5722650	1.6311280
C	-3.6364360	-3.8945240	1.2427740
C	-3.5114440	-3.3937300	-0.0538020
C	-4.5555710	-3.6105300	-0.9515510
C	-5.7005650	-4.2870450	-0.5634750
H	-6.7076710	-5.3011720	1.0363650
H	-4.8565430	-4.9570440	2.6401200
H	-2.8270730	-3.7682430	1.9508760
H	-4.4695290	-3.2331100	-1.9637940
H	-6.5028090	-4.4393410	-1.2742570
C	-2.3301910	-2.6485880	-0.5074660
C	-1.4151950	-2.0694180	0.2616790
H	-2.2289810	-2.5439420	-1.5841740
C	-0.2433600	-1.3258090	-0.2929110
H	-1.4954820	-2.0895820	1.3446600
H	-0.1663630	-1.4710150	-1.3703320
C	1.0485630	-1.7279270	0.3795990
H	1.1084890	-2.8205970	0.3839230
H	1.0565230	-1.3824610	1.4165360
O	2.1116270	-1.1624100	-0.3489710
N	3.3431510	-1.3207450	0.3380670
C	5.5421530	-2.3439480	0.2880610
H	5.3632650	-2.8912910	1.2186070
H	6.2163970	-2.9530590	-0.3187140
C	6.1716280	-1.0015700	0.6209300
H	7.0841390	-1.1520770	1.2014020
H	6.4727440	-0.4819470	-0.2920010
C	5.1847910	-0.1611940	1.4139870
H	5.5965670	0.8276210	1.6293490
H	4.9982800	-0.6488560	2.3756870
C	3.8396660	0.0280250	0.7015810
C	2.8494830	0.6265770	1.7038150
C	3.9703140	1.0045900	-0.4781490
H	2.6835870	-0.0610300	2.5347350
H	3.2538200	1.5580360	2.1041960
H	1.8942240	0.8513410	1.2304680
H	4.8616770	0.8297570	-1.0764150
H	3.0986920	0.9352970	-1.1263630
H	4.0259940	2.0260210	-0.0985930
C	4.2080440	-2.2203290	-0.4594950
C	4.4275250	-1.7848550	-1.9170960
C	3.5464390	-3.6006600	-0.4787030
H	5.1285140	-0.9594180	-2.0140640
H	4.8291430	-2.6244620	-2.4873810
H	3.4823190	-1.4884470	-2.3704960
H	3.2782680	-3.9115460	0.5319170
H	2.6514090	-3.6035800	-1.1017580
H	4.2404830	-4.3339210	-0.8934320
O	-0.4165370	0.0951490	-0.0406770
C	-1.1400200	0.7777710	-0.9182920
C	-1.2978030	2.2076250	-0.4805320
C	-0.2155480	3.0669110	-0.3950510
C	-2.5555830	2.7074440	-0.1834730
C	-0.3722190	4.3916100	-0.0260060
C	-2.7349420	4.0265970	0.1962330
C	-1.6378310	4.8693760	0.2721910
O	-1.6245590	0.3452450	-1.9235440
F	1.0028120	2.6366530	-0.6935850
F	0.6733630	5.2019100	0.0388500
F	-3.9413970	4.4854250	0.4912080
F	-3.6160110	1.9152920	-0.2331090
F	-1.7990930	6.1313330	0.6290970

PFBA product intermediate

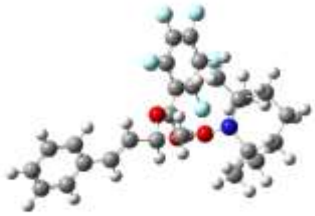

64
1 2

C	-8.1297540	-0.9579520	-0.4699790

C	-7.1655310	-0.8290560	-1.4621660
C	-5.8618240	-0.5181990	-1.1283870
C	-5.4979510	-0.3315440	0.2073890
C	-6.4767870	-0.4599900	1.1926350
C	-7.7837960	-0.7723720	0.8583570
H	-9.1503640	-1.2002520	-0.7361000
H	-7.4360200	-0.9696590	-2.5004520
H	-5.1273220	-0.4160530	-1.9169530
H	-6.2095600	-0.3135150	2.2327810
H	-8.5314200	-0.8689110	1.6344820
C	-4.1337520	-0.0092970	0.6215160
C	-3.0547480	0.1132840	-0.1527030
H	-4.0102790	0.1500720	1.6905860
C	-1.7441710	0.4948200	0.4051230
H	-3.0989180	-0.0322320	-1.2258520
H	-1.7871900	0.6875030	1.4738380
C	-1.0420990	1.6103800	-0.3373910
H	-1.6547370	2.5094540	-0.2274980
H	-0.9678920	1.3729770	-1.4013600
O	0.2292610	1.7658050	0.2406640
N	1.0181330	2.6996000	-0.4927200
C	2.1274340	4.8543500	-0.4353820
H	1.5366820	5.2363350	-1.2727320
H	2.3635920	5.7068740	0.2042500
C	3.3941450	4.2136500	-0.9765350
H	3.9420200	4.9326090	-1.5869950
H	4.0659760	3.9366250	-0.1604160
C	3.0318970	3.0002030	-1.8157620
H	3.9277430	2.4925350	-2.1804460
H	2.4718630	3.3316350	-2.6944470
C	2.1765660	1.9732710	-1.0613240
C	1.6368130	0.9707570	-2.0838010
C	3.0279500	1.2096010	-0.0286700
H	0.9896540	1.4688740	-2.8059730
H	2.4662930	0.5155840	-2.6271390
H	1.0692070	0.1707500	-1.6075600
H	3.7685350	1.8402920	0.4568920
H	2.3971630	0.7831740	0.7508800
H	3.5661680	0.3984140	-0.5229900
C	1.2511580	3.8853980	0.3693220
C	1.8786300	3.5726060	1.7354300
C	-0.1022210	4.5563400	0.6118470
H	2.9311600	3.3078710	1.6727010
H	1.8025800	4.4530100	2.3745500
H	1.3428800	2.7592660	2.2256590
H	-0.6305200	4.7114640	-0.3296700
H	-0.7282080	3.9671130	1.2837740
H	0.0513750	5.5290250	1.0804610
O	-0.8114080	-0.7029330	0.2171060
C	0.1265130	-0.9560990	1.0123520
C	1.1765410	-1.8727740	0.5834400
C	1.1377500	-2.5038480	-0.6715780
C	2.3096730	-2.1092450	1.3763520
C	2.1767210	-3.3112390	-1.1002370
C	3.3514830	-2.9049910	0.9647900
C	3.2826490	-3.5101920	-0.2868250
O	0.1110940	-0.4188480	2.1844180
F	0.1181500	-2.3612870	-1.4820540
F	2.1171700	-3.8903180	-2.2764860
F	4.3989330	-3.0913120	1.7333700
F	2.4177210	-1.5344400	2.5782140
F	4.2621850	-4.2689940	-0.6921080
H	0.8938720	-0.6402320	2.7119580

10. References

1. (a) Borodkin, G. I.; Elanov, I. R.; Shakirov, M. M.; Shubin, V. G., "Reaction of Nitrosonium Tetrafluoroborate with Nitroxyl Radicals." *Russ. J. Org. Chem.* **2003,** *39*, 1144-1150; (b) Cheng, H.-P.; Yang, X.-H.; Lan, L.; Xie, L.-J.; Chen, C.; Liu, C.; Chu, J.; Li, Z.-Y.; Liu, L.; Zhang, T.-Q.; Luo, D.-Q.; Cheng, L., "Chemical Deprenylation of N6-Isopentenyladenosine (i6A) RNA." *Angew. Chem. Int. Ed.* **2020,** *59*, 10645-10650.
2. Hemric, B. N.; Chen, A. W.; Wang, Q., "Copper-Catalyzed 1,2-Amino Oxygenation of 1,3-Dienes: A Chemo-, Regio-, and Site-Selective Three-Component Reaction with O-Acylhydroxylamines and Carboxylic Acids." *ACS Catal.* **2019,** *9*, 10070-10076.
3. Menard, T.; Laverny, A.; Denmark, S. E., "Synthesis of Enantioenriched 3,4-Disubstituted Chromans through Lewis Base Catalyzed Carbosulfenylation." *J. Org. Chem.* **2021,** *86*, 14290-14310.
4. Mundal, D. A.; Lutz, K. E.; Thomson, R. J., "Stereoselective Synthesis of Dienes from N-Allylhydrazones." *Org. Lett.* **2009,** *11*, 465-468.
5. Bhowmik, A.; Fernandes, R. A., "Iron(III)/O2-Mediated Regioselective Oxidative Cleavage of 1-Arylbutadienes to Cinnamaldehydes." *Org. Lett.* **2019,** *21*, 9203-9207.
6. Gao, W.; Zhang, D.; Zhang, X.; Cai, X.; Xie, P.; Loh, T.-P., "One-Pot and Unsymmetrical Bis-Allylation of Malononitrile with Conjugated Dienes and Allylic Alcohols." *Org. Lett.* **2022,** *24*, 9355-9360.
7. (a) Barluenga, J.; Tomás-Gamasa, M.; Aznar, F.; Valdés, C., "Synthesis of Dienes by Palladium-Catalyzed Couplings of Tosylhydrazones with Aryl and Alkenyl Halides." *Adv. Synth. Catal.* **2010,** *352*, 3235-3240; (b) Fuchter, M. J.; Levy, J.-N., "One-Pot Formation of Allylic Chlorides from Carbonyl Derivatives." *Org. Lett.* **2008,** *10*, 4919-4922.
8. Al-Huniti, M. H.; Perez, M. A.; Garr, M. K.; Croatt, M. P., "Palladium-Catalyzed Chemoselective Protodecarboxylation of Polyenoic Acids." *Org. Lett.* **2018,** *20*, 7375-7379.
9. Nguyen, V. T.; Dang, H. T.; Pham, H. H.; Nguyen, V. D.; Flores-Hansen, C.; Arman, H. D.; Larionov, O. V., "Highly Regio- and Stereoselective Catalytic Synthesis of Conjugated Dienes and Polyenes." *J. Am. Chem. Soc.* **2018,** *140*, 8434-8438.
10. Preuß, T.; Saak, W.; Doye, S., "Titanium-Catalyzed Intermolecular Hydroaminoalkylation of Conjugated Dienes." *Chem. Eur. J.* **2013,** *19*, 3833-3837.
11. (a) Matsubara, R.; Jamison, T. F., "Nickel-Catalyzed Allylic Substitution of Simple Alkenes." *J. Am. Chem. Soc.* **2010,** *132*, 6880-6881; (b) Watkins, A. L.; Landis, C. R., "Regioselective Rhodium-Catalyzed Hydroformylation of 1,3-Dienes to Highly Enantioenriched β,γ-Unsaturated Aldehyes with Diazaphospholane Ligands." *Org. Lett.* **2011,** *13*, 164-167.
12. Shi, M.; Wang, B.-Y.; Huang, J.-W., "Palladium-Catalyzed Isomerization of Methylenecyclopropanes in Acetic Acid." *J. Org. Chem.* **2005,** *70*, 5606-5610.
13. Frisch, M. J.; Trucks, G. W.; Schlegel, H. B.; Scuseria, G. E.; Robb, M. A.; Cheeseman, J. R.; Scalmani, G.; Barone, V.; Petersson, G. A.; Nakatsuji, H.; Li, X.; Caricato, M.; Marenich, A. V.; Bloino, J.; Janesko, B. G.; Gomperts, R.; Mennucci, B.; Hratchian, H. P.; Ortiz, J. V.; Izmaylov, A. F.; Sonnenberg, J. L.; Williams; Ding, F.; Lipparini, F.; Egidi, F.; Goings, J.; Peng, B.; Petrone, A.; Henderson, T.; Ranasinghe, D.; Zakrzewski, V. G.; Gao, J.; Rega, N.; Zheng, G.; Liang, W.; Hada, M.; Ehara, M.; Toyota, K.; Fukuda, R.; Hasegawa, J.; Ishida, M.; Nakajima, T.; Honda, Y.; Kitao, O.; Nakai, H.; Vreven, T.; Throssell, K.; Montgomery Jr., J. A.; Peralta, J. E.; Ogliaro, F.; Bearpark, M. J.; Heyd, J. J.; Brothers, E. N.; Kudin, K. N.; Staroverov, V. N.; Keith, T. A.; Kobayashi, R.; Normand, J.; Raghavachari, K.; Rendell, A. P.; Burant, J. C.; Iyengar, S. S.; Tomasi, J.; Cossi, M.; Millam, J. M.; Klene, M.; Adamo, C.; Cammi, R.; Ochterski, J. W.; Martin, R. L.; Morokuma, K.; Farkas, O.; Foresman, J. B.; Fox, D. J. Gaussian 16 Rev. C.01, 2016.
14. Dennington, R.; Keith, T. A.; Millam, J. M. GaussView Version 6, 2019.
15. Chai, J.-D.; Head-Gordon, M., "Long-range corrected hybrid density functionals with damped atom–atom dispersion corrections." *Phys. Chem. Chem. Phys.* **2008**, *10*, 6615-6620.
16. Weigend, F.; Ahlrichs, R., "Balanced basis sets of split valence, triple zeta valence and quadruple zeta valence quality for H to Rn: Design and assessment of accuracy." *Phys. Chem. Chem. Phys.* **2005**, *7*, 3297-3305.
17. Marenich, A. V.; Cramer, C. J.; Truhlar, D. G. Universal Solvation Model Based on Solute Electron Density and on a Continuum Model of the Solvent Defined by the Bulk Dielectric Constant and Atomic Surface Tensions. *J. Phys. Chem. B* **2009**, *113*, 6378–6396.
18. Ribeiro, R. F.; Marenich, A. V.; Cramer, C. J.; Truhlar, D. G. Use of Solution-Phase Vibrational Frequencies in Continuum Models for the Free Energy of Solvation. *J. Phys. Chem. B* **2011**, *115*, 14556–14562.
19. Ho, J.; Klamt, A.; Coote, M. L. Comment on the Correct Use of Continuum Solvent Models. *J. Phys. Chem. A* **2010**, *114*, 13442–13444.
20. Jiang, S.; Xie, Y.; Xie, Y.; Yu, L.-J.; Yan, X.; Zhao, F.-G.; Mudugamuwa, C. J.; Coote, M. L.; Jia, Z.; Zhang, K., "Lewis Acid-Induced Reversible Disproportionation of TEMPO Enables Aqueous Aluminum Radical Batteries." *J. Am. Chem. Soc.* **2023**, *145*, 14519-14528.

11. ^{1}H, ^{13}C, and ^{19}F NMR Spectra

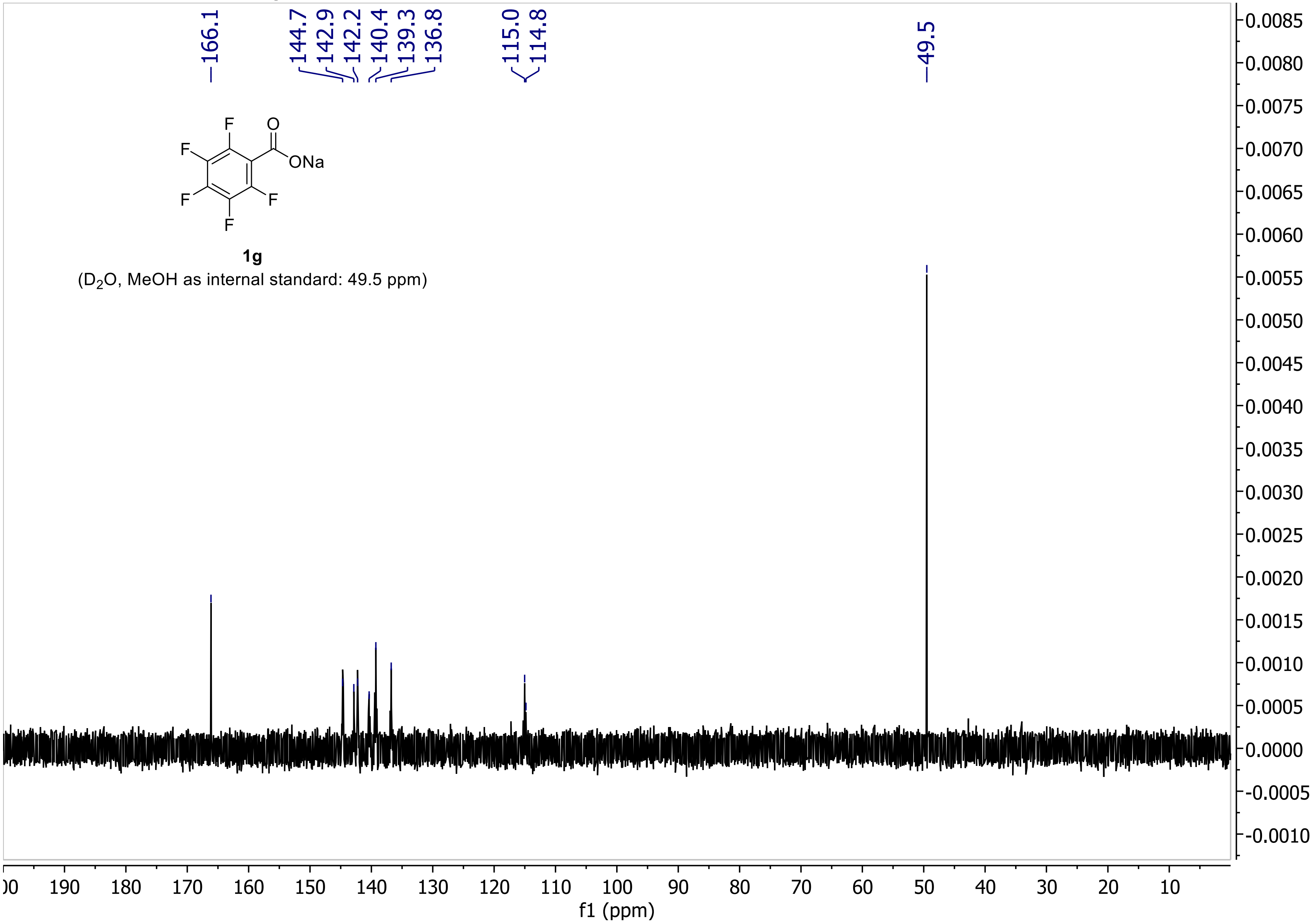

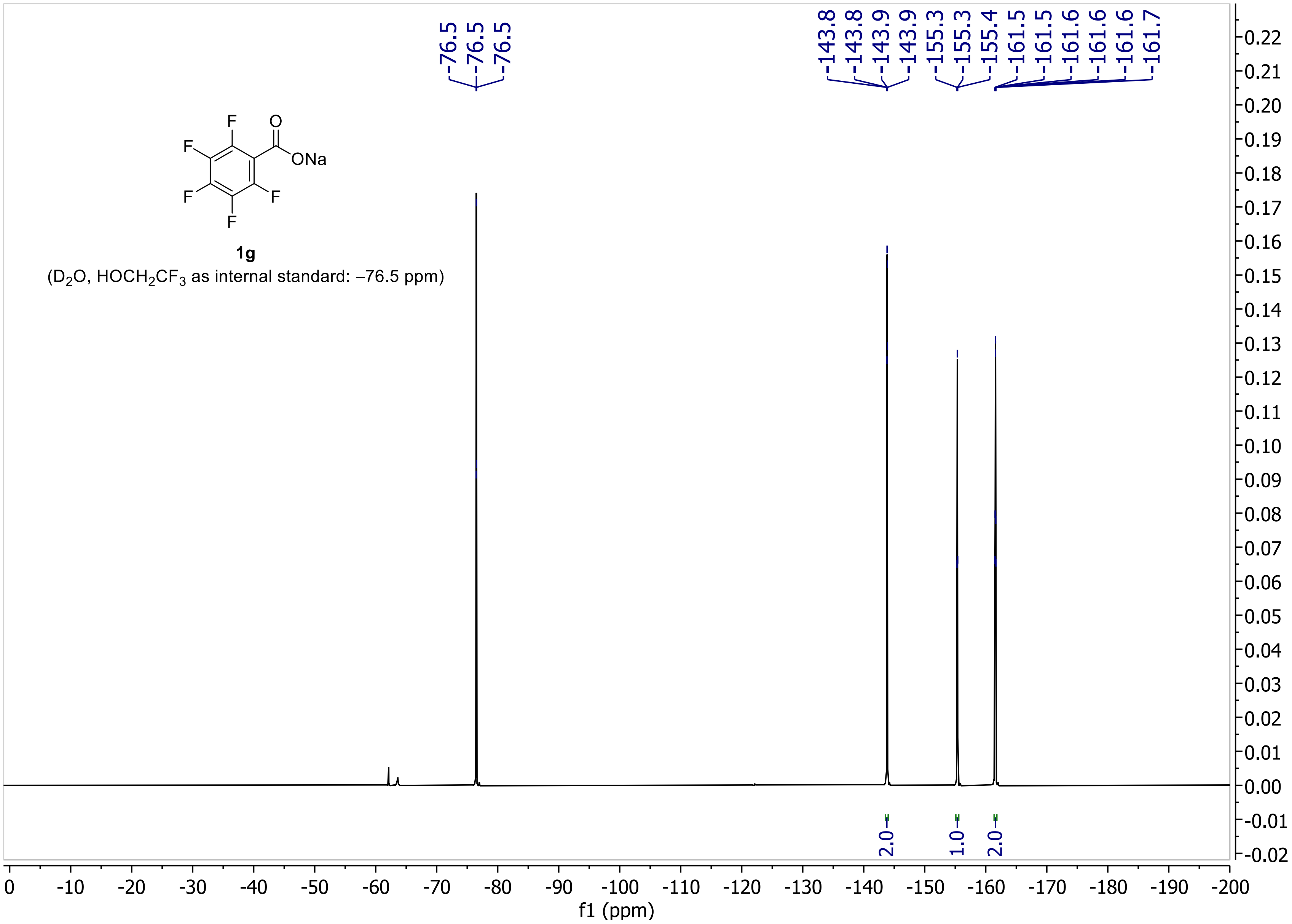
F
O
F
ONa
F
F
F
1g
(D_2O, $HOCH_2CF_3$ as internal standard: –76.5 ppm)
-76.5
-76.5
-76.5
-143.8
-143.8
-143.9
-143.9
-155.3
-155.3
-155.4
-161.5
-161.5
-161.6
-161.6
-161.6
-161.7
2.0
1.0
2.0
f1 (ppm)

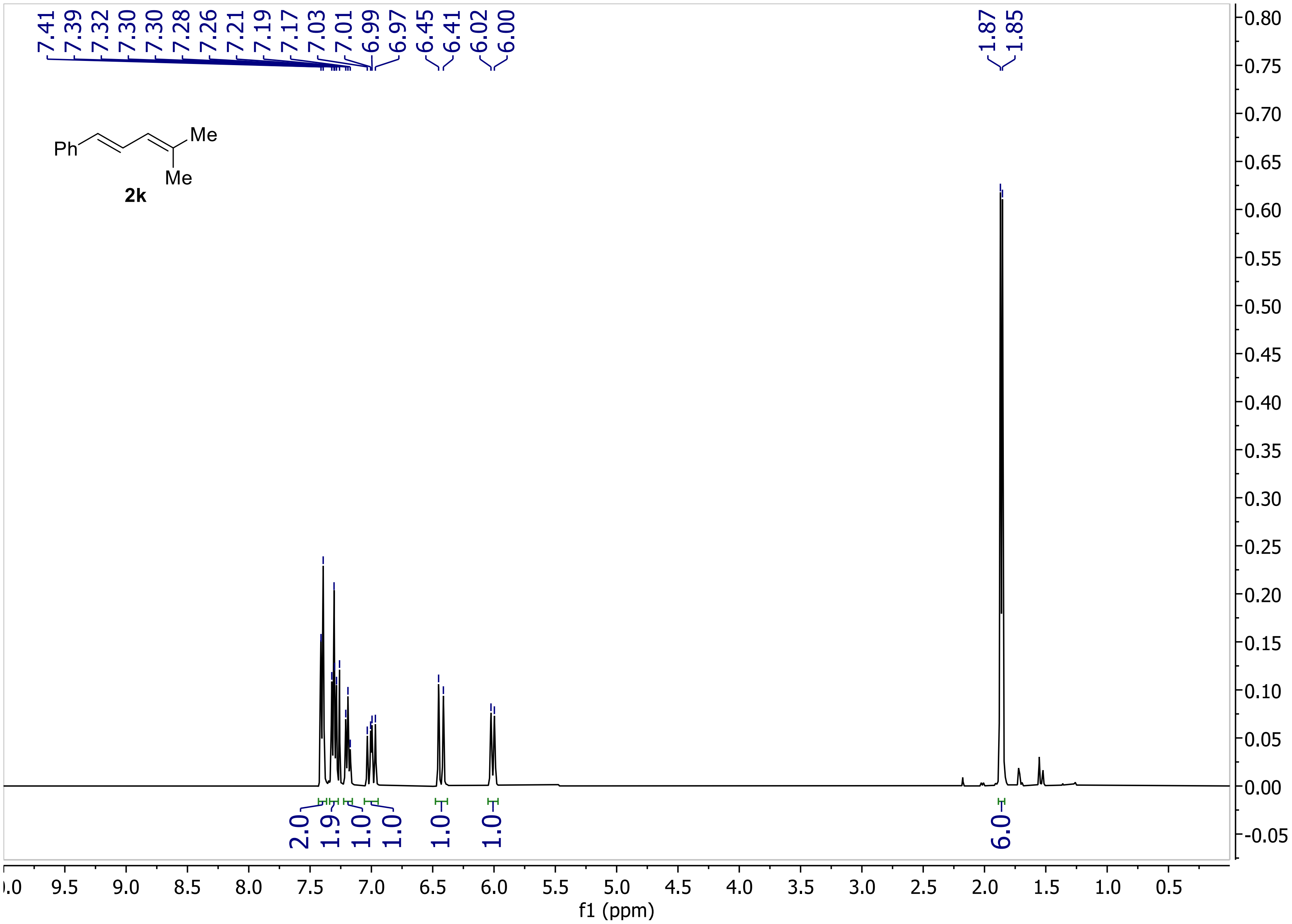
7.41
7.39
7.32
7.30
7.30
7.28
7.26
7.21
7.19
7.17
7.03
7.01
6.99
6.97
6.45
6.41
6.02
6.00
1.87
1.85
Ph
Me
Me
2k
2.0
1.9
1.0
1.0
1.0
1.0
6.0
f1 (ppm)

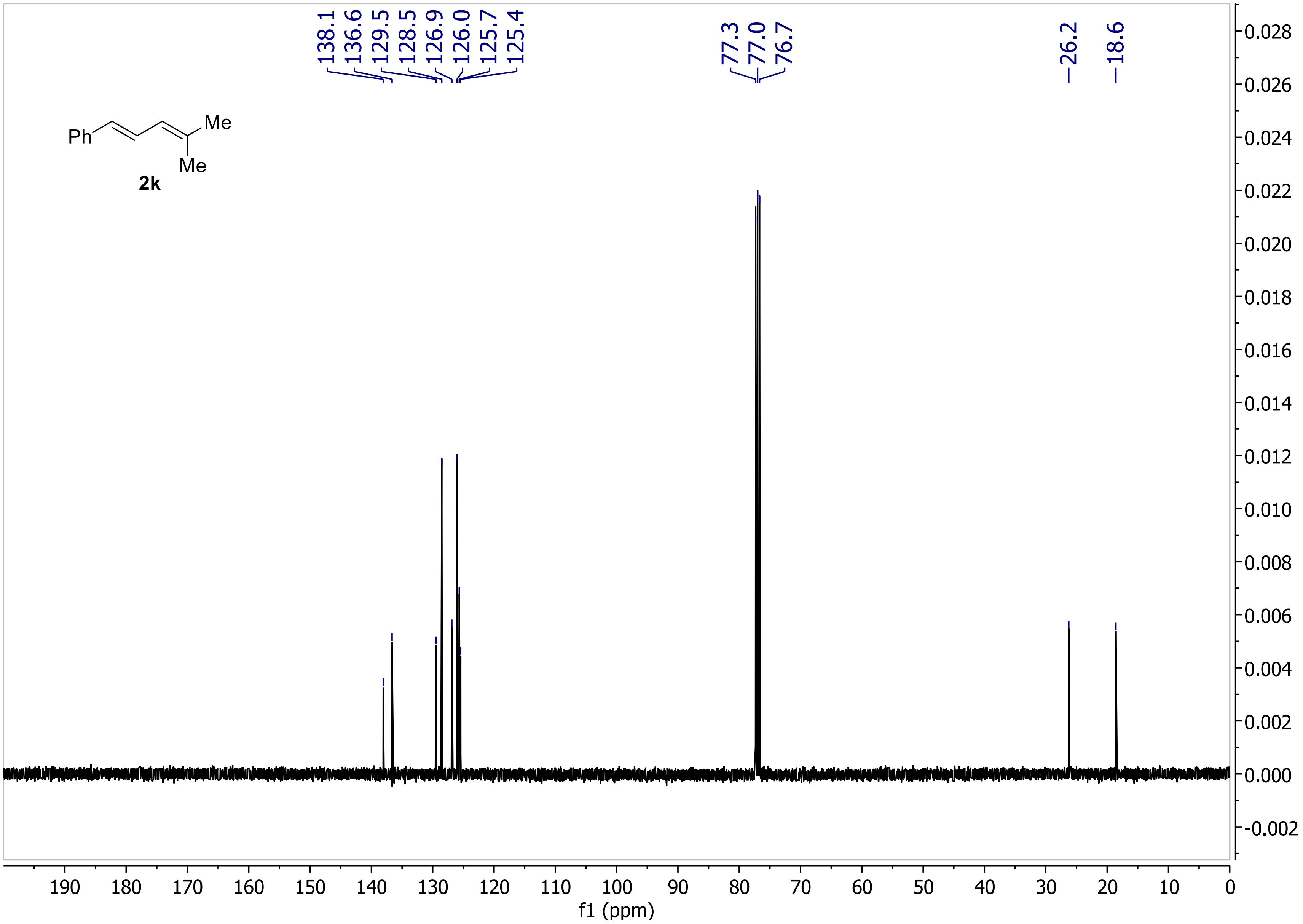
138.1
136.6
129.5
128.5
126.9
126.0
125.7
125.4
77.3
77.0
76.7
26.2
18.6
Ph
Me
Me
2k
f1 (ppm)

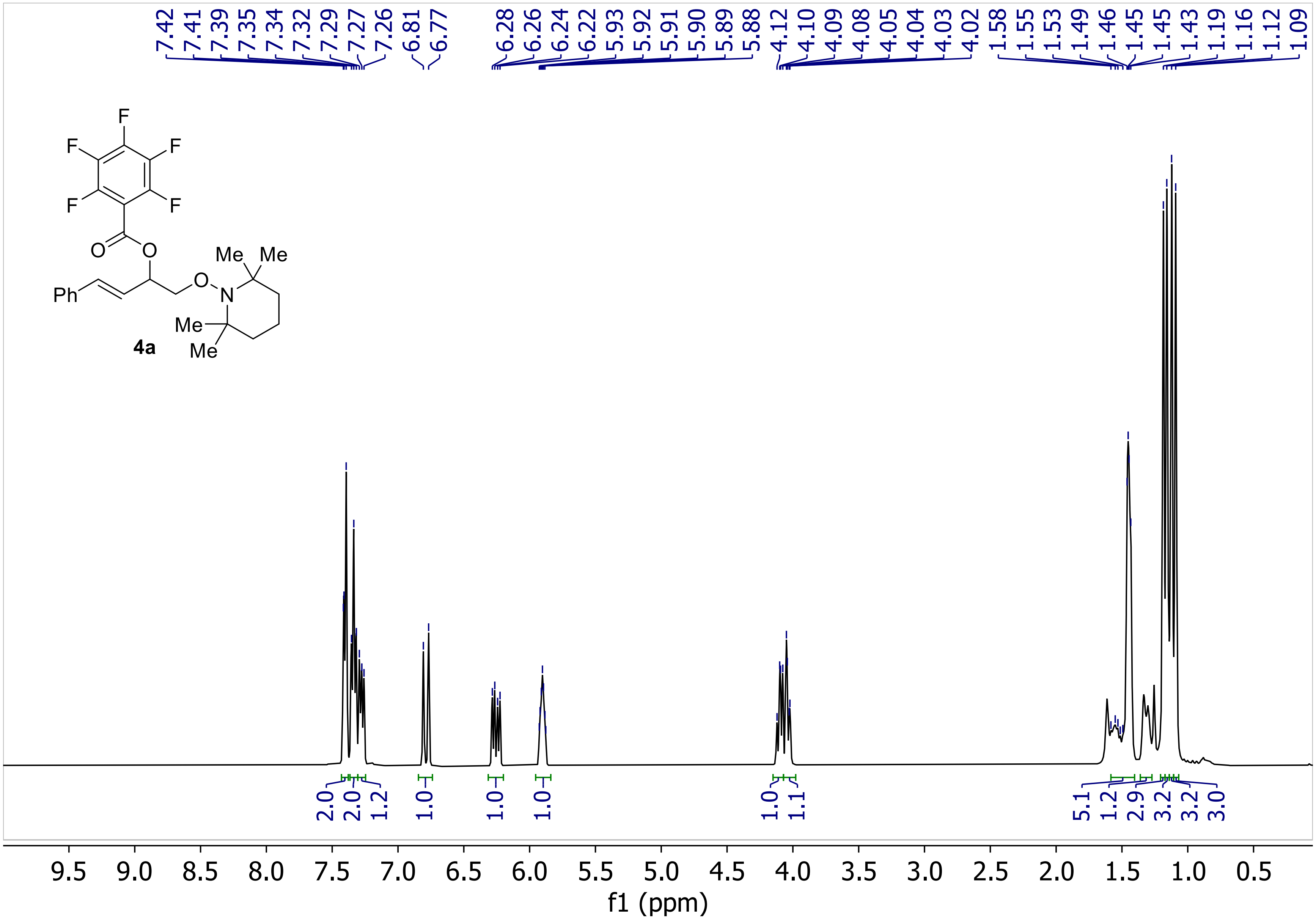
7.42
7.41
7.39
7.35
7.34
7.32
7.29
7.27
7.26
6.81
6.77
6.28
6.26
6.24
6.22
5.93
5.92
5.91
5.90
5.89
5.88
4.12
4.10
4.09
4.08
4.05
4.04
4.03
4.02
1.58
1.55
1.53
1.49
1.46
1.45
1.45
1.43
1.19
1.16
1.12
1.09
F
F
F
F
F
O
O
O
N
Me
Me
Me
Me
Ph
4a
2.0
2.0
1.2
1.0
1.0
1.0
1.0
1.1
5.1
1.2
2.9
3.2
3.2
3.0
9.5
9.0
8.5
8.0
7.5
7.0
6.5
6.0
5.5
5.0
4.5
4.0
3.5
3.0
2.5
2.0
1.5
1.0
0.5
f1 (ppm)

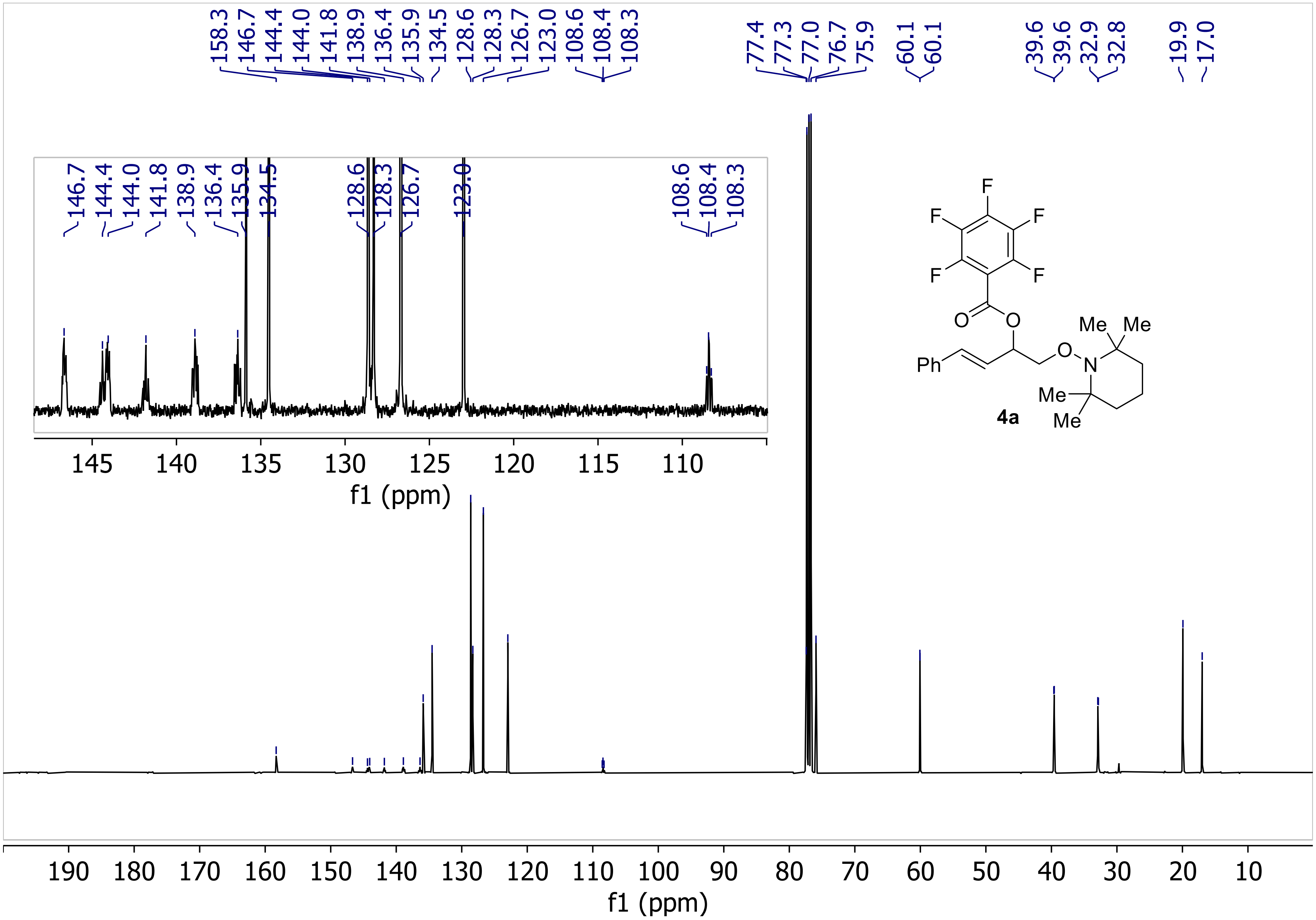
158.3
146.7
144.4
144.0
141.8
138.9
136.4
135.9
134.5
128.6
128.3
126.7
123.0
108.6
108.4
108.3
77.4
77.3
77.0
76.7
75.9
60.1
60.1
39.6
39.6
32.9
32.8
19.9
17.0
f1 (ppm)
4a
Ph
Me
N
O
F

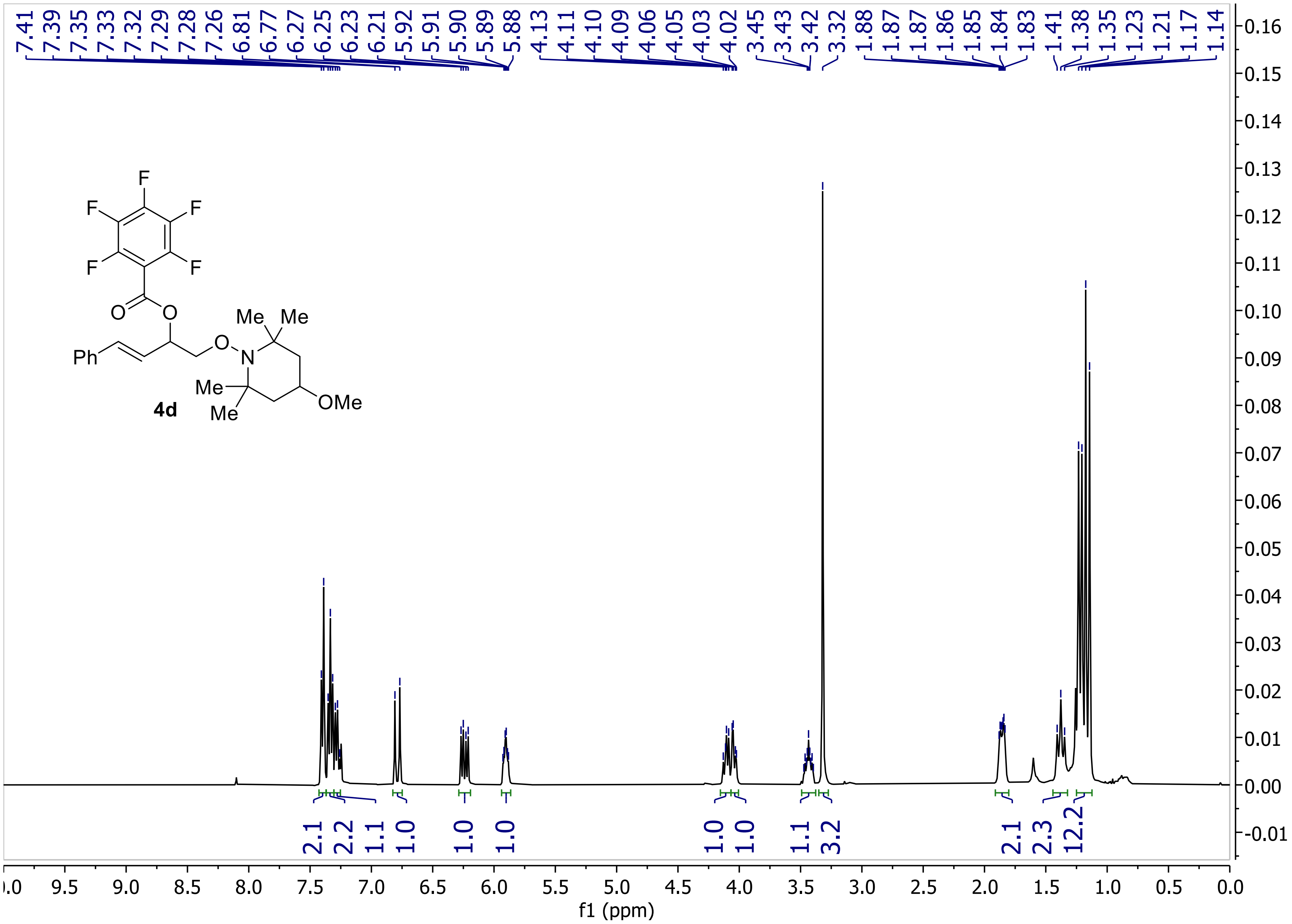

7.41 7.39 7.35 7.33 7.32 7.29 7.28 7.26 6.81 6.77 6.27 6.25 6.23 6.21 5.92 5.91 5.90 5.89 5.88 4.13 4.11 4.10 4.09 4.06 4.05 4.03 4.02 3.45 3.43 3.42 3.32 1.88 1.87 1.87 1.86 1.85 1.84 1.83 1.41 1.38 1.35 1.23 1.21 1.17 1.14
F
F
F
F
F
O
O
Me
Me
Ph
O
N
Me
Me
OMe
4d
2.1 2.2 1.1 1.0 1.0 1.0 1.0 1.0 1.1 3.2 2.1 2.3 12.2
f1 (ppm)

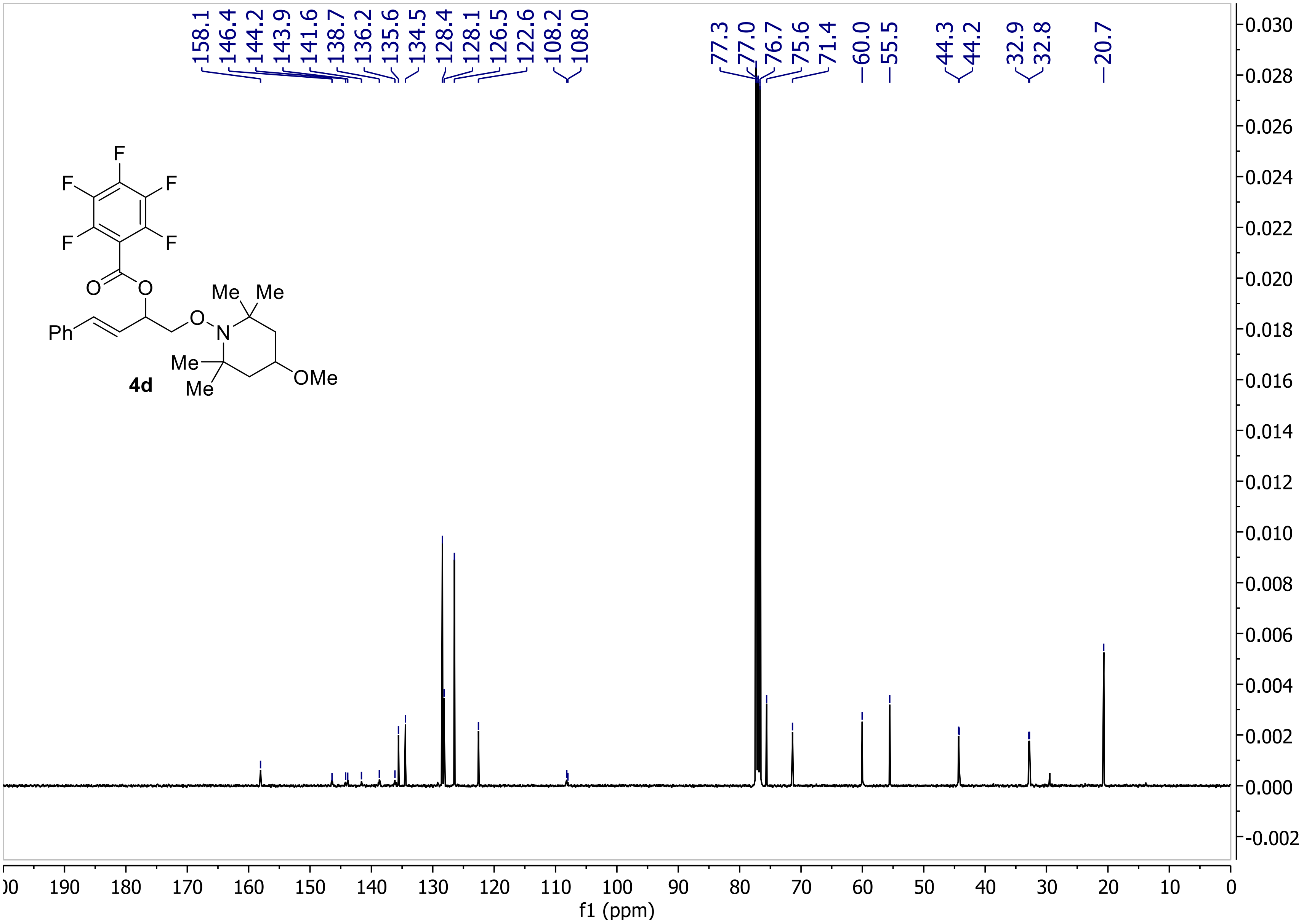
F
F
F
F
F
O
O
Me
Me
Ph
O
N
Me
Me
OMe
4d
158.1
146.4
144.2
143.9
141.6
138.7
136.2
135.6
134.5
128.4
128.1
126.5
122.6
108.2
108.0
77.3
77.0
76.7
75.6
71.4
60.0
55.5
44.3
44.2
32.9
32.8
20.7
f1 (ppm)

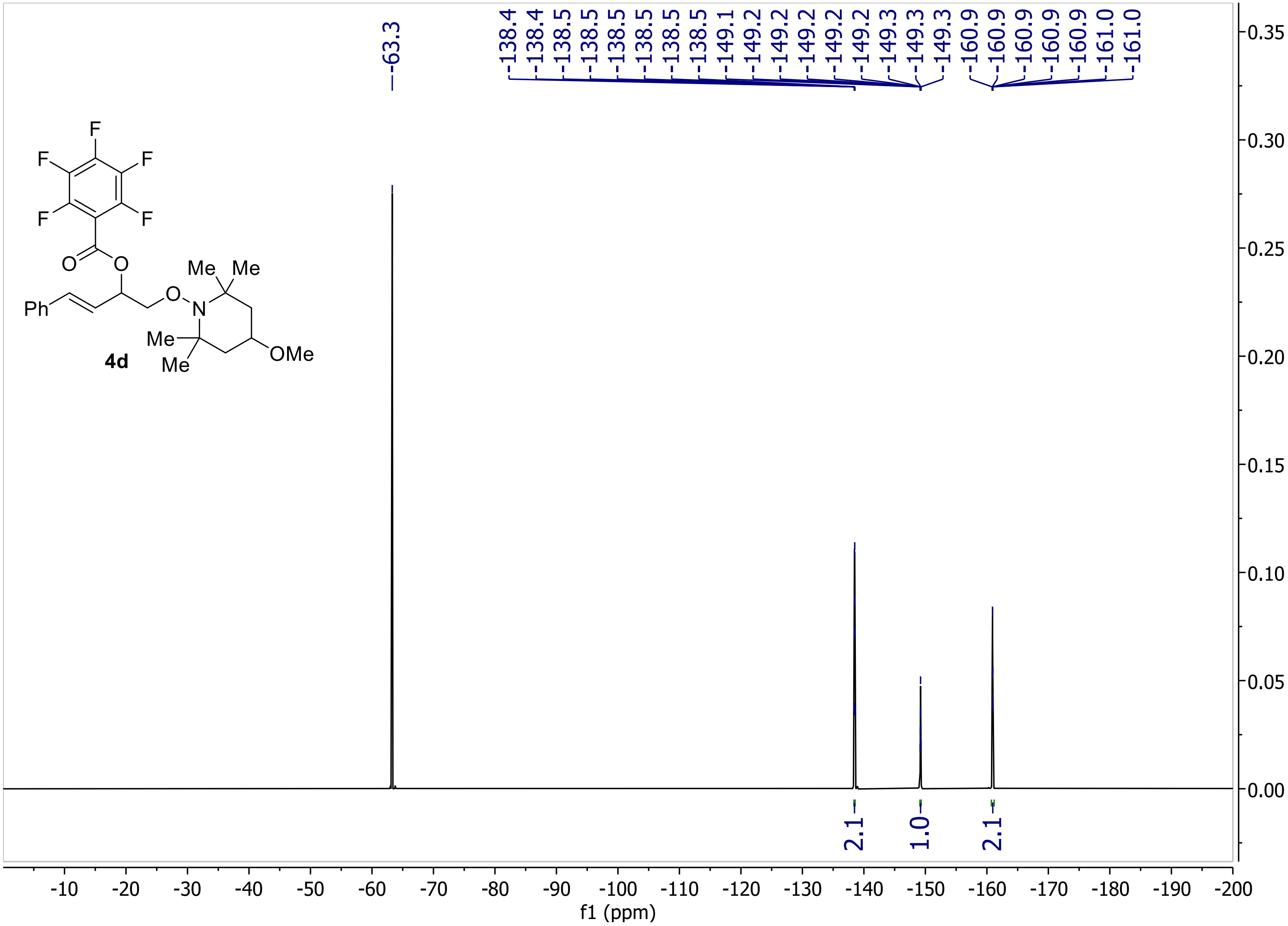
-63.3
-138.4
-138.4
-138.5
-138.5
-138.5
-138.5
-138.5
-138.5
-149.1
-149.2
-149.2
-149.2
-149.2
-149.2
-149.3
-149.3
-149.3
-160.9
-160.9
-160.9
-160.9
-160.9
-161.0
-161.0
2.1
1.0
2.1
0.35
0.30
0.25
0.20
0.15
0.10
0.05
0.00
-10
-20
-30
-40
-50
-60
-70
-80
-90
-100
-110
-120
-130
-140
-150
-160
-170
-180
-190
-200
f1 (ppm)

4d

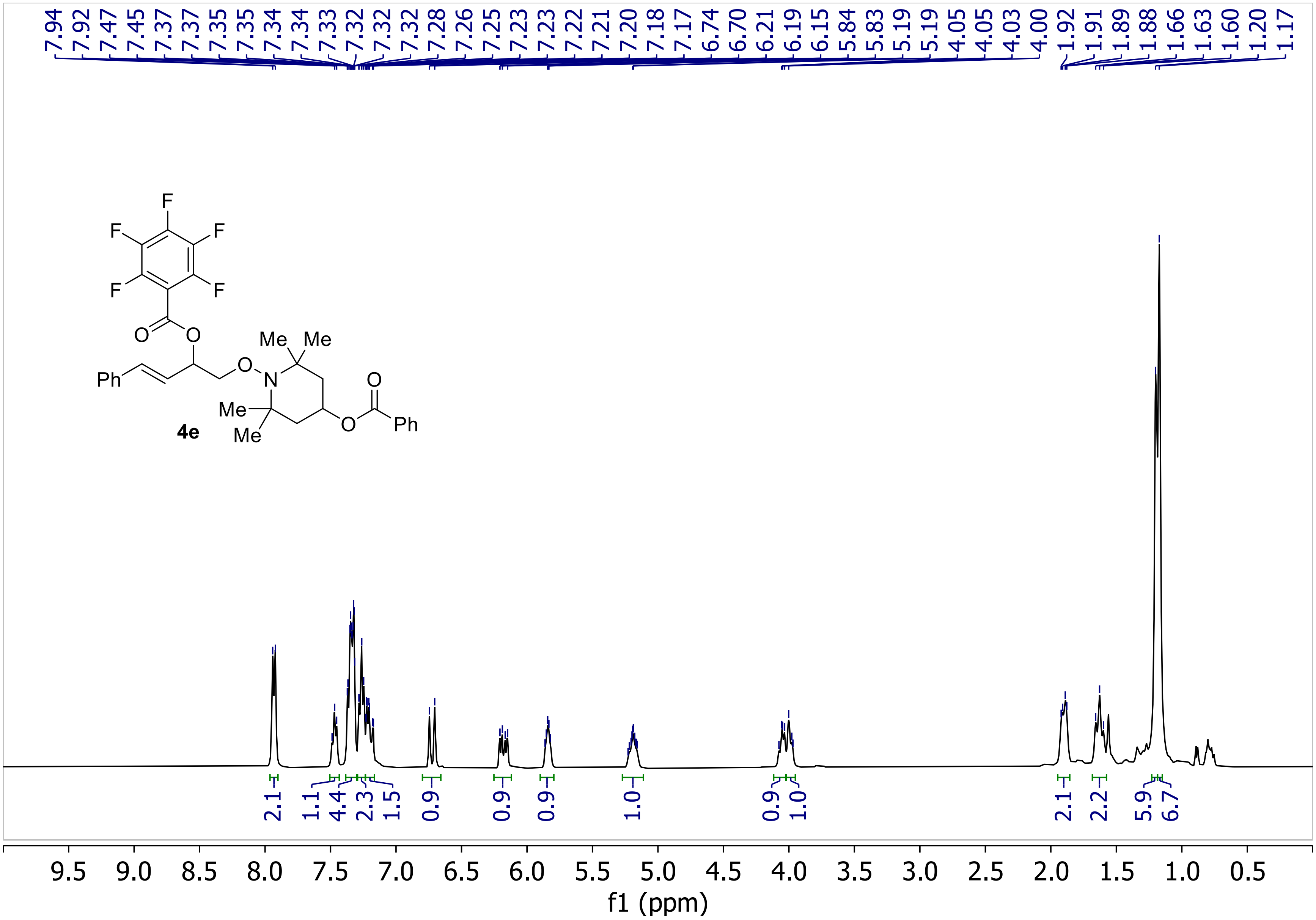
7.94
7.92
7.47
7.45
7.37
7.37
7.35
7.35
7.34
7.34
7.33
7.32
7.32
7.32
7.28
7.26
7.25
7.23
7.23
7.22
7.21
7.20
7.18
7.17
6.74
6.70
6.21
6.19
6.15
5.84
5.83
5.19
5.19
4.05
4.05
4.03
4.00
1.92
1.91
1.89
1.88
1.66
1.63
1.60
1.20
1.17
F
F
F
F
F
O
O
Me
Me
O
Ph
O
N
O
Me
Me
O
Ph
4e
2.1
1.1
4.4
2.3
1.5
0.9
0.9
0.9
1.0
0.9
1.0
2.1
2.2
5.9
6.7
9.5
9.0
8.5
8.0
7.5
7.0
6.5
6.0
5.5
5.0
4.5
4.0
3.5
3.0
2.5
2.0
1.5
1.0
0.5
f1 (ppm)

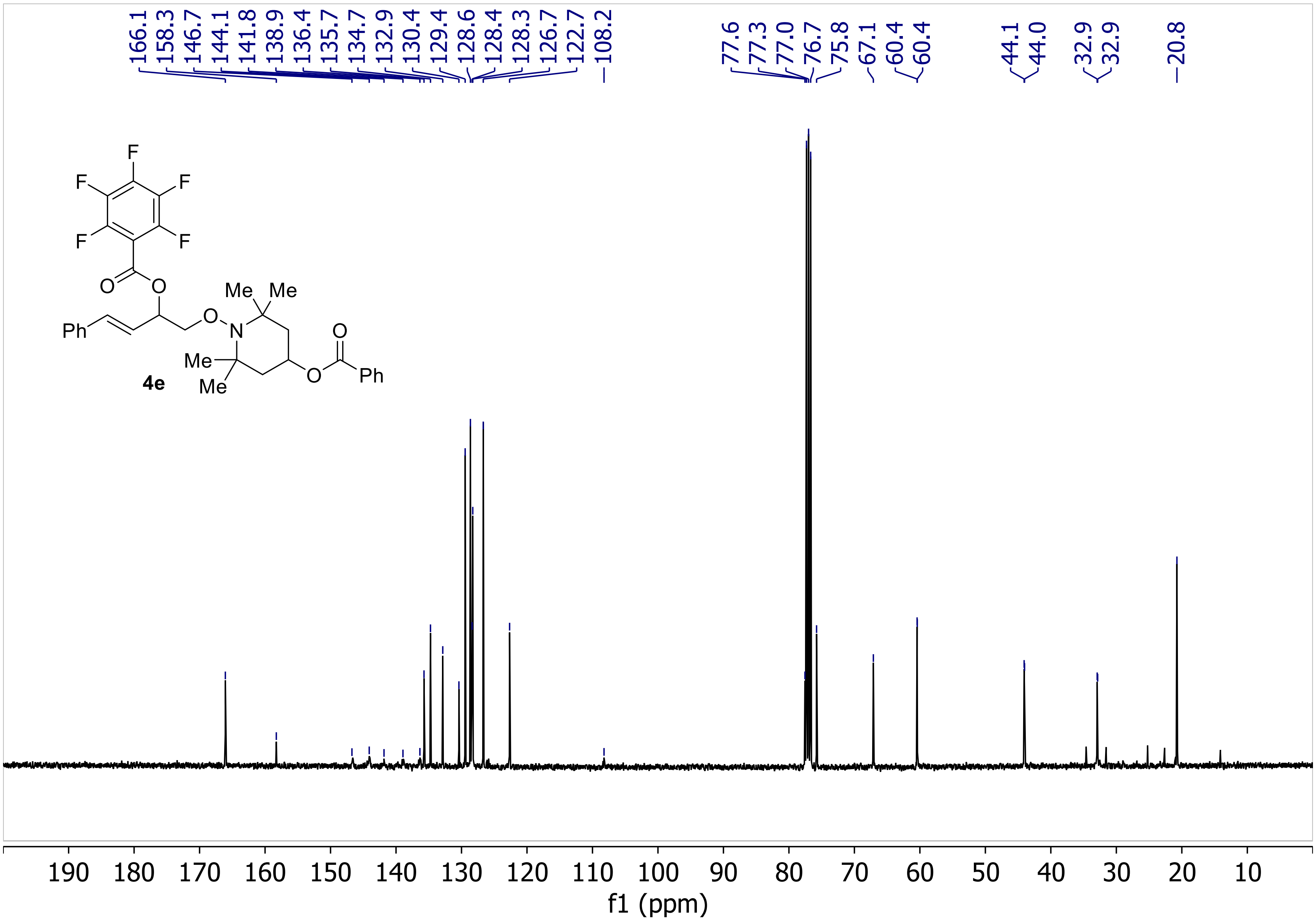
166.1
158.3
146.7
144.1
141.8
138.9
136.4
135.7
134.7
132.9
130.4
129.4
128.6
128.4
128.3
126.7
122.7
108.2
77.6
77.3
77.0
76.7
75.8
67.1
60.4
60.4
44.1
44.0
32.9
32.9
20.8
F
F
F
F
F
O
O
Me
Me
O
N
O
Ph
Me
Me
O
Ph
4e
190 180 170 160 150 140 130 120 110 100 90 80 70 60 50 40 30 20 10
f1 (ppm)

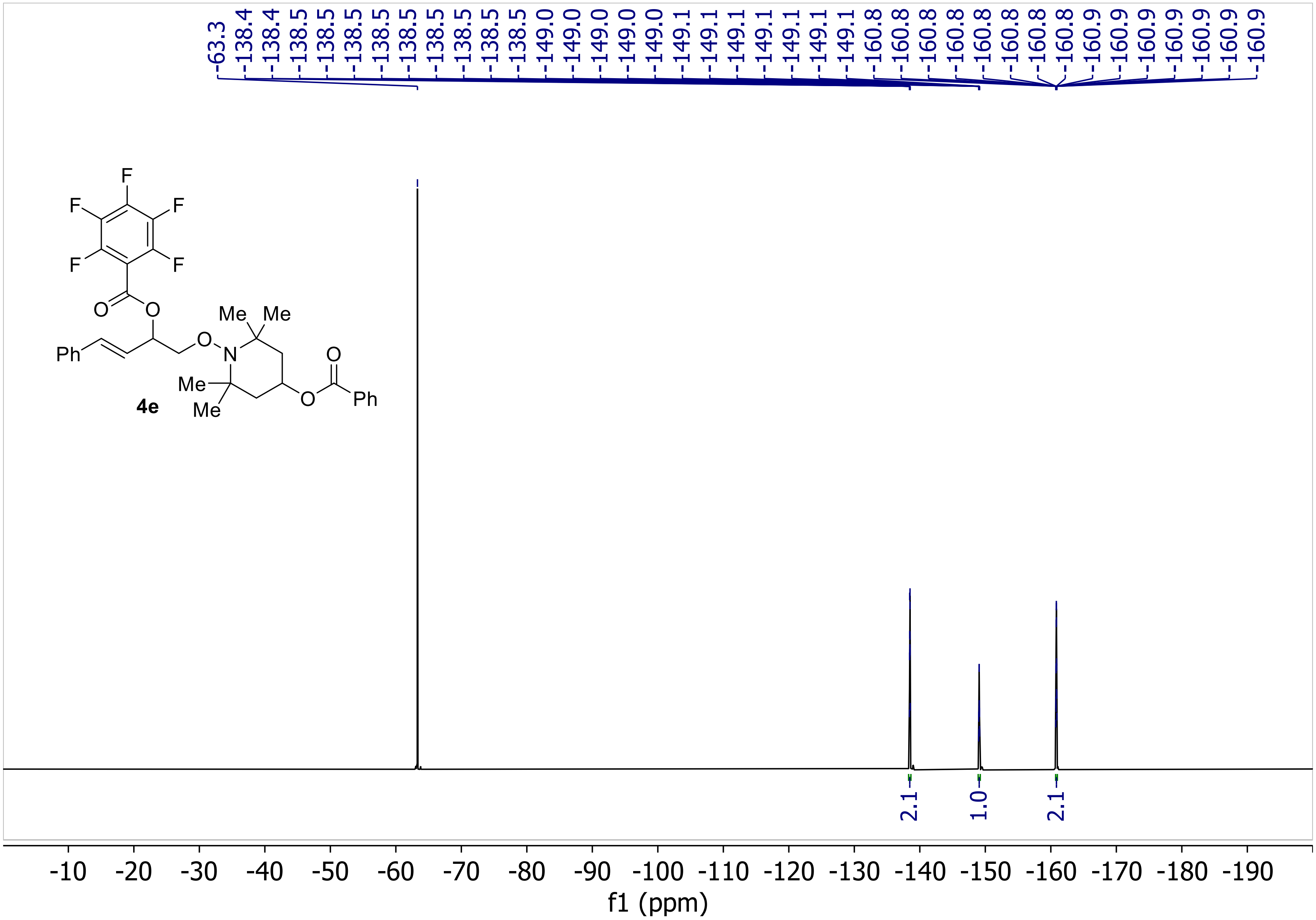

4e
-63.3
-138.4
-138.4
-138.5
-138.5
-138.5
-138.5
-138.5
-138.5
-138.5
-138.5
-138.5
-149.0
-149.0
-149.0
-149.0
-149.0
-149.1
-149.1
-149.1
-149.1
-149.1
-149.1
-149.1
-160.8
-160.8
-160.8
-160.8
-160.8
-160.8
-160.8
-160.8
-160.9
-160.9
-160.9
-160.9
-160.9
-160.9
-160.9
2.1
1.0
2.1
-10 -20 -30 -40 -50 -60 -70 -80 -90 -100 -110 -120 -130 -140 -150 -160 -170 -180 -190
f1 (ppm)

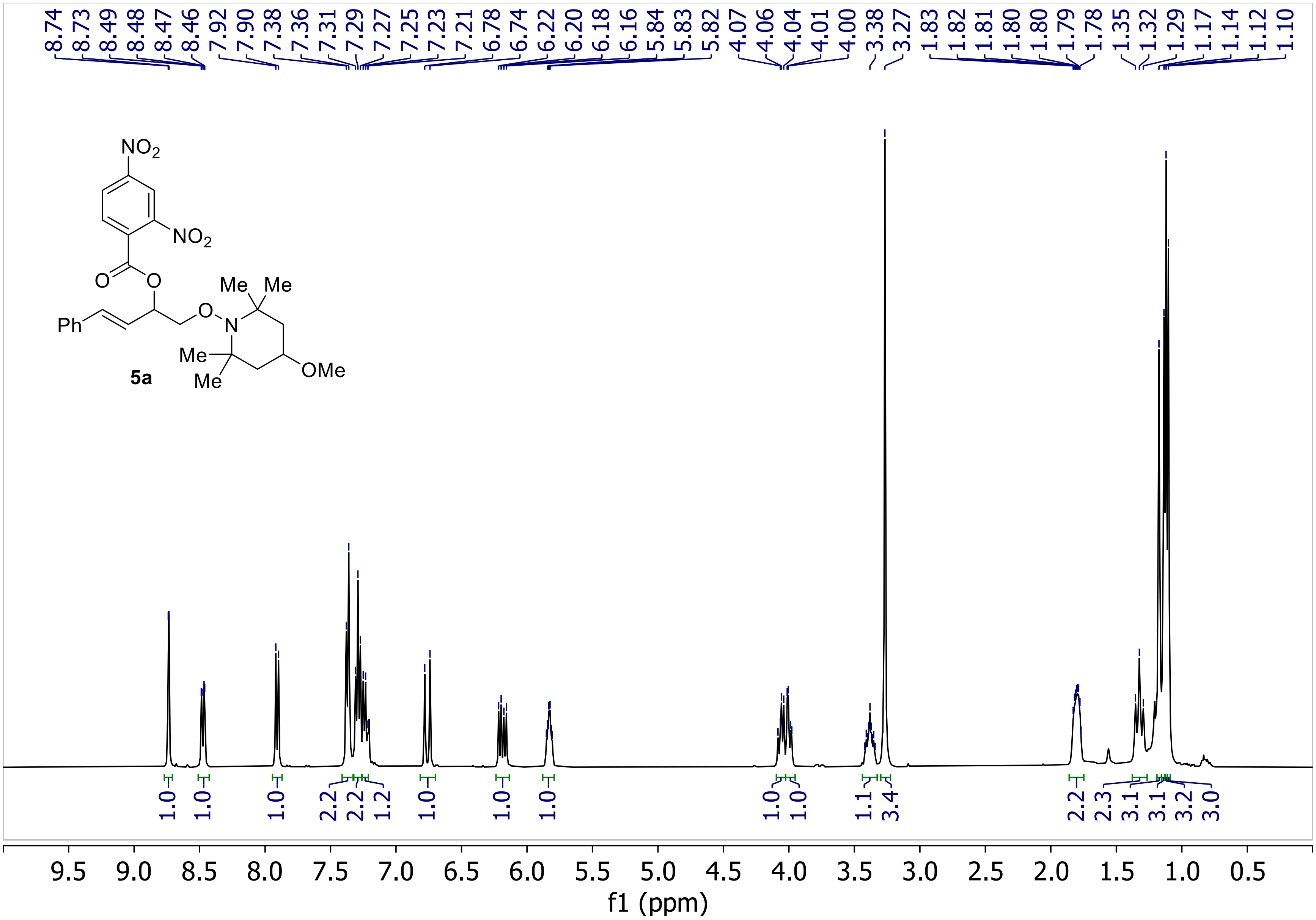
NO2
NO2
O
O
Ph
O
N
Me Me
Me
Me
OMe
5a
f1 (ppm)

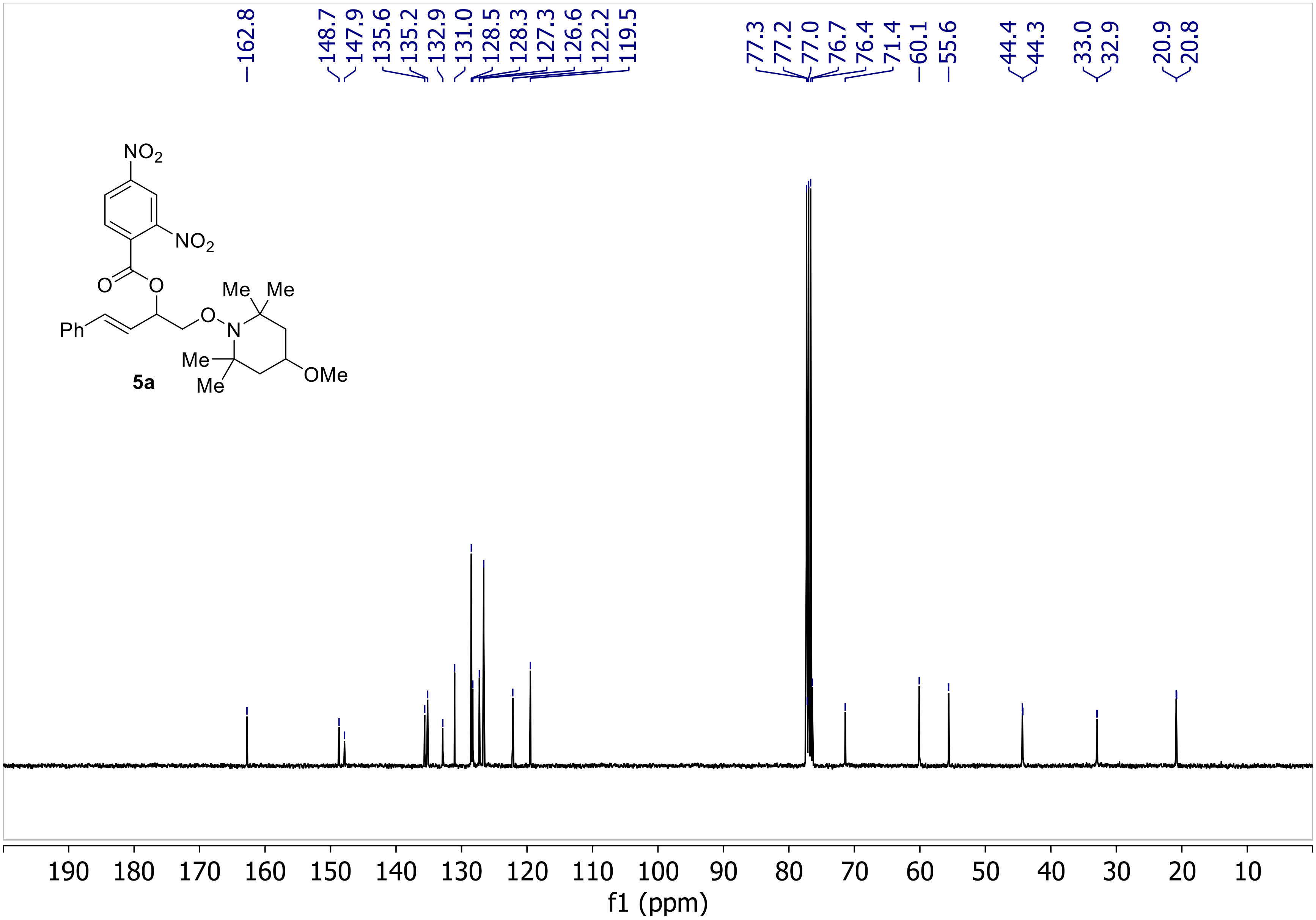
162.8
148.7
147.9
135.6
135.2
132.9
131.0
128.5
128.3
127.3
126.6
122.2
119.5
77.3
77.2
77.0
76.7
76.4
71.4
60.1
55.6
44.4
44.3
33.0
32.9
20.9
20.8
NO_2
NO_2
O
O
Ph
O
N
Me
Me
Me
Me
OMe
5a
190 180 170 160 150 140 130 120 110 100 90 80 70 60 50 40 30 20 10
f1 (ppm)

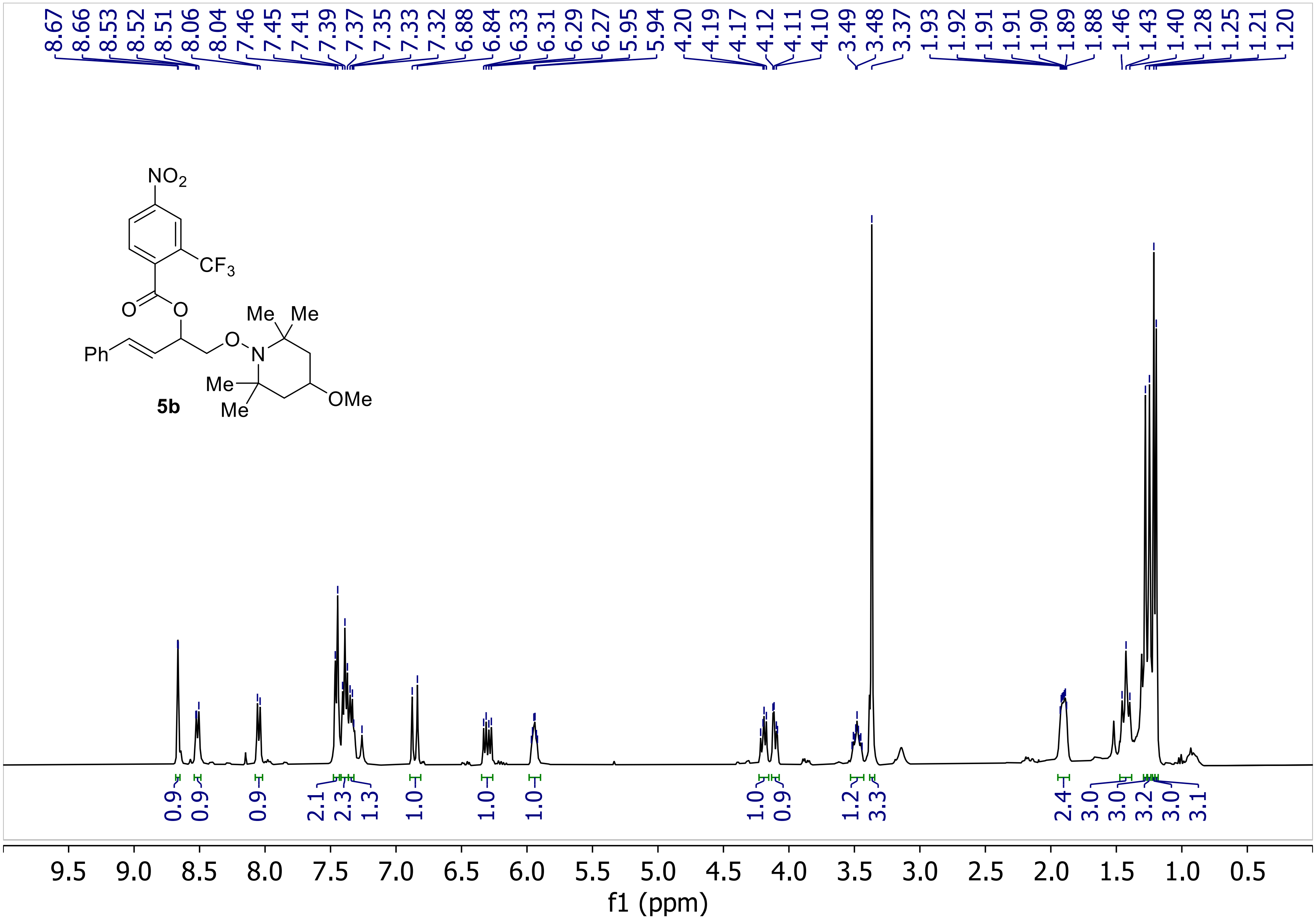
NO2
CF3
O
O
Ph
O
N
Me
Me
Me
Me
OMe
5b
f1 (ppm)

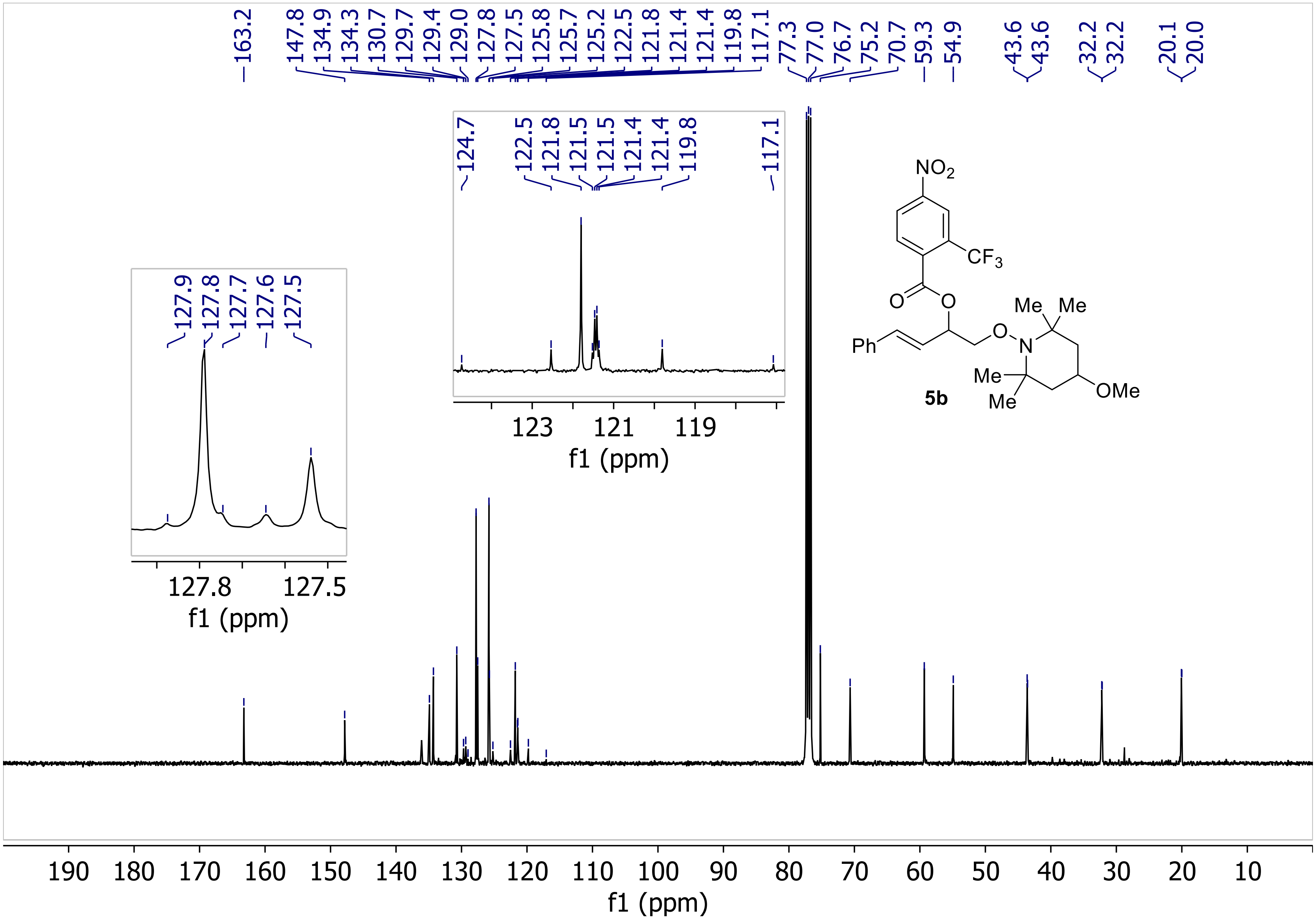
163.2
147.8
134.9
134.3
130.7
129.7
129.4
129.0
127.8
127.5
125.8
125.7
125.2
122.5
121.8
121.4
121.4
119.8
117.1
77.3
77.0
76.7
75.2
70.7
59.3
54.9
43.6
43.6
32.2
32.2
20.1
20.0
127.9
127.8
127.7
127.6
127.5
124.7
122.5
121.8
121.5
121.5
121.4
121.4
119.8
117.1
NO2
CF3
O
O
Me
Me
Ph
O
N
Me
Me
OMe
5b
f1 (ppm)

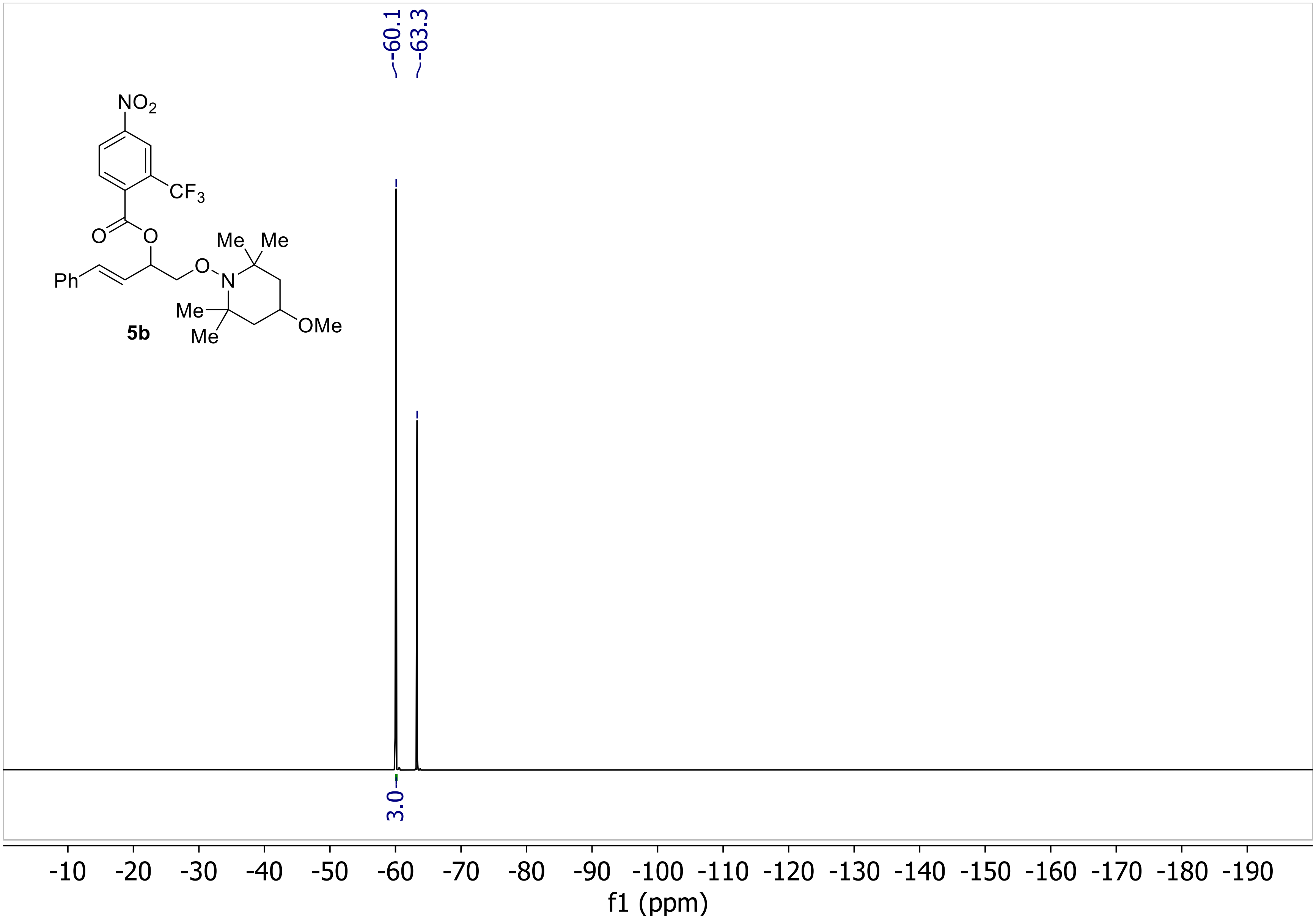
-60.1
-63.3
NO2
CF3
O
O
Me
Me
O
N
Ph
Me
Me
OMe
5b
3.0
-10
-20
-30
-40
-50
-60
-70
-80
-90
-100
-110
-120
-130
-140
-150
-160
-170
-180
-190
f1 (ppm)

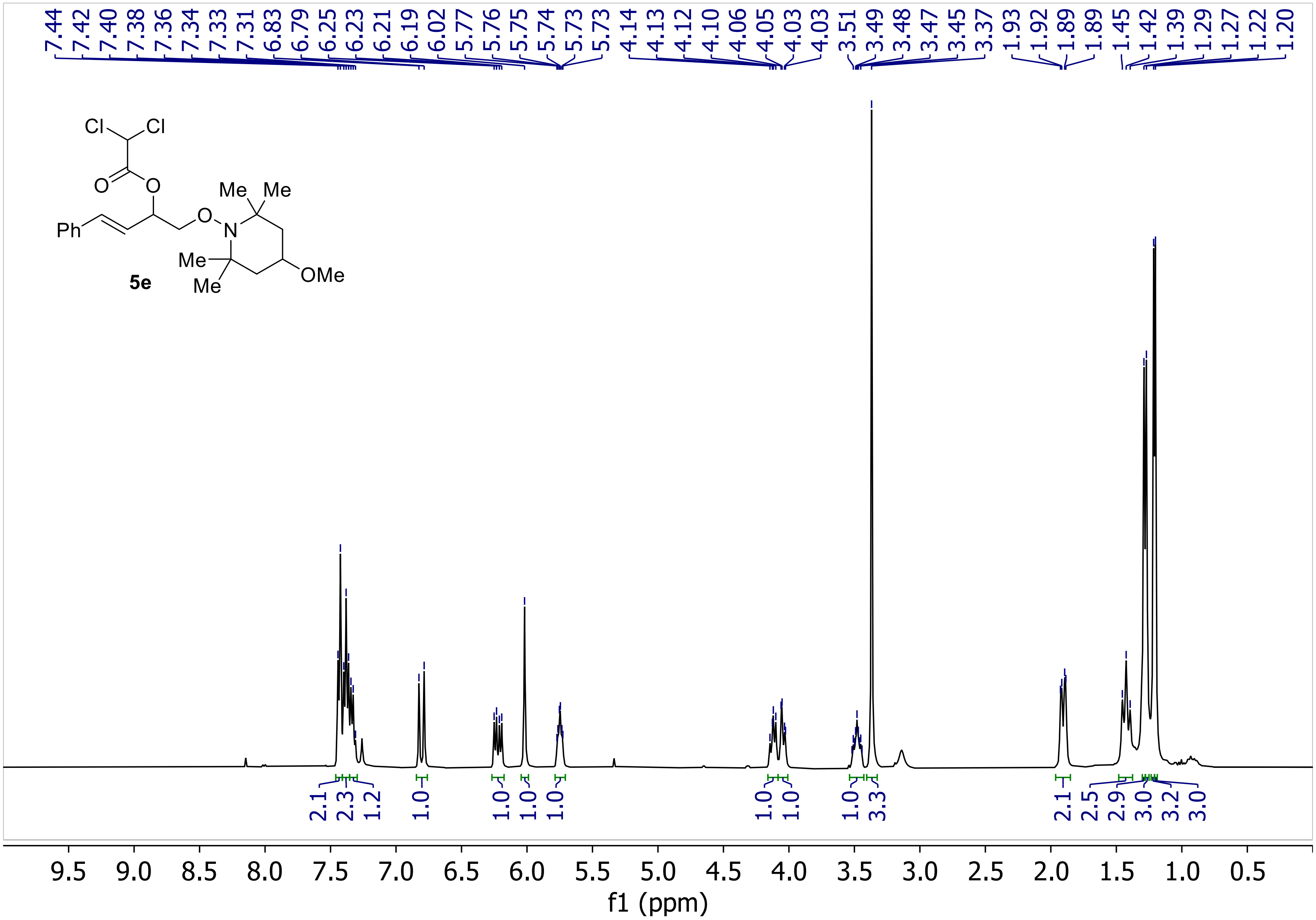
7.44 7.42 7.40 7.38 7.36 7.34 7.33 7.31 6.83 6.79 6.25 6.23 6.21 6.19 6.02 5.77 5.76 5.75 5.74 5.73 5.73 4.14 4.13 4.12 4.10 4.06 4.05 4.03 4.03 3.51 3.49 3.48 3.47 3.45 3.37 1.93 1.92 1.89 1.89 1.45 1.42 1.39 1.29 1.27 1.22 1.20
Cl
Cl
O
O
Ph
O
N
Me
Me
Me
Me
OMe
5e
2.1 2.3 1.2 1.0 1.0 1.0 1.0 1.0 1.0 1.0 3.3 2.1 2.5 2.9 3.0 3.2 3.0
9.5 9.0 8.5 8.0 7.5 7.0 6.5 6.0 5.5 5.0 4.5 4.0 3.5 3.0 2.5 2.0 1.5 1.0 0.5
f1 (ppm)

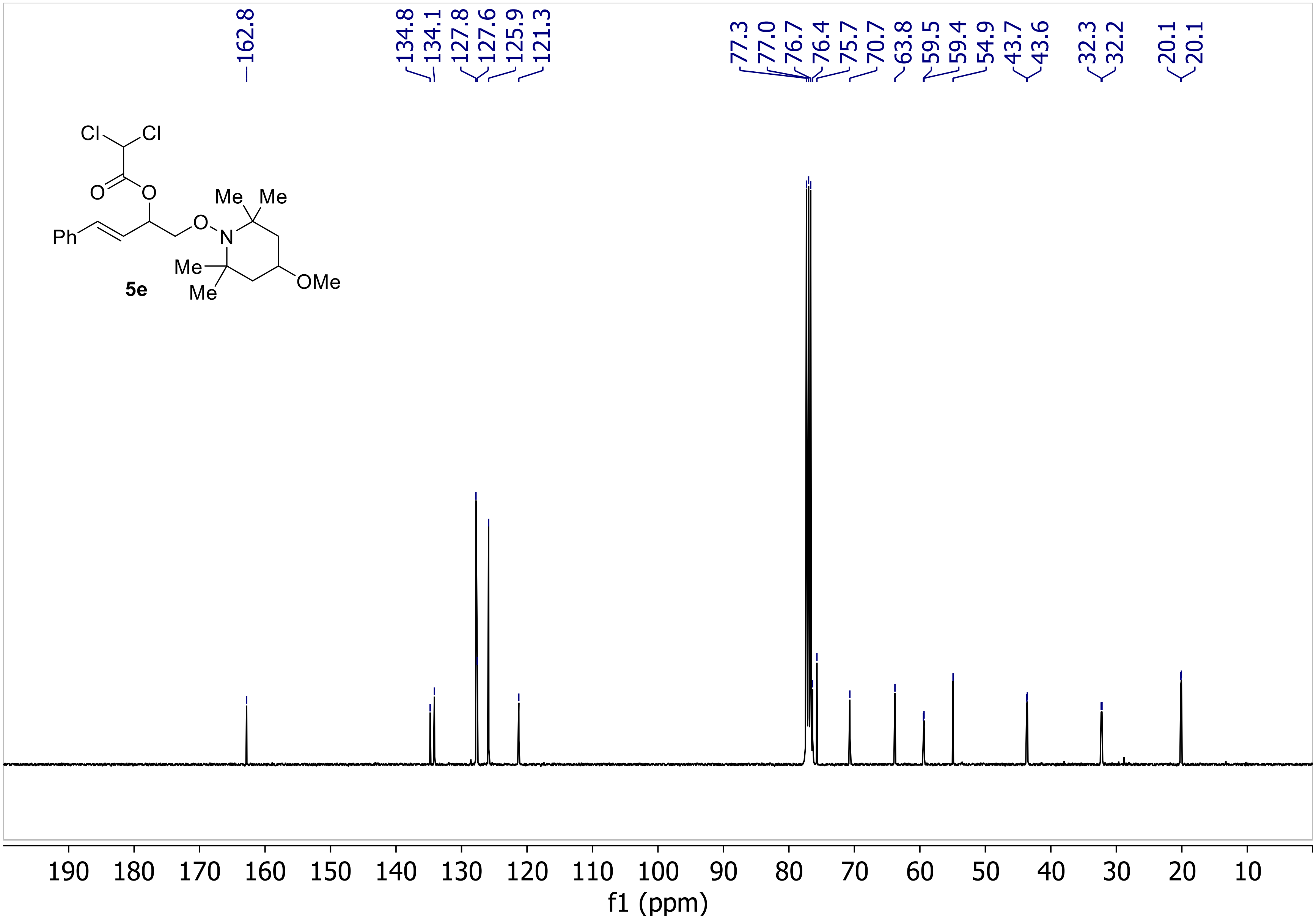

162.8
134.8
134.1
127.8
127.6
125.9
121.3
77.3
77.0
76.7
76.4
75.7
70.7
63.8
59.5
59.4
54.9
43.7
43.6
32.3
32.2
20.1
20.1
Cl
Cl
O
O
Me
Me
Ph
O
N
Me
Me
OMe
5e
190 180 170 160 150 140 130 120 110 100 90 80 70 60 50 40 30 20 10
f1 (ppm)

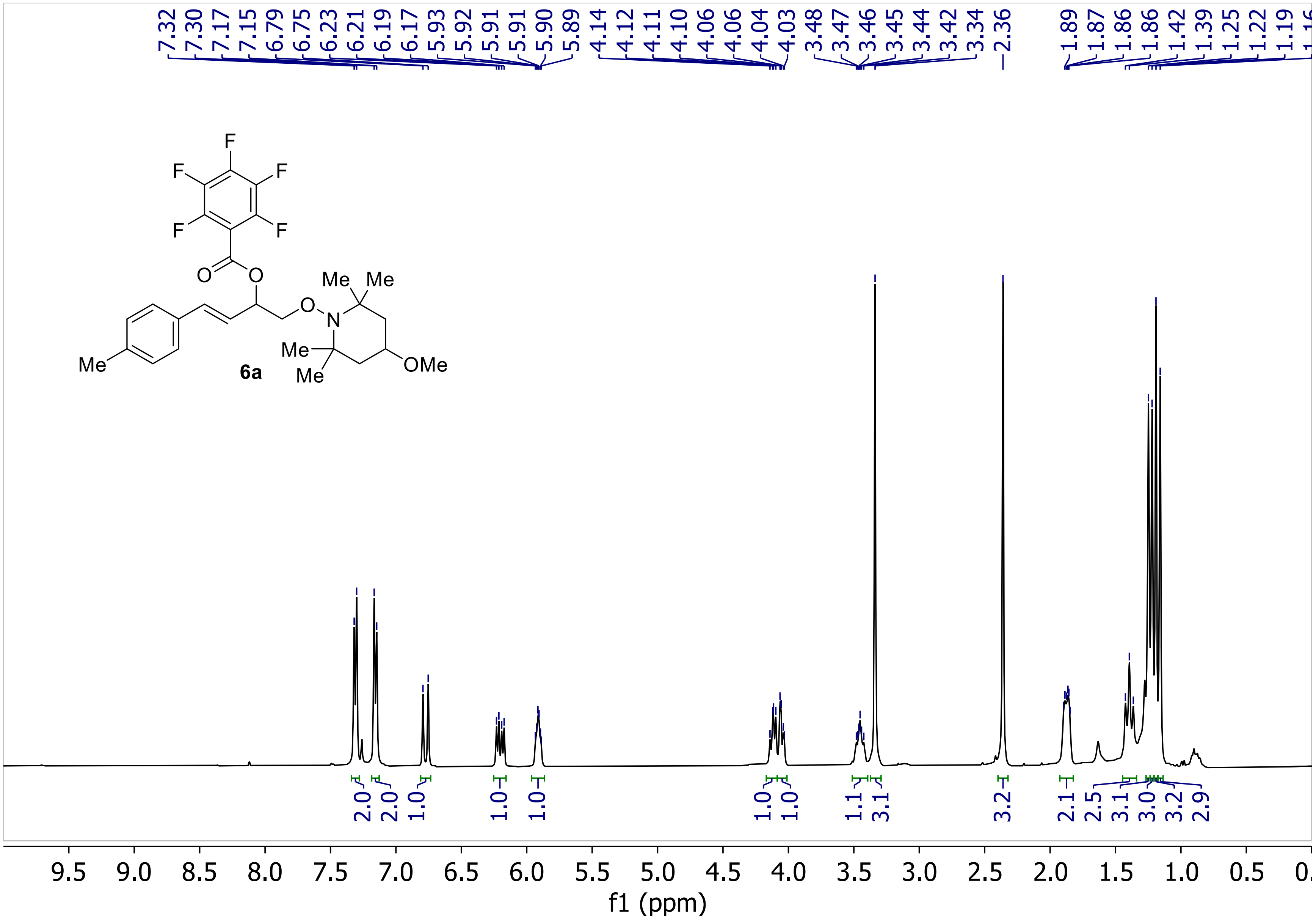
7.32
7.30
7.17
7.15
6.79
6.75
6.23
6.21
6.19
6.17
5.93
5.92
5.91
5.91
5.90
5.89
4.14
4.12
4.11
4.10
4.06
4.06
4.04
4.03
3.48
3.47
3.46
3.45
3.44
3.42
3.34
2.36
1.89
1.87
1.86
1.86
1.42
1.39
1.25
1.22
1.19
F
F
F
F
F
O
O
O
N
Me
Me
Me
Me
Me
OMe
6a
2.0
2.0
1.0
1.0
1.0
1.0
1.0
1.1
3.1
3.2
2.1
2.5
3.1
3.0
3.2
2.9
9.5
9.0
8.5
8.0
7.5
7.0
6.5
6.0
5.5
5.0
4.5
4.0
3.5
3.0
2.5
2.0
1.5
1.0
0.5
f1 (ppm)

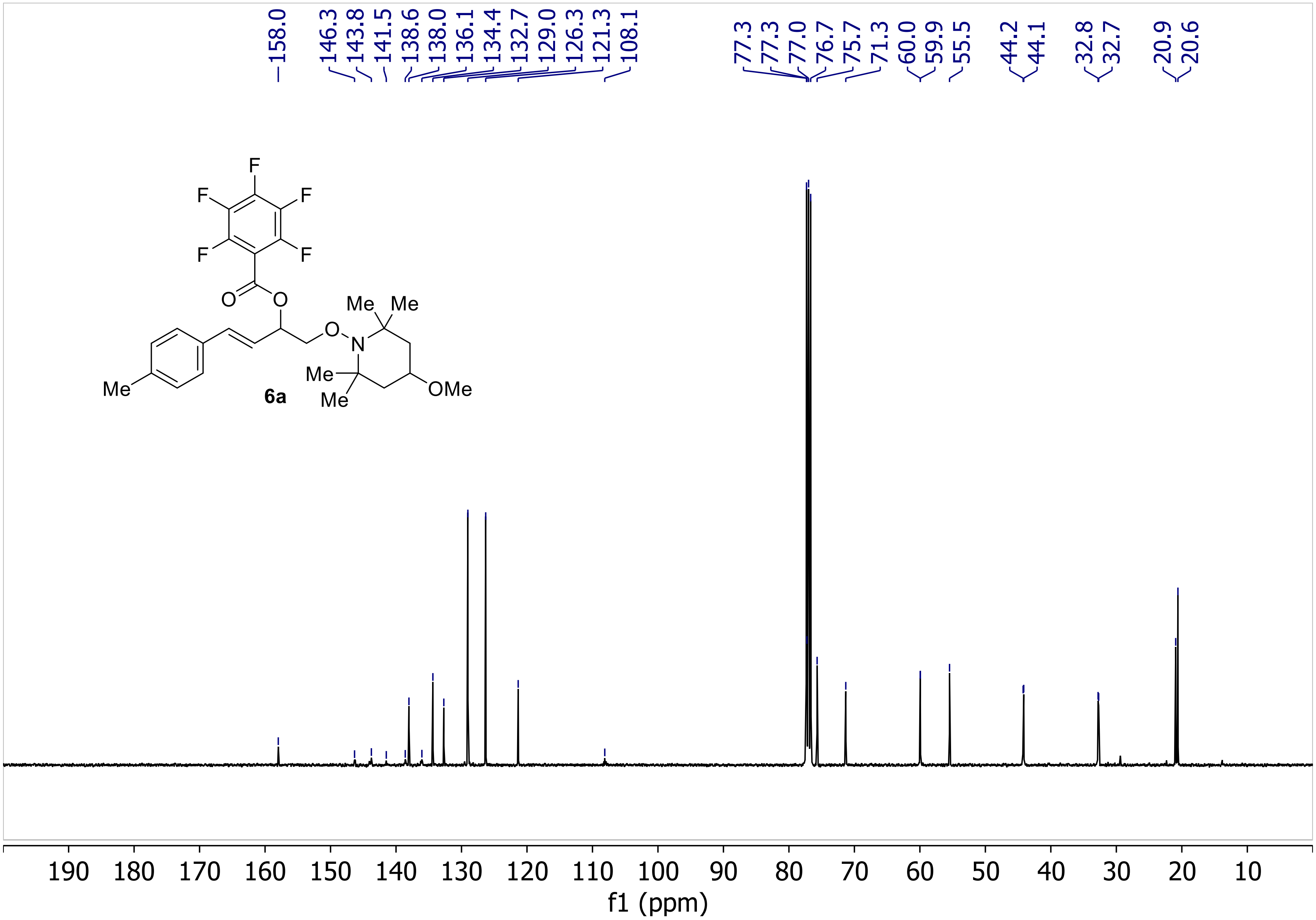
158.0
146.3
143.8
141.5
138.6
138.0
136.1
134.4
132.7
129.0
126.3
121.3
108.1
77.3
77.3
77.0
76.7
75.7
71.3
60.0
59.9
55.5
44.2
44.1
32.8
32.7
20.9
20.6
F
F
F
F
F
O
O
O
N
Me
Me
Me
Me
Me
OMe
6a
190
180
170
160
150
140
130
120
110
100
90
80
70
60
50
40
30
20
10
f1 (ppm)

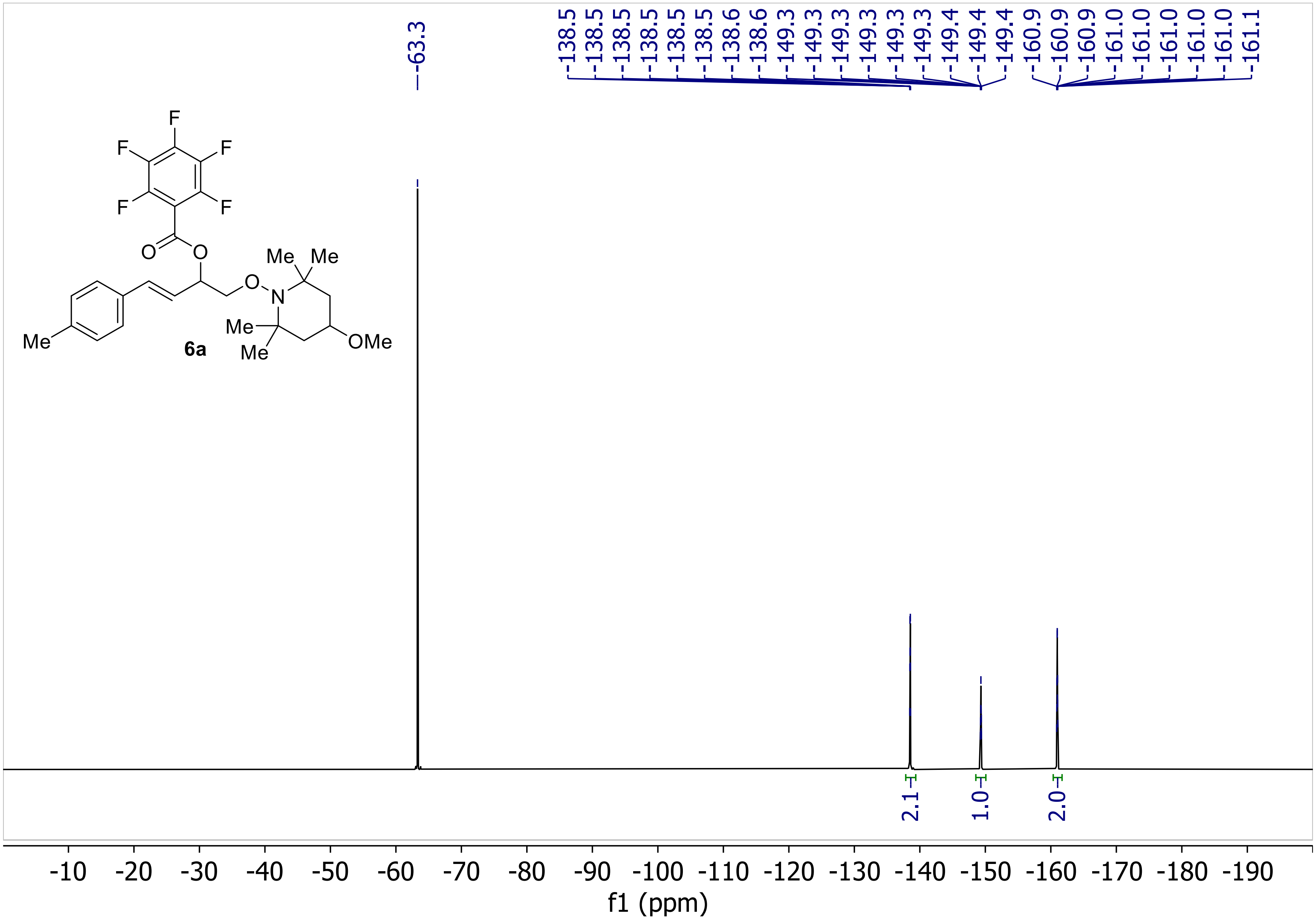
F
F
F
F
F
O
O
O
N
Me
Me
Me
Me
Me
OMe
6a
-63.3
-138.5
-138.5
-138.5
-138.5
-138.5
-138.5
-138.6
-138.6
-149.3
-149.3
-149.3
-149.3
-149.3
-149.3
-149.4
-149.4
-149.4
-160.9
-160.9
-160.9
-161.0
-161.0
-161.0
-161.0
-161.0
-161.1
2.1
1.0
2.0
-10
-20
-30
-40
-50
-60
-70
-80
-90
-100
-110
-120
-130
-140
-150
-160
-170
-180
-190
f1 (ppm)

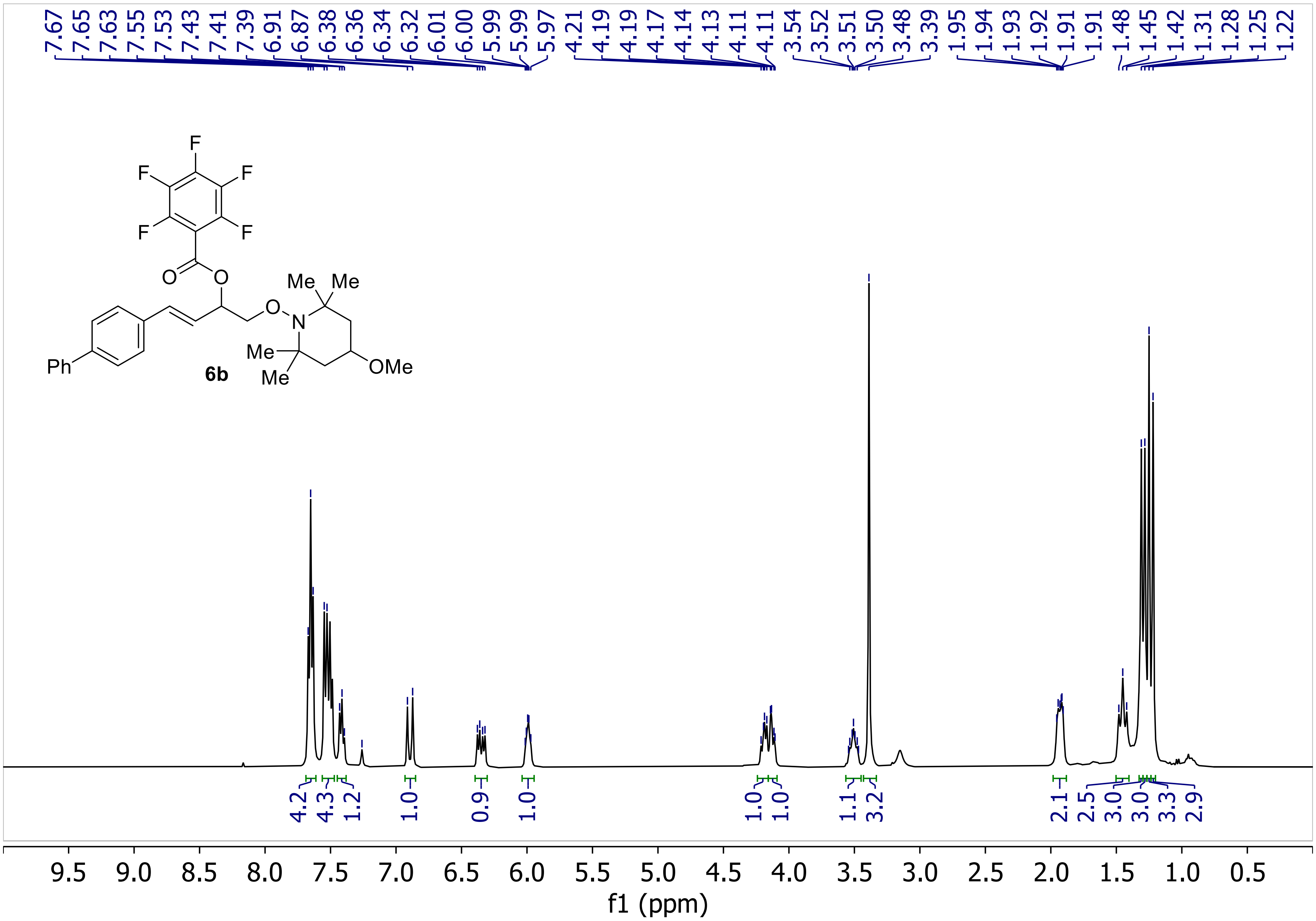
7.67 7.65 7.63 7.55 7.53 7.43 7.41 7.39 6.91 6.87 6.38 6.36 6.34 6.32 6.01 6.00 5.99 5.99 5.97 4.21 4.19 4.19 4.17 4.14 4.13 4.11 4.11 3.54 3.52 3.51 3.50 3.48 3.39 1.95 1.94 1.93 1.92 1.91 1.91 1.48 1.45 1.42 1.31 1.28 1.25 1.22
F
F
F
F
F
O
O
O
N
Me
Me
Me
Me
OMe
Ph
6b
4.2 4.3 1.2 1.0 0.9 1.0 1.0 1.0 1.1 3.2 2.1 2.5 3.0 3.0 3.3 2.9
9.5 9.0 8.5 8.0 7.5 7.0 6.5 6.0 5.5 5.0 4.5 4.0 3.5 3.0 2.5 2.0 1.5 1.0 0.5
f1 (ppm)

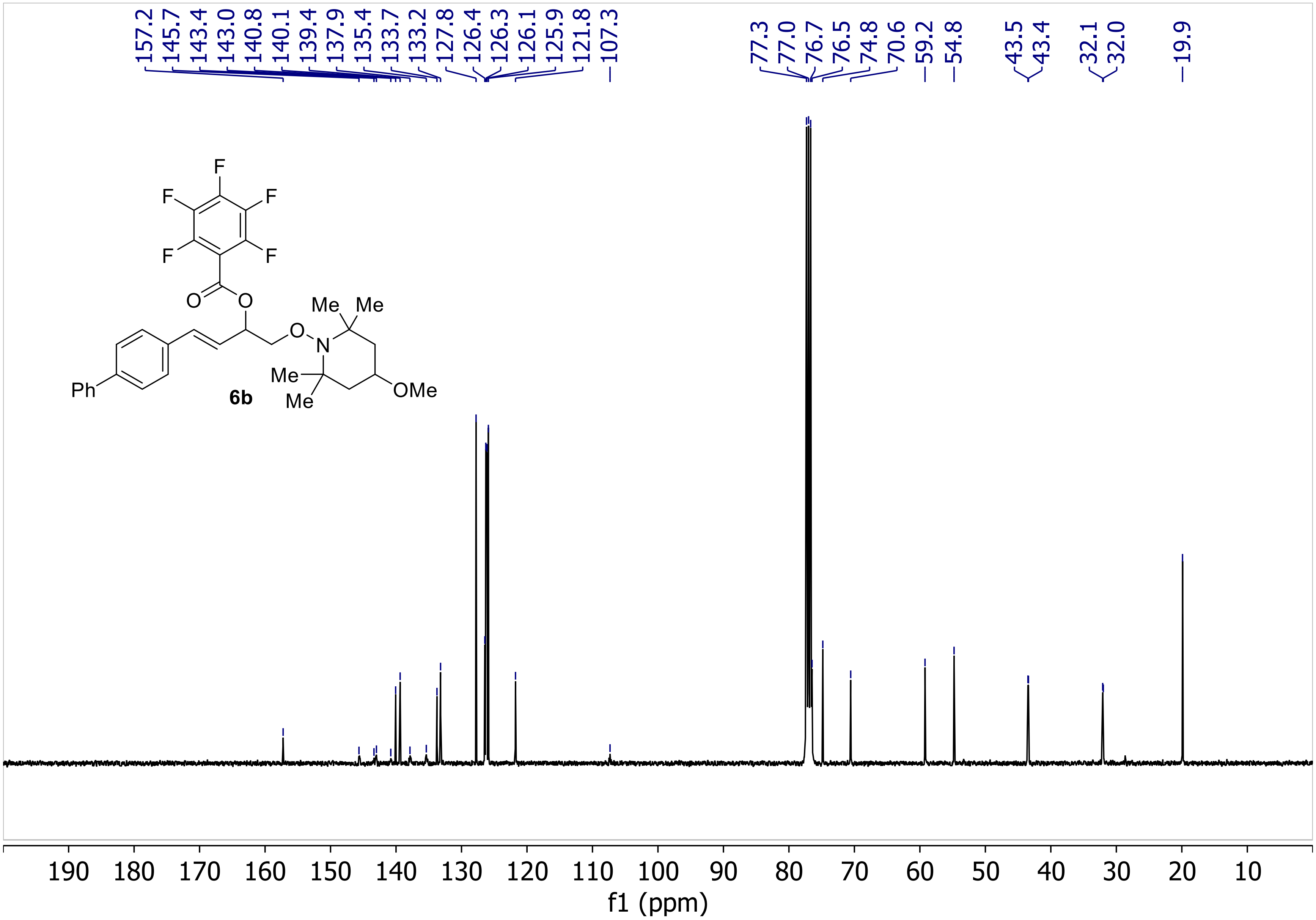
157.2
145.7
143.4
143.0
140.8
140.1
139.4
137.9
135.4
133.7
133.2
127.8
126.4
126.3
126.1
125.9
121.8
107.3
77.3
77.0
76.7
76.5
74.8
70.6
59.2
54.8
43.5
43.4
32.1
32.0
19.9
F
F
F
F
F
O
O
Me
Me
O
N
Ph
Me
Me
OMe
6b
190 180 170 160 150 140 130 120 110 100 90 80 70 60 50 40 30 20 10
f1 (ppm)

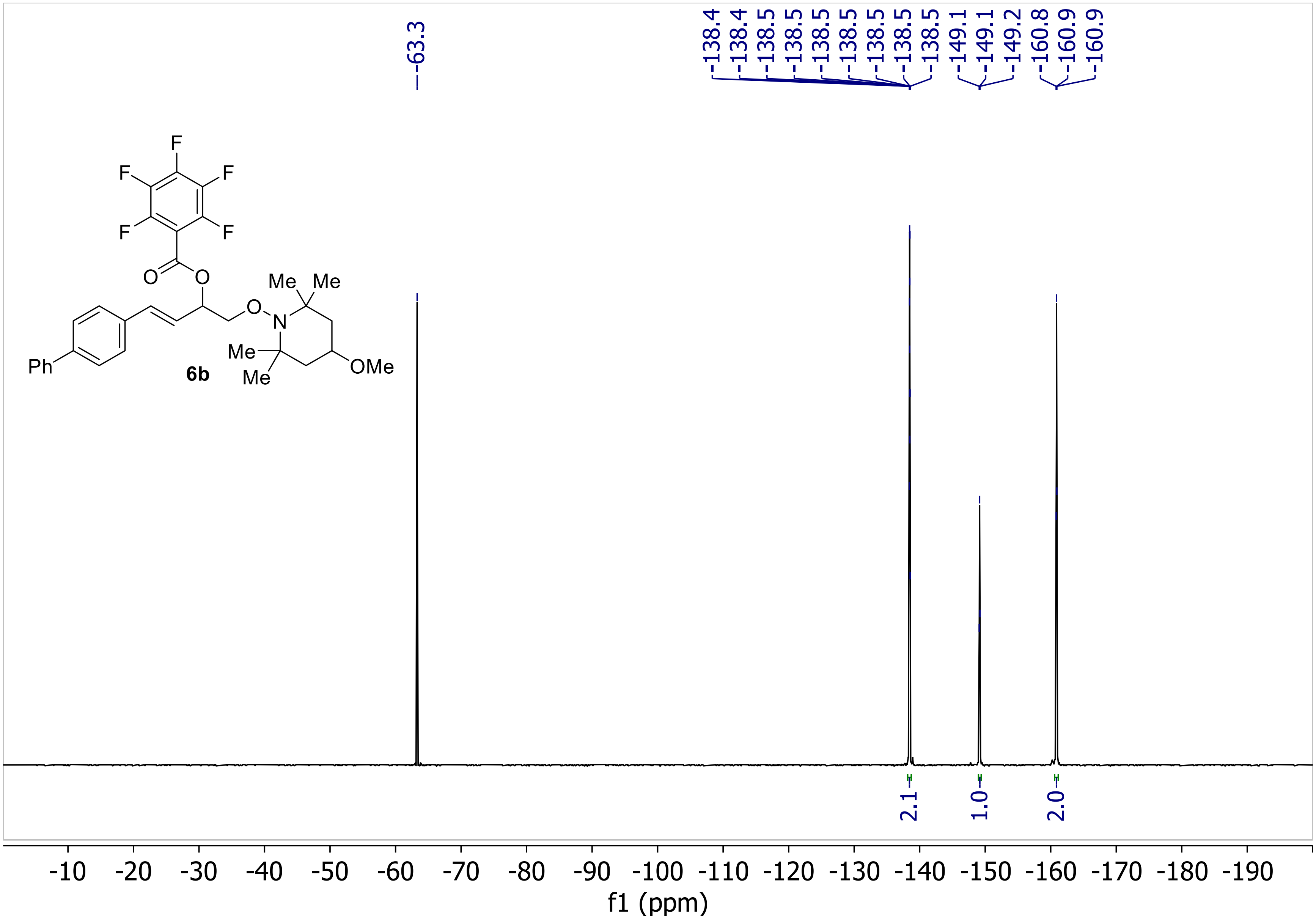
6b
Ph
OMe
Me
F
O
N
-63.3
-138.4
-138.4
-138.5
-138.5
-138.5
-138.5
-138.5
-138.5
-138.5
-149.1
-149.1
-149.2
-160.8
-160.9
-160.9
2.1
1.0
2.0
-10 -20 -30 -40 -50 -60 -70 -80 -90 -100 -110 -120 -130 -140 -150 -160 -170 -180 -190
f1 (ppm)

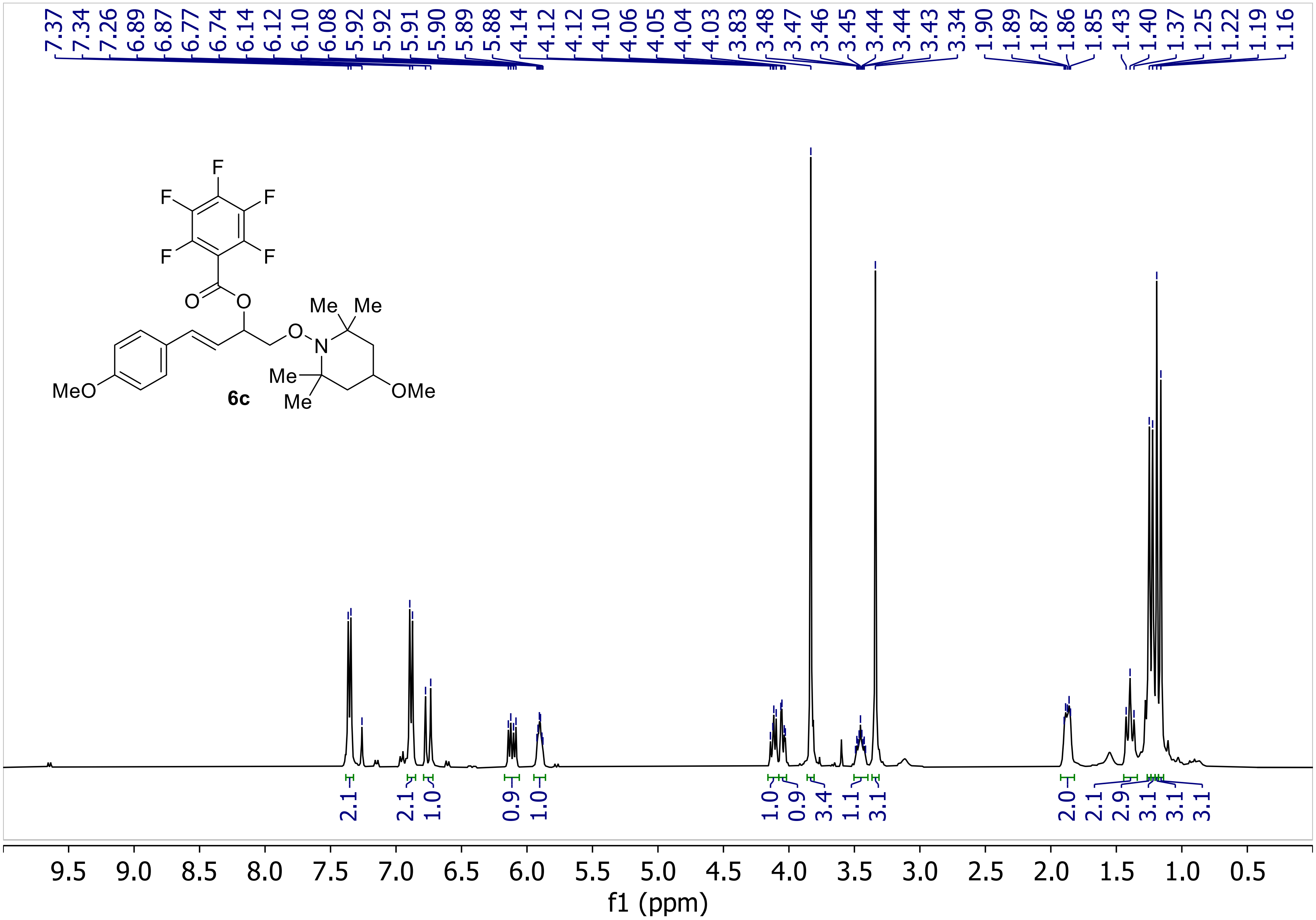
7.37 7.34 7.26 6.89 6.87 6.77 6.74 6.14 6.12 6.10 6.08 5.92 5.92 5.91 5.90 5.89 5.88 4.14 4.12 4.12 4.10 4.06 4.05 4.04 4.03 3.83 3.48 3.47 3.46 3.45 3.44 3.44 3.43 3.34 1.90 1.89 1.87 1.86 1.85 1.43 1.40 1.37 1.25 1.22 1.19 1.16
F
F
F
F
F
O
O
O
N
Me
Me
Me
Me
MeO
OMe
6c
2.1 2.1 1.0 0.9 1.0 1.0 0.9 3.4 1.1 3.1 2.0 2.1 2.9 3.1 3.1 3.1
9.5 9.0 8.5 8.0 7.5 7.0 6.5 6.0 5.5 5.0 4.5 4.0 3.5 3.0 2.5 2.0 1.5 1.0 0.5
f1 (ppm)

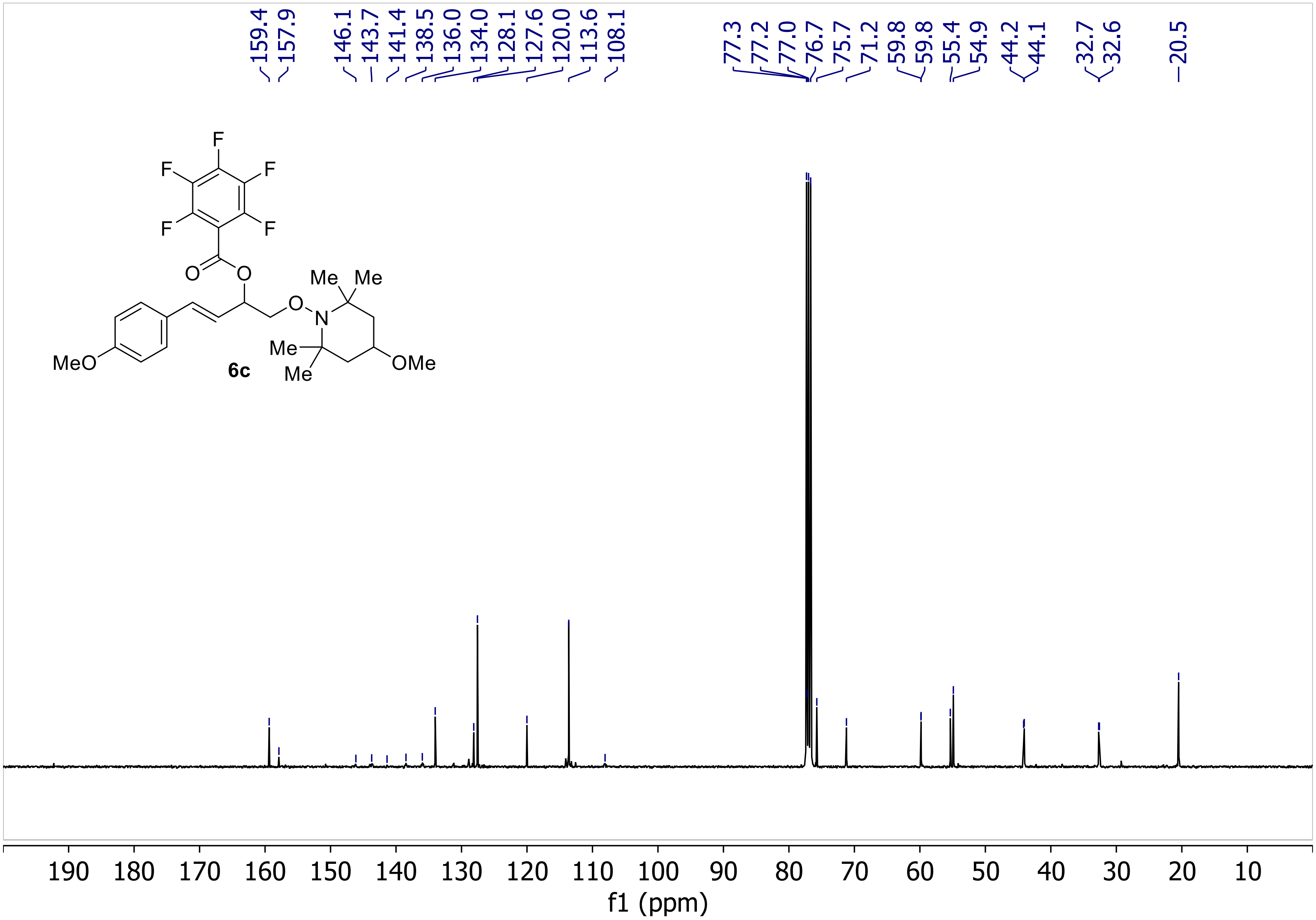

159.4
157.9
146.1
143.7
141.4
138.5
136.0
134.0
128.1
127.6
120.0
113.6
108.1
77.3
77.2
77.0
76.7
75.7
71.2
59.8
59.8
55.4
54.9
44.2
44.1
32.7
32.6
20.5
F
F
F
F
F
O
O
O
N
Me
Me
Me
Me
MeO
OMe
6c
190 180 170 160 150 140 130 120 110 100 90 80 70 60 50 40 30 20 10
f1 (ppm)

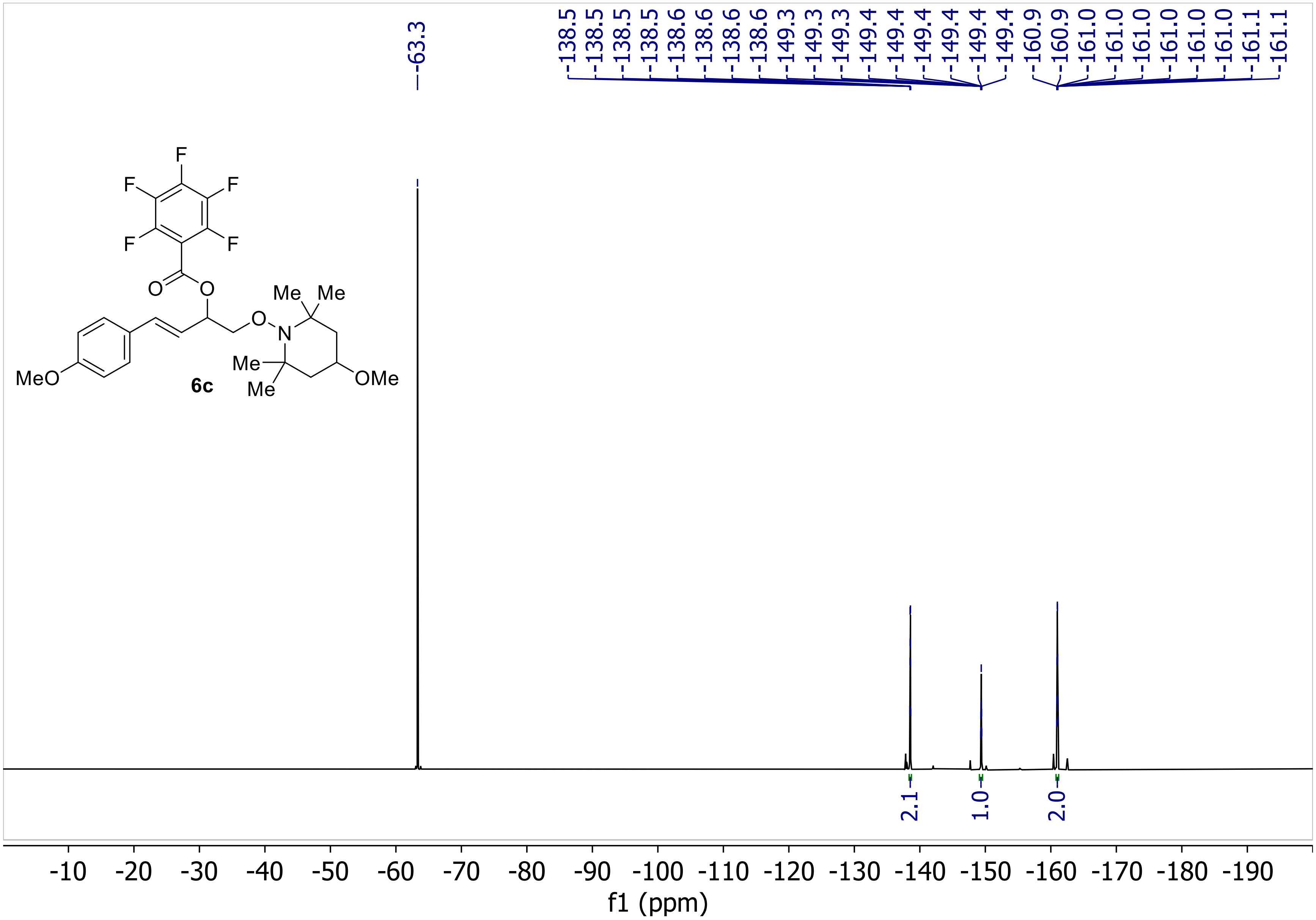
-63.3
-138.5
-138.5
-138.5
-138.5
-138.6
-138.6
-138.6
-138.6
-149.3
-149.3
-149.3
-149.4
-149.4
-149.4
-149.4
-149.4
-149.4
-160.9
-160.9
-161.0
-161.0
-161.0
-161.0
-161.0
-161.0
-161.1
-161.1
F
F
F
F
F
O
O
O
N
Me
Me
Me
Me
MeO
OMe
6c
2.1
1.0
2.0
-10 -20 -30 -40 -50 -60 -70 -80 -90 -100 -110 -120 -130 -140 -150 -160 -170 -180 -190
f1 (ppm)

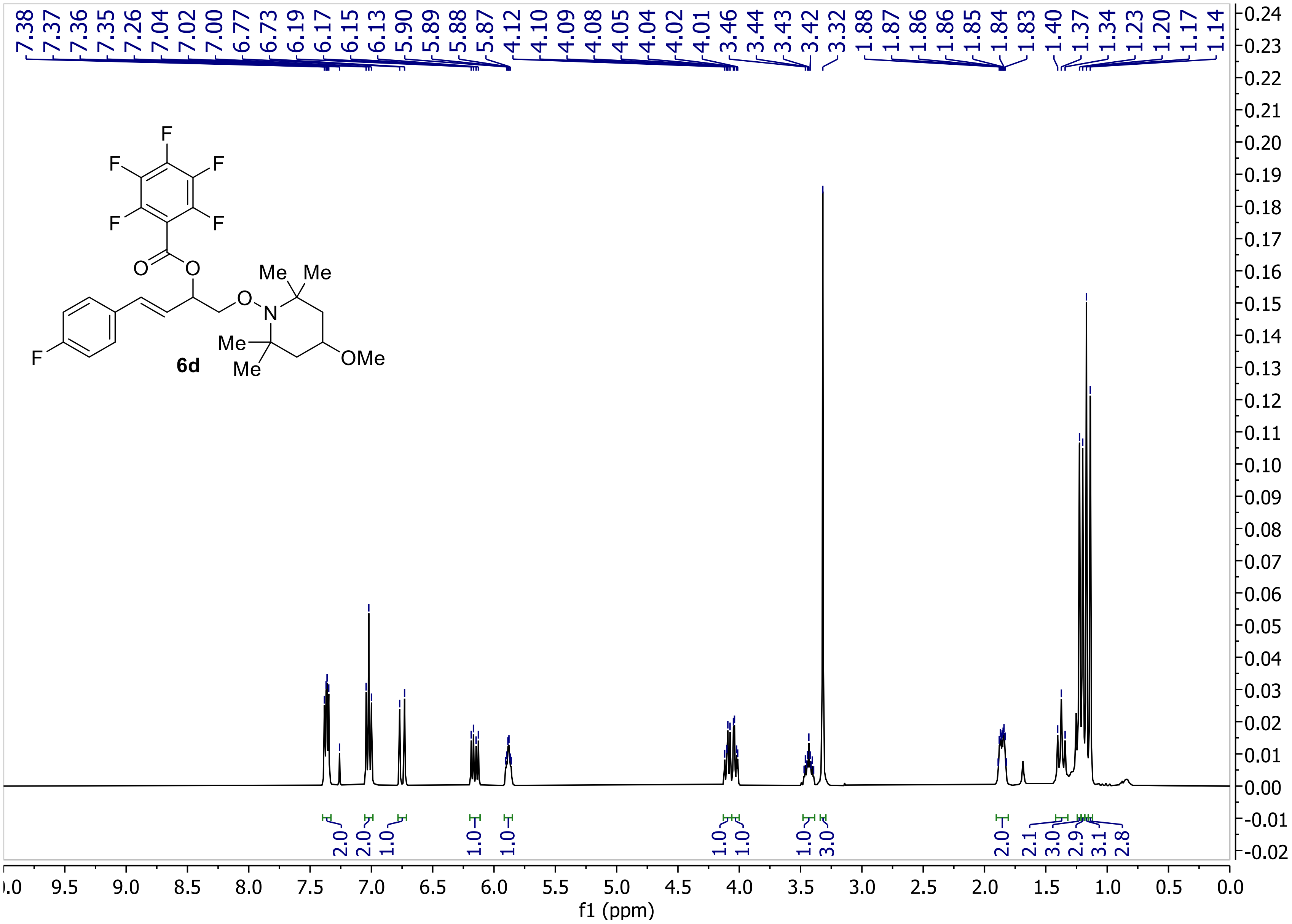
6d
f1 (ppm)

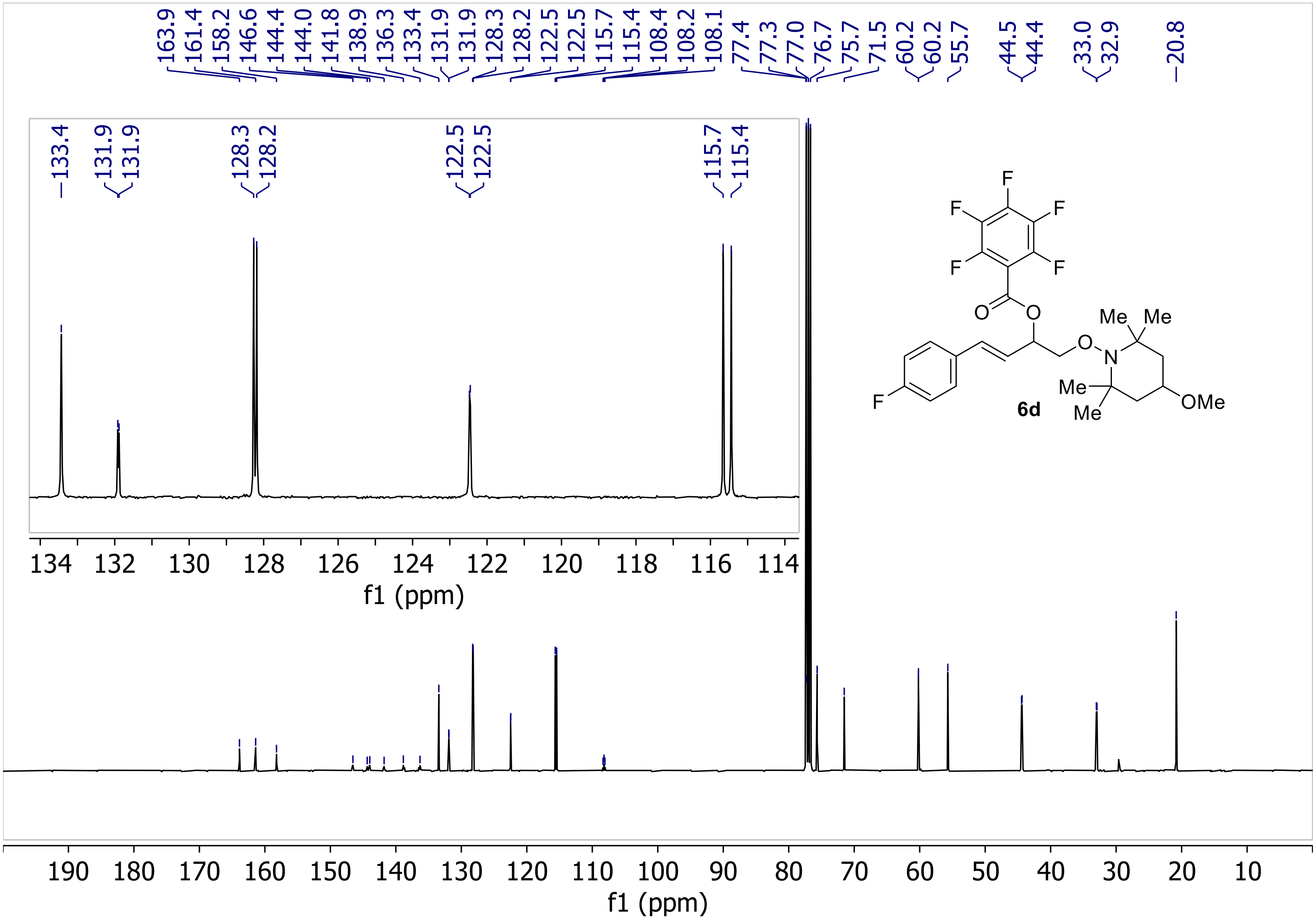
163.9
161.4
158.2
146.6
144.4
144.0
141.8
138.9
136.3
133.4
131.9
131.9
128.3
128.2
122.5
122.5
115.7
115.4
108.4
108.2
108.1
77.4
77.3
77.0
76.7
75.7
71.5
60.2
60.2
55.7
44.5
44.4
33.0
32.9
20.8
F
F
F
F
F
O
O
O
N
Me
Me
Me
Me
OMe
F
6d
f1 (ppm)
f1 (ppm)

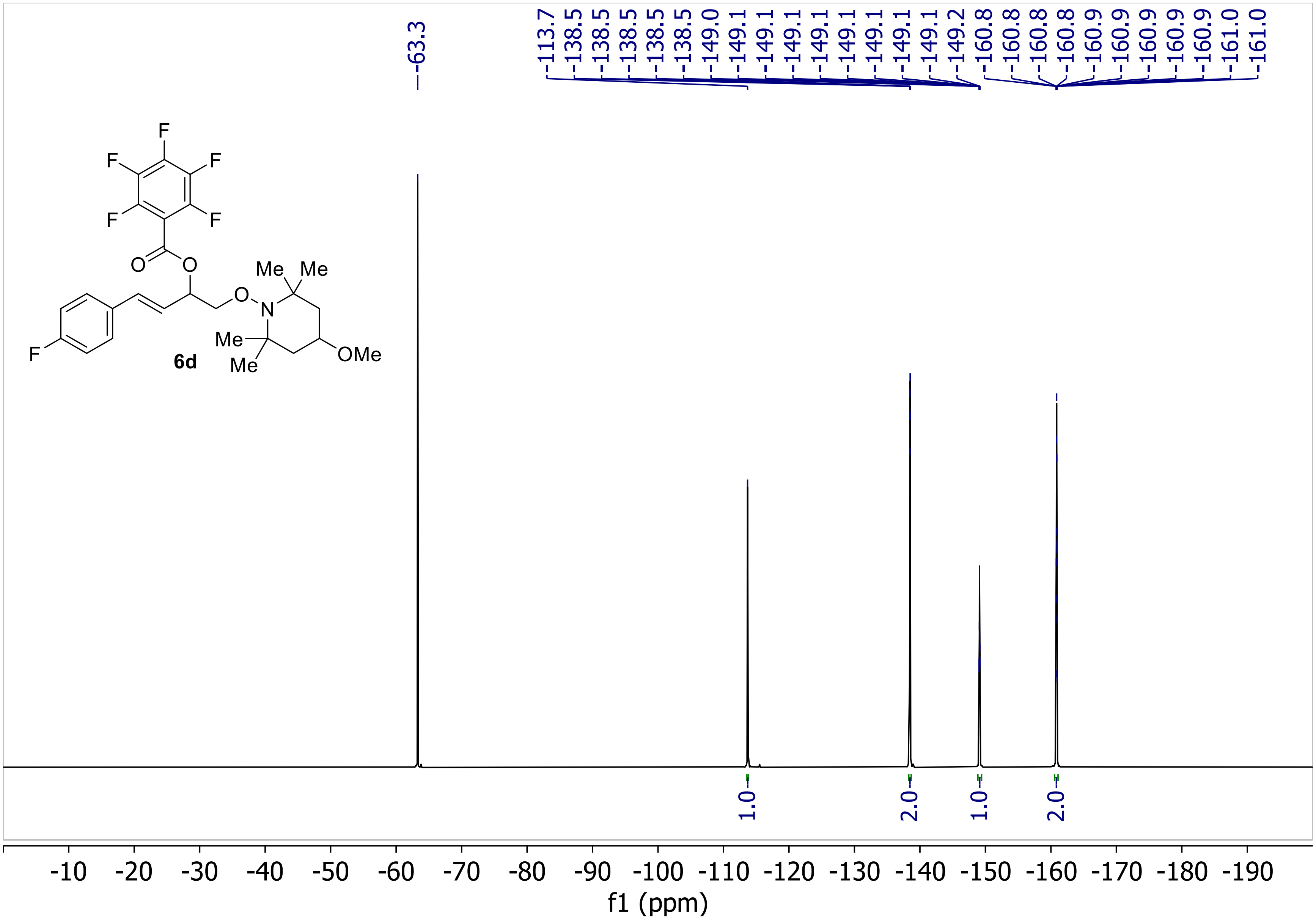
-63.3
-113.7
-138.5
-138.5
-138.5
-138.5
-138.5
-149.0
-149.1
-149.1
-149.1
-149.1
-149.1
-149.1
-149.1
-149.1
-149.2
-160.8
-160.8
-160.8
-160.8
-160.9
-160.9
-160.9
-160.9
-160.9
-161.0
-161.0
F
F
F
F
F
O
O
Me
Me
O
N
Me
Me
OMe
F
6d
1.0
2.0
1.0
2.0
-10 -20 -30 -40 -50 -60 -70 -80 -90 -100 -110 -120 -130 -140 -150 -160 -170 -180 -190
f1 (ppm)

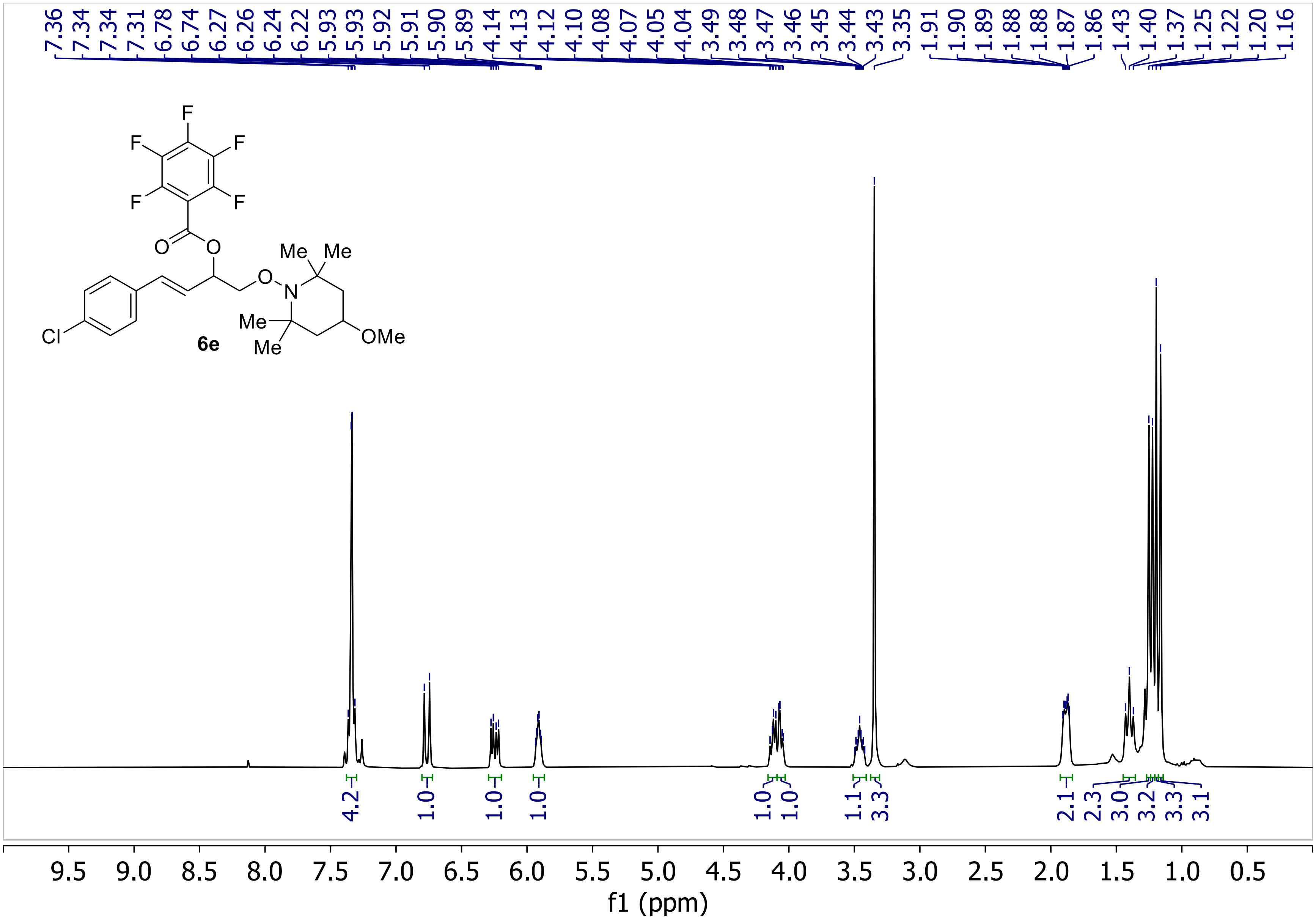
6e
f1 (ppm)

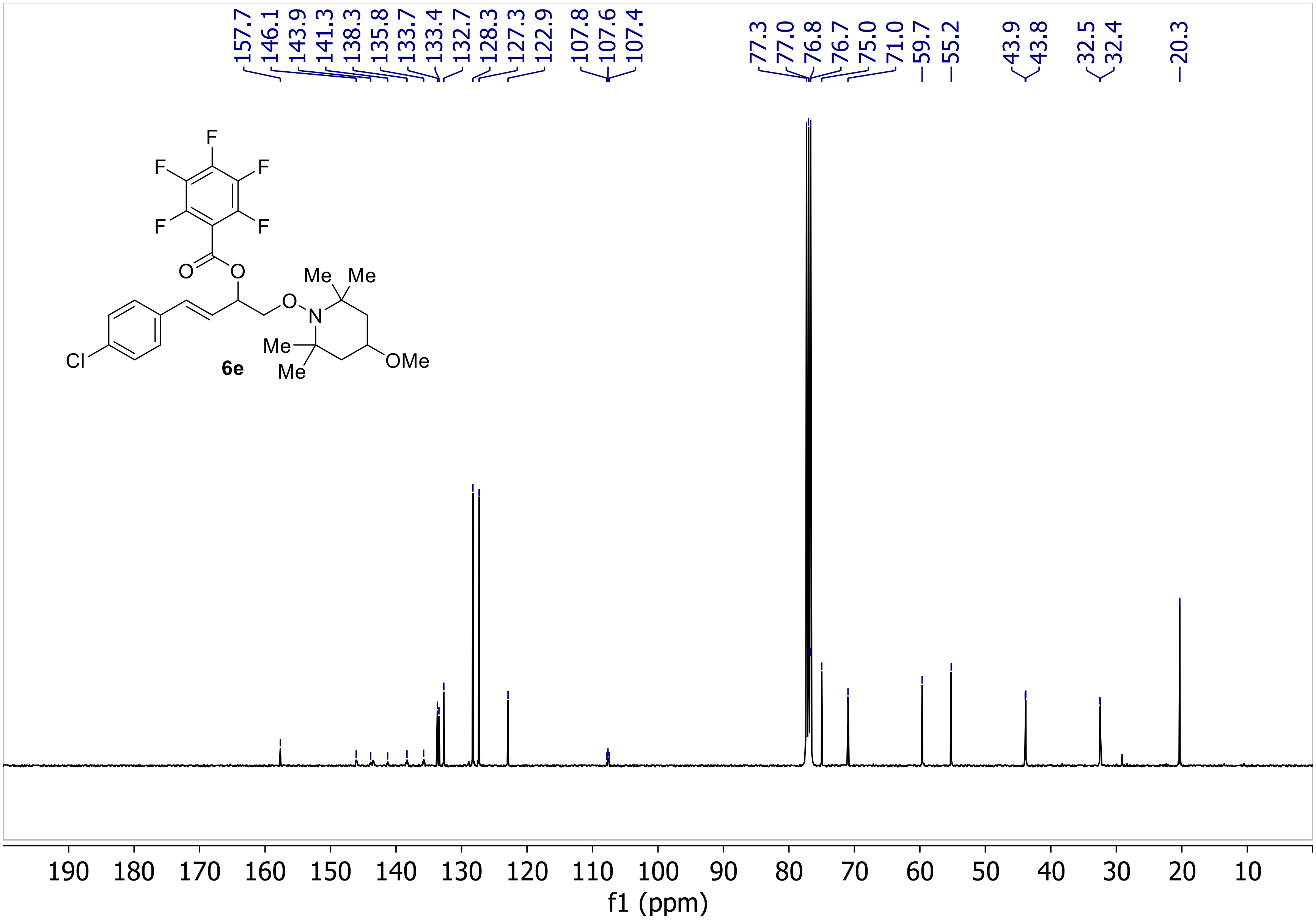
157.7
146.1
143.9
141.3
138.3
135.8
133.7
133.4
132.7
128.3
127.3
122.9
107.8
107.6
107.4
77.3
77.0
76.8
76.7
75.0
71.0
59.7
55.2
43.9
43.8
32.5
32.4
20.3
F
F
F
F
F
O
O
O
N
Me
Me
Me
Me
OMe
Cl
6e
190 180 170 160 150 140 130 120 110 100 90 80 70 60 50 40 30 20 10
f1 (ppm)

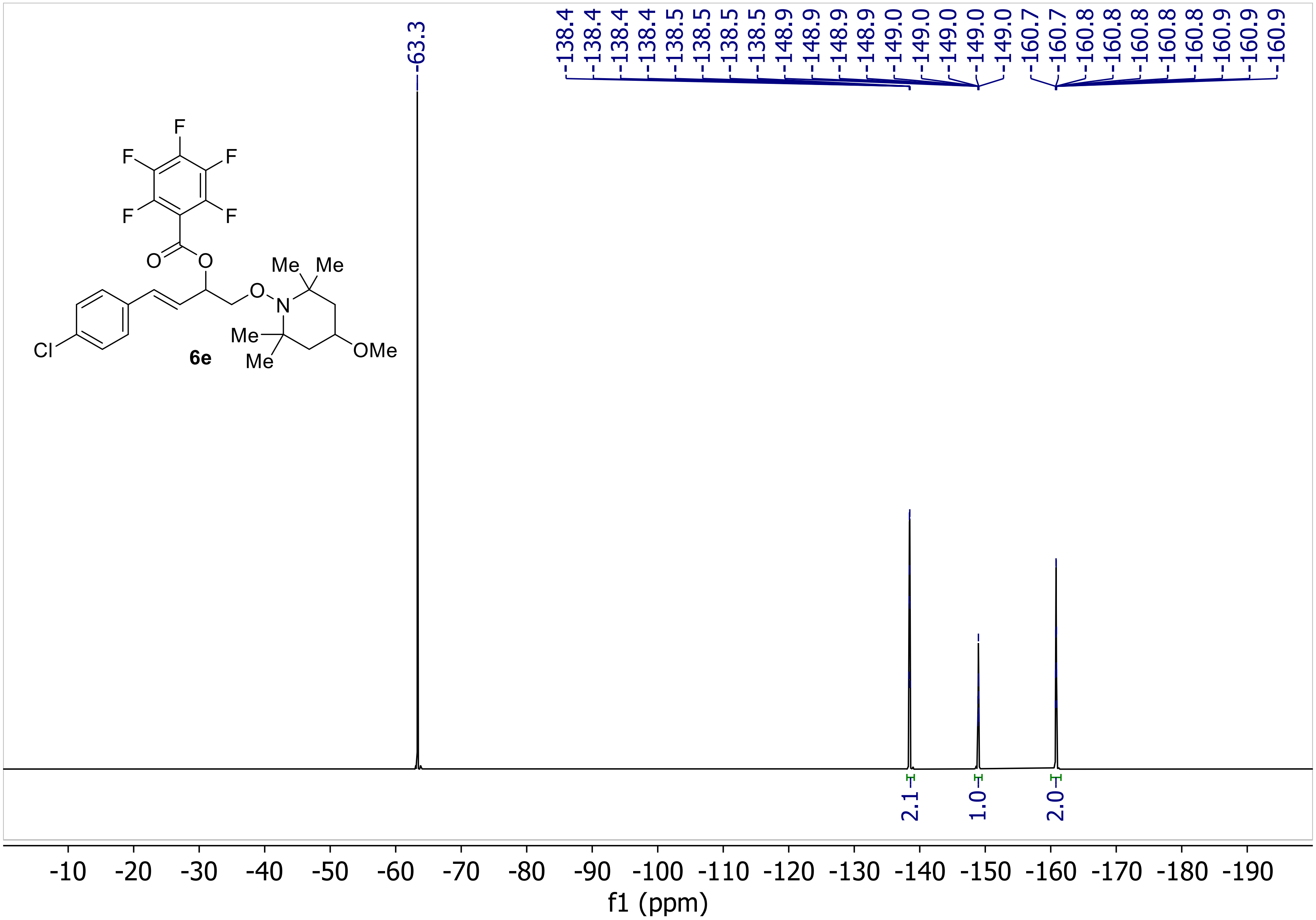
6e
-63.3
-138.4
-138.4
-138.4
-138.4
-138.5
-138.5
-138.5
-138.5
-148.9
-148.9
-148.9
-148.9
-149.0
-149.0
-149.0
-149.0
-149.0
-160.7
-160.7
-160.8
-160.8
-160.8
-160.8
-160.8
-160.9
-160.9
-160.9
2.1
1.0
2.0
-10 -20 -30 -40 -50 -60 -70 -80 -90 -100 -110 -120 -130 -140 -150 -160 -170 -180 -190
f1 (ppm)

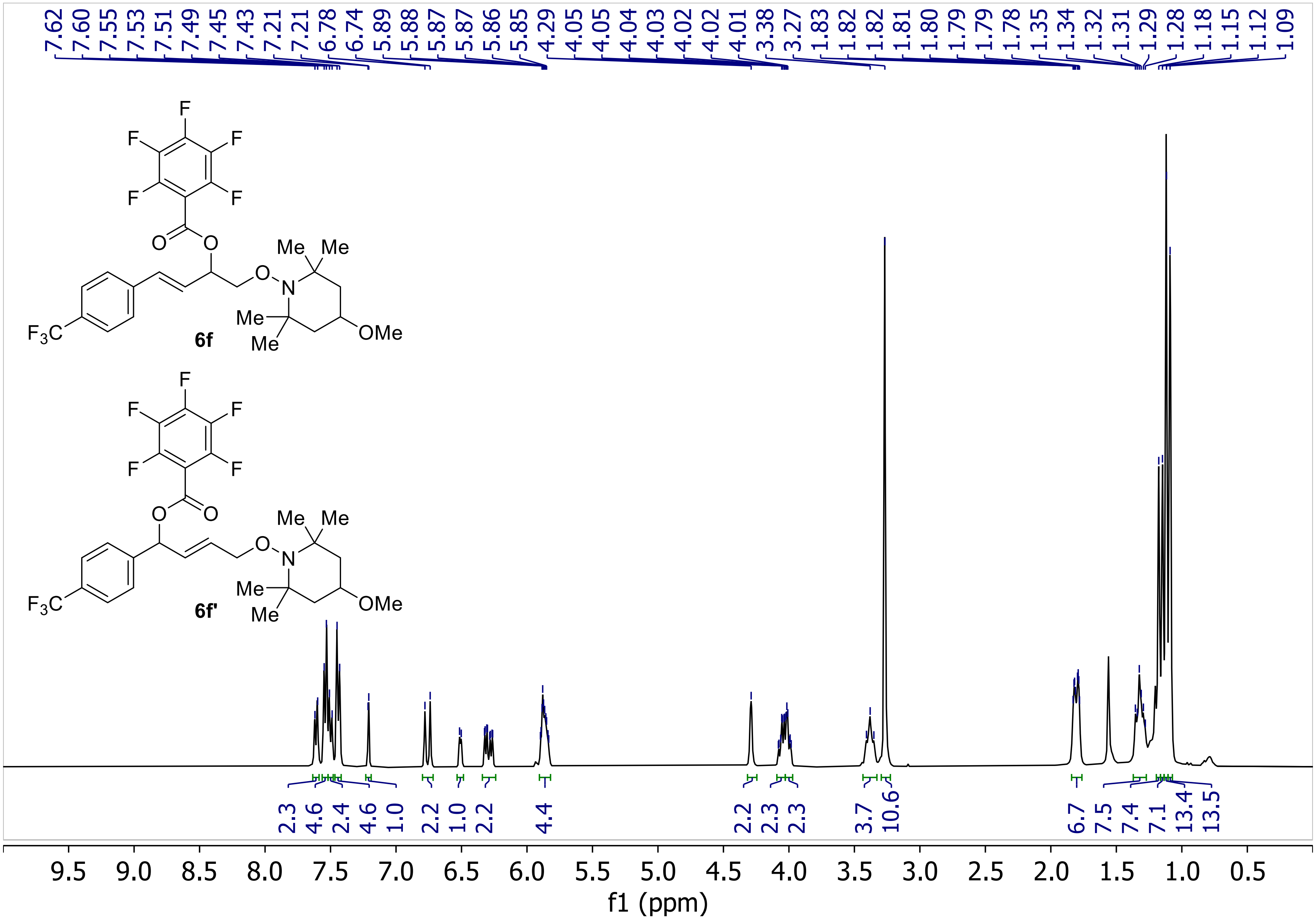
7.62 7.60 7.55 7.53 7.51 7.49 7.45 7.43 7.21 7.21 6.78 6.74 5.89 5.88 5.87 5.87 5.86 5.85 4.29 4.05 4.05 4.04 4.03 4.02 4.02 4.01 3.38 3.27 1.83 1.82 1.82 1.81 1.80 1.79 1.79 1.78 1.35 1.34 1.32 1.31 1.29 1.28 1.18 1.15 1.12 1.09
6f
6f'
2.3 4.6 2.4 4.6 1.0 2.2 1.0 2.2 4.4 2.2 2.3 2.3 3.7 10.6 6.7 7.5 7.4 7.1 13.4 13.5
9.5 9.0 8.5 8.0 7.5 7.0 6.5 6.0 5.5 5.0 4.5 4.0 3.5 3.0 2.5 2.0 1.5 1.0 0.5
f1 (ppm)

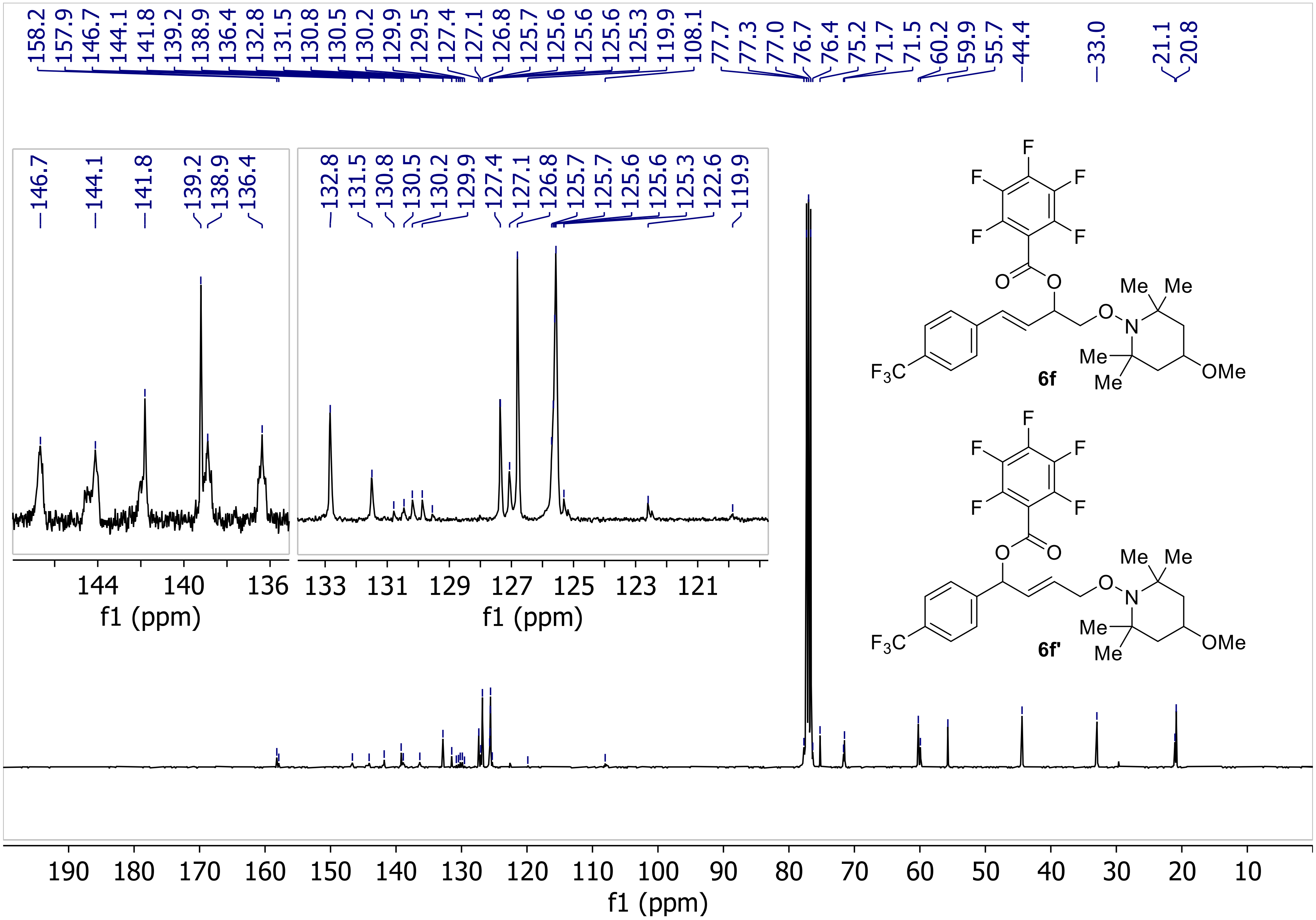
6f
6f'
f1 (ppm)

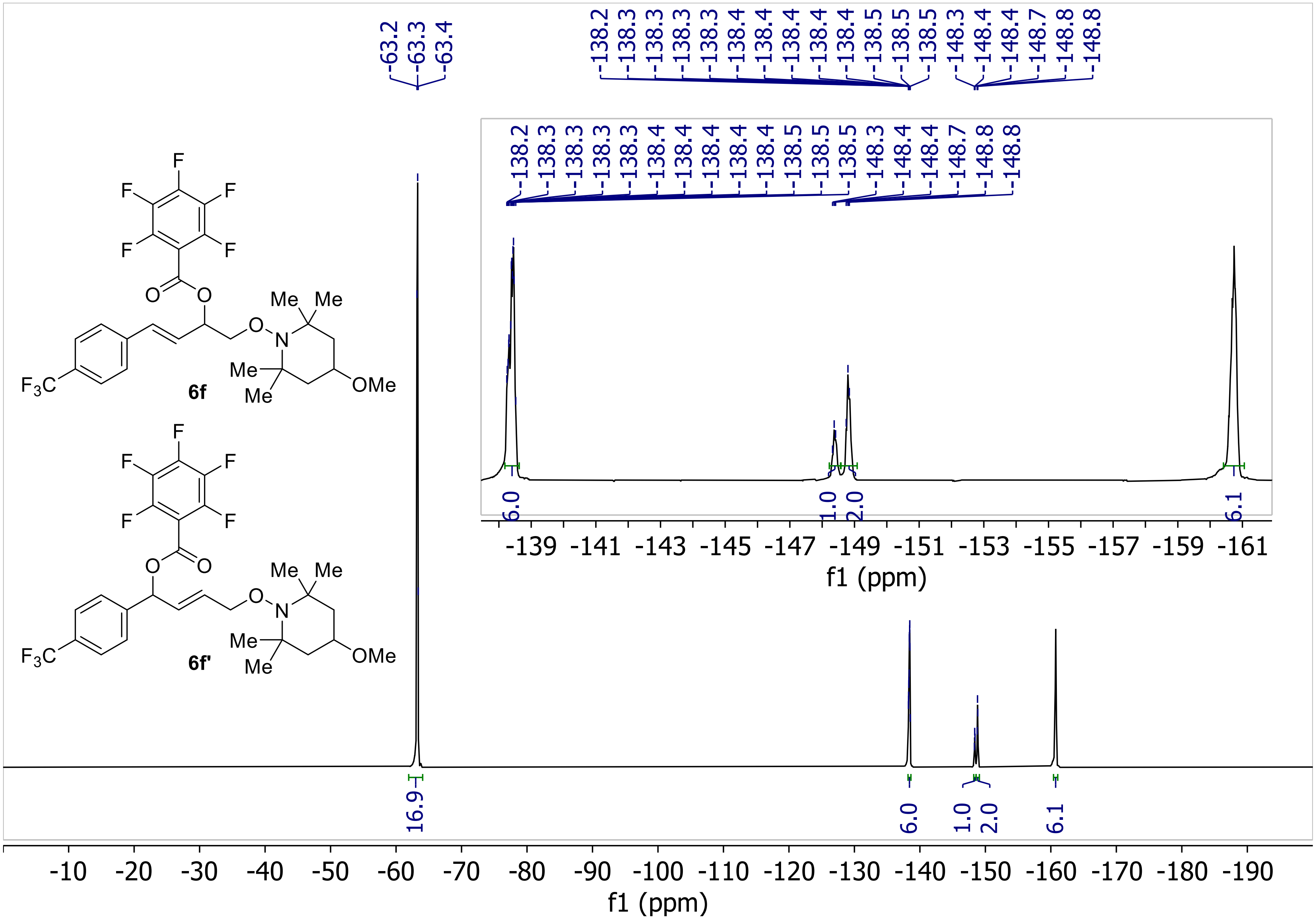
6f
6f'
-63.2
-63.3
-63.4
16.9
6.0
1.0
2.0
6.1
f1 (ppm)

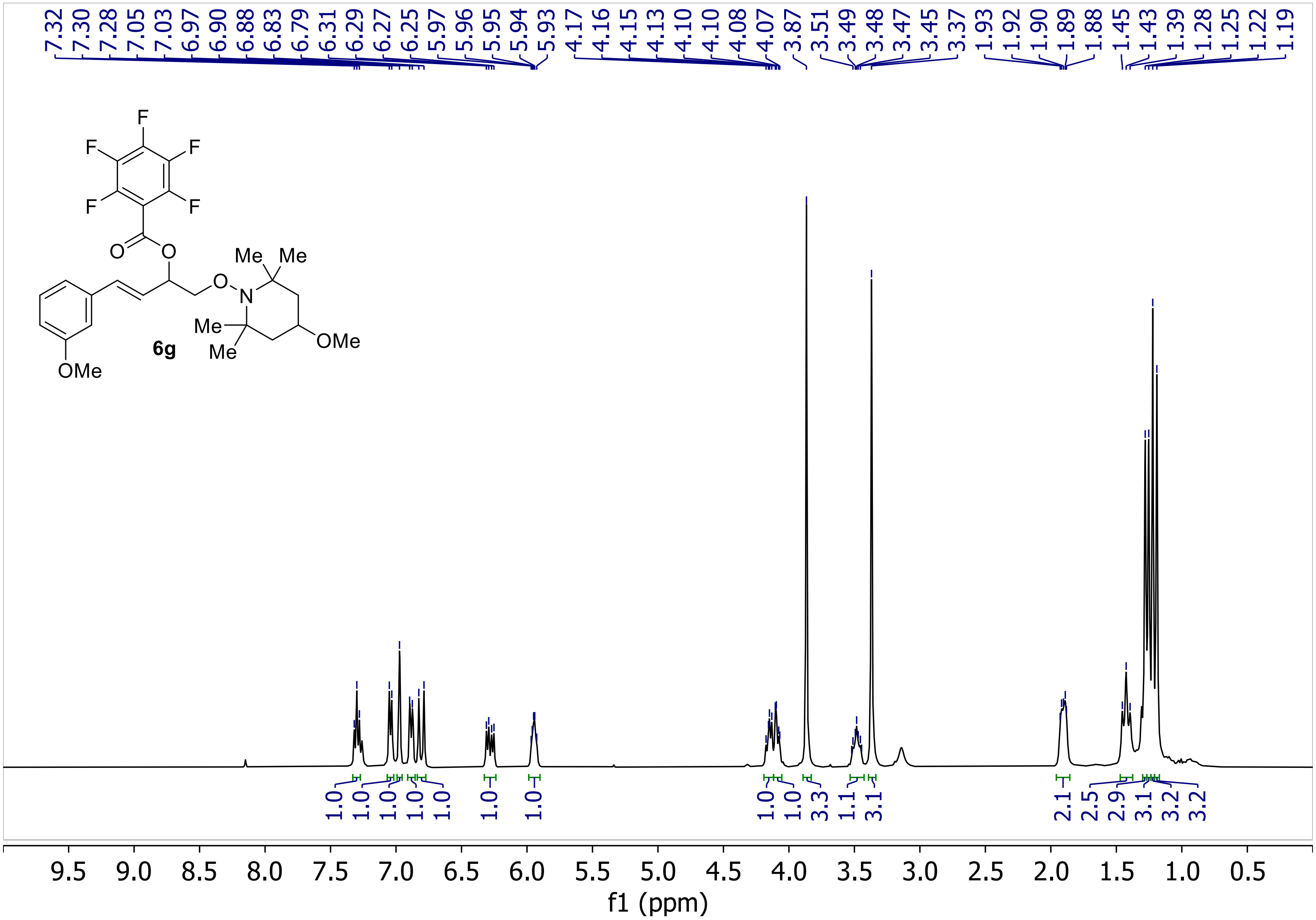
6g
7.32 7.30 7.28 7.05 7.03 6.97 6.90 6.88 6.83 6.79 6.31 6.29 6.27 6.25 5.97 5.96 5.95 5.94 5.93 4.17 4.16 4.15 4.13 4.10 4.10 4.08 4.07 3.87 3.51 3.49 3.48 3.47 3.45 3.37 1.93 1.92 1.90 1.89 1.88 1.45 1.43 1.39 1.28 1.25 1.22 1.19
1.0 1.0 1.0 1.0 1.0 1.0 1.0 1.0 1.0 3.3 1.1 3.1 2.1 2.5 2.9 3.1 3.2 3.2
9.5 9.0 8.5 8.0 7.5 7.0 6.5 6.0 5.5 5.0 4.5 4.0 3.5 3.0 2.5 2.0 1.5 1.0 0.5
f1 (ppm)

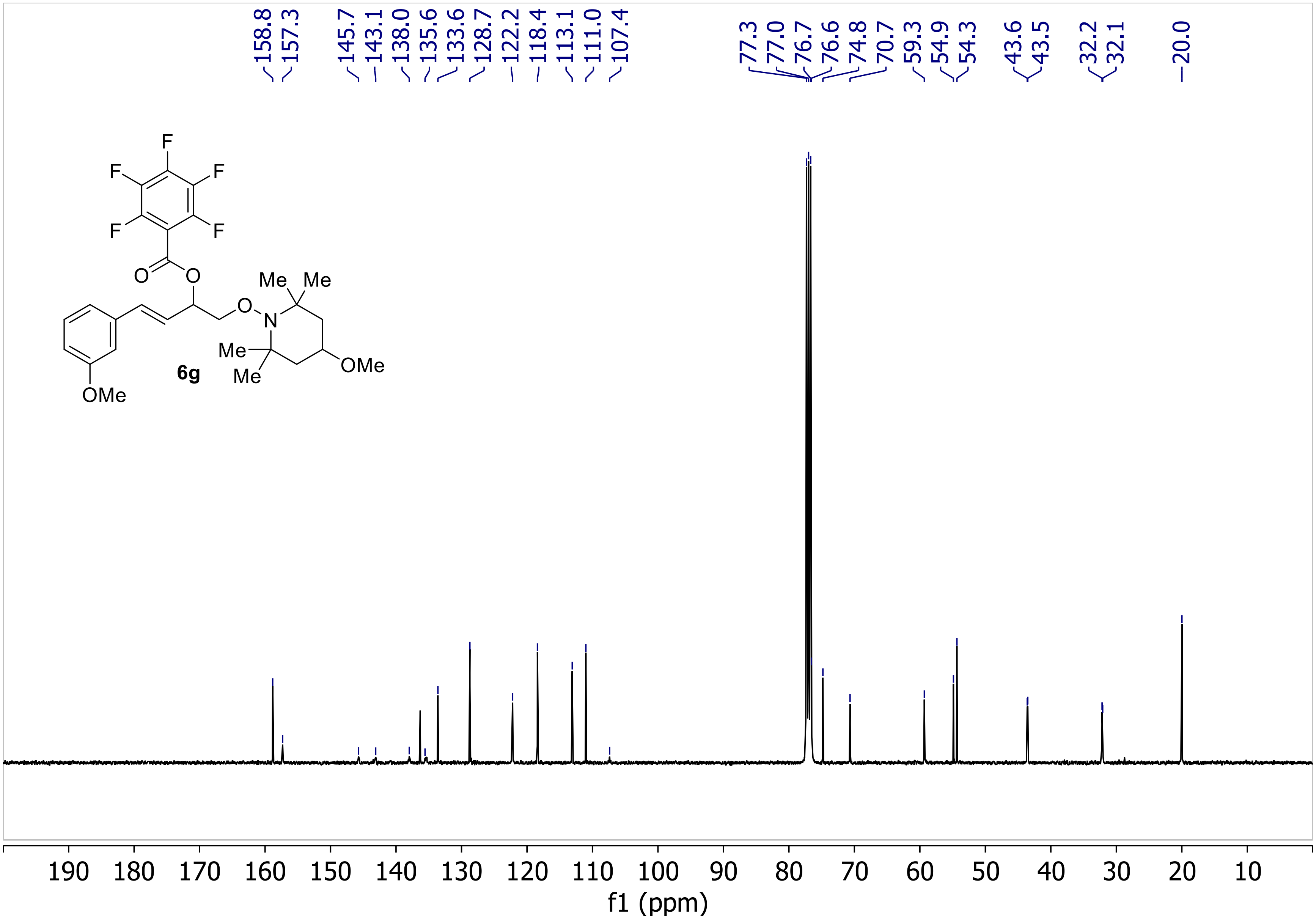
158.8
157.3
145.7
143.1
138.0
135.6
133.6
128.7
122.2
118.4
113.1
111.0
107.4
77.3
77.0
76.7
76.6
74.8
70.7
59.3
54.9
54.3
43.6
43.5
32.2
32.1
20.0
F
F
F
F
F
O
O
O
N
Me
Me
Me
Me
OMe
OMe
6g
190 180 170 160 150 140 130 120 110 100 90 80 70 60 50 40 30 20 10
f1 (ppm)

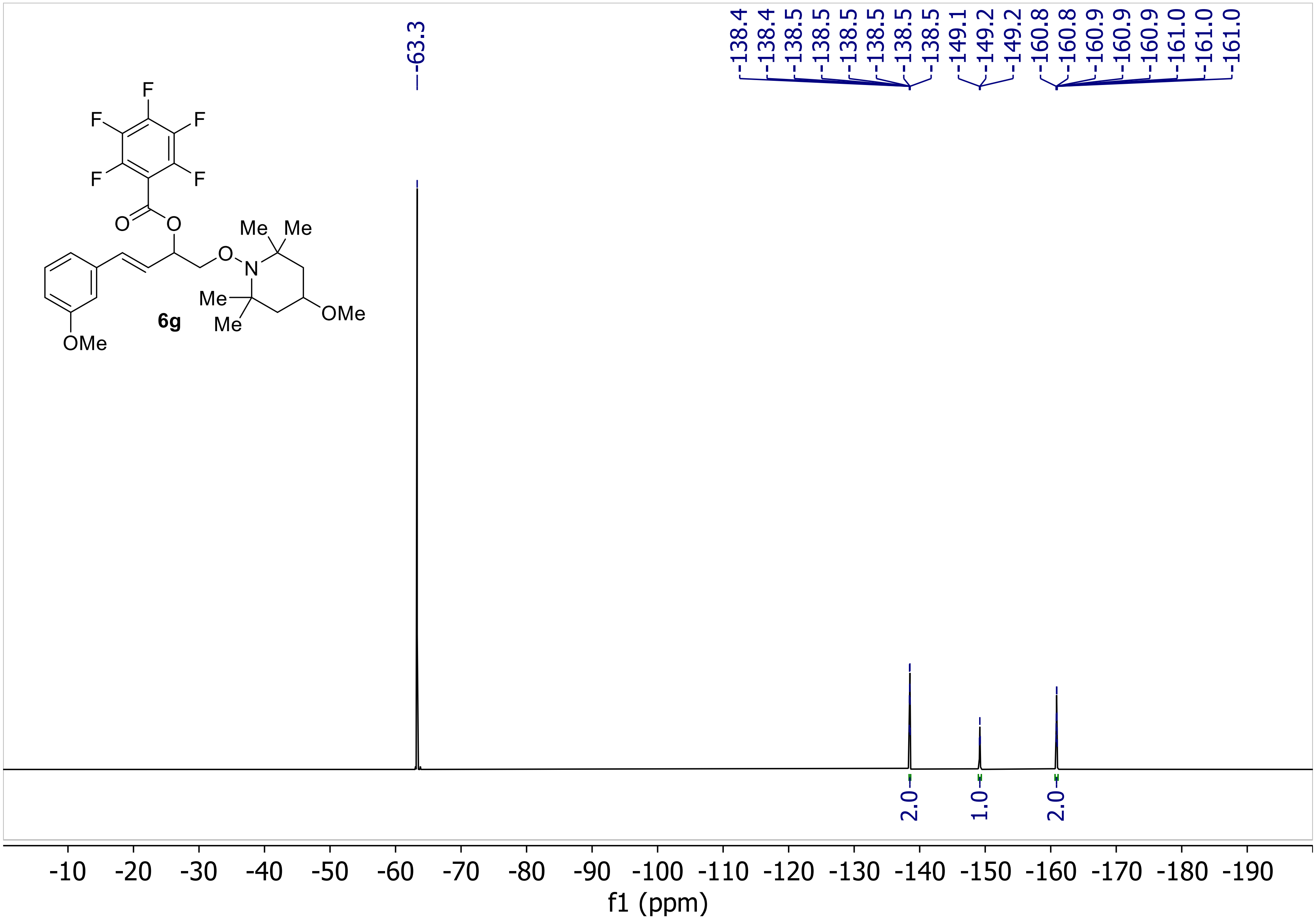
-63.3
-138.4
-138.4
-138.5
-138.5
-138.5
-138.5
-138.5
-138.5
-149.1
-149.2
-149.2
-160.8
-160.8
-160.9
-160.9
-160.9
-161.0
-161.0
-161.0
6g
OMe
Me
F
O
N
2.0
1.0
2.0
-10 -20 -30 -40 -50 -60 -70 -80 -90 -100 -110 -120 -130 -140 -150 -160 -170 -180 -190
f1 (ppm)

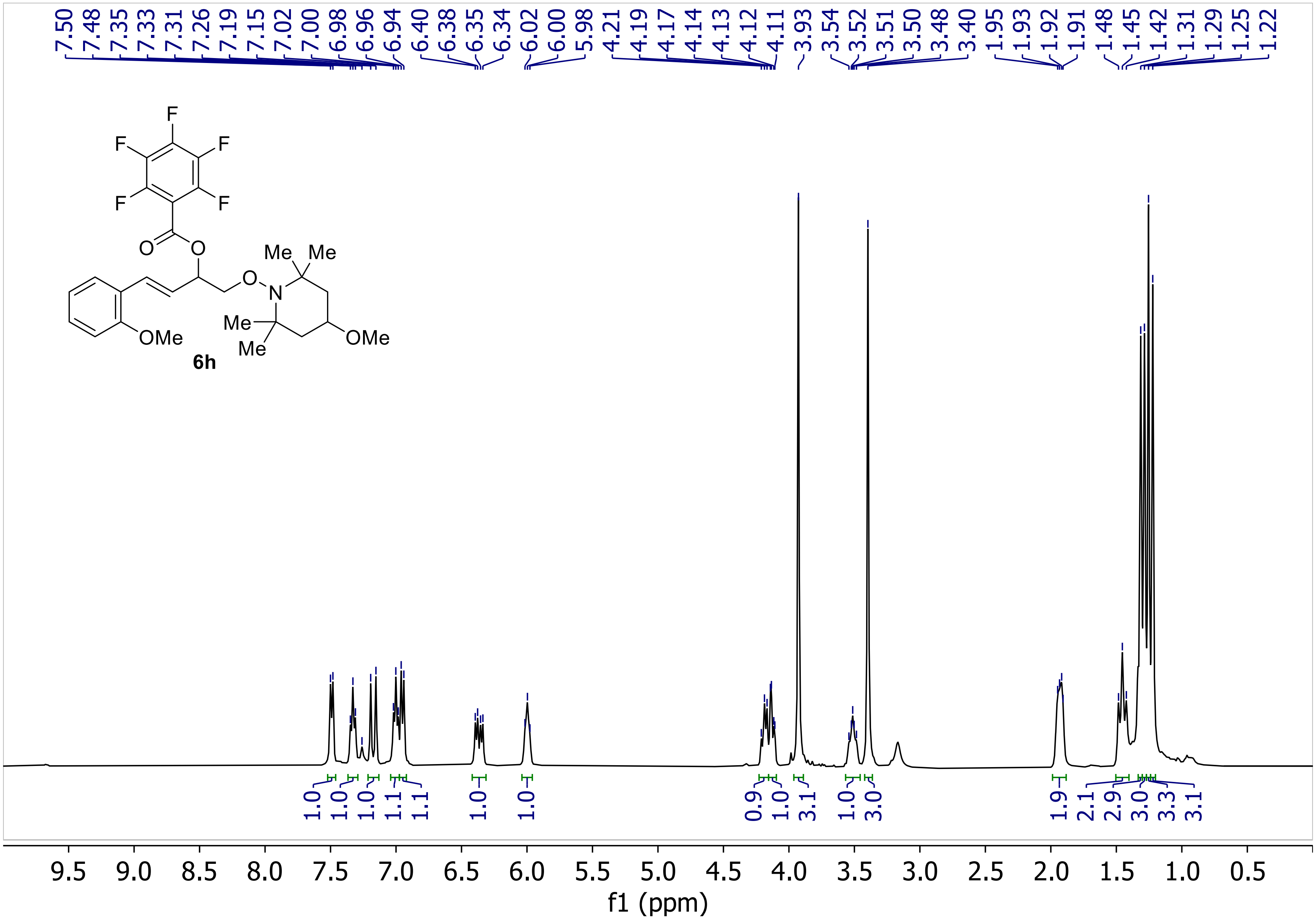
7.50 7.48 7.35 7.33 7.31 7.26 7.19 7.15 7.02 7.00 6.98 6.96 6.94 6.40 6.38 6.35 6.34 6.02 6.00 5.98 4.21 4.19 4.17 4.14 4.13 4.12 4.11 3.93 3.54 3.52 3.51 3.50 3.48 3.40 1.95 1.93 1.92 1.91 1.48 1.45 1.42 1.31 1.29 1.25 1.22
F
O
Me
OMe
N
6h
1.0 1.0 1.0 1.1 1.1 1.0 1.0 0.9 1.0 3.1 1.0 3.0 1.9 2.1 2.9 3.0 3.3 3.1
9.5 9.0 8.5 8.0 7.5 7.0 6.5 6.0 5.5 5.0 4.5 4.0 3.5 3.0 2.5 2.0 1.5 1.0 0.5
f1 (ppm)

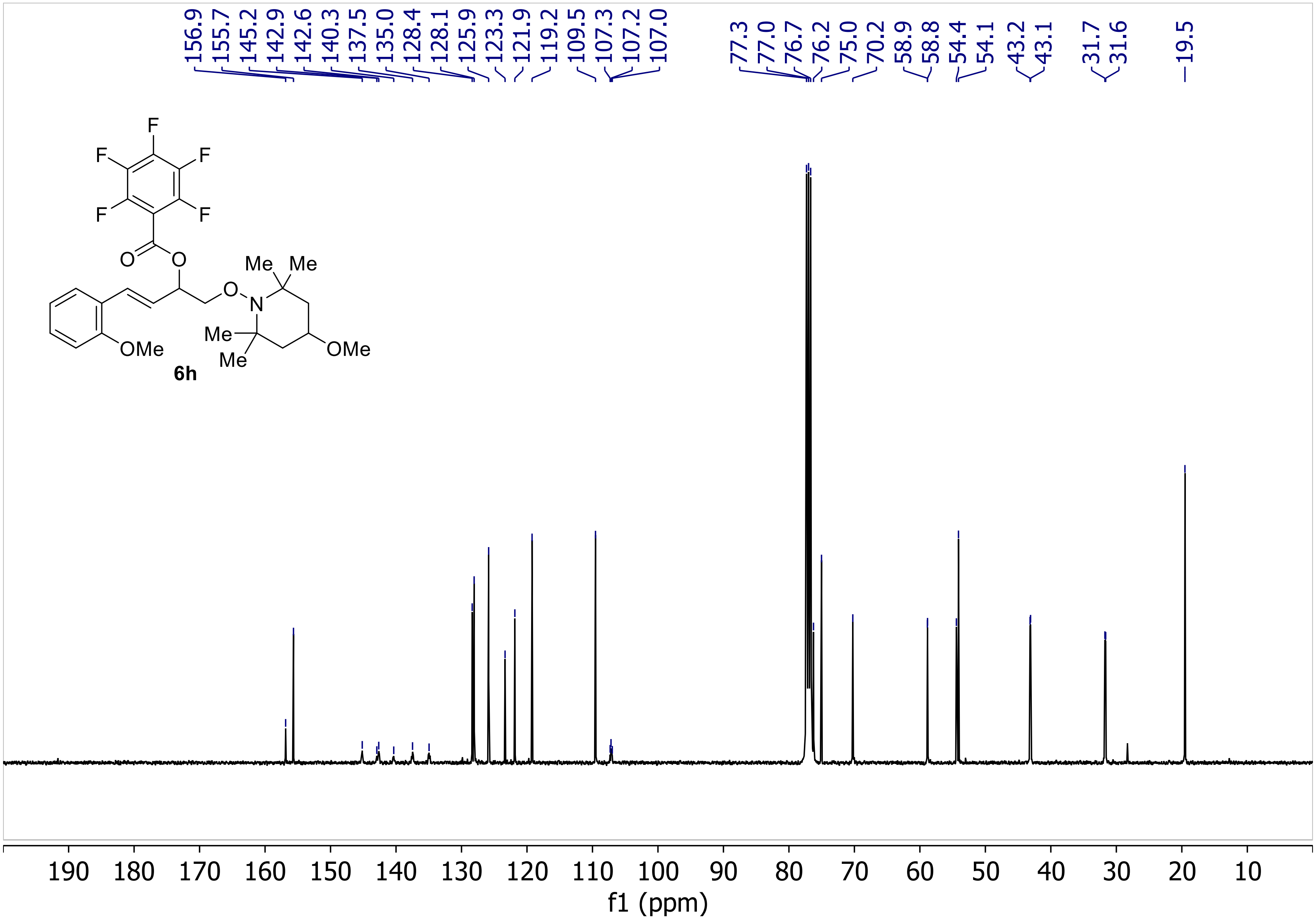
156.9
155.7
145.2
142.9
142.6
140.3
137.5
135.0
128.4
128.1
125.9
123.3
121.9
119.2
109.5
107.3
107.2
107.0
77.3
77.0
76.7
76.2
75.0
70.2
58.9
58.8
54.4
54.1
43.2
43.1
31.7
31.6
19.5
F
F
F
F
F
O
O
Me
Me
O
N
Me
Me
OMe
OMe
6h
190
180
170
160
150
140
130
120
110
100
90
80
70
60
50
40
30
20
10
f1 (ppm)

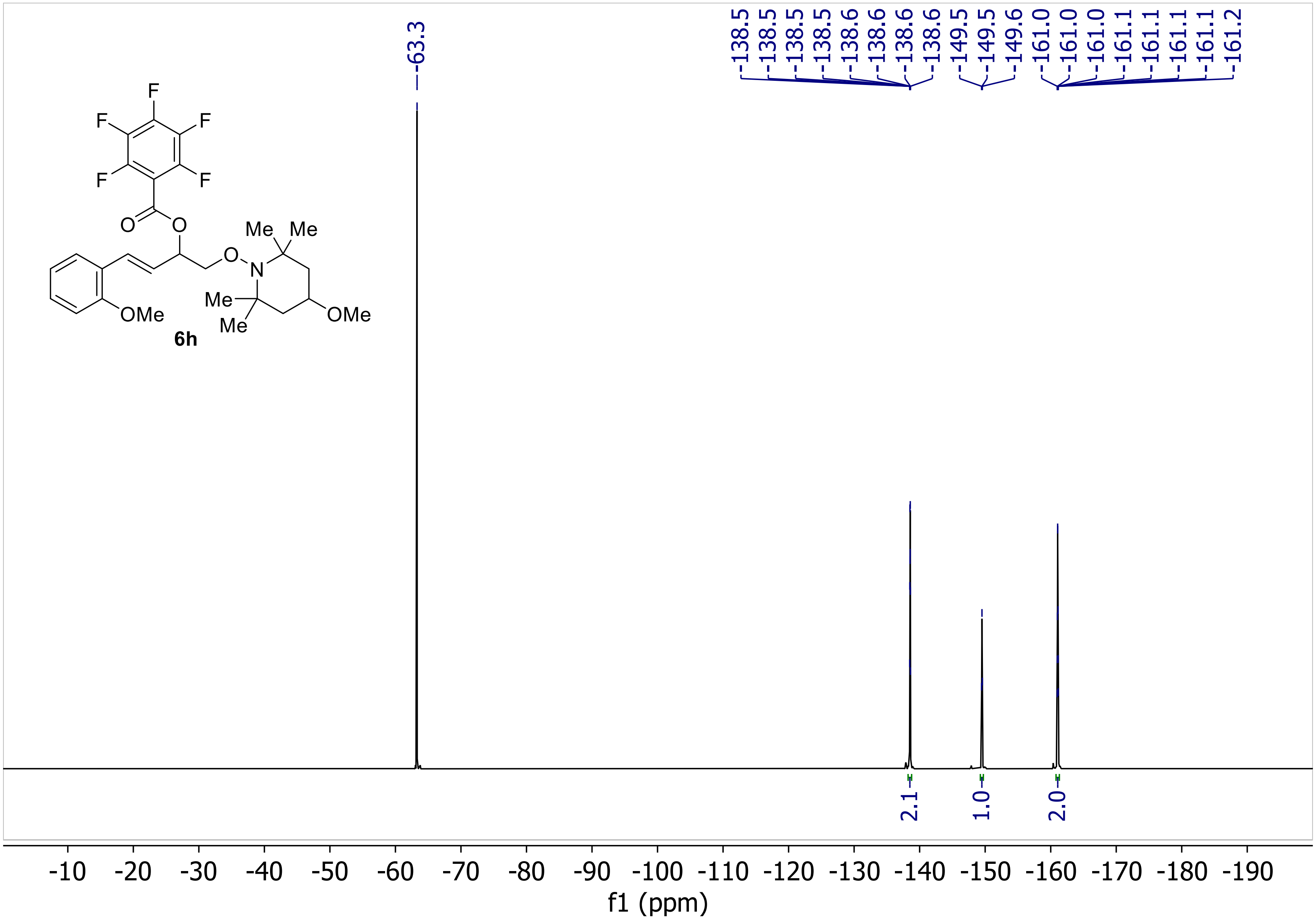

F
F
F
F
F
O
O
Me
Me
O
N
Me
Me
OMe
OMe
6h
-63.3
-138.5
-138.5
-138.5
-138.5
-138.6
-138.6
-138.6
-138.6
-149.5
-149.5
-149.6
-161.0
-161.0
-161.0
-161.1
-161.1
-161.1
-161.1
-161.2
2.1
1.0
2.0
-10 -20 -30 -40 -50 -60 -70 -80 -90 -100 -110 -120 -130 -140 -150 -160 -170 -180 -190
f1 (ppm)

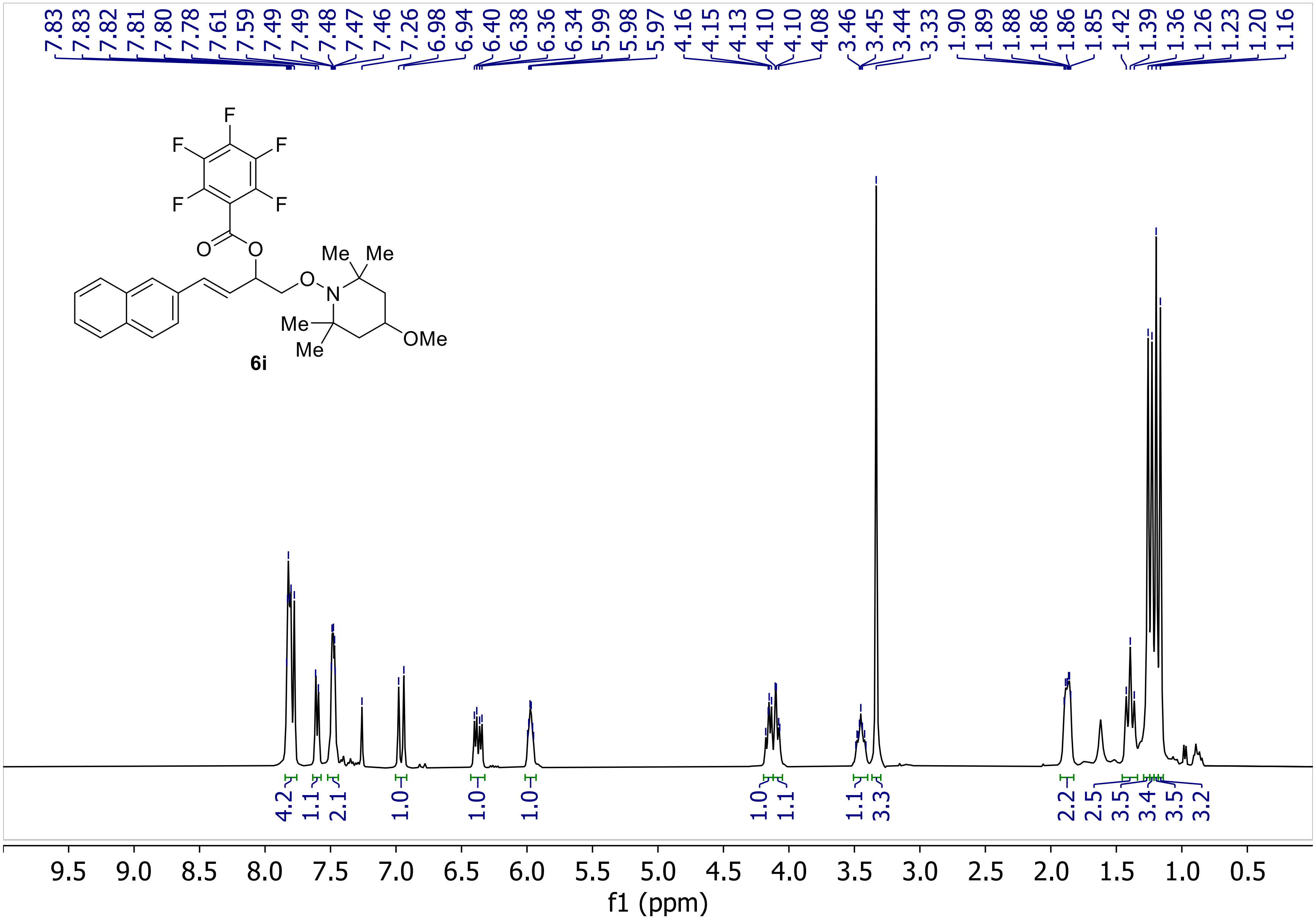
7.83
7.83
7.82
7.81
7.80
7.78
7.61
7.59
7.49
7.49
7.48
7.47
7.46
7.26
6.98
6.94
6.40
6.38
6.36
6.34
5.99
5.98
5.97
4.16
4.15
4.13
4.10
4.10
4.08
3.46
3.45
3.44
3.33
1.90
1.89
1.88
1.86
1.86
1.85
1.42
1.39
1.36
1.26
1.23
1.20
1.16
F
F
F
F
F
O
O
O
N
Me
Me
Me
Me
OMe
6i
4.2
1.1
2.1
1.0
1.0
1.0
1.0
1.1
1.1
3.3
2.2
2.5
3.5
3.4
3.5
3.2
9.5
9.0
8.5
8.0
7.5
7.0
6.5
6.0
5.5
5.0
4.5
4.0
3.5
3.0
2.5
2.0
1.5
1.0
0.5
f1 (ppm)

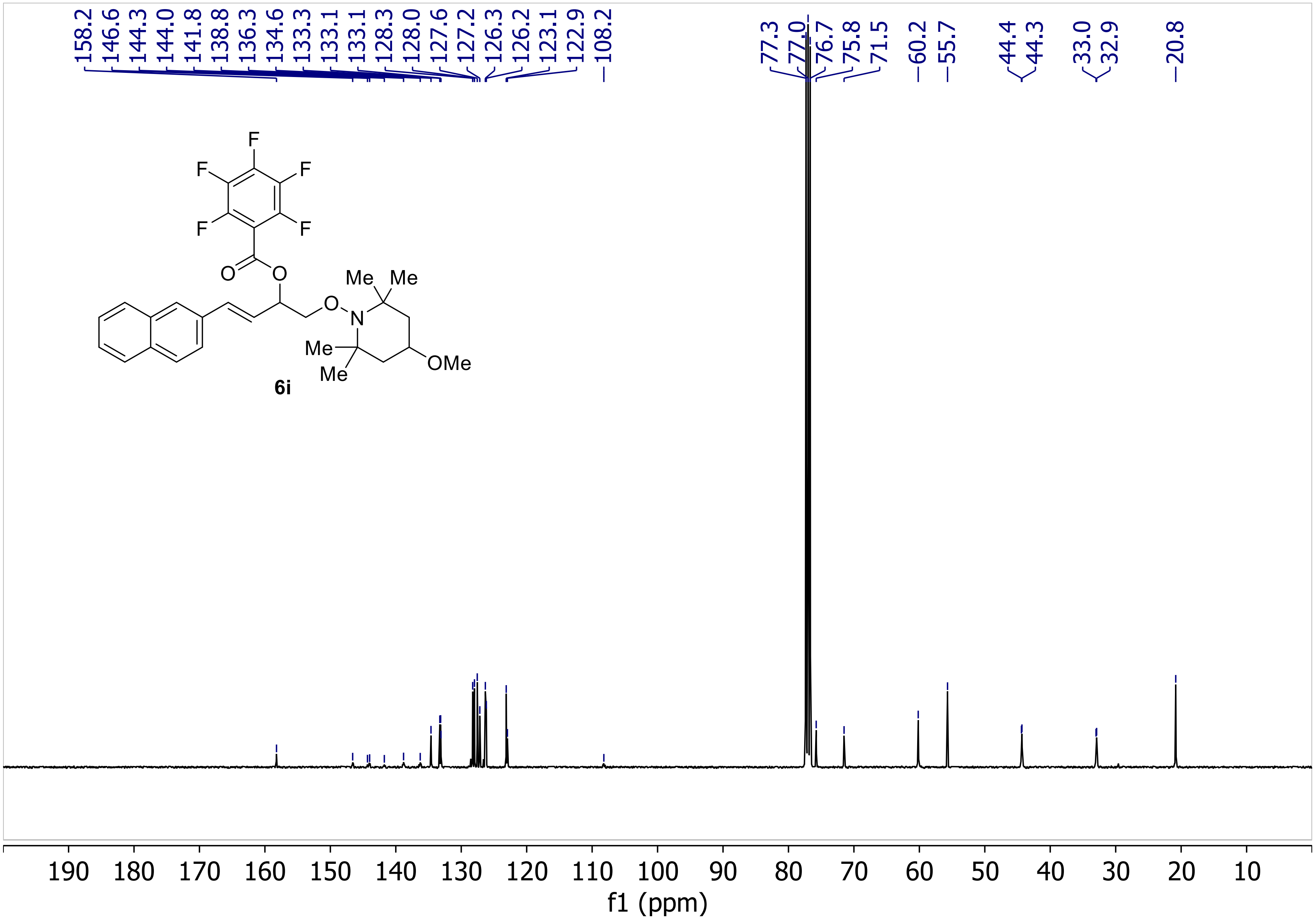
158.2
146.6
144.3
144.0
141.8
138.8
136.3
134.6
133.3
133.1
133.1
128.3
128.0
127.6
127.2
126.3
126.2
123.1
122.9
108.2
77.3
77.0
76.7
75.8
71.5
60.2
55.7
44.4
44.3
33.0
32.9
20.8
6i
190 180 170 160 150 140 130 120 110 100 90 80 70 60 50 40 30 20 10
f1 (ppm)

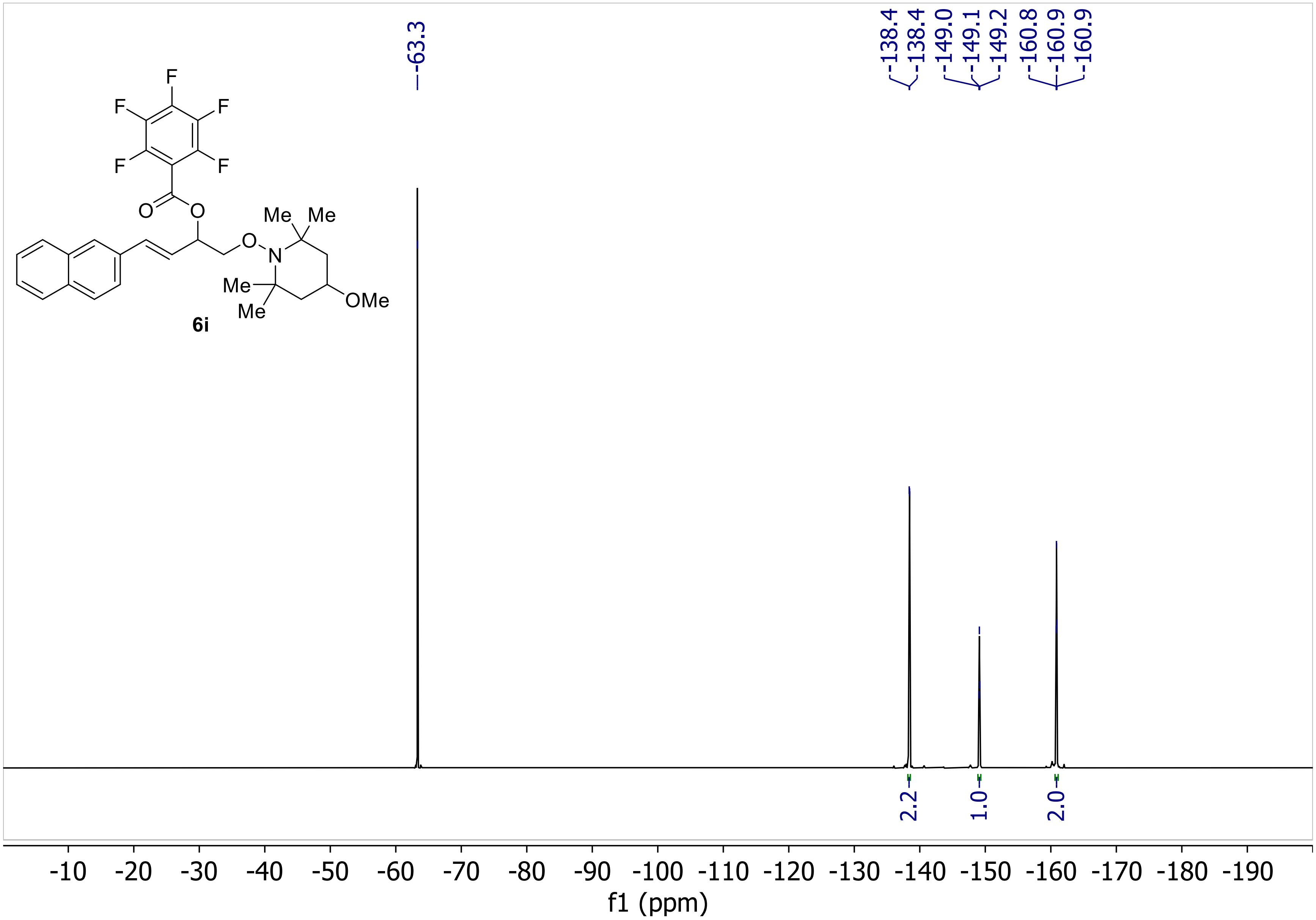

6i
-63.3
-138.4
-138.4
-149.0
-149.1
-149.2
-160.8
-160.9
-160.9
2.2
1.0
2.0
-10 -20 -30 -40 -50 -60 -70 -80 -90 -100 -110 -120 -130 -140 -150 -160 -170 -180 -190
f1 (ppm)

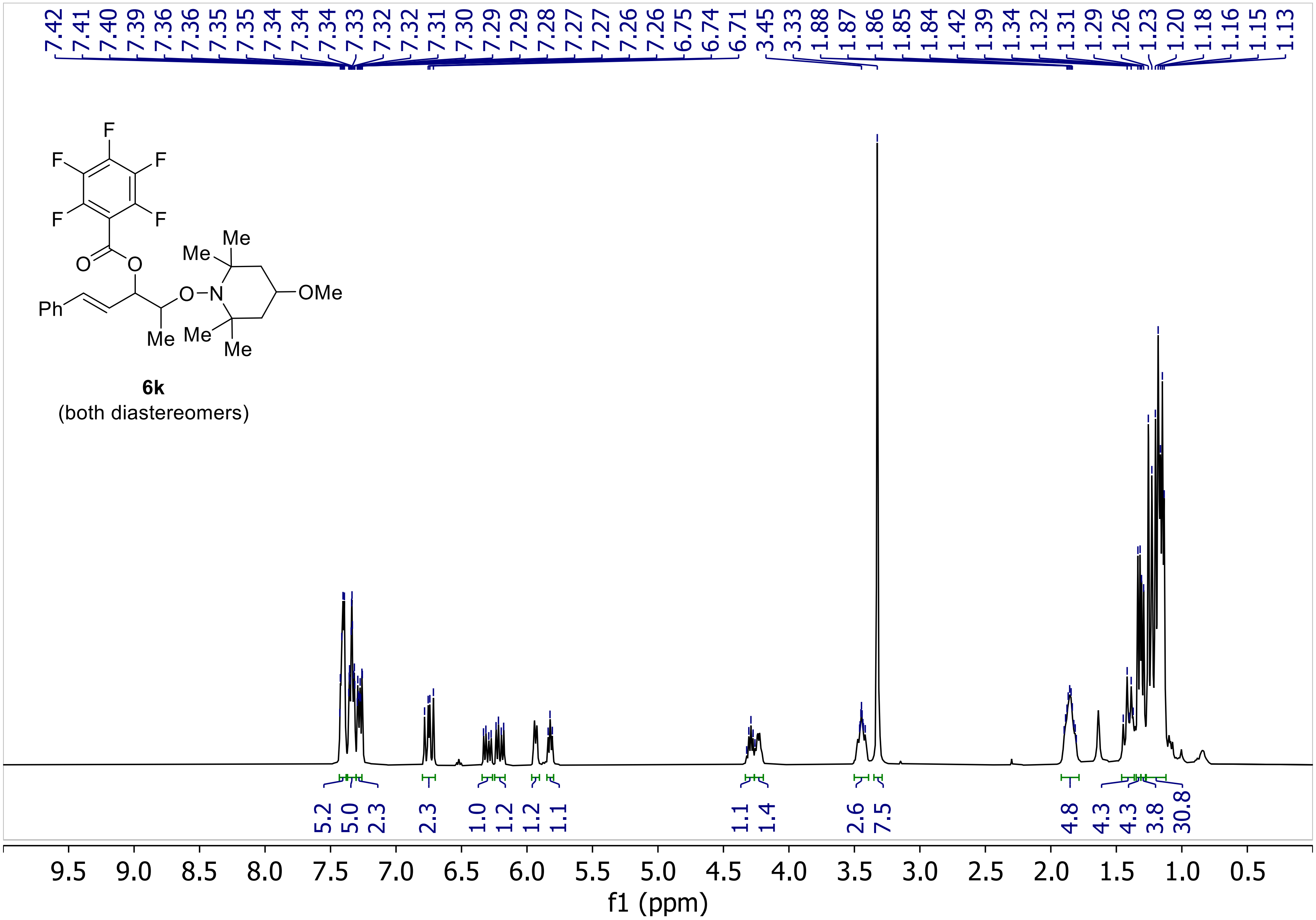
7.42 7.41 7.40 7.39 7.36 7.36 7.35 7.35 7.34 7.34 7.34 7.33 7.32 7.32 7.31 7.30 7.29 7.29 7.28 7.27 7.27 7.26 7.26 6.75 6.74 6.71 3.45 3.33 1.88 1.87 1.86 1.85 1.84 1.42 1.39 1.34 1.32 1.31 1.29 1.26 1.23 1.20 1.18 1.16 1.15 1.13
6k
(both diastereomers)
5.2 5.0 2.3 2.3 1.0 1.2 1.2 1.1 1.1 1.4 2.6 7.5 4.8 4.3 4.3 3.8 30.8
9.5 9.0 8.5 8.0 7.5 7.0 6.5 6.0 5.5 5.0 4.5 4.0 3.5 3.0 2.5 2.0 1.5 1.0 0.5
f1 (ppm)

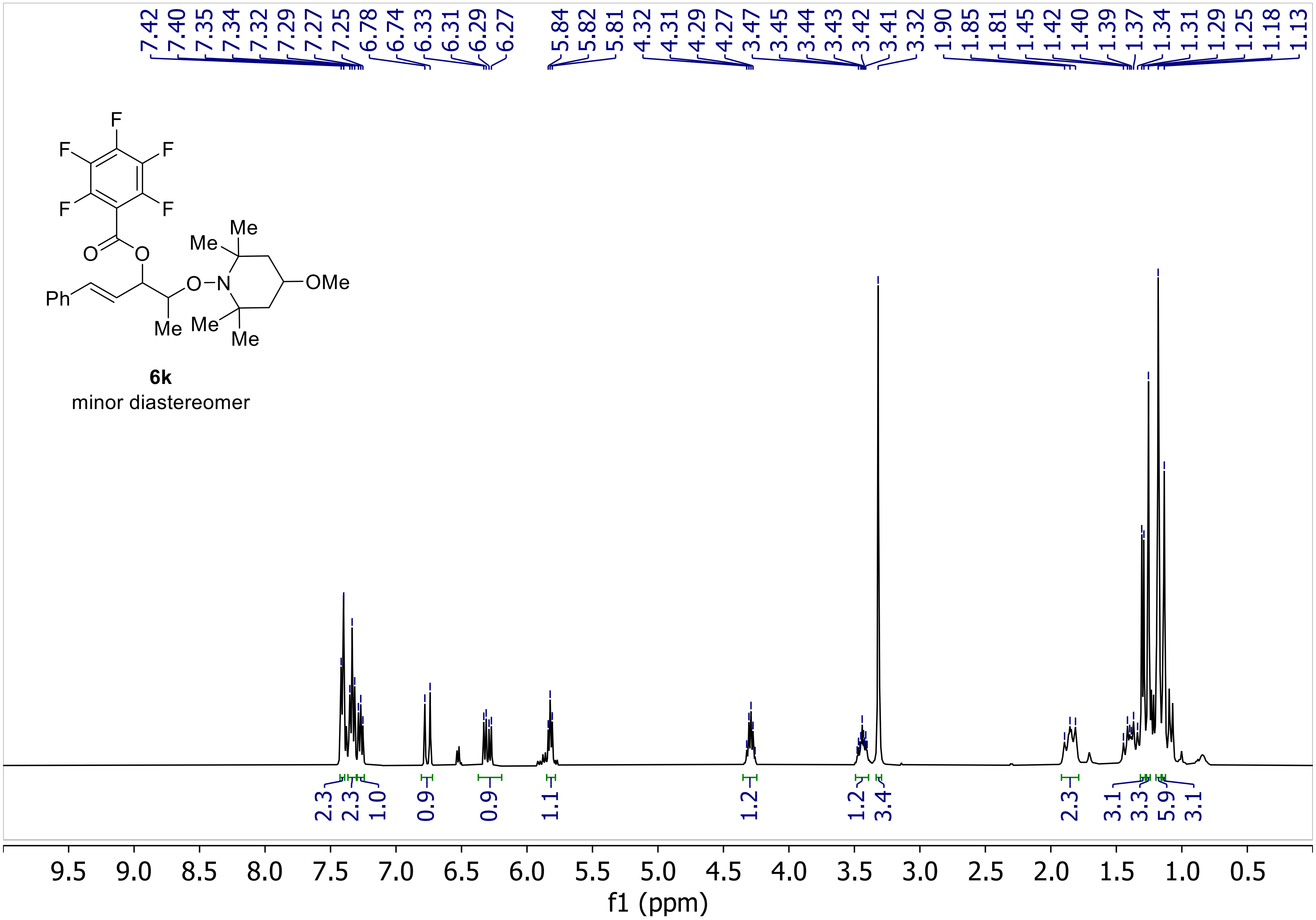

6k
minor diastereomer

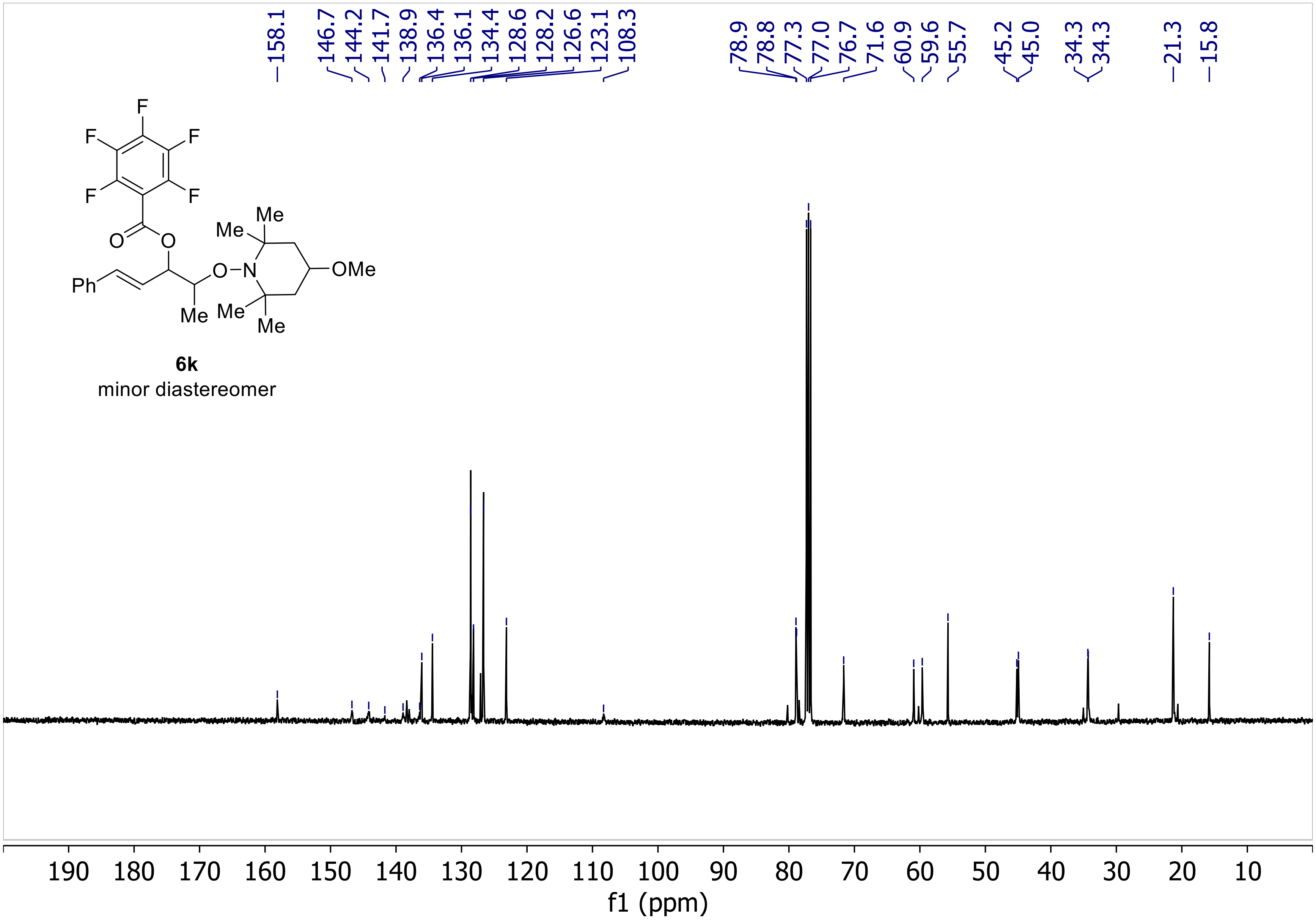

6k
minor diastereomer

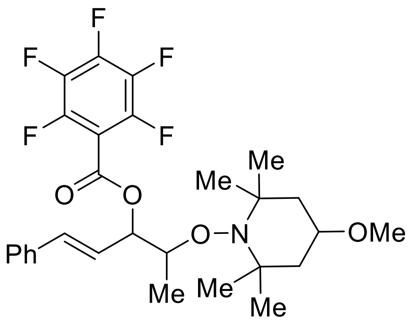

6k
minor diastereomer

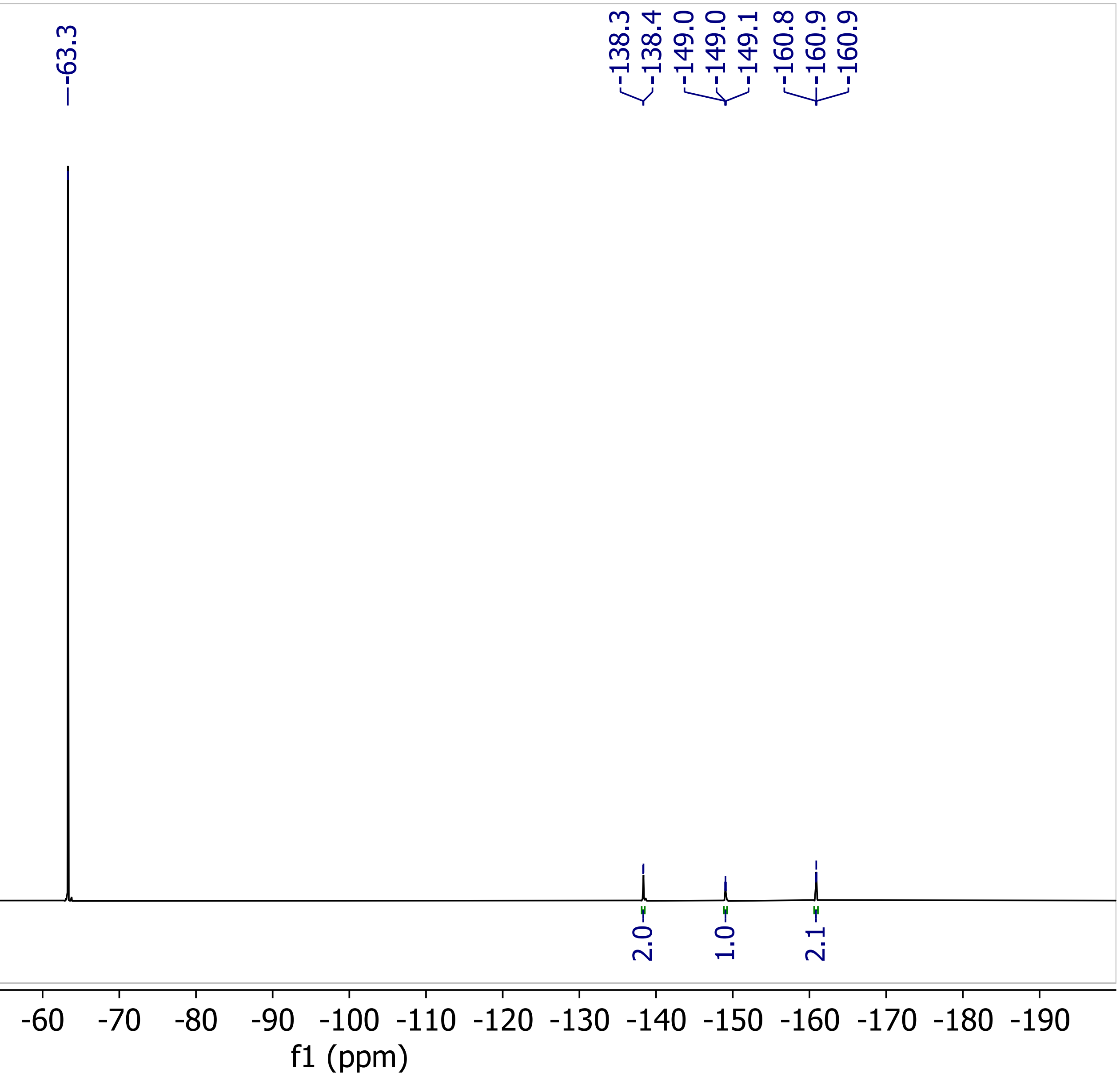

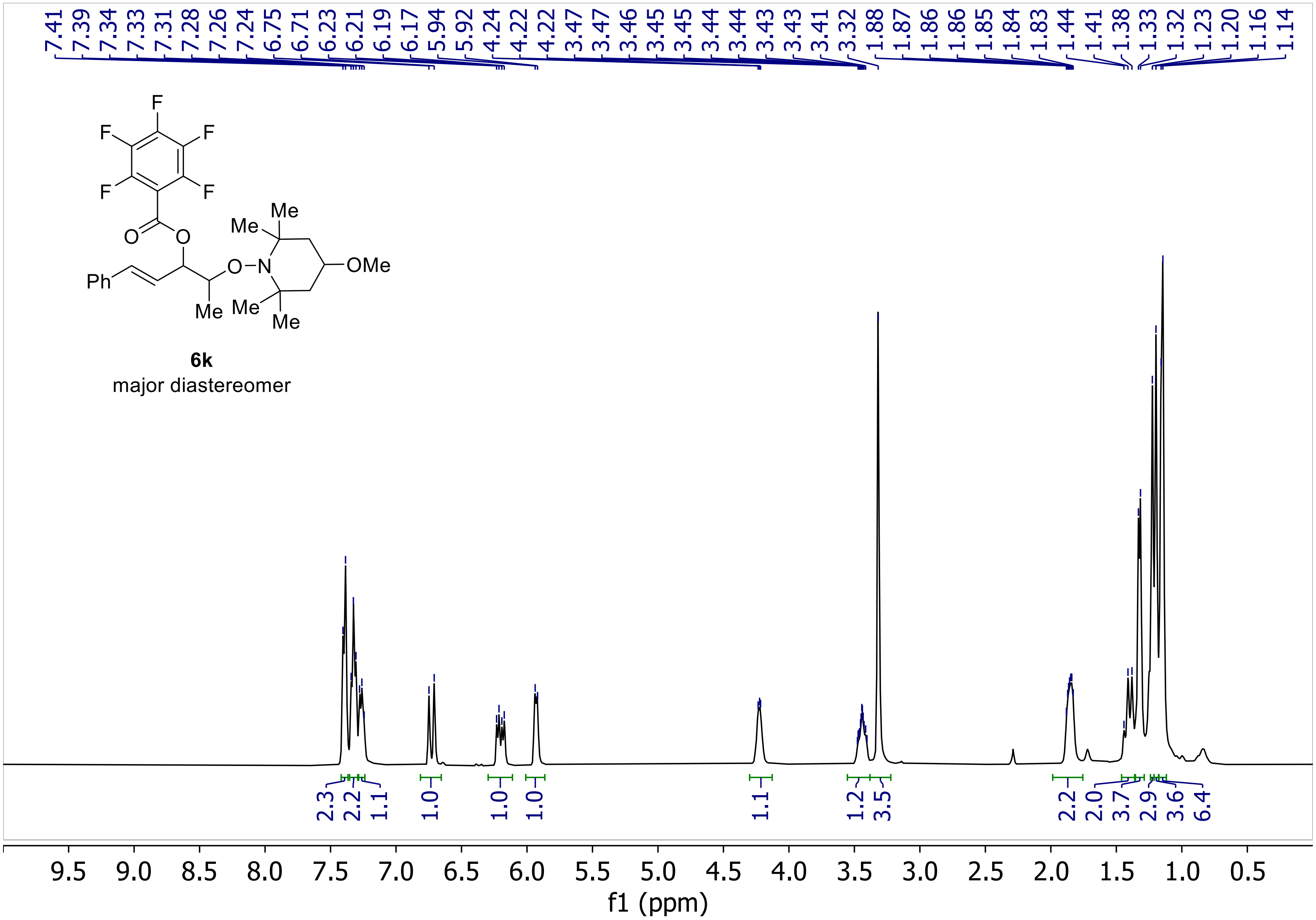

6k
major diastereomer

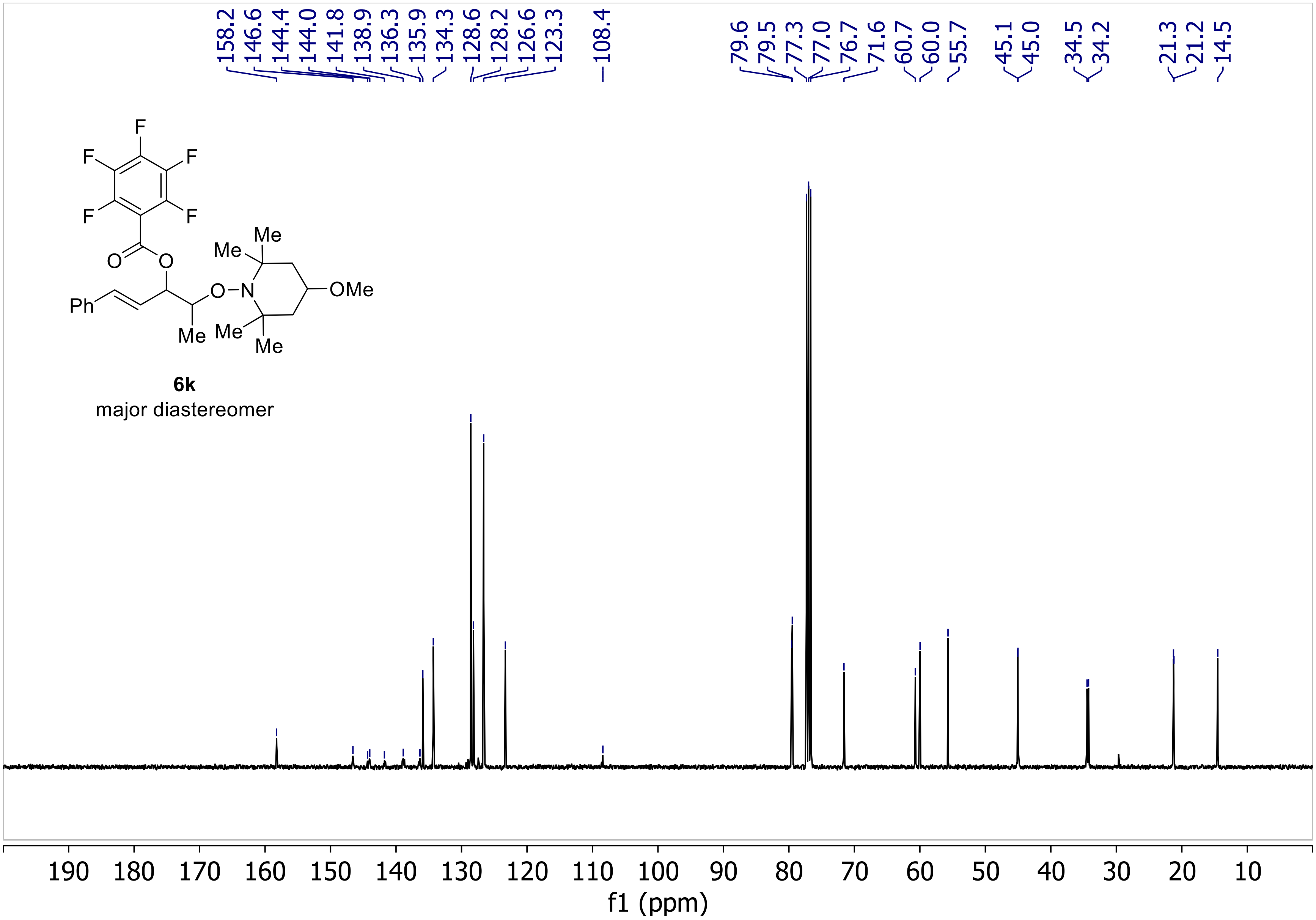

6k
major diastereomer

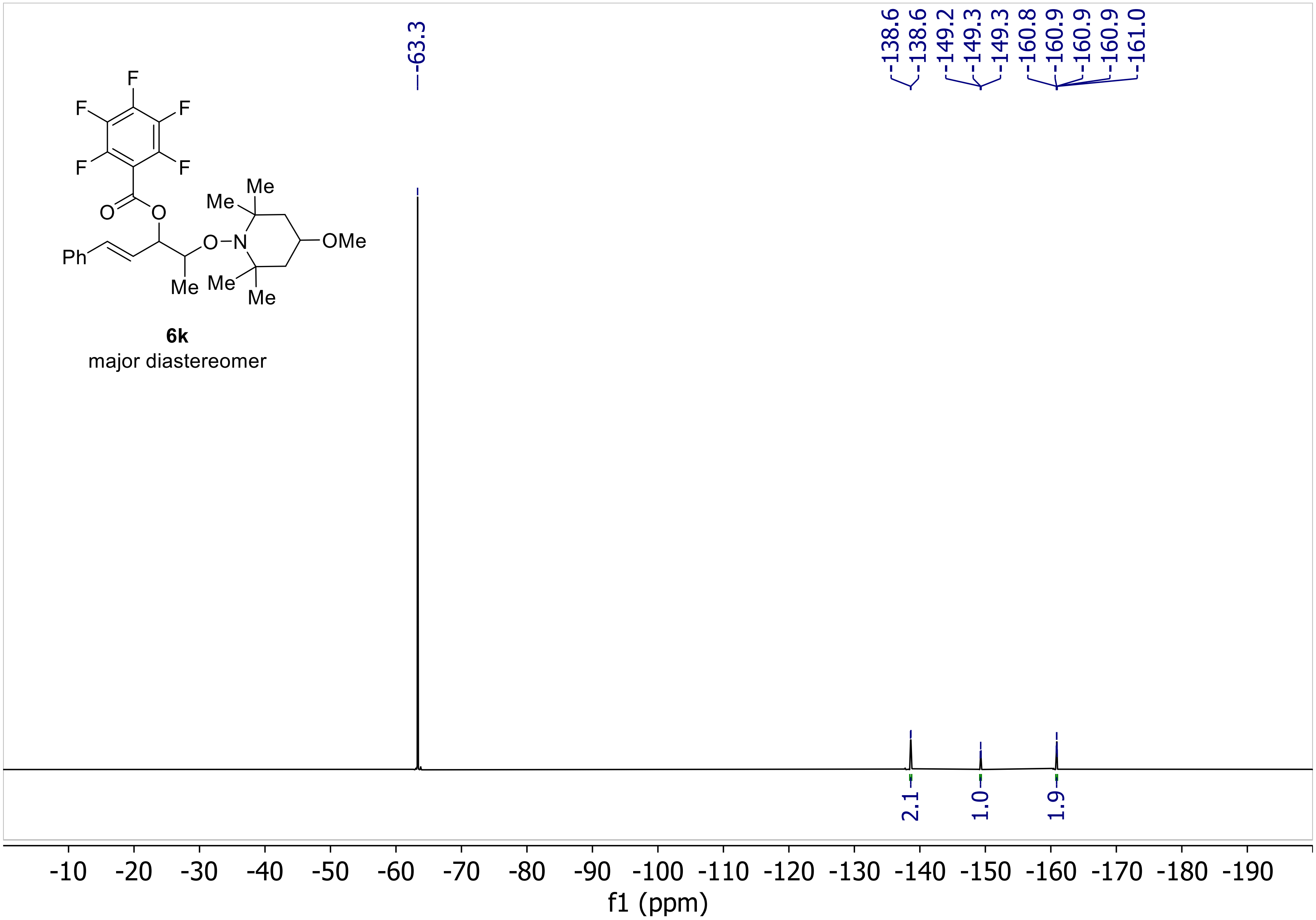
F
F
F
F
F
O
O
Ph
Me
O
N
Me
Me
Me
Me
OMe
6k
major diastereomer
-63.3
-138.6
-138.6
-149.2
-149.3
-149.3
-160.8
-160.9
-160.9
-160.9
-161.0
2.1
1.0
1.9
-10
-20
-30
-40
-50
-60
-70
-80
-90
-100
-110
-120
-130
-140
-150
-160
-170
-180
-190
f1 (ppm)

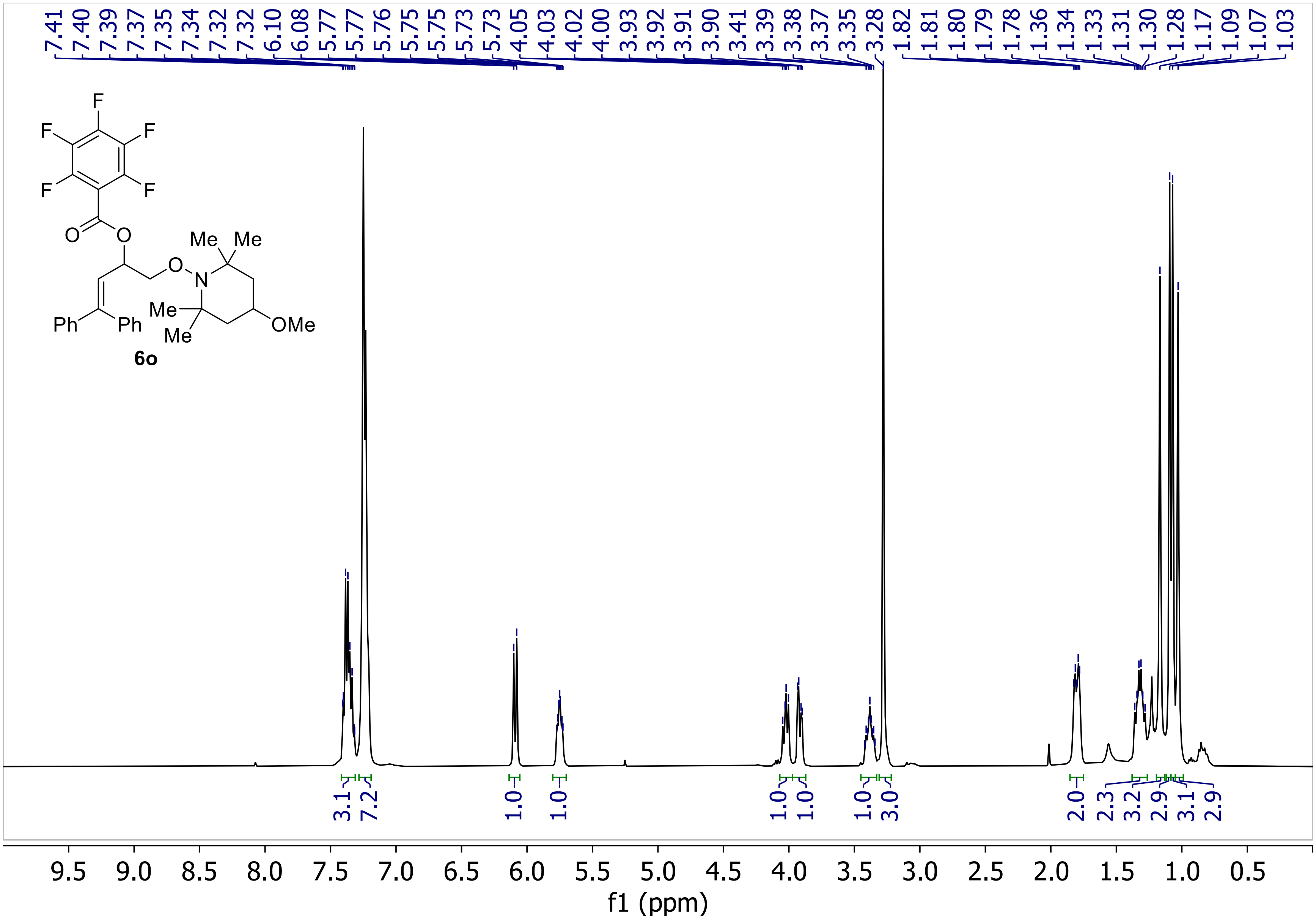
7.41 7.40 7.39 7.37 7.35 7.34 7.32 7.32 6.10 6.08 5.77 5.77 5.76 5.75 5.75 5.75 5.73 5.73 4.05 4.03 4.02 4.00 3.93 3.92 3.91 3.90 3.41 3.39 3.38 3.37 3.35 3.28 1.82 1.81 1.80 1.79 1.78 1.36 1.34 1.33 1.31 1.30 1.28 1.17 1.09 1.07 1.03
F
O
Me
N
OMe
Ph
6o
3.1
7.2
1.0
1.0
1.0
1.0
1.0
3.0
2.0
2.3
3.2
2.9
3.1
2.9
9.5 9.0 8.5 8.0 7.5 7.0 6.5 6.0 5.5 5.0 4.5 4.0 3.5 3.0 2.5 2.0 1.5 1.0 0.5
f1 (ppm)

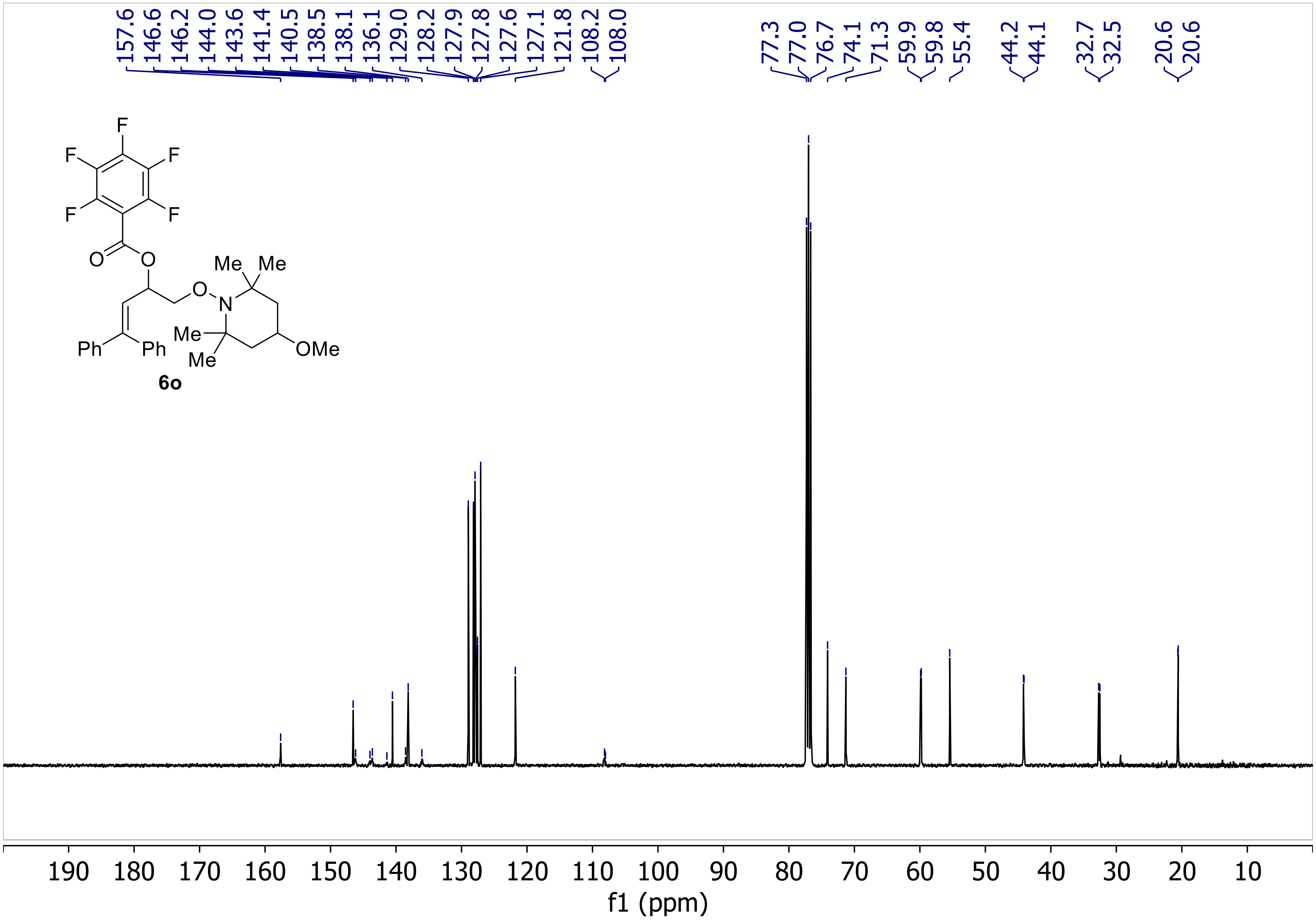
157.6
146.6
146.2
144.0
143.6
141.4
140.5
138.5
138.1
136.1
129.0
128.2
127.9
127.8
127.6
127.1
121.8
108.2
108.0
77.3
77.0
76.7
74.1
71.3
59.9
59.8
55.4
44.2
44.1
32.7
32.5
20.6
20.6
F
F
F
F
F
O
O
O
N
Me
Me
Me
Me
OMe
Ph
Ph
6o
190
180
170
160
150
140
130
120
110
100
90
80
70
60
50
40
30
20
10
f1 (ppm)

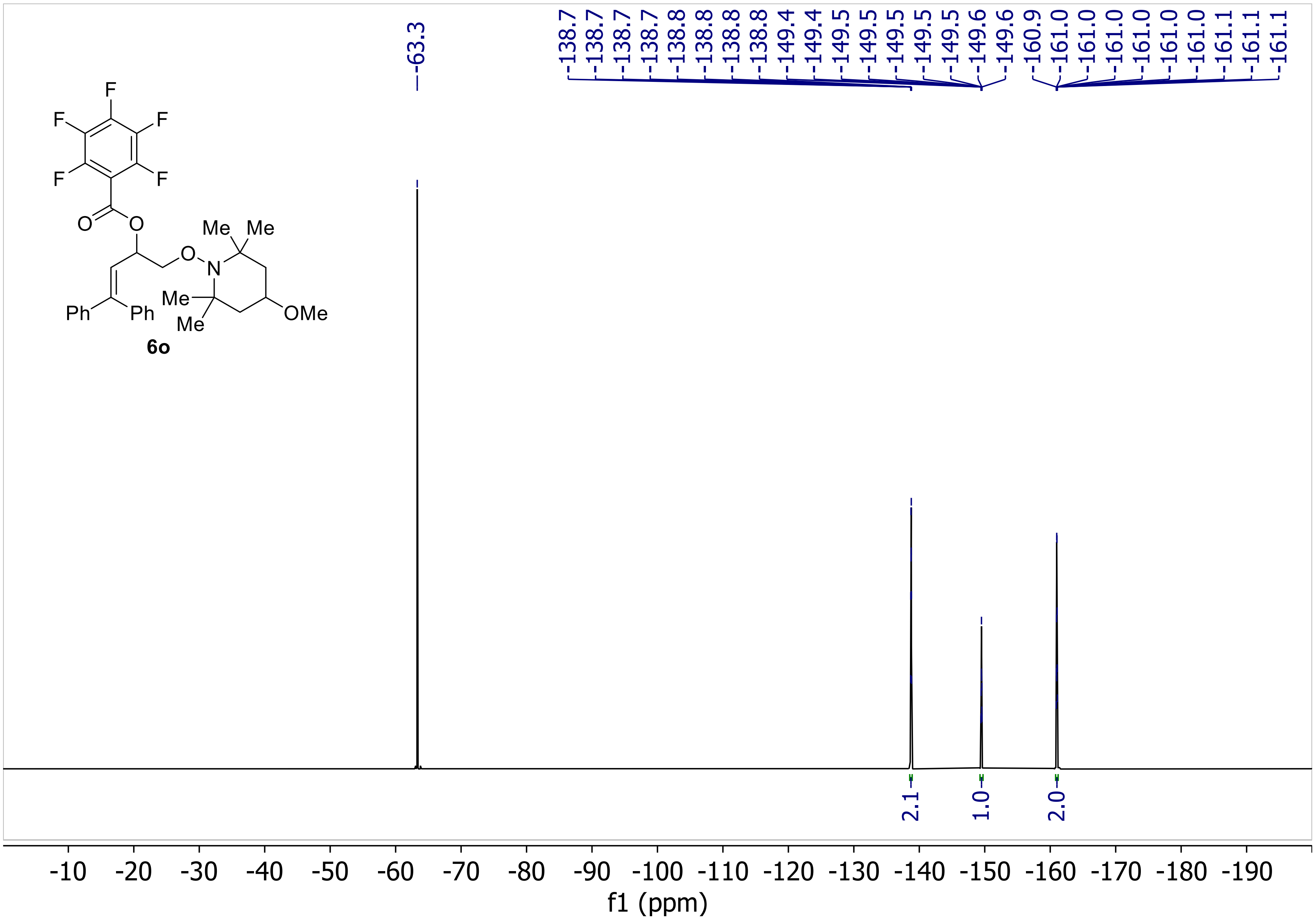
F
F
F
F
F
O
O
Me
Me
O
N
Me
Me
Ph
Ph
OMe
6o
-63.3
-138.7
-138.7
-138.7
-138.7
-138.8
-138.8
-138.8
-138.8
-149.4
-149.4
-149.5
-149.5
-149.5
-149.5
-149.5
-149.6
-149.6
-160.9
-161.0
-161.0
-161.0
-161.0
-161.0
-161.0
-161.1
-161.1
-161.1
2.1
1.0
2.0
-10 -20 -30 -40 -50 -60 -70 -80 -90 -100 -110 -120 -130 -140 -150 -160 -170 -180 -190
f1 (ppm)

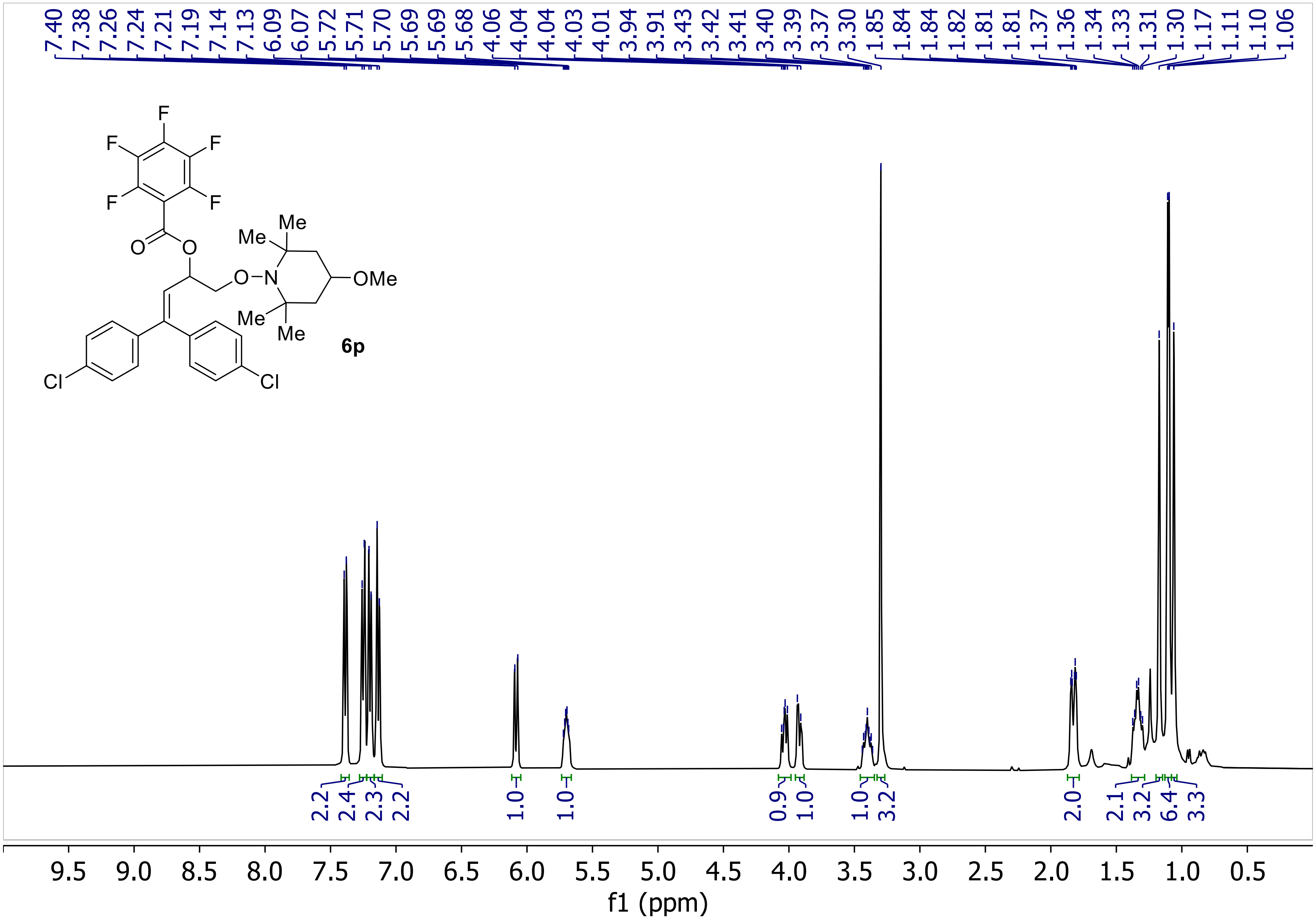
7.40
7.38
7.26
7.24
7.21
7.19
7.14
7.13
6.09
6.07
5.72
5.71
5.70
5.69
5.69
5.68
4.06
4.04
4.04
4.03
4.01
3.94
3.91
3.43
3.42
3.41
3.40
3.39
3.37
3.30
1.85
1.84
1.84
1.82
1.81
1.81
1.37
1.36
1.34
1.33
1.31
1.30
1.17
1.11
1.10
1.06
F
F
F
F
F
O
O
O–N
Me
Me
Me
Me
OMe
Cl
Cl
6p
2.2
2.4
2.3
2.2
1.0
1.0
0.9
1.0
1.0
3.2
2.0
2.1
3.2
6.4
3.3
9.5
9.0
8.5
8.0
7.5
7.0
6.5
6.0
5.5
5.0
4.5
4.0
3.5
3.0
2.5
2.0
1.5
1.0
0.5
f1 (ppm)

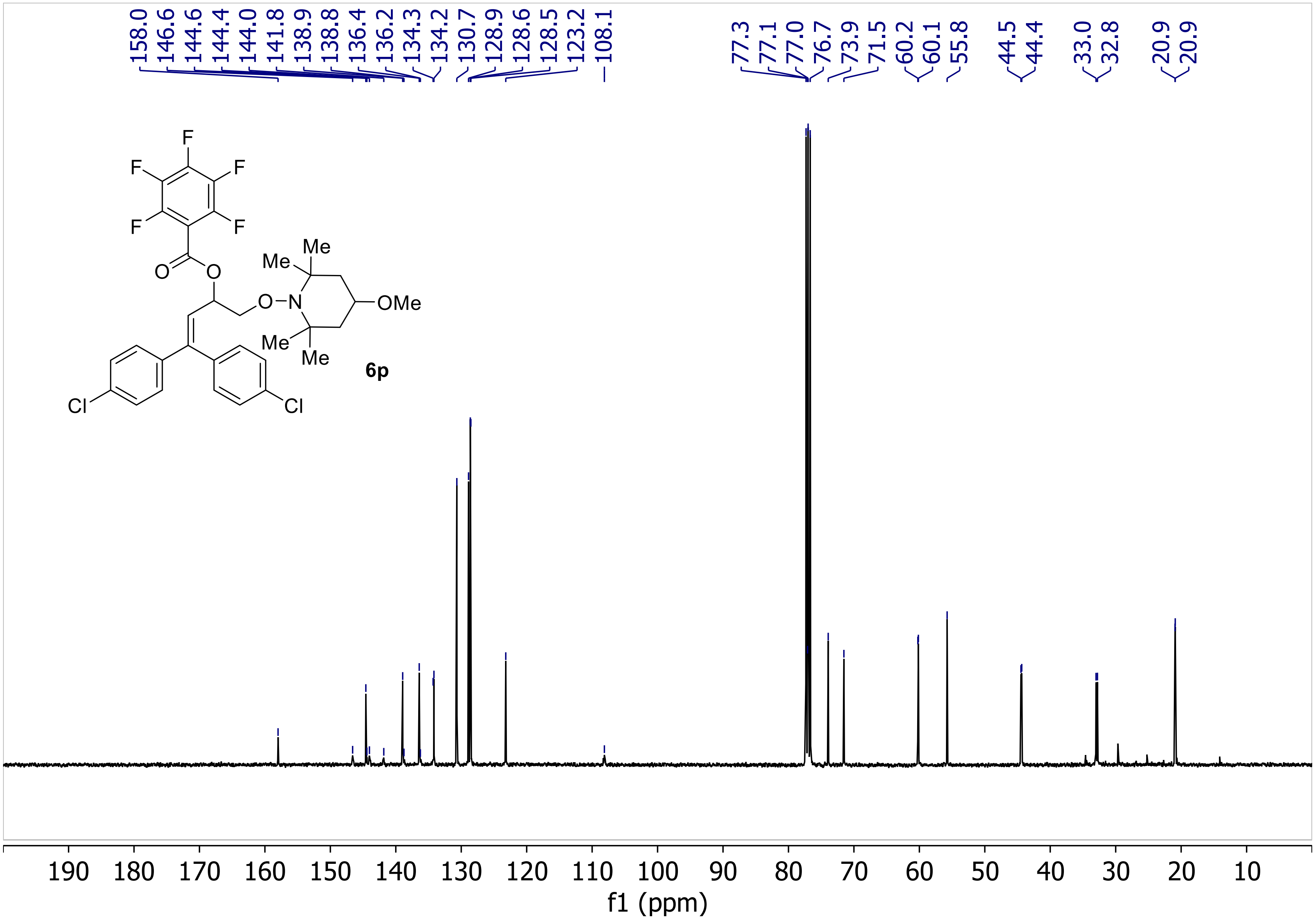

158.0
146.6
144.6
144.4
144.0
141.8
138.9
138.8
136.4
136.2
134.3
134.2
130.7
128.9
128.6
128.5
123.2
108.1
77.3
77.1
77.0
76.7
73.9
71.5
60.2
60.1
55.8
44.5
44.4
33.0
32.8
20.9
20.9
F
F
F
F
F
O
O
O
N
Me
Me
Me
Me
OMe
Cl
Cl
6p
190
180
170
160
150
140
130
120
110
100
90
80
70
60
50
40
30
20
10
f1 (ppm)

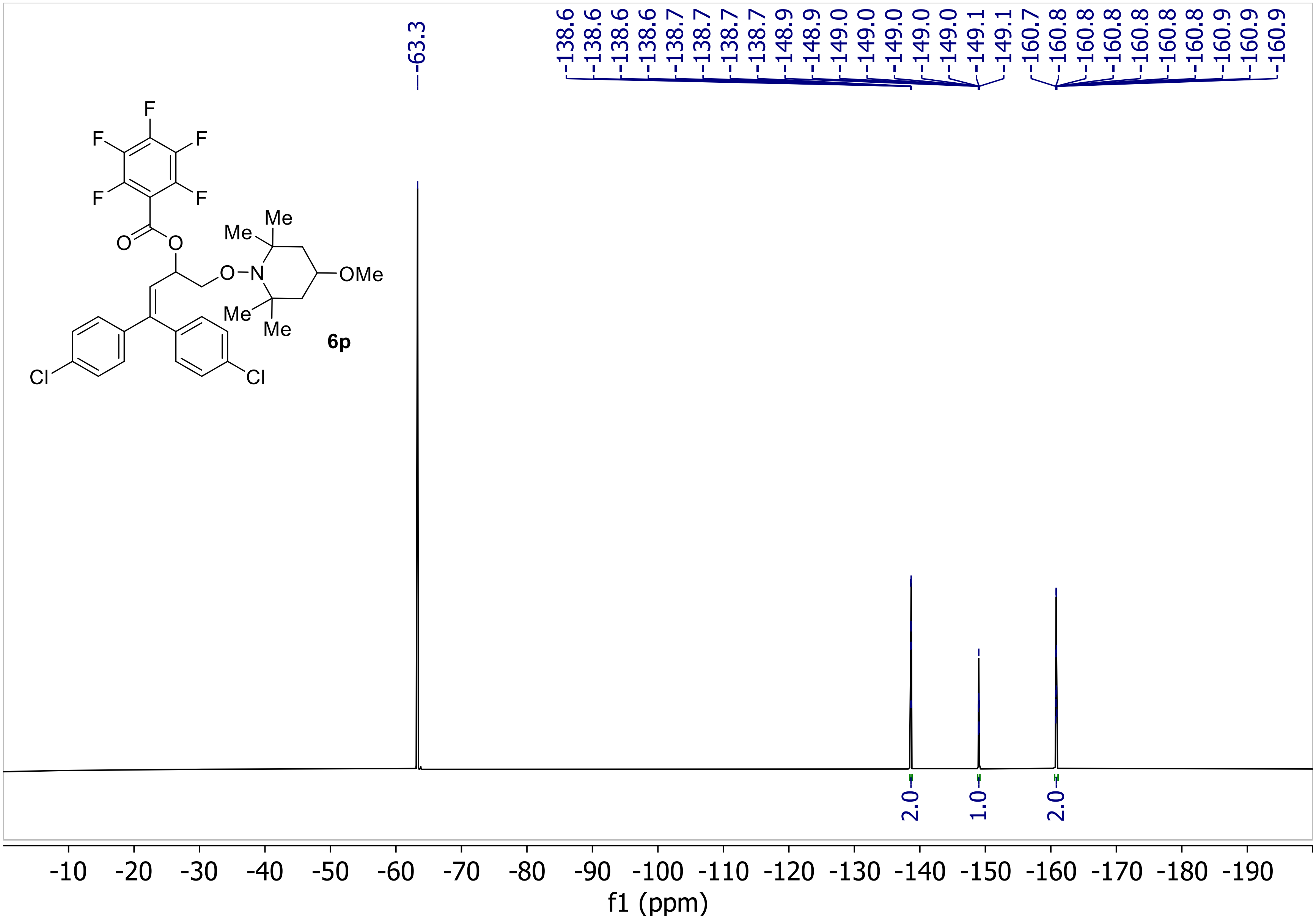
-63.3
-138.6
-138.6
-138.6
-138.6
-138.7
-138.7
-138.7
-138.7
-148.9
-148.9
-149.0
-149.0
-149.0
-149.0
-149.0
-149.1
-149.1
-160.7
-160.8
-160.8
-160.8
-160.8
-160.8
-160.8
-160.9
-160.9
-160.9
F
F
F
F
F
O
O
Me
Me
O
N
OMe
Me
Me
Cl
Cl
6p
2.0
1.0
2.0
-10 -20 -30 -40 -50 -60 -70 -80 -90 -100 -110 -120 -130 -140 -150 -160 -170 -180 -190
f1 (ppm)

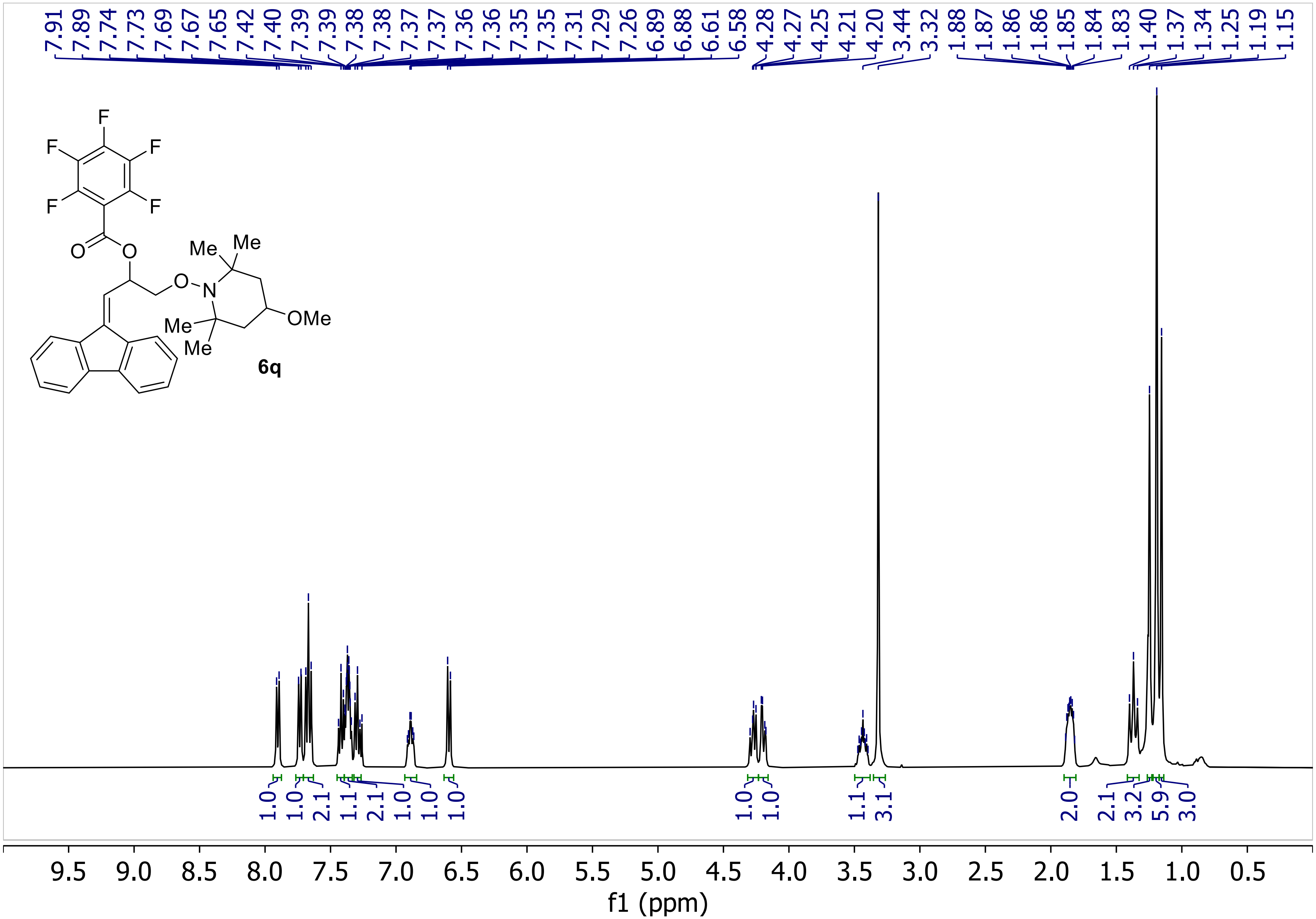
7.91 7.89 7.74 7.73 7.69 7.67 7.65 7.42 7.40 7.39 7.39 7.38 7.38 7.37 7.37 7.36 7.36 7.35 7.35 7.31 7.29 7.26 6.89 6.88 6.61 6.58 4.28 4.27 4.25 4.21 4.20 3.44 3.32 1.88 1.87 1.86 1.86 1.85 1.84 1.83 1.40 1.37 1.34 1.25 1.19 1.15
F
O
Me
N
OMe
6q
1.0 1.0 2.1 1.1 2.1 1.0 1.0 1.0 1.0 1.0 1.1 3.1 2.0 2.1 3.2 5.9 3.0
9.5 9.0 8.5 8.0 7.5 7.0 6.5 6.0 5.5 5.0 4.5 4.0 3.5 3.0 2.5 2.0 1.5 1.0 0.5
f1 (ppm)

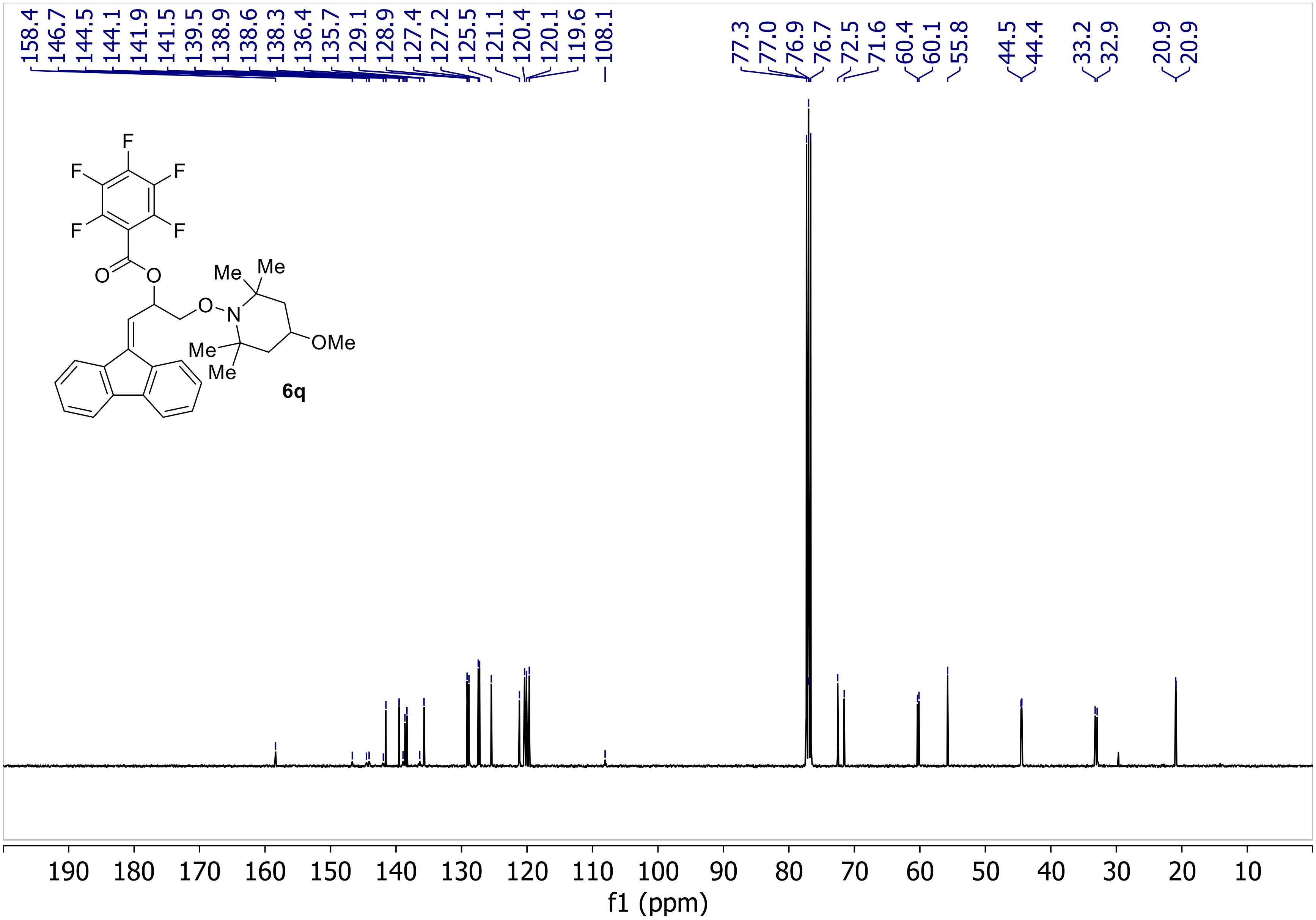
158.4
146.7
144.5
144.1
141.9
141.5
139.5
138.9
138.6
138.3
136.4
135.7
129.1
128.9
127.4
127.2
125.5
121.1
120.4
120.1
119.6
108.1
77.3
77.0
76.9
76.7
72.5
71.6
60.4
60.1
55.8
44.5
44.4
33.2
32.9
20.9
20.9
6q
190 180 170 160 150 140 130 120 110 100 90 80 70 60 50 40 30 20 10
f1 (ppm)

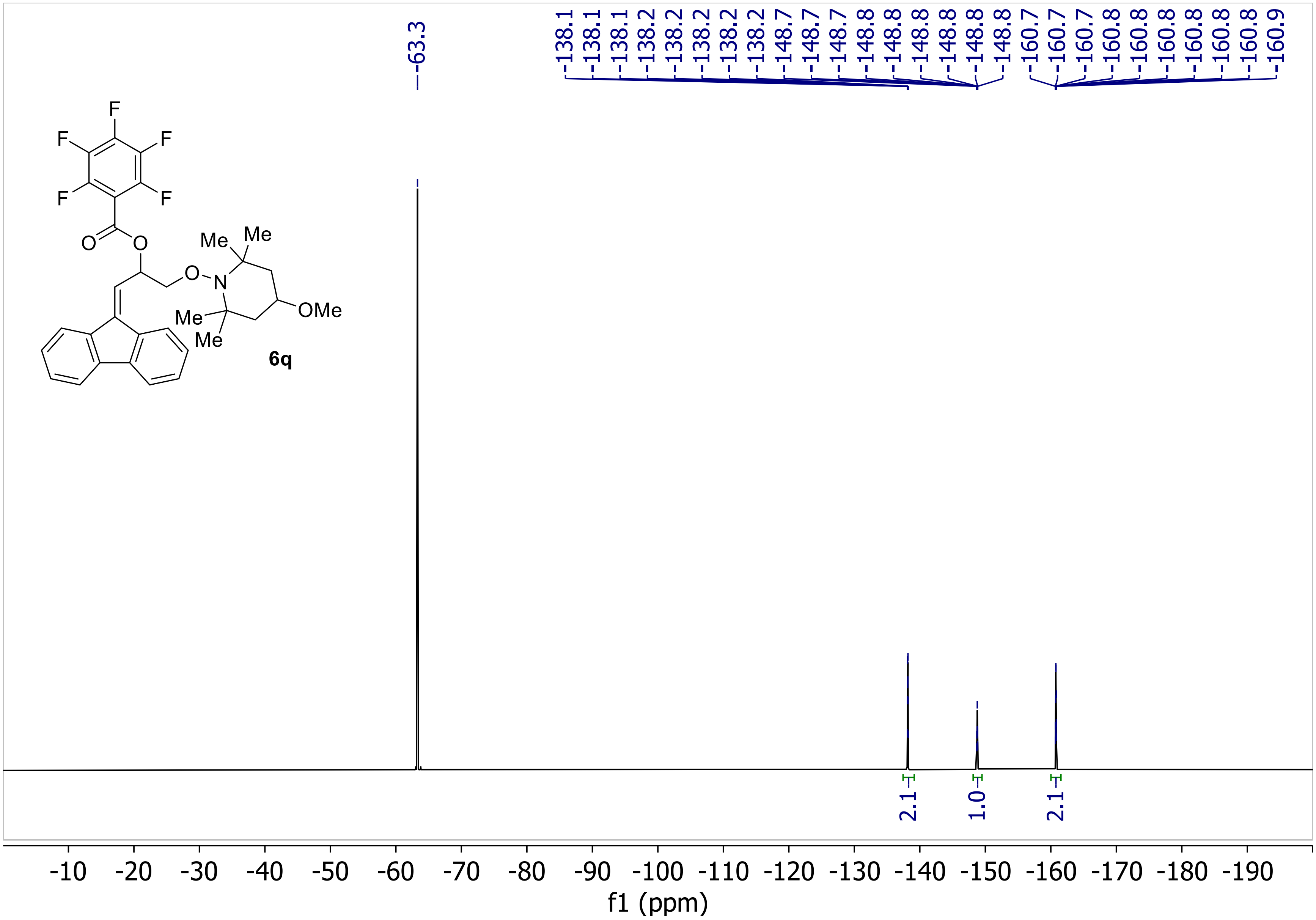
F
F
F
F
F
O
O
Me
Me
O
N
OMe
Me
Me
6q
-63.3
-138.1
-138.1
-138.1
-138.2
-138.2
-138.2
-138.2
-138.2
-148.7
-148.7
-148.7
-148.8
-148.8
-148.8
-148.8
-148.8
-148.8
-160.7
-160.7
-160.7
-160.8
-160.8
-160.8
-160.8
-160.8
-160.8
-160.9
2.1
1.0
2.1
-10 -20 -30 -40 -50 -60 -70 -80 -90 -100 -110 -120 -130 -140 -150 -160 -170 -180 -190
f1 (ppm)

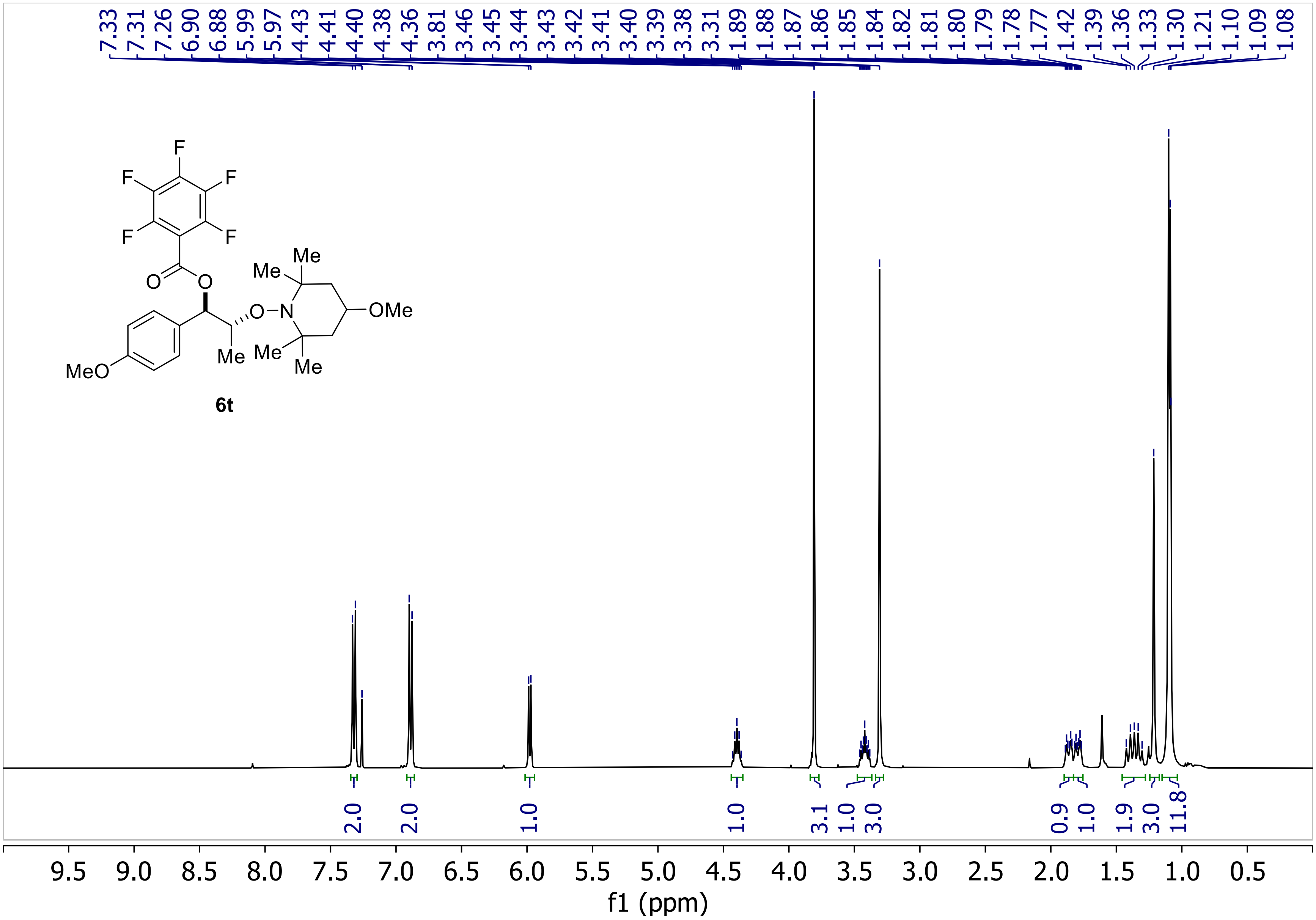
7.33 7.31 7.26 6.90 6.88 5.99 5.97 4.43 4.41 4.40 4.38 4.36 3.81 3.46 3.45 3.44 3.43 3.42 3.41 3.40 3.39 3.38 3.31 1.89 1.88 1.87 1.86 1.85 1.84 1.82 1.81 1.80 1.79 1.78 1.77 1.42 1.39 1.36 1.33 1.30 1.21 1.10 1.09 1.08
F
F
F
F
F
O
O
Me
Me
O
N
OMe
Me
Me
Me
MeO
6t
2.0 2.0 1.0 1.0 3.1 1.0 3.0 0.9 1.0 1.9 3.0 11.8
9.5 9.0 8.5 8.0 7.5 7.0 6.5 6.0 5.5 5.0 4.5 4.0 3.5 3.0 2.5 2.0 1.5 1.0 0.5
f1 (ppm)

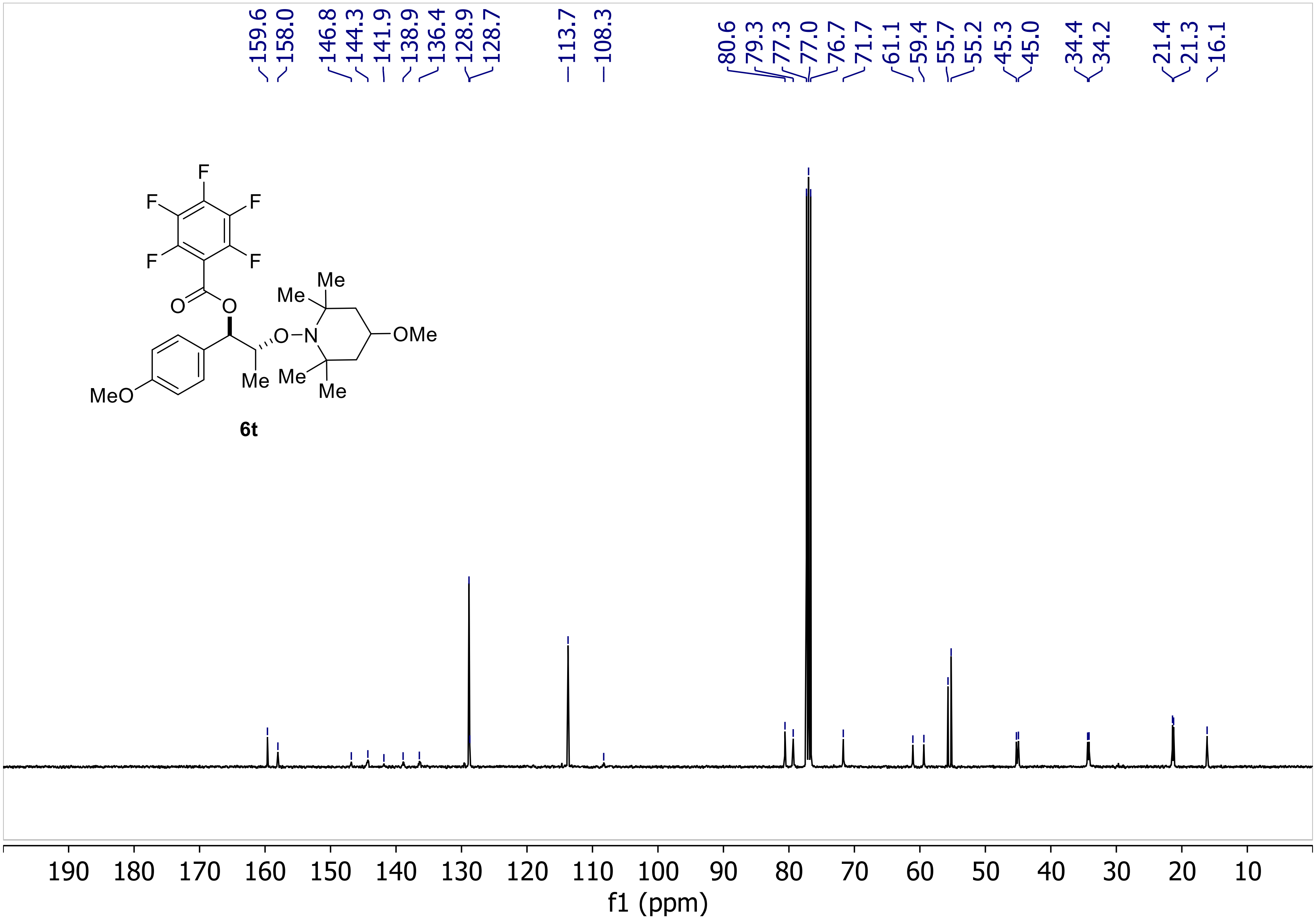
159.6
158.0
146.8
144.3
141.9
138.9
136.4
128.9
128.7
113.7
108.3
80.6
79.3
77.3
77.0
76.7
71.7
61.1
59.4
55.7
55.2
45.3
45.0
34.4
34.2
21.4
21.3
16.1
F
F
F
F
F
O
O
O
N
OMe
Me
Me
Me
Me
Me
MeO
6t
190 180 170 160 150 140 130 120 110 100 90 80 70 60 50 40 30 20 10
f1 (ppm)

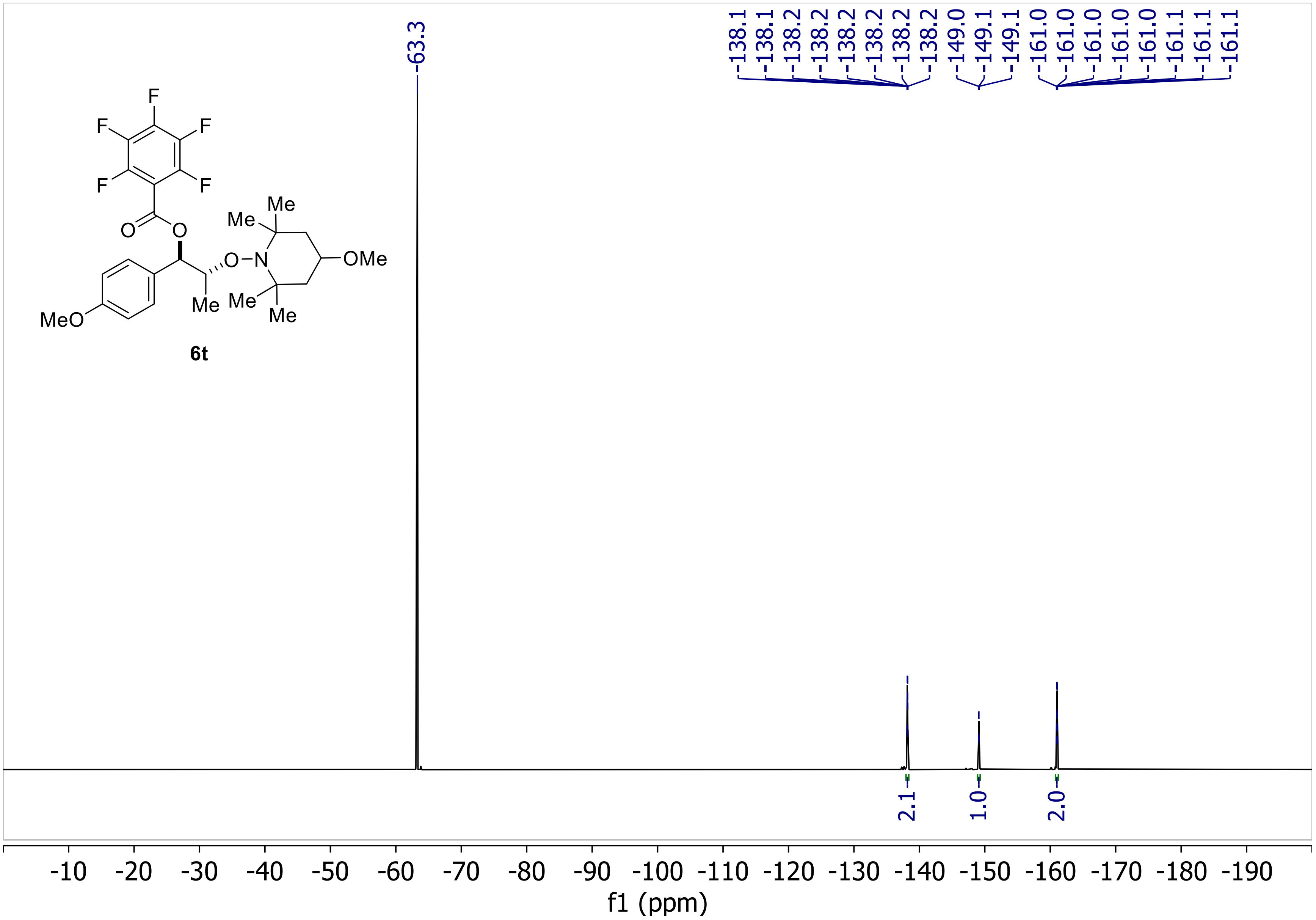
-63.3
-138.1
-138.1
-138.2
-138.2
-138.2
-138.2
-138.2
-138.2
-149.0
-149.1
-149.1
-161.0
-161.0
-161.0
-161.0
-161.0
-161.1
-161.1
-161.1
F
F
F
F
F
O
O
Me
Me
O–N
OMe
MeO
Me
Me
Me
6t
2.1
1.0
2.0
-10 -20 -30 -40 -50 -60 -70 -80 -90 -100 -110 -120 -130 -140 -150 -160 -170 -180 -190
f1 (ppm)

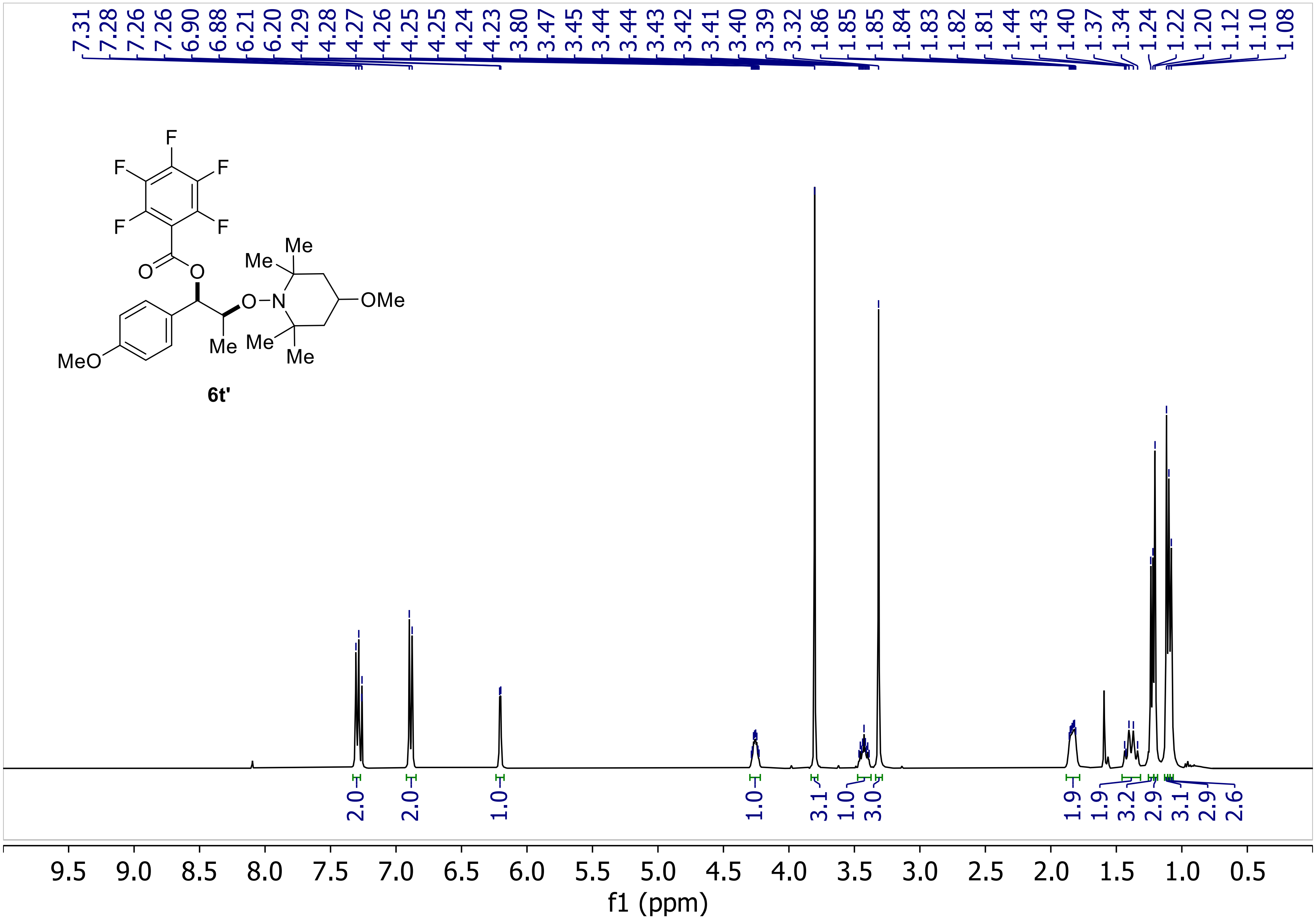
7.31
7.28
7.26
7.26
6.90
6.88
6.21
6.20
4.29
4.28
4.27
4.26
4.25
4.25
4.24
4.23
3.80
3.47
3.45
3.44
3.44
3.43
3.42
3.41
3.40
3.39
3.32
1.86
1.85
1.85
1.84
1.83
1.82
1.81
1.44
1.43
1.40
1.37
1.34
1.24
1.22
1.20
1.12
1.10
1.08
6t'
2.0
2.0
1.0
1.0
3.1
1.0
3.0
1.9
1.9
3.2
2.9
3.1
2.9
2.6
9.5
9.0
8.5
8.0
7.5
7.0
6.5
6.0
5.5
5.0
4.5
4.0
3.5
3.0
2.5
2.0
1.5
1.0
0.5
f1 (ppm)

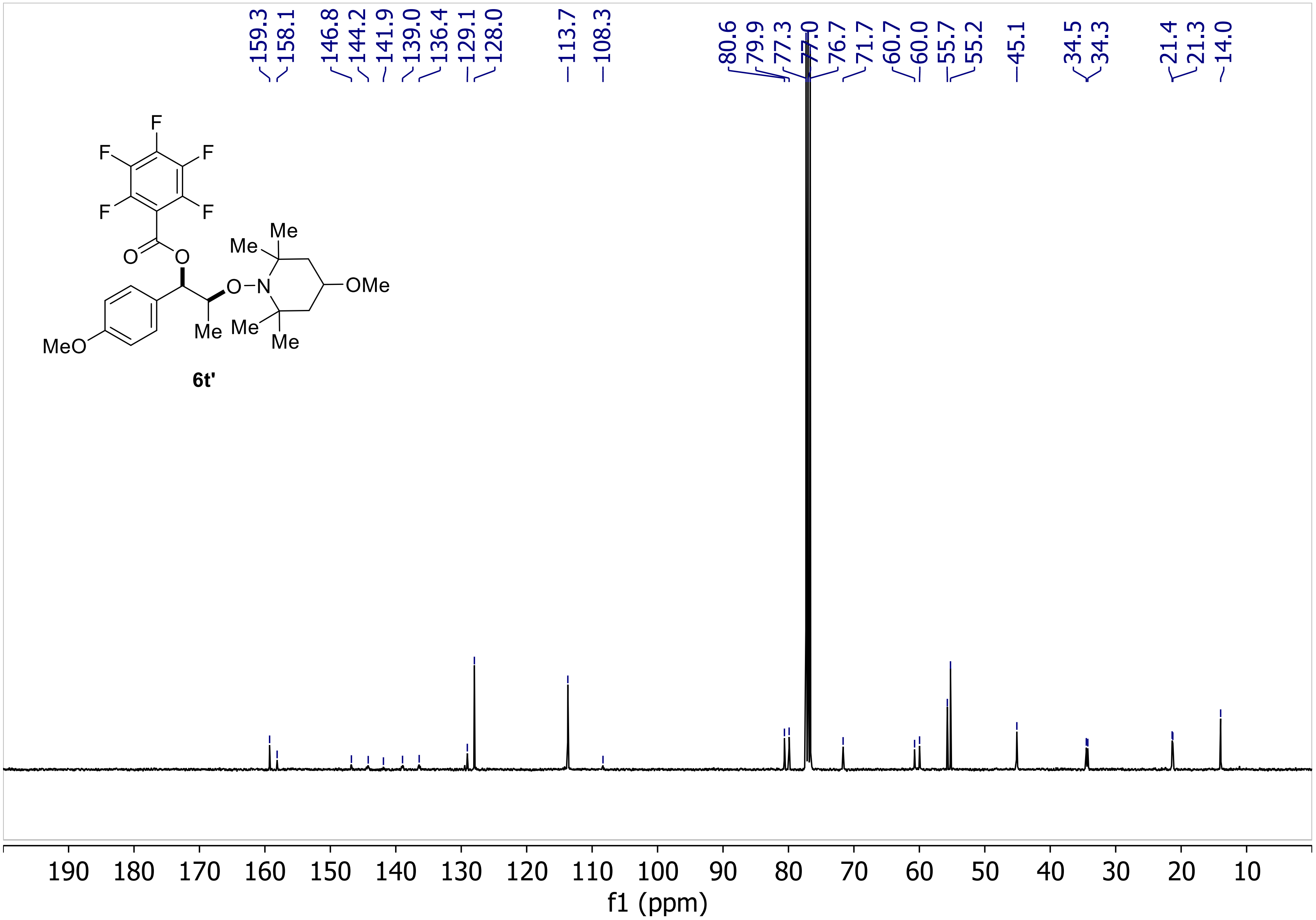

159.3
158.1
146.8
144.2
141.9
139.0
136.4
129.1
128.0
113.7
108.3
80.6
79.9
77.3
77.0
76.7
71.7
60.7
60.0
55.7
55.2
45.1
34.5
34.3
21.4
21.3
14.0
F
F
F
F
F
O
O
Me
Me
O
N
OMe
MeO
Me
Me
Me
6t'
190 180 170 160 150 140 130 120 110 100 90 80 70 60 50 40 30 20 10
f1 (ppm)

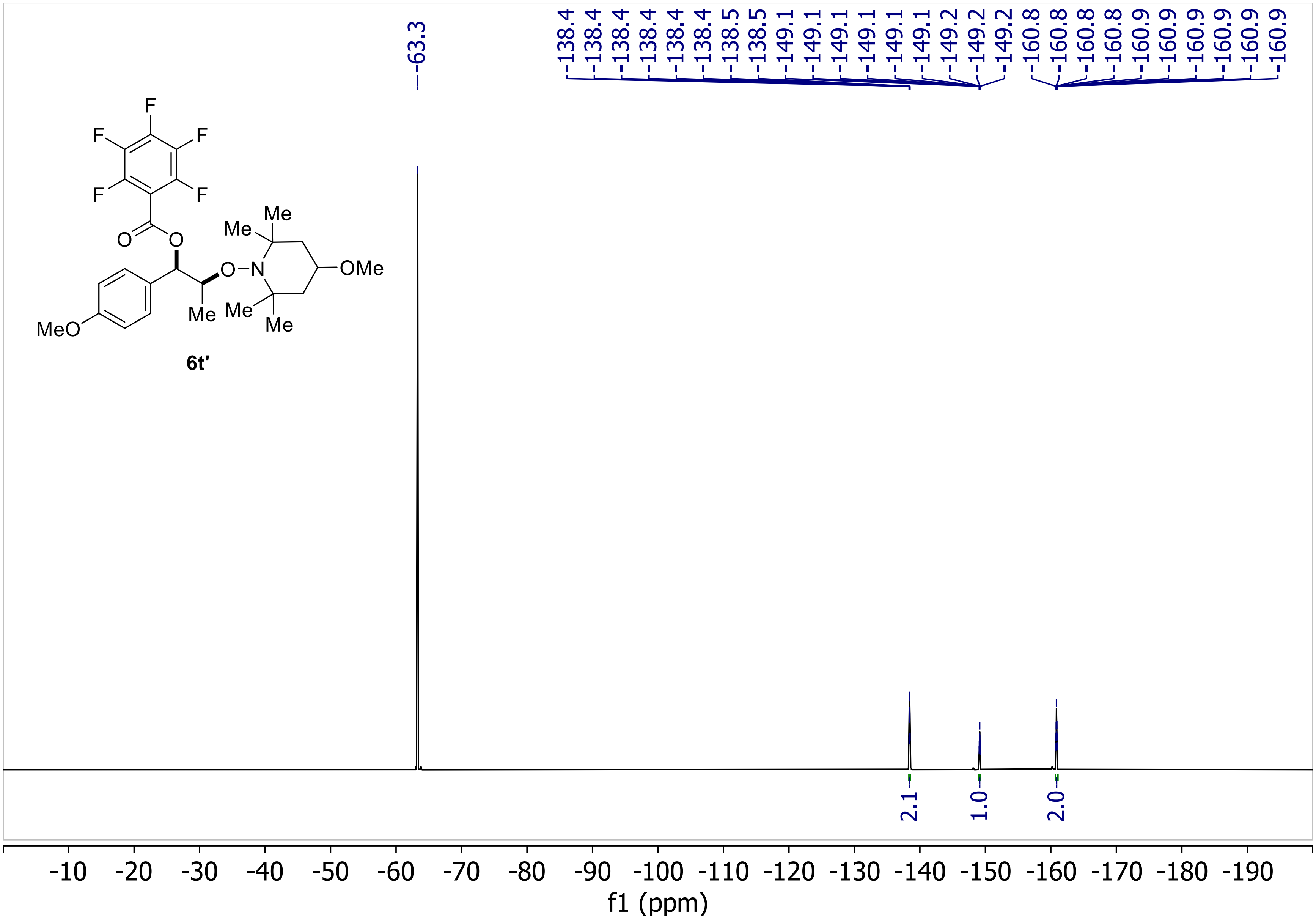
F
F
F
F
F
O
O
Me
Me
O
N
OMe
Me
Me
Me
MeO
-63.3
-138.4
-138.4
-138.4
-138.4
-138.4
-138.4
-138.5
-138.5
-149.1
-149.1
-149.1
-149.1
-149.1
-149.1
-149.2
-149.2
-149.2
-160.8
-160.8
-160.8
-160.8
-160.9
-160.9
-160.9
-160.9
-160.9
-160.9
2.1
1.0
2.0
-10 -20 -30 -40 -50 -60 -70 -80 -90 -100 -110 -120 -130 -140 -150 -160 -170 -180 -190
f1 (ppm)

6t'

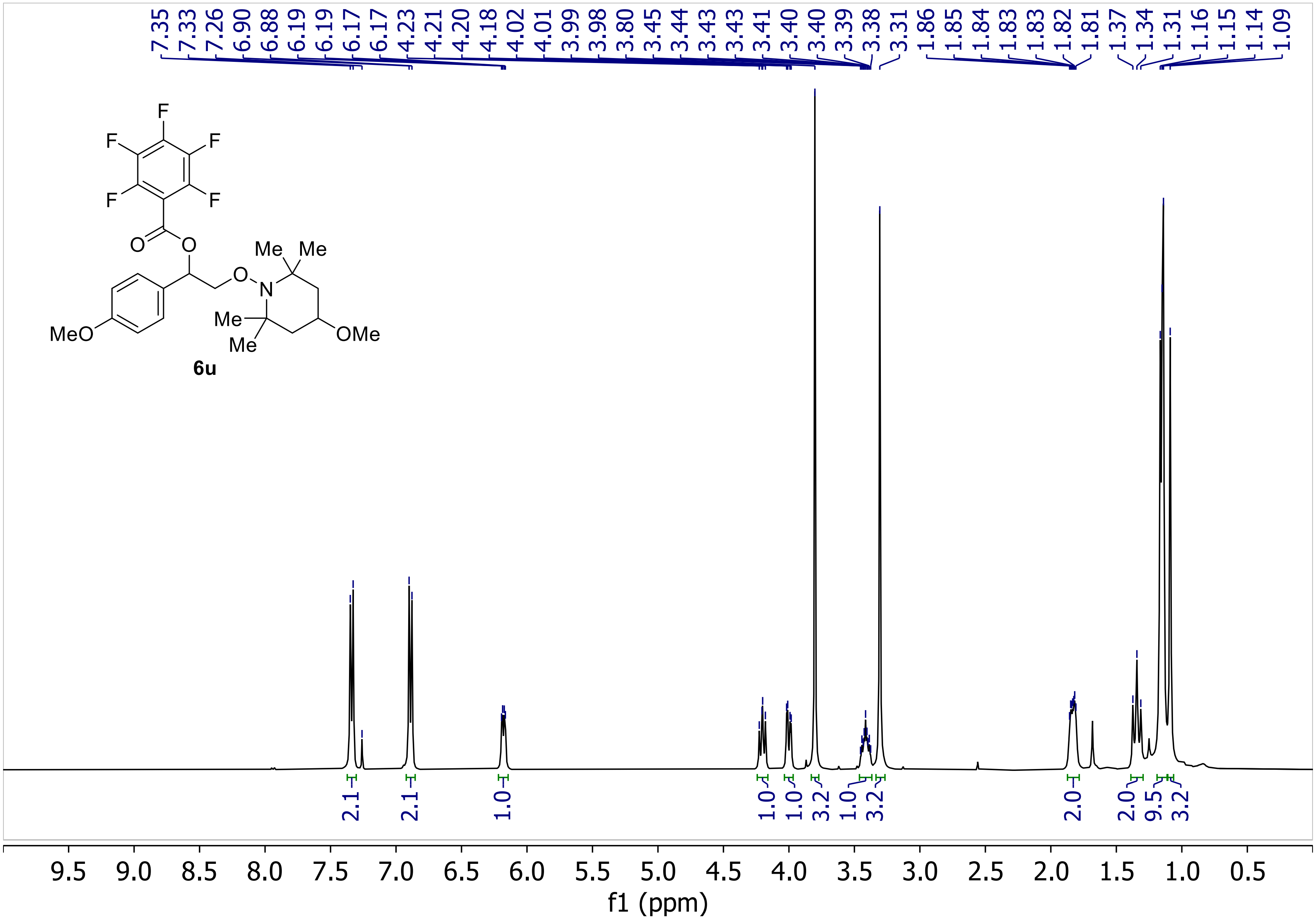
7.35
7.33
7.26
6.90
6.88
6.19
6.19
6.17
6.17
4.23
4.21
4.20
4.18
4.02
4.01
3.99
3.98
3.80
3.45
3.44
3.43
3.43
3.41
3.40
3.40
3.39
3.38
3.31
1.86
1.85
1.84
1.83
1.83
1.82
1.81
1.37
1.34
1.31
1.16
1.15
1.14
1.09
F
F
F
F
F
O
O
Me
Me
O
N
MeO
Me
Me
OMe
6u
2.1
2.1
1.0
1.0
1.0
3.2
1.0
3.2
2.0
2.0
9.5
3.2
9.5
9.0
8.5
8.0
7.5
7.0
6.5
6.0
5.5
5.0
4.5
4.0
3.5
3.0
2.5
2.0
1.5
1.0
0.5
f1 (ppm)

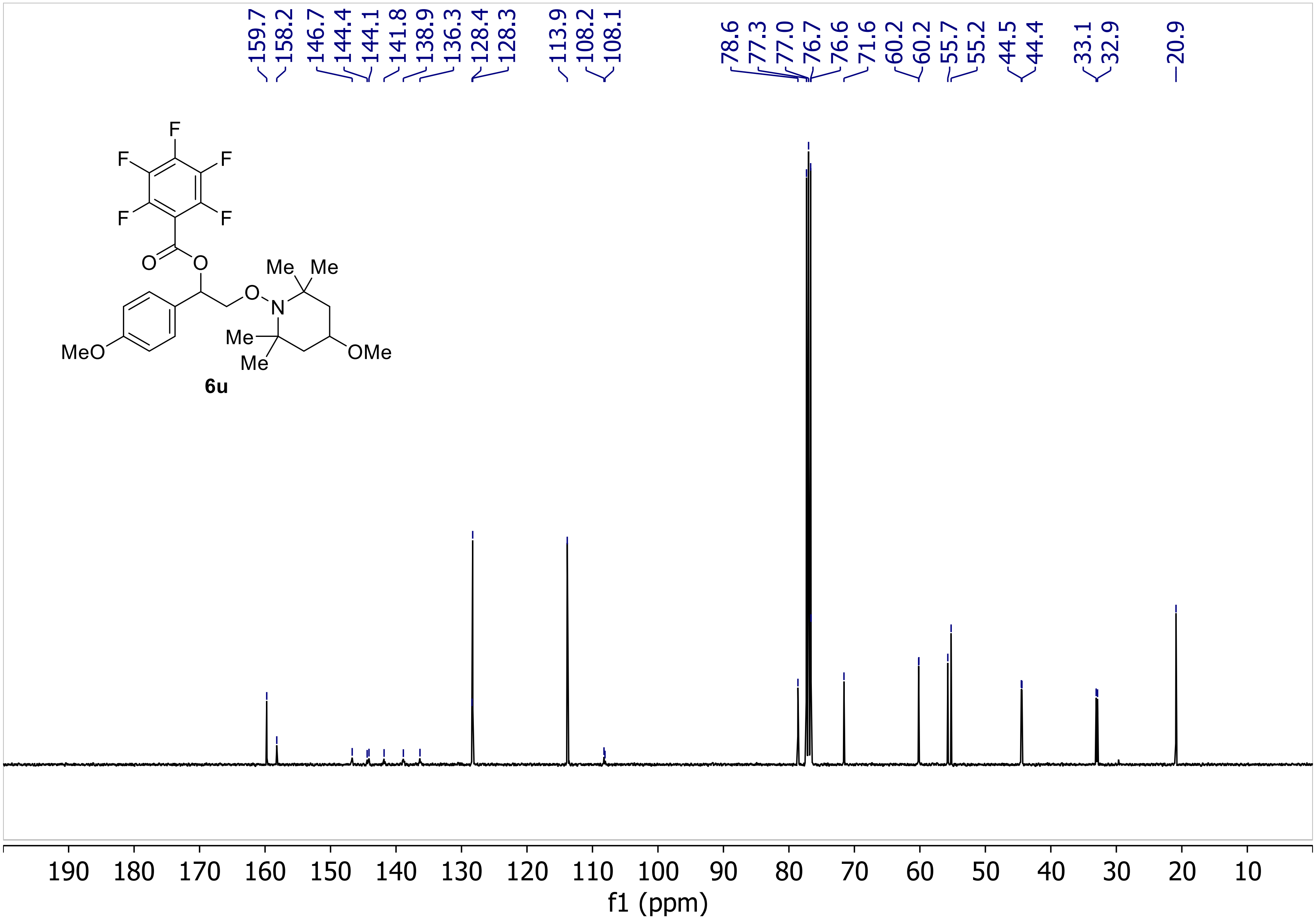
159.7
158.2
146.7
144.4
144.1
141.8
138.9
136.3
128.4
128.3
113.9
108.2
108.1
78.6
77.3
77.0
76.7
76.6
71.6
60.2
60.2
55.7
55.2
44.5
44.4
33.1
32.9
20.9
F
O
Me
OMe
MeO
N
190 180 170 160 150 140 130 120 110 100 90 80 70 60 50 40 30 20 10
f1 (ppm)

6u

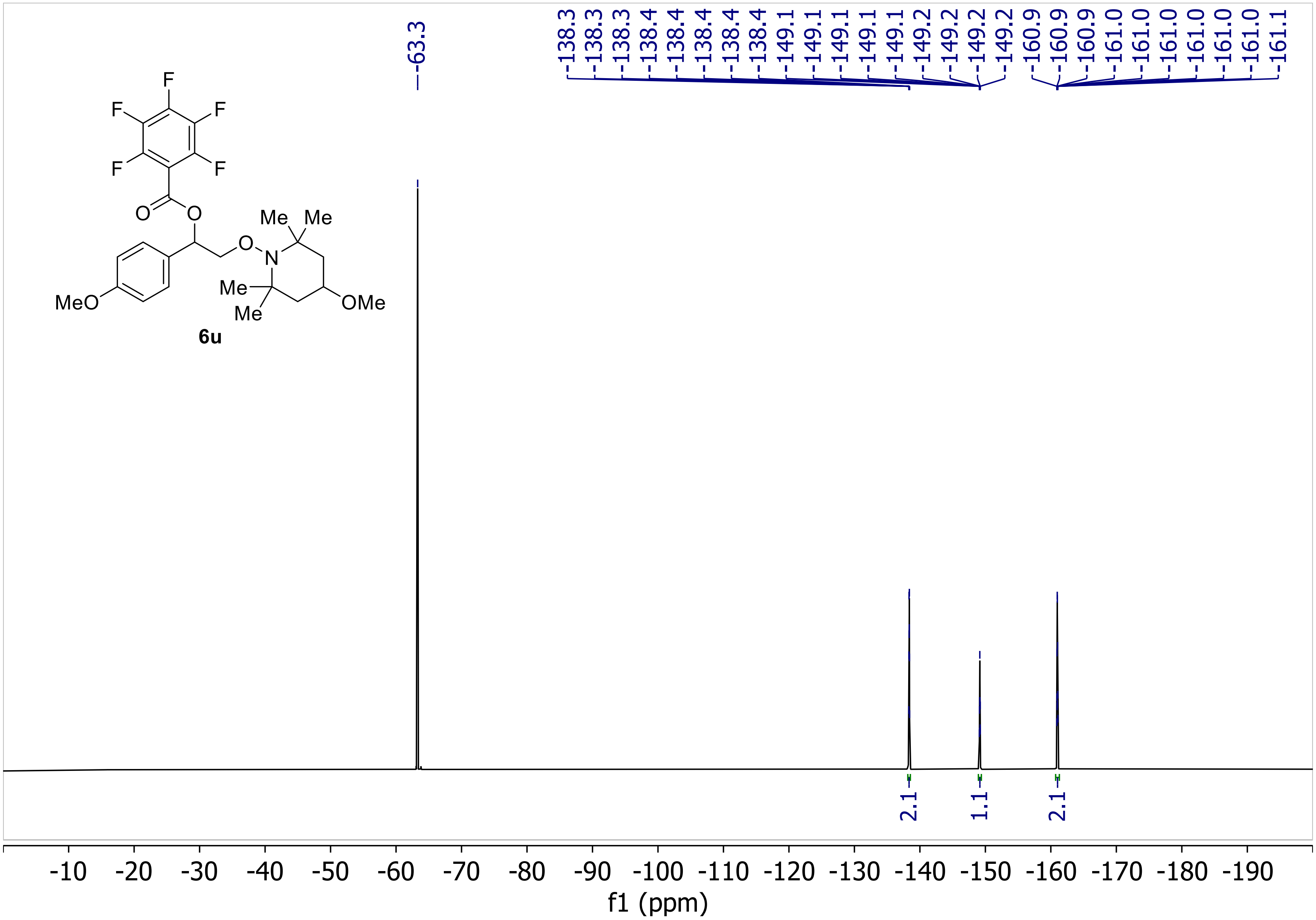

F
F
F
F
F
O
O
Me
Me
O
N
Me
Me
MeO
OMe
6u
-63.3
-138.3
-138.3
-138.3
-138.4
-138.4
-138.4
-138.4
-138.4
-149.1
-149.1
-149.1
-149.1
-149.1
-149.2
-149.2
-149.2
-149.2
-160.9
-160.9
-160.9
-161.0
-161.0
-161.0
-161.0
-161.0
-161.0
-161.1
2.1
1.1
2.1
-10 -20 -30 -40 -50 -60 -70 -80 -90 -100 -110 -120 -130 -140 -150 -160 -170 -180 -190
f1 (ppm)

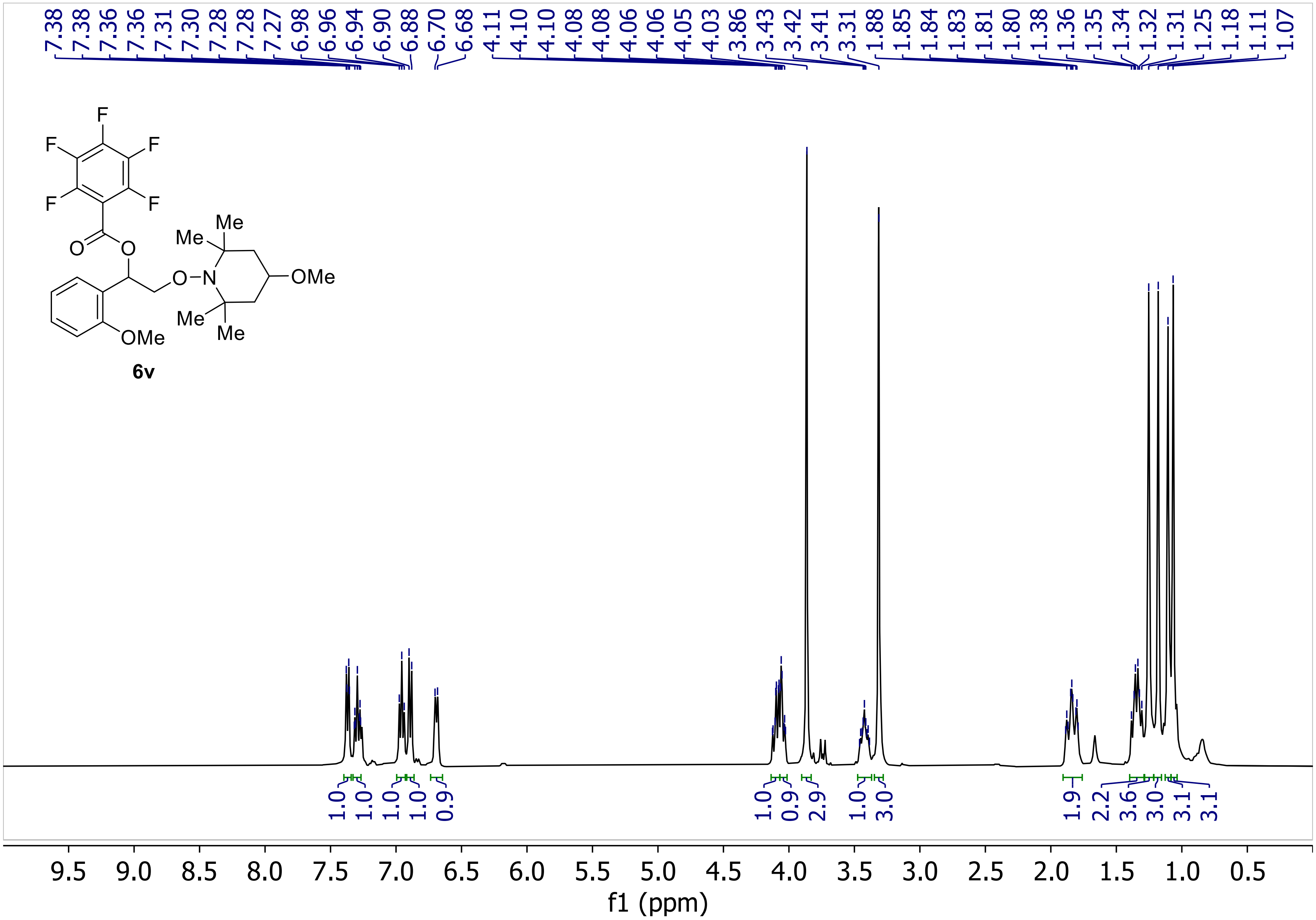
F
F
F
F
F
O
O
O
N
Me
Me
Me
Me
OMe
OMe
6v
7.38
7.38
7.36
7.36
7.31
7.30
7.28
7.28
7.27
6.98
6.96
6.94
6.90
6.88
6.70
6.68
4.11
4.10
4.10
4.08
4.08
4.06
4.06
4.05
4.03
3.86
3.43
3.42
3.41
3.31
1.88
1.85
1.84
1.83
1.81
1.80
1.38
1.36
1.35
1.34
1.32
1.31
1.25
1.18
1.11
1.07
1.0
1.0
1.0
1.0
0.9
1.0
0.9
2.9
1.0
3.0
1.9
2.2
3.6
3.0
3.1
3.1
9.5
9.0
8.5
8.0
7.5
7.0
6.5
6.0
5.5
5.0
4.5
4.0
3.5
3.0
2.5
2.0
1.5
1.0
0.5
f1 (ppm)

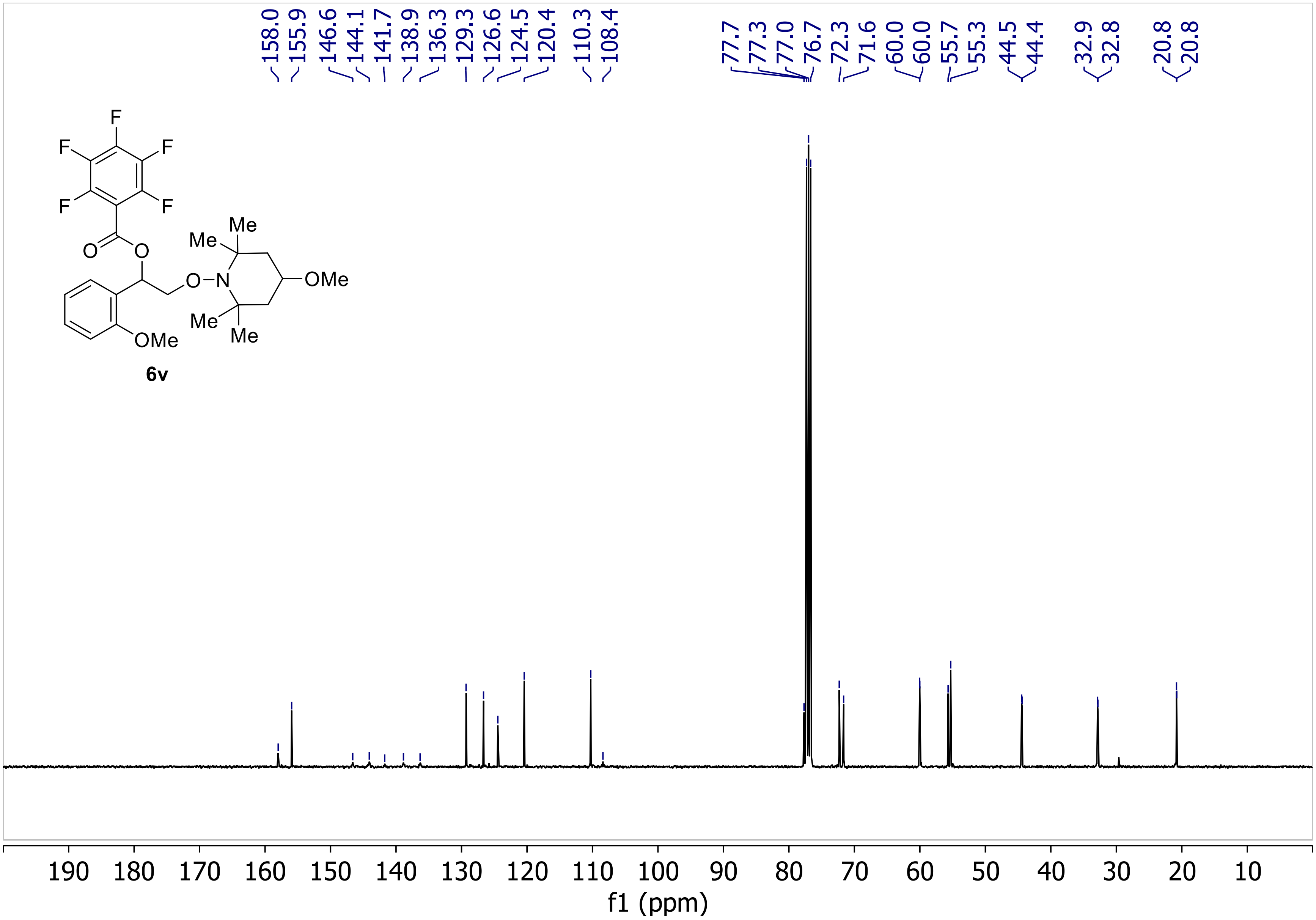

158.0
155.9
146.6
144.1
141.7
138.9
136.3
129.3
126.6
124.5
120.4
110.3
108.4
77.7
77.3
77.0
76.7
72.3
71.6
60.0
60.0
55.7
55.3
44.5
44.4
32.9
32.8
20.8
20.8
F
F
F
F
F
O
O
O
N
Me
Me
Me
Me
OMe
OMe
190
180
170
160
150
140
130
120
110
100
90
80
70
60
50
40
30
20
10
f1 (ppm)

6v

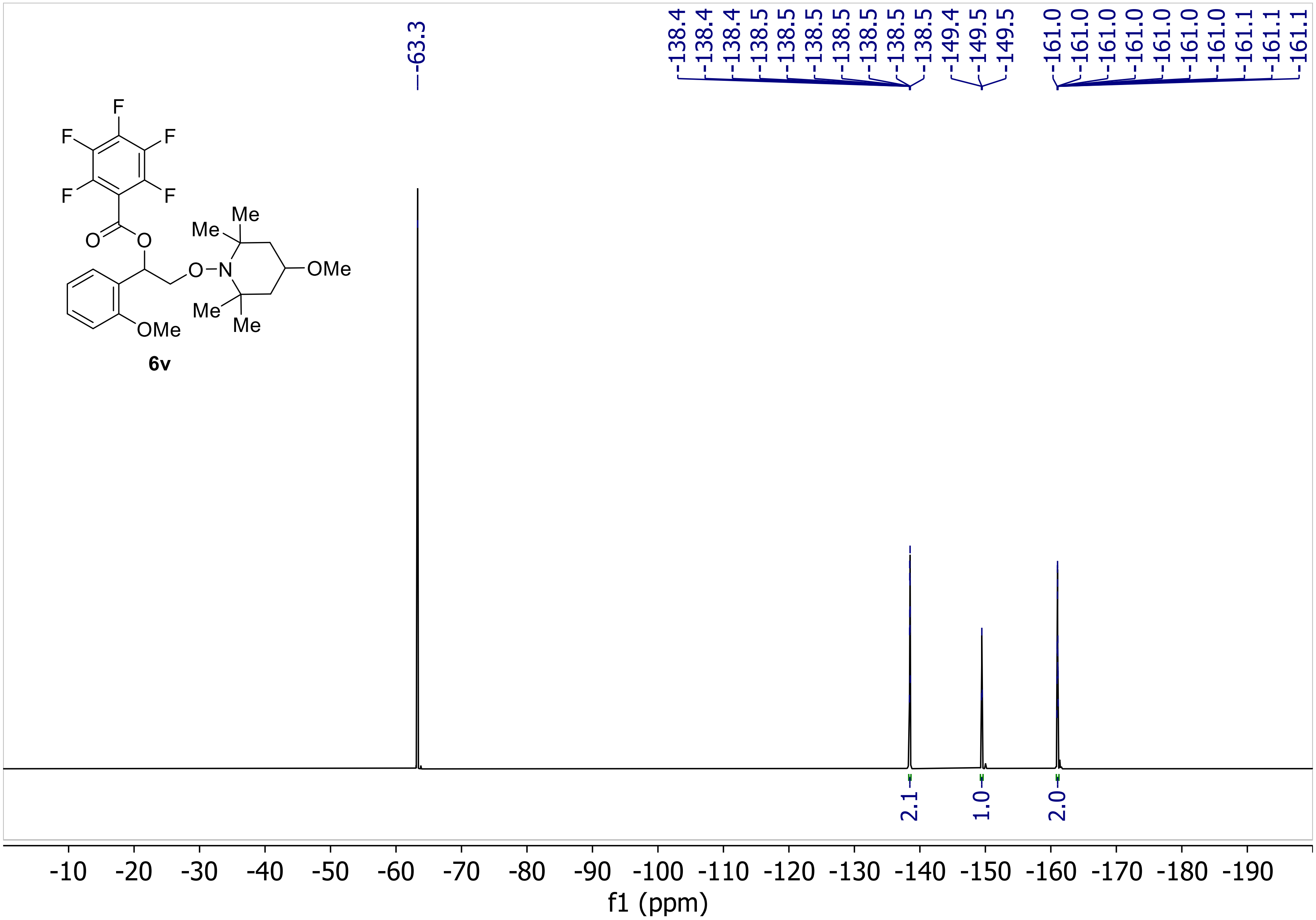
F
F
F
F
F
O
O
Me
Me
O–N
OMe
Me
Me
OMe
6v
-63.3
-138.4
-138.4
-138.4
-138.5
-138.5
-138.5
-138.5
-138.5
-138.5
-138.5
-149.4
-149.5
-149.5
-161.0
-161.0
-161.0
-161.0
-161.0
-161.0
-161.0
-161.1
-161.1
-161.1
2.1
1.0
2.0
-10 -20 -30 -40 -50 -60 -70 -80 -90 -100 -110 -120 -130 -140 -150 -160 -170 -180 -190
f1 (ppm)

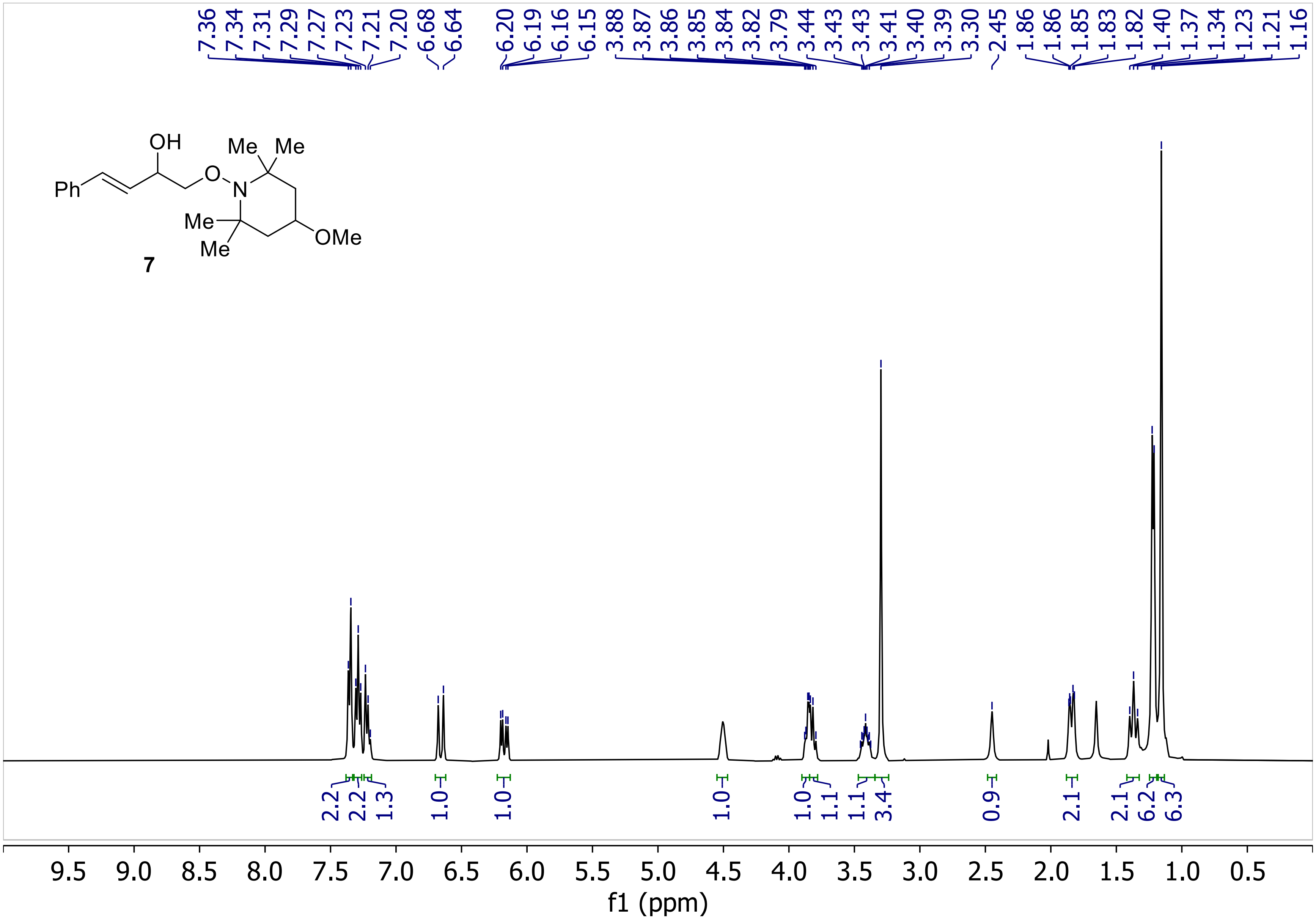
7.36
7.34
7.31
7.29
7.27
7.23
7.21
7.20
6.68
6.64
6.20
6.19
6.16
6.15
3.88
3.87
3.86
3.85
3.84
3.82
3.79
3.44
3.43
3.43
3.41
3.40
3.39
3.30
2.45
1.86
1.86
1.85
1.83
1.82
1.40
1.37
1.34
1.23
1.21
1.16
OH
Me
Me
Ph
O
N
Me
Me
OMe
7
2.2
2.2
1.3
1.0
1.0
1.0
1.0
1.1
1.1
3.4
0.9
2.1
2.1
6.2
6.3
9.5
9.0
8.5
8.0
7.5
7.0
6.5
6.0
5.5
5.0
4.5
4.0
3.5
3.0
2.5
2.0
1.5
1.0
0.5
f1 (ppm)

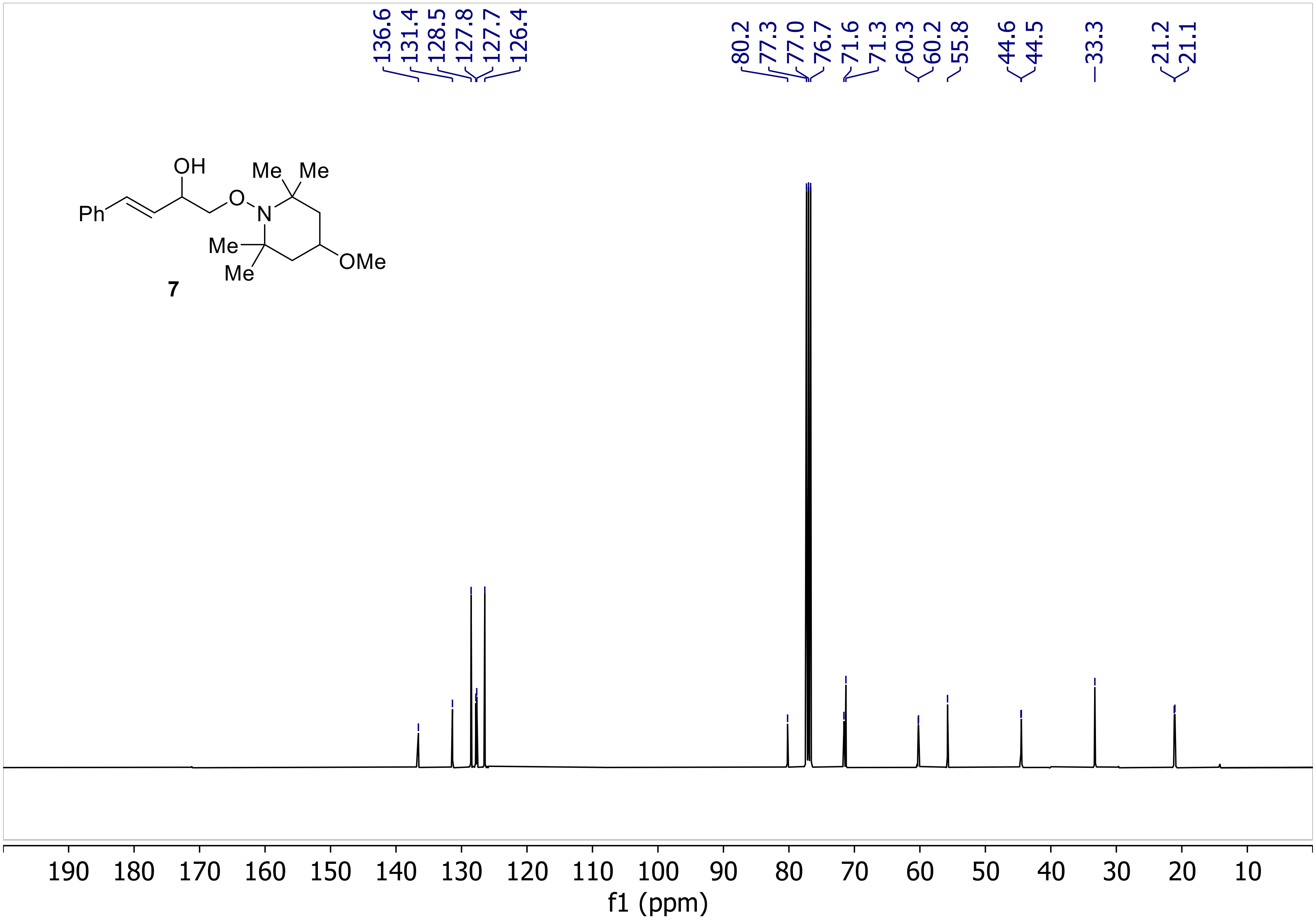
136.6
131.4
128.5
127.8
127.7
126.4
80.2
77.3
77.0
76.7
71.6
71.3
60.3
60.2
55.8
44.6
44.5
33.3
21.2
21.1
OH
Me
Me
O
N
Ph
Me
Me
OMe
7
190
180
170
160
150
140
130
120
110
100
90
80
70
60
50
40
30
20
10
f1 (ppm)

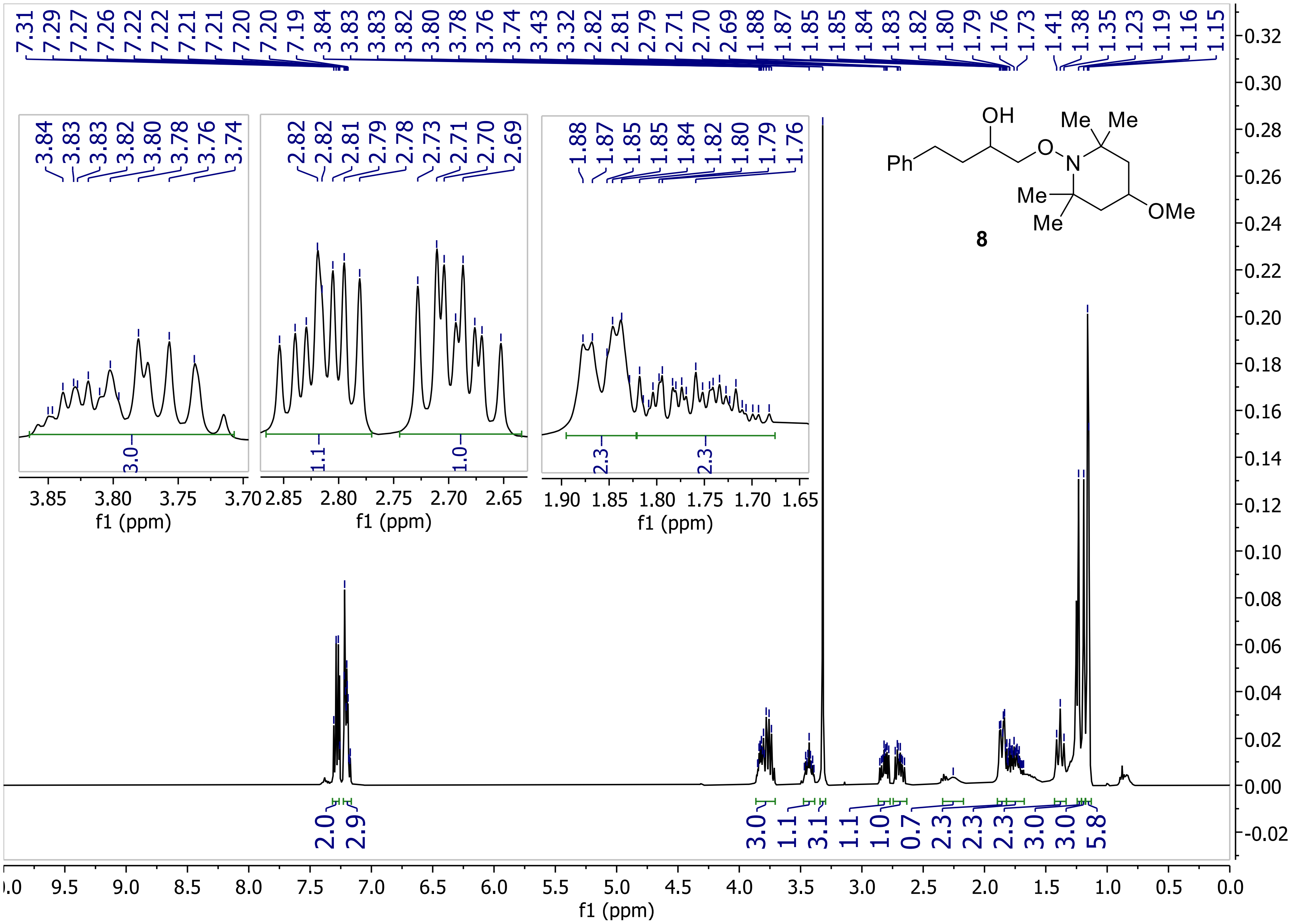
OH
Me
Me
Ph
O
N
Me
Me
OMe
8
f1 (ppm)

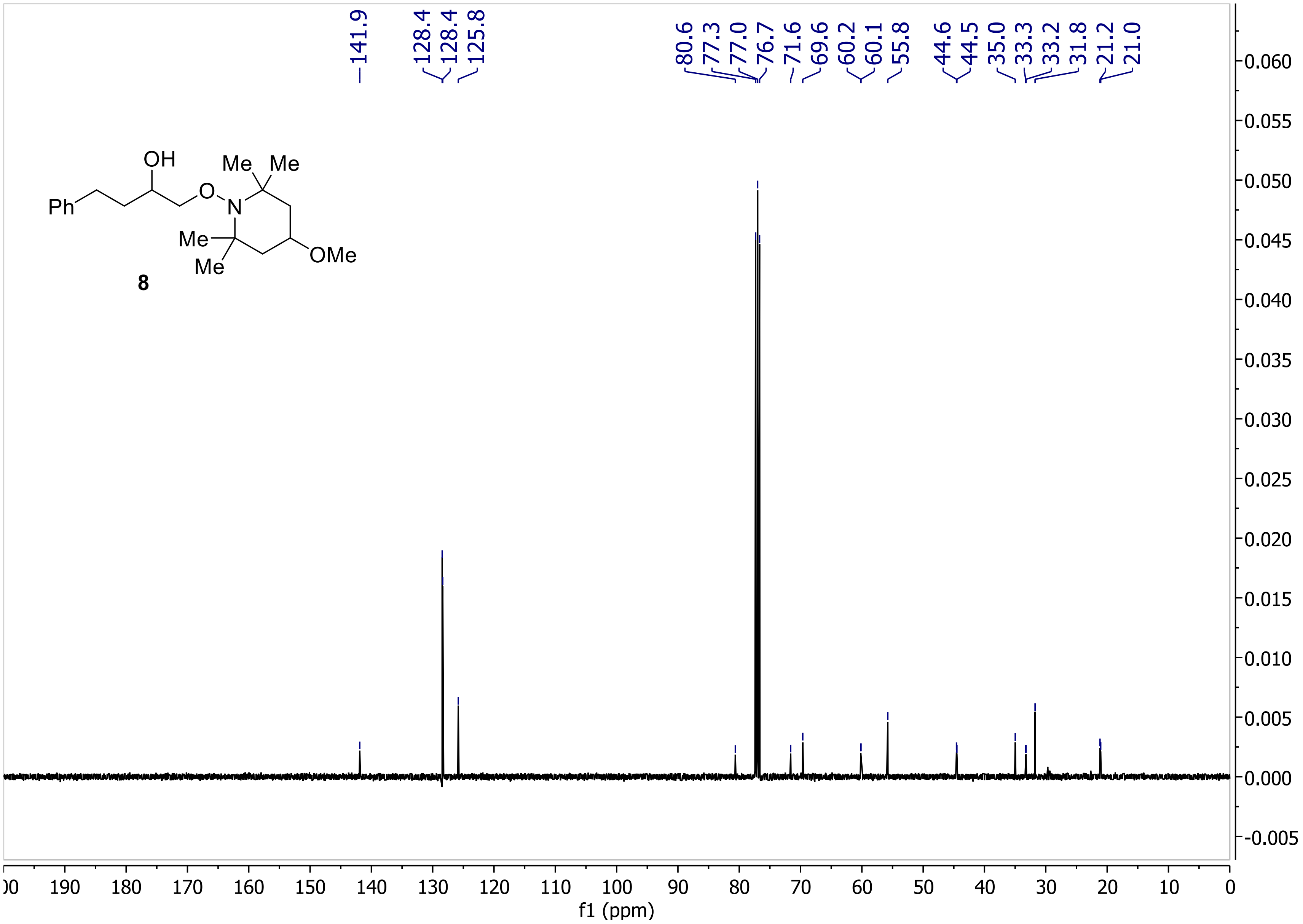
141.9
128.4
128.4
125.8
80.6
77.3
77.0
76.7
71.6
69.6
60.2
60.1
55.8
44.6
44.5
35.0
33.3
33.2
31.8
21.2
21.0
OH
Me Me
Ph
O
N
Me
Me
OMe
8
f1 (ppm)

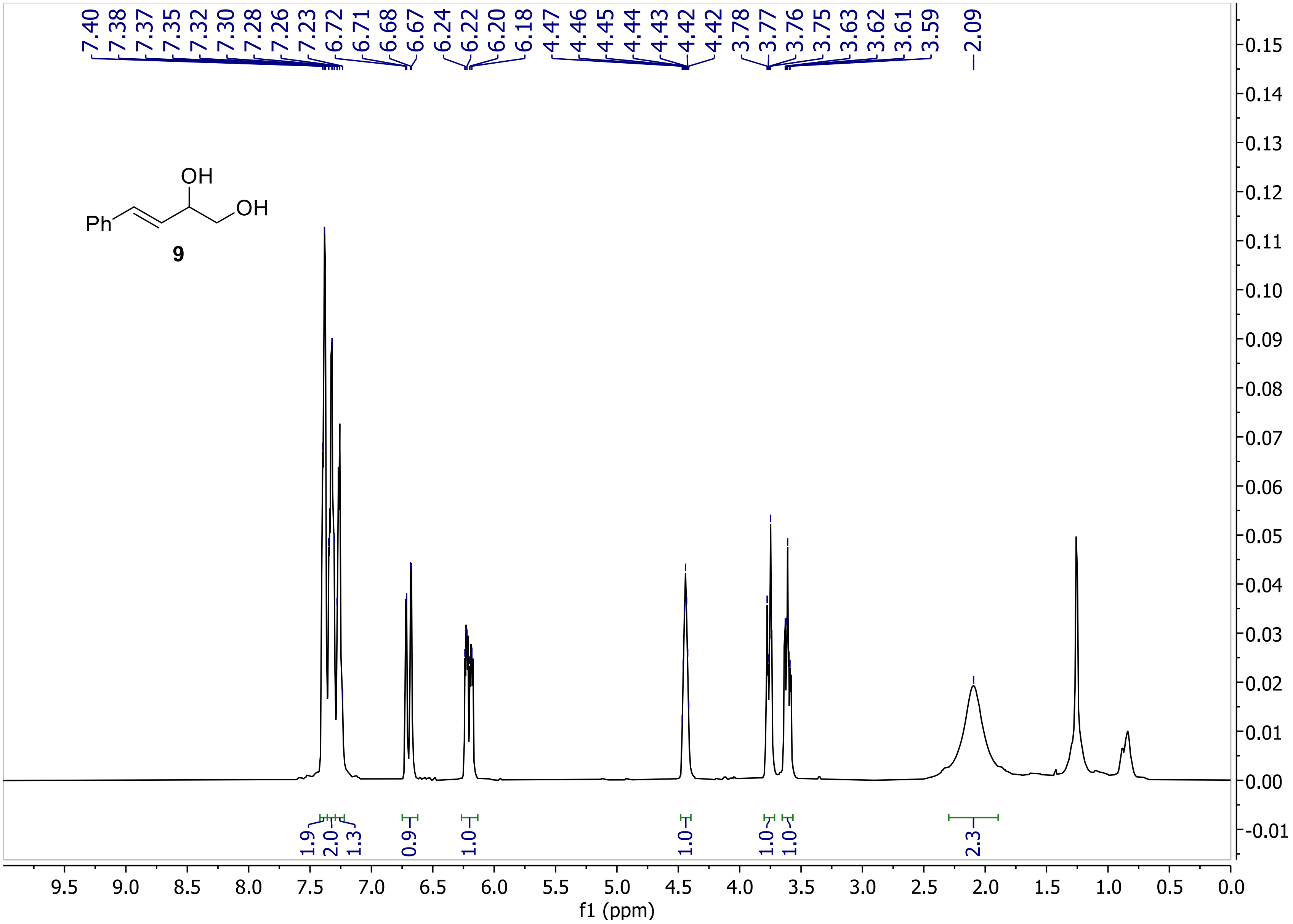
OH
OH
Ph
9
7.40 7.38 7.37 7.35 7.32 7.30 7.28 7.26 7.23 6.72 6.71 6.68 6.67 6.24 6.22 6.20 6.18 4.47 4.46 4.45 4.44 4.43 4.42 4.42 3.78 3.77 3.76 3.75 3.63 3.62 3.61 3.59 2.09
1.9 2.0 1.3 0.9 1.0 1.0 1.0 1.0 2.3
f1 (ppm)

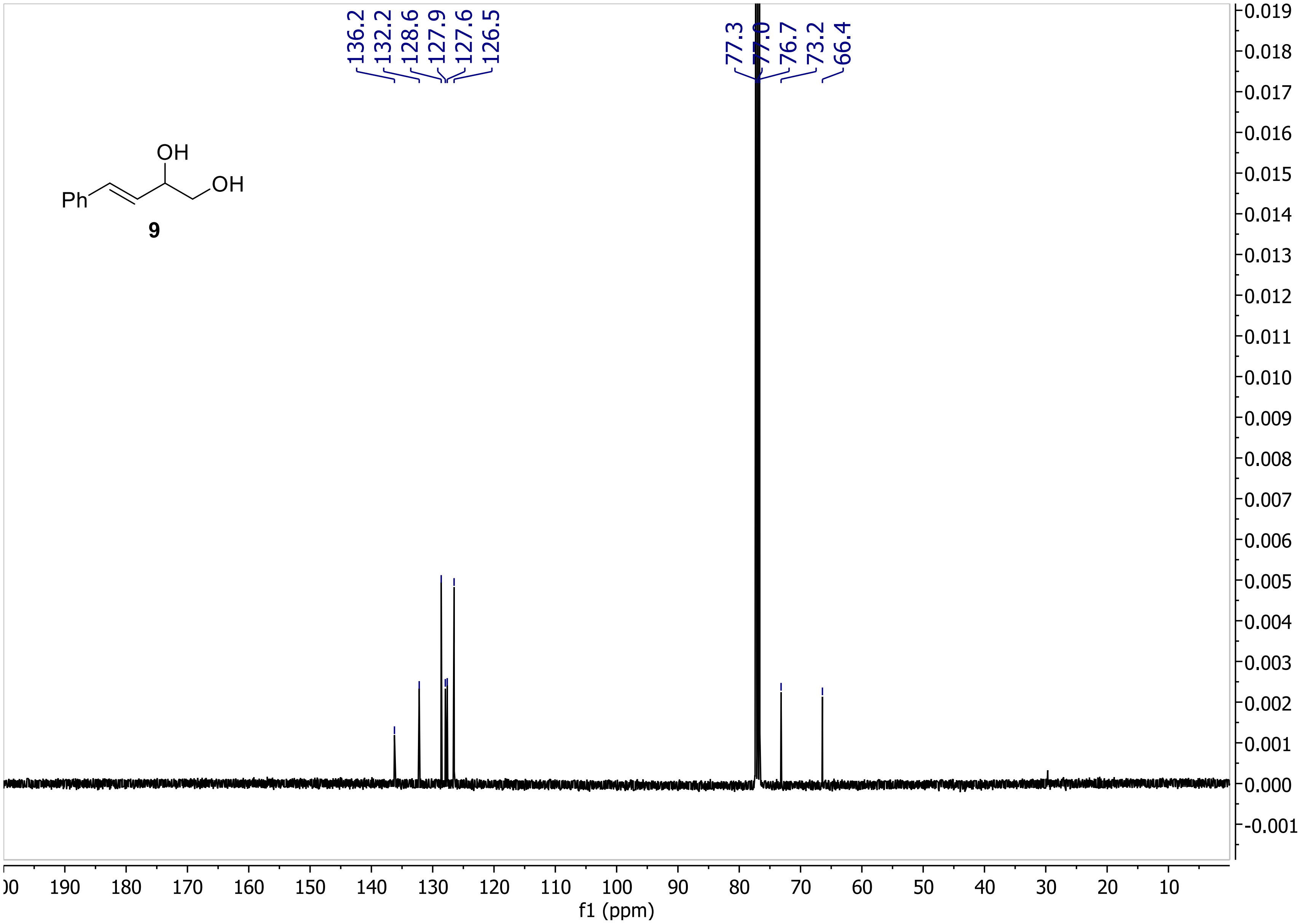
136.2
132.2
128.6
127.9
127.6
126.5
77.3
77.0
76.7
73.2
66.4
OH
OH
Ph
9
0.019
0.018
0.017
0.016
0.015
0.014
0.013
0.012
0.011
0.010
0.009
0.008
0.007
0.006
0.005
0.004
0.003
0.002
0.001
0.000
-0.001
190
180
170
160
150
140
130
120
110
100
90
80
70
60
50
40
30
20
10
f1 (ppm)

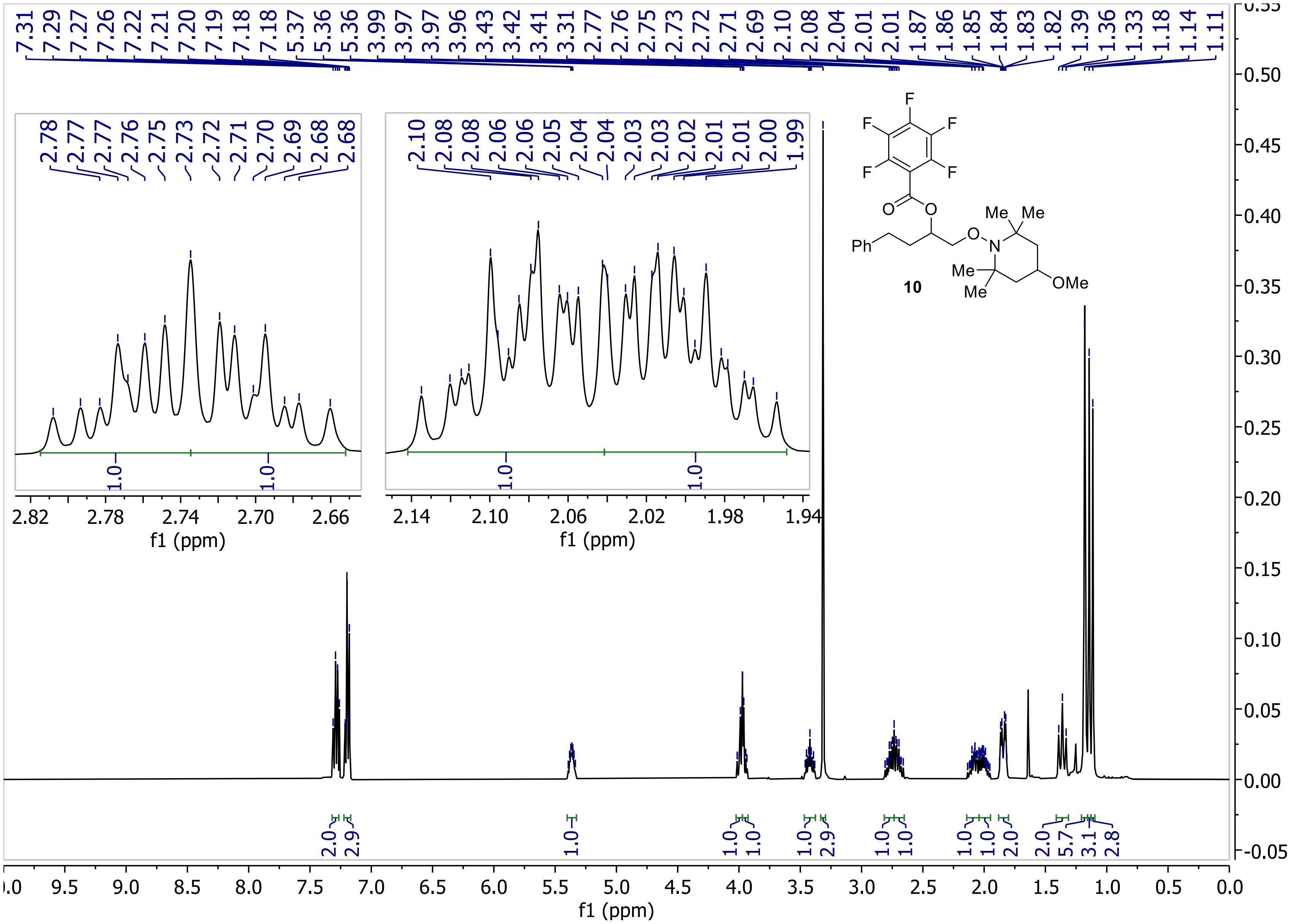
7.31 7.29 7.27 7.26 7.22 7.21 7.20 7.19 7.18 7.18 5.37 5.36 5.36 3.99 3.97 3.97 3.96 3.43 3.42 3.41 3.31 2.77 2.76 2.75 2.73 2.72 2.71 2.69 2.10 2.08 2.04 2.01 2.01 1.87 1.86 1.85 1.84 1.83 1.82 1.39 1.36 1.33 1.18 1.14 1.11
2.78 2.77 2.77 2.76 2.75 2.73 2.72 2.71 2.70 2.69 2.68 2.68
2.10 2.08 2.08 2.06 2.06 2.05 2.04 2.04 2.03 2.03 2.02 2.01 2.01 2.00 1.99
F
F
F
F
F
O
O
Ph
O
N
Me
Me
Me
Me
OMe
10
f1 (ppm)
f1 (ppm)
f1 (ppm)

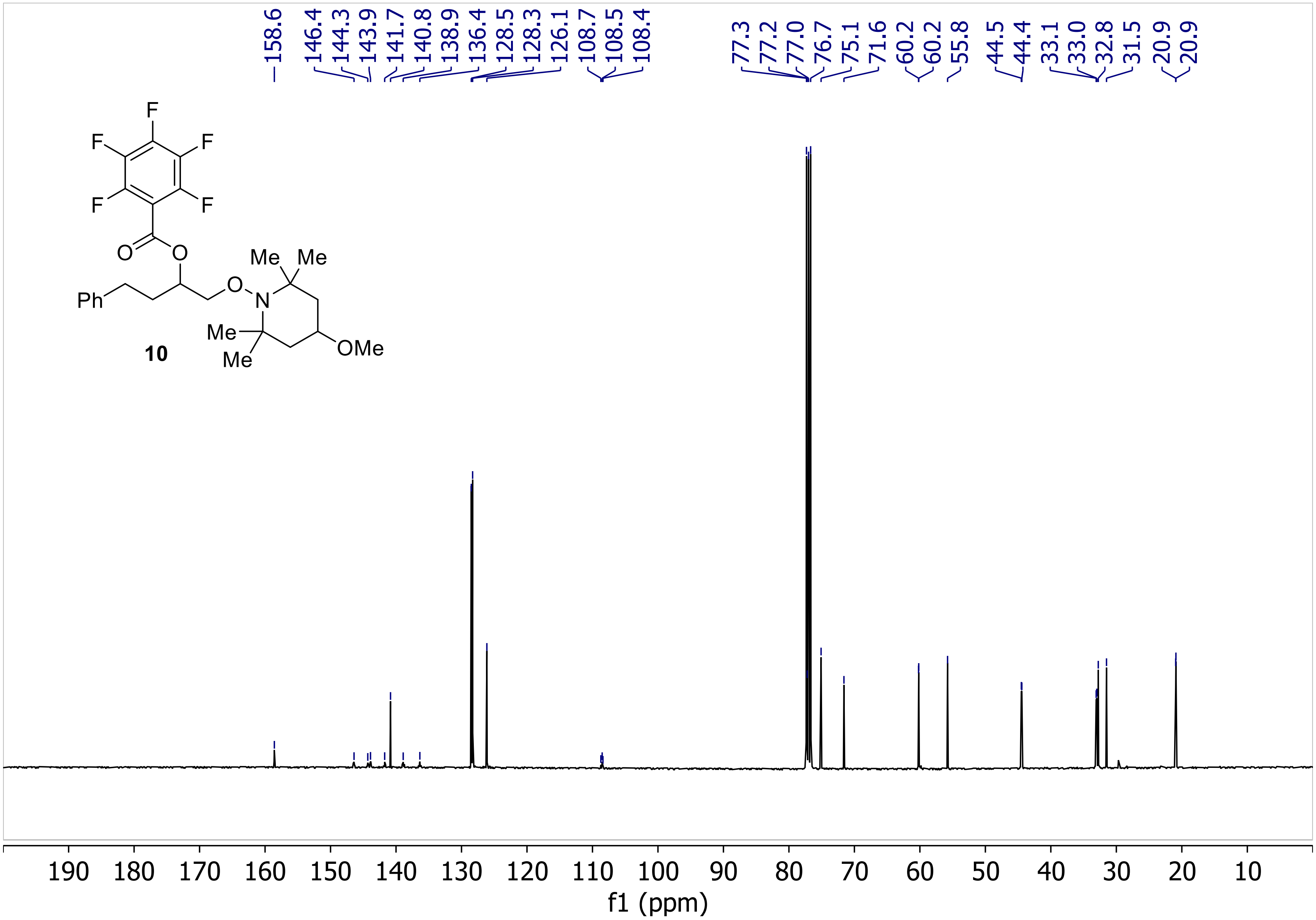
158.6
146.4
144.3
143.9
141.7
140.8
138.9
136.4
128.5
128.3
126.1
108.7
108.5
108.4
77.3
77.2
77.0
76.7
75.1
71.6
60.2
60.2
55.8
44.5
44.4
33.1
33.0
32.8
31.5
20.9
20.9
F
F
F
F
F
O
O
Me
Me
O
N
Ph
Me
Me
OMe
10
190
180
170
160
150
140
130
120
110
100
90
80
70
60
50
40
30
20
10
f1 (ppm)

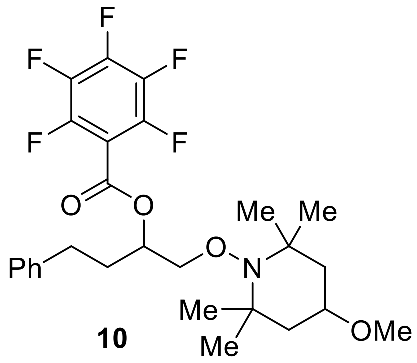
F
F
F
F
F
O
O
Ph
O
N
Me
Me
Me
Me
OMe

10

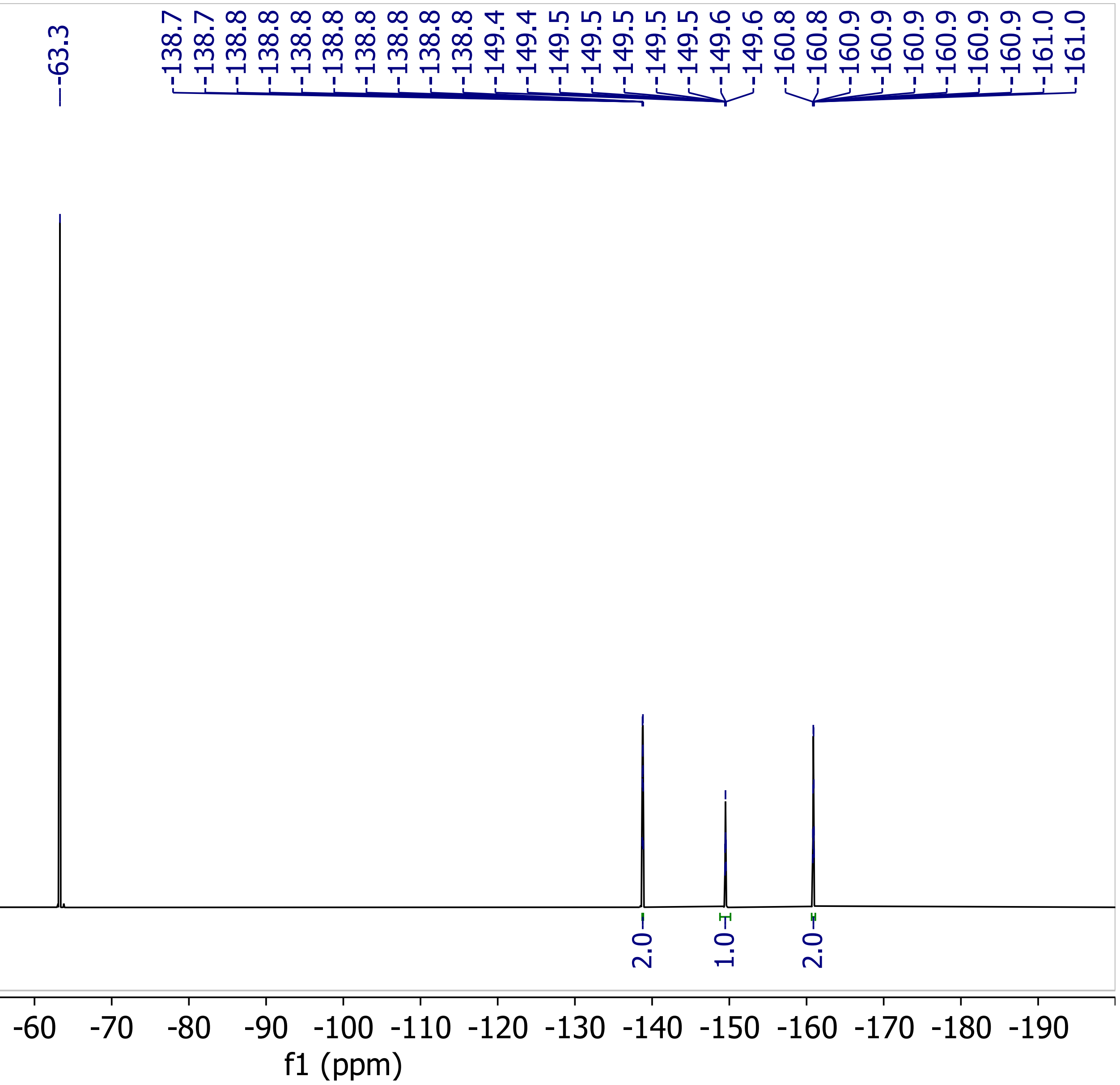
-63.3
-138.7
-138.7
-138.8
-138.8
-138.8
-138.8
-138.8
-138.8
-138.8
-138.8
-149.4
-149.4
-149.5
-149.5
-149.5
-149.5
-149.5
-149.6
-149.6
-160.8
-160.8
-160.9
-160.9
-160.9
-160.9
-160.9
-160.9
-161.0
-161.0
2.0
1.0
2.0
-10 -20 -30 -40 -50 -60 -70 -80 -90 -100 -110 -120 -130 -140 -150 -160 -170 -180 -190
f1 (ppm)

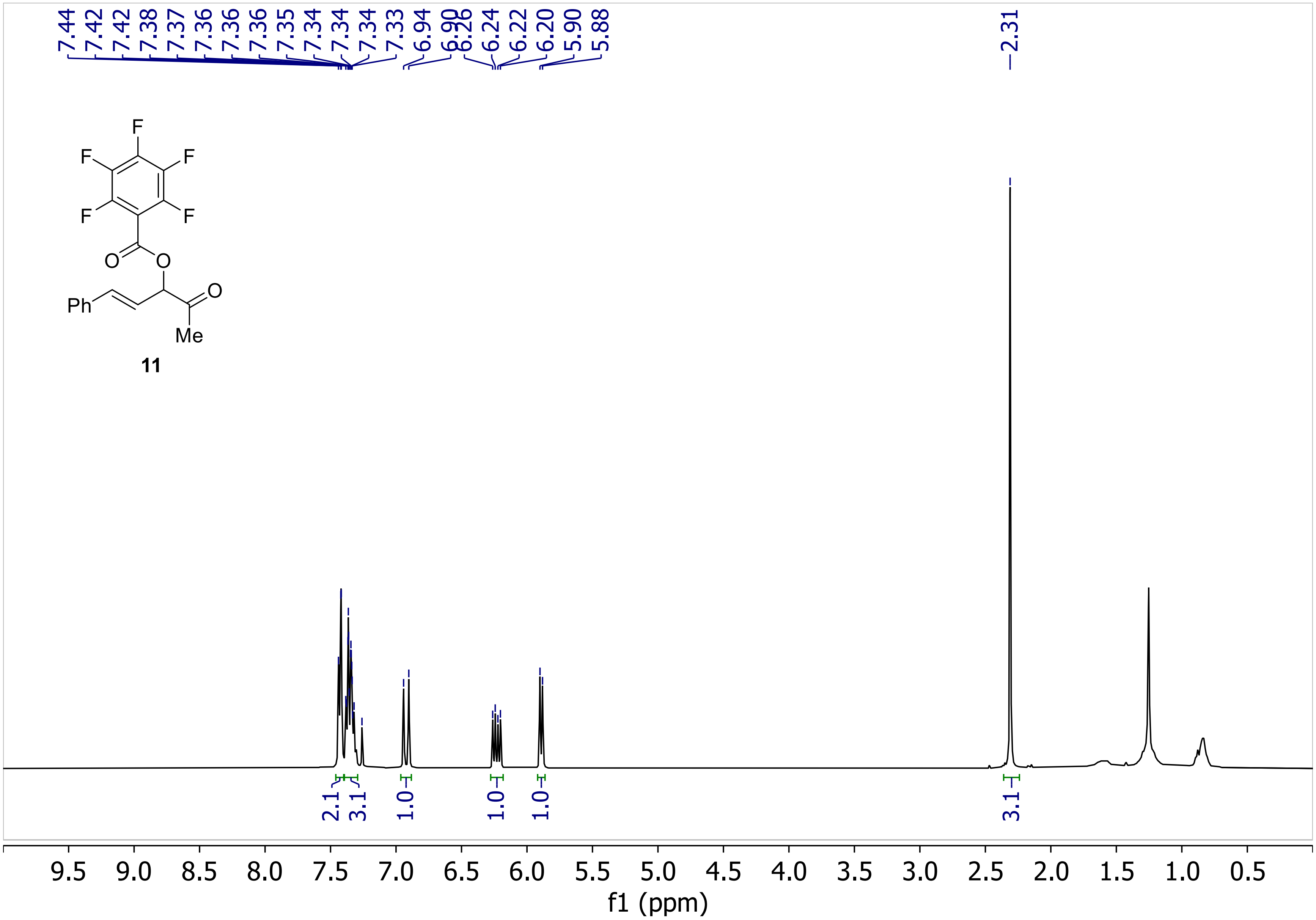
7.44
7.42
7.42
7.38
7.37
7.36
7.36
7.36
7.35
7.34
7.34
7.34
7.33
6.94
6.90
6.26
6.24
6.22
6.20
5.90
5.88
2.31
F
F
F
F
F
O
O
O
Ph
Me
11
2.1
3.1
1.0
1.0
1.0
3.1
9.5
9.0
8.5
8.0
7.5
7.0
6.5
6.0
5.5
5.0
4.5
4.0
3.5
3.0
2.5
2.0
1.5
1.0
0.5
f1 (ppm)

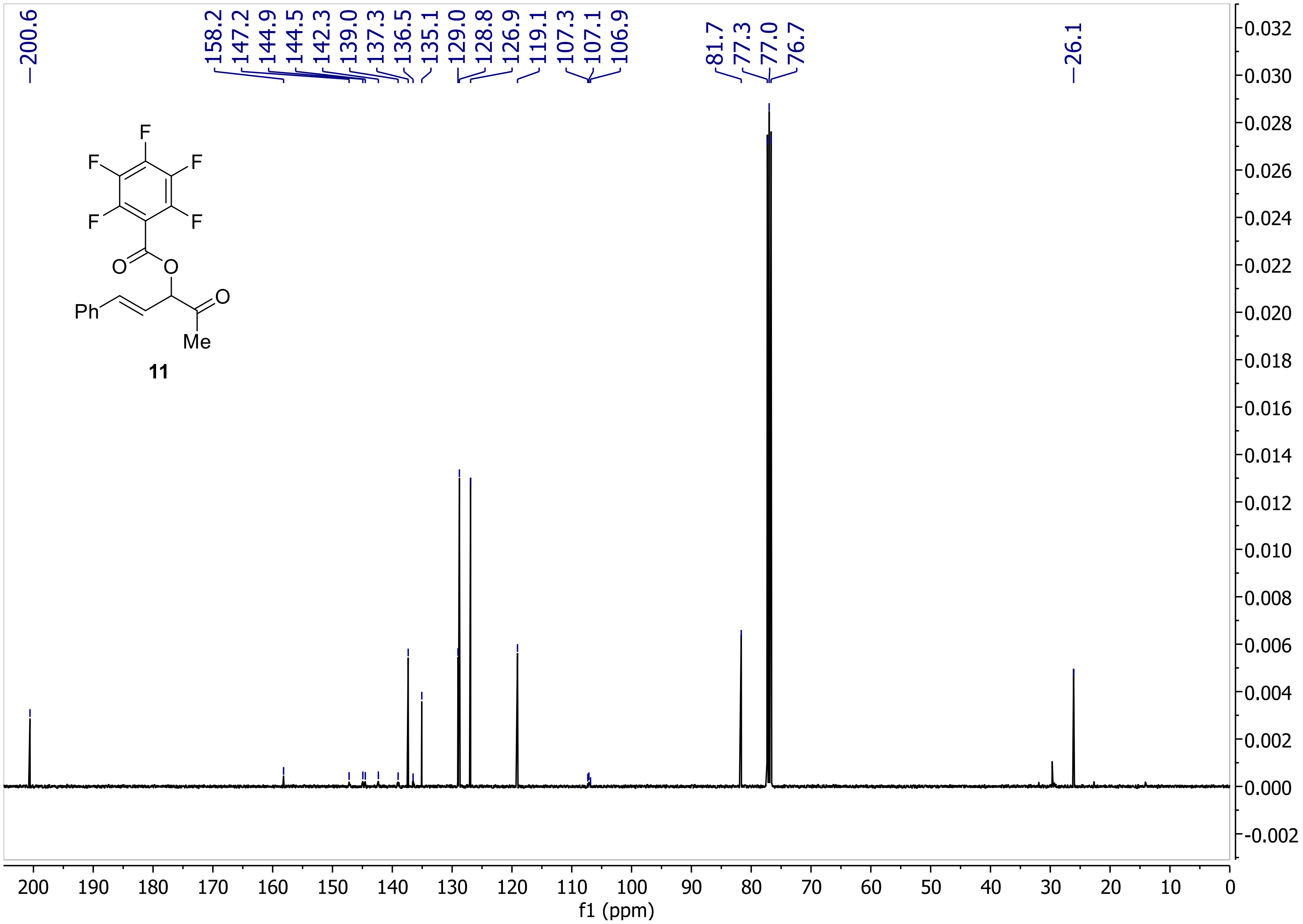

200.6
158.2
147.2
144.9
144.5
142.3
139.0
137.3
136.5
135.1
129.0
128.8
126.9
119.1
107.3
107.1
106.9
81.7
77.3
77.0
76.7
26.1
F
F
F
F
F
O
O
O
Ph
Me
11
f1 (ppm)

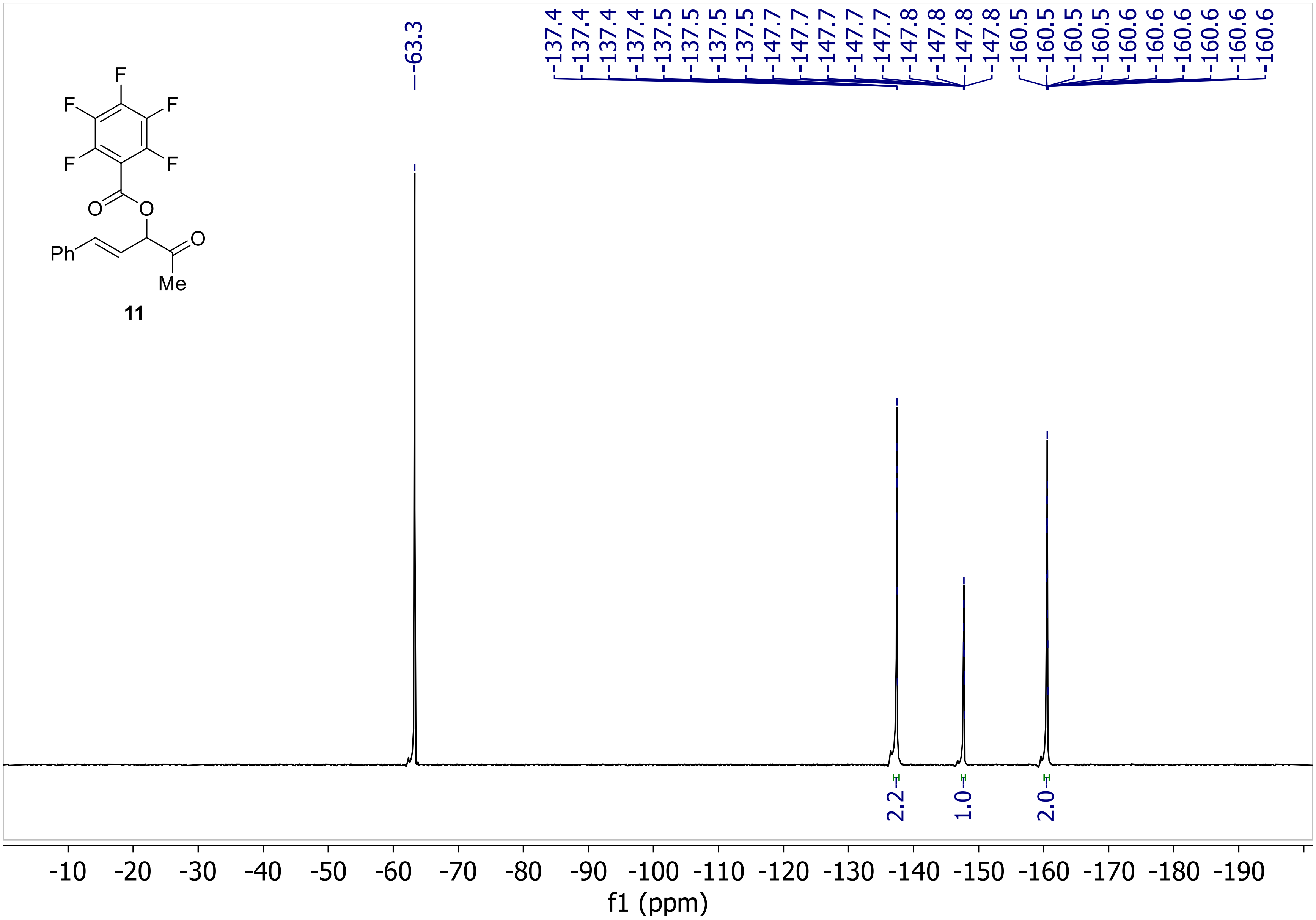

F
F
F
F
F
O
O
O
Ph
Me
11
-63.3
-137.4
-137.4
-137.4
-137.4
-137.5
-137.5
-137.5
-137.5
-147.7
-147.7
-147.7
-147.7
-147.7
-147.8
-147.8
-147.8
-147.8
-160.5
-160.5
-160.5
-160.5
-160.6
-160.6
-160.6
-160.6
-160.6
-160.6
2.2
1.0
2.0
-10 -20 -30 -40 -50 -60 -70 -80 -90 -100 -110 -120 -130 -140 -150 -160 -170 -180 -190
f1 (ppm)

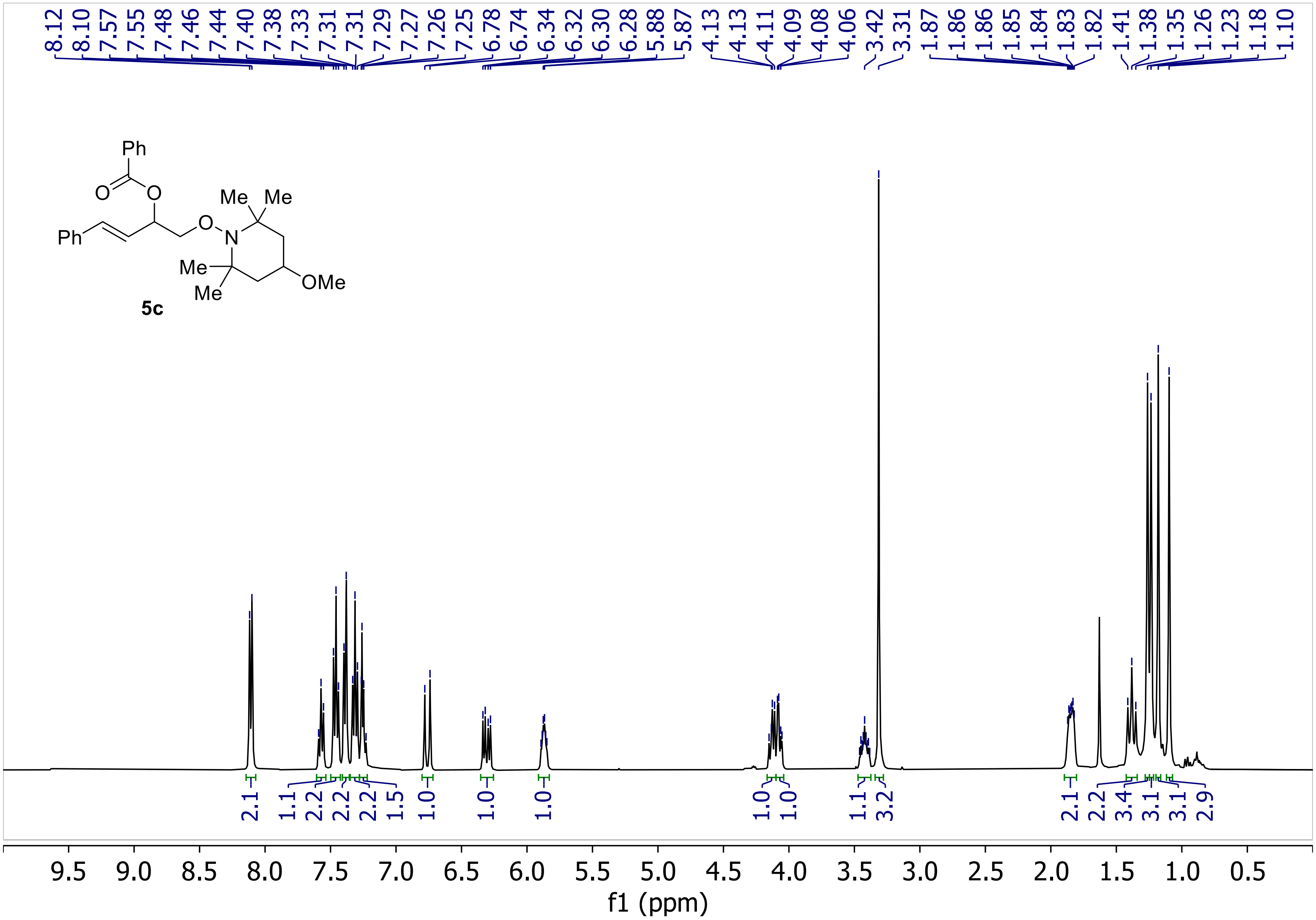
Ph
O
O
Me
Me
Ph
O
N
Me
Me
OMe
5c
8.12
8.10
7.57
7.55
7.48
7.46
7.44
7.40
7.38
7.33
7.31
7.31
7.29
7.27
7.26
7.25
6.78
6.74
6.34
6.32
6.30
6.28
5.88
5.87
4.13
4.13
4.11
4.09
4.08
4.06
3.42
3.31
1.87
1.86
1.86
1.85
1.84
1.83
1.82
1.41
1.38
1.35
1.26
1.23
1.18
1.10
2.1
1.1
2.2
2.2
2.2
1.5
1.0
1.0
1.0
1.0
1.0
1.1
3.2
2.1
2.2
3.4
3.1
3.1
2.9
9.5
9.0
8.5
8.0
7.5
7.0
6.5
6.0
5.5
5.0
4.5
4.0
3.5
3.0
2.5
2.0
1.5
1.0
0.5
f1 (ppm)

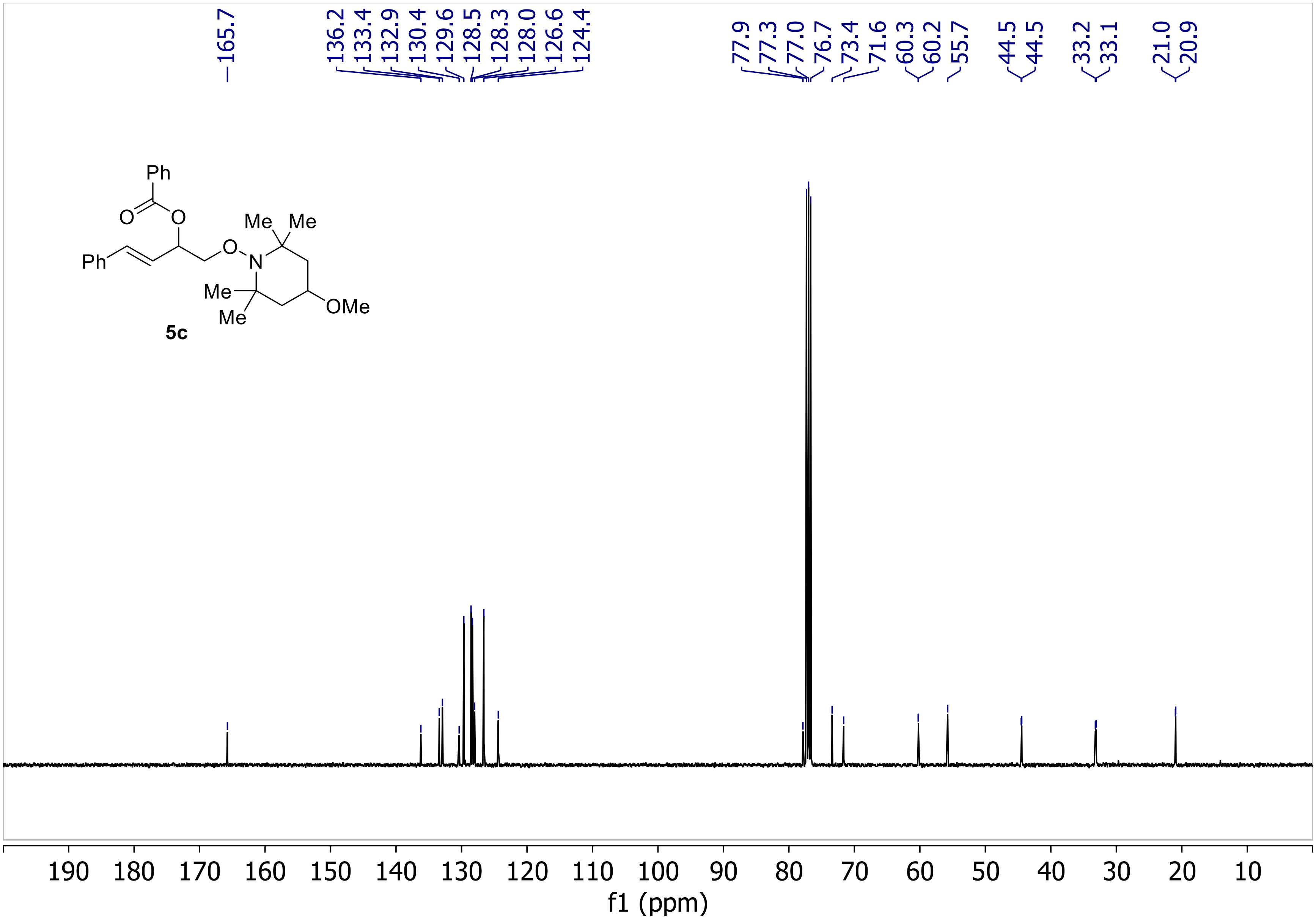
165.7
136.2
133.4
132.9
130.4
129.6
128.5
128.3
128.0
126.6
124.4
77.9
77.3
77.0
76.7
73.4
71.6
60.3
60.2
55.7
44.5
44.5
33.2
33.1
21.0
20.9
Ph
O
O
Ph
O
N
Me
Me
Me
Me
OMe
5c
190
180
170
160
150
140
130
120
110
100
90
80
70
60
50
40
30
20
10
f1 (ppm)
